# Identification of a Five-Gene Prognostic Model and Its Potential Drug Repurposing in Colorectal Cancer Based on TCGA, GTEx and GEO Databases

**DOI:** 10.3389/fgene.2020.622659

**Published:** 2021-01-18

**Authors:** Feng Yang, Shaoyi Cai, Li Ling, Haiji Zhang, Liang Tao, Qin Wang

**Affiliations:** Zhongshan School of Medicine, Sun Yat-Sen University, Guangzhou, China

**Keywords:** colorectal cancer, mechanisms, prognosis, drug-repurposing, WGCNA

## Abstract

Colorectal cancer (CRC) is a major cause of cancer deaths worldwide. Unfortunately, many CRC patients are still being diagnosed at an advanced stage of the cancer, and the 5-year survival rate is only ~30%. Effective prognostic markers of CRC are therefore urgently needed. To address this issue, we performed a detailed bioinformatics analysis based on the Cancer Genome Atlas (TCGA), Genotype-Tissue Expression (GTEx), and Gene Expression Omnibus (GEO) databases to identify prognostic biomarkers for CRC, which in turn help in exploring potential drug-repurposing. We identified five hub genes (PGM2, PODXL, RHNO1, SCD, and SEPHS1), which had good performance in survival prediction and might be involved in CRC through three key pathways (“Cell cycle,” “Purine metabolism,” and “Spliceosome” KEGG pathways) identified by a KEGG pathway enrichment analysis. What is more, we performed a co-expression analysis between five hub genes and transcription factors to explore the upstream regulatory region. Furthermore, we screened the potential drug-repurposing for the five hub genes in CRC according to the Binding DB and ZINC15 databases. Taking together, we constructed a five-gene signature to predict overall survival of CRC and found the potential drug-repurposing, which may improve the outcome of CRC in the future.

## Introduction

Colorectal cancer (CRC) is one of the most common types of tumors and is the third leading cause of death among cancer patients worldwide (Siegel et al., 2020). Since the prognosis of CRC mainly depends on the clinicopathological features or the tumor stage (Messersmith, 2019), many patients are still diagnosed at advanced stages, and the 5-year survival rate is only ~30% (Siegel et al., 2020). Therefore, a better understanding of the molecular mechanism, to identify new promising prognostic biomarkers, is essential for the development of effective treatment strategies in CRC.

Recent studies have documented some prognostic related biomarkers in CRC. For example, upregulated CIP2A could contribute to tumor cell survival and a poor prognosis in CRC (Liu et al., 2018). Moreover, overexpression of HOXB13 in CRC could inhibit the tumor growth and be related to the poor outcome (Xie et al., 2019). Additionally, the expression of HOXB9 could promote metastasis and indicate a poor prognosis in CRC (Huang et al., 2014). However, the single biomarker might not be enough to predict the outcome of CRC, due to the large individual differences. To address this question, some multi-gene signatures have been identified which help in predicting the cancer prognosis outcomes (Deng et al., 2019; Yang et al., 2020). However, these findings were constructed based on the differently expressed genes (DEGs) or on small datasets. It is well-known that DEGs are screened using an artificially set threshold, which would exclude some important prognostic genes. Therefore, a systematic analysis of mRNA expression with a large sample size using an unsupervised analysis method may help in identifying more effective prognostic signatures in CRC.

In this study, we aimed to confirm a detailed molecular mechanism to identify the prognostic genes and potential drug-repurposing for CRC, which might provide preliminary bioinformatic evidence to better understand the complex mechanism of CRC progression and which might help improve the outcome of CRC.

## Materials and Methods

### Data Collection

In order to maximize the sample size, the data involved in the analysis was combined with the Cancer Genome Atlas (TCGA) and the Genotype-Tissue Expression (GTEx) databases. The Fragments Per Kilobase Million (FPKM) values of the mRNA expression profile and clinical traits, containing 471 colorectal cancer (CRC) and 349 normal control samples, were downloaded from TCGA and GTEx. The GSE17536 dataset (Freeman et al., 2012) was downloaded from the Gene Expression Omnibus (GEO) database, containing 177 CRC samples, to further validate independently of the analysis.

### Study Design

**Figure 1:**
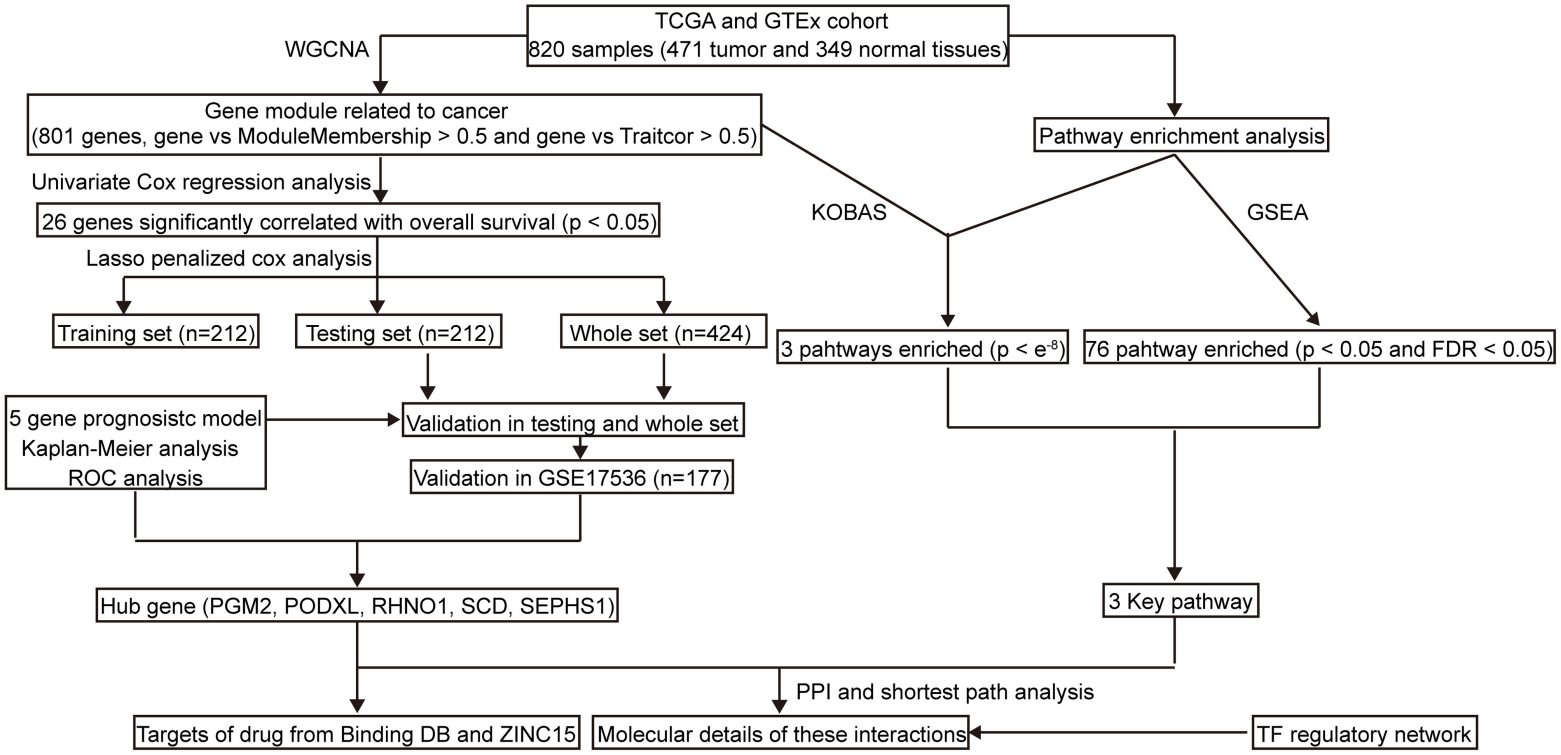
The workflow of the study. TCGA, The Cancer Genome Atlas; GEO, Gene Expression Omnibus; GTEx, Genotype-Tissue Expression; KEGG, Kyto Encyclopedia of Genes and Genomes; WGCNA, Weighted correlation network analysis; GSEA, Gene Set Enrichment Analysis; PPI, Protein-protein interaction; ROC, Receiver operating characteristic; TF, Transcription factor.

The workflow of this study is shown in Figure 1. Briefly, we applied Weighted Correlation Network Analysis (WGCNA) to screen the genes that are significantly associated with CRC. Subsequently, we employed Cox regression to build a five-gene prognostic signature. To evaluate the prognostic model, we then performed the Kaplan–Meier survival curve, the time-dependent receiver operating characteristic (ROC), and the area under the curve (AUC). Furthermore, to explore the molecular mechanism of five hub genes involved in CRC, we performed a pathway enrichment. We also identified the interactions among the hub genes and core genes involved in key pathways using the Protein-protein interaction (PPI) and shortest path analysis. Furthermore, transcription factors (TFs)-mRNA's regulatory network was constructed by a co-expression analysis. Lastly, we elucidated potential drug-repurposing for five genes according to the Binding DB database and structure-based virtual screening from the ZINC15 database.

### Identification of Genes Related to CRC by WGCNA

To obtain the modules related to CRC, WGCNA was performed by R package WGCNA (Langfelder and Horvath, 2012). First, to improve data analysis efficiency, we selected 6,442 genes from the TCGA and GTEx databases for the analysis. These genes were in the top 35% of median absolute deviation. Second, the power value β was selected to determine a scale-free topology model. The adjacency matrix was then transformed into the Topological Overlap Matrix (TOM) to minimize effects of noise and spurious associations. TOM-based dissimilarity was used to form modules by a dynamic tree cutoff (minModuleSize = 30, mergeCutHeight = 0.2 and deepSplit = 3). Finally, according to a previous study (Qiu et al., 2020), the CRC-associated module was identified with the cutoff of the highest absolute correlation values and *p* < 0.05. Furthermore, the correlated genes with module-membership > 0.5 and CRC-correlation value > 0.5, were thought to be putative genes related to CRC.

### Establishment of the Prognostic Model and Validation

CRC patients with an overall survival of <30 days were excluded for the construction of the prognostic model. To select prognostic genes, we applied Univariate Cox regression analysis by R package survival (https://github.com/therneau/survival) with a cut-off of *p* < 0.05. The whole data set was randomly separated into the training dataset and the test dataset through R package caret (https://github.com/topepo/caret). Afterwards, we employed the Lasso-penalized Cox regression model to further select the most useful prognostic markers. We then built the prognostic model using a linear combination of the regression coefficient coming from the Lasso Cox regression model coefficients (β), multiplied with its mRNA expression value. According to the expression of the outcome-related genes and the coefficient in the prognosis model, we calculated the risk score for each sample. Subsequently, samples were separated into a low-risk or high-risk group based on the median value of the risk score. Finally, we used the R package survivalROC (Heagerty et al., 2000) to evaluate the prognostic performance of the model using the ROC curve. Second, we performed the Kaplan–Meier survival curve and a log-rank test to assess the survival difference by the R package survival. The predictive value of the prognostic hub-gene signature was then further investigated in the testing set, the whole set, and the independent GSE17536 cohort. Additionally, we explored the expression of hub genes in TCGA and GTEx databases and the subcellular location in the UniProt database (UniProt, 2019).

### Identification of Key Signaling Pathways by Pathway Enrichment Analysis

To understand the molecular mechanism of hub genes involved in CRC, we performed a pathway enrichment analysis. The KEGG over-representation test was performed by KOBAS 3.0 (Xie et al., 2011) based on the genes related to CRC with the cut-off of *p* < e^−8^. To increase the reliability and credibility of the results, we employed the Gene Set Enrichment Analysis (GSEA) (Subramanian et al., 2005), which targeted the whole gene's expression to further explore the potential molecular mechanisms based on the threshold with FDR < 0.05 and *p* < 0.05. Finally, we identified the key pathways of hub genes involved in CRC using the two methods.

### Protein-Protein Interaction and Shortest-Path Analysis

For the purpose of finding possible interactions among the hub genes and the core genes involved in the signaling pathways of interest, we first built the PPI based on the genes related to CRC from the String database (Szklarczyk et al., 2017). Second, according to a previous study (Yang et al., 2018), the shortest path was considered to be the minimum number of edges required to travel from one node in the PPI network. We then performed a shortest-pathway analysis using the Python package NetworkX (http://networkx.github.io) to find the shortest path among the hub genes and core genes involved in the key pathways.

### Constructing the Transcription Factors and Hub Genes Network

In order to explore regulatory links between TFs and hub genes, we obtained all the TFs related to cancer from Cistrome Cancer (Mei et al., 2017). Differentially expressed TFs were then calculated using the R package limma (Ritchie et al., 2015) based on the TCGA and GTEx databases. The threshold for screening the differentially expressed TFs was set at |logFC| > 1 and *p* < 0.05. Subsequently, we calculated the correlation between differentially expressed TFs and hub genes with the cut-off of the |pearson correlation value| > 0.35 and *p* < 0.05. We visualized the results using Cytoscape 3.7.1 (Shannon et al., 2003).

### Potential Drug-Repurposing

According to the previous study (Gordon et al., 2020), we used two approaches to identify the drugs that modulate the hub genes in the present study. (1) We downloaded the drug targets from the Binding DB (Gilson et al., 2016). We then constructed the PPI network among drug targets and hub genes based on the String database, with a cut-off of the interaction score > 0.7. We ranked the drug targets based on the interaction score and the results were visualized by Cytoscape 3.7.1. (2) We performed structure-based virtual screening and molecular docking to find the potential drug of hub genes. We obtained the 3D structure of hub genes from the PDB or SWISS-MODEL databases and predicted the active sites of hub genes using Schrodinger maestro. A library with 2,106 Food and Drug Administration (FDA) approved drugs was then built on the ZINC15 database (Irwin et al., 2012). Afterwards, we screened the potential drug repurposing of hub genes using the docking scores.

### Statistical Analysis

All statistical analyses were conducted using R 3.5.3 (https://www.r-project.org/). The Cox proportion hazard regression method was used to identify genes that are significantly related to overall survival of CRC with *p* < 0.05. The Lasso penalized cox analysis was used to construct the gene signature. The Kaplan–Meier survival curve analysis and a log-rank test were used to compare differences in overall survival time between the high-risk group and low-risk group. The calculations of *p*-values were two-sided, and *p* < 0.05 was defined as statistically significant. The time-dependent ROC curve was calculated to compare the sensitivity and specificity of survival prediction based on the nearest neighbor method. Additionally, the DEGs were screened using the limma package with empirical Bayes methods. The co-expression analysis, using the Spearman correlation method, was applied to exam the TFs-mRNA's regulatory relationship with a cut-off of |person correlation value| > 0.35 and *p* < 0.05.

## Results

### Identification of CRC Related Genes by WGCNA

**Figure 2:**
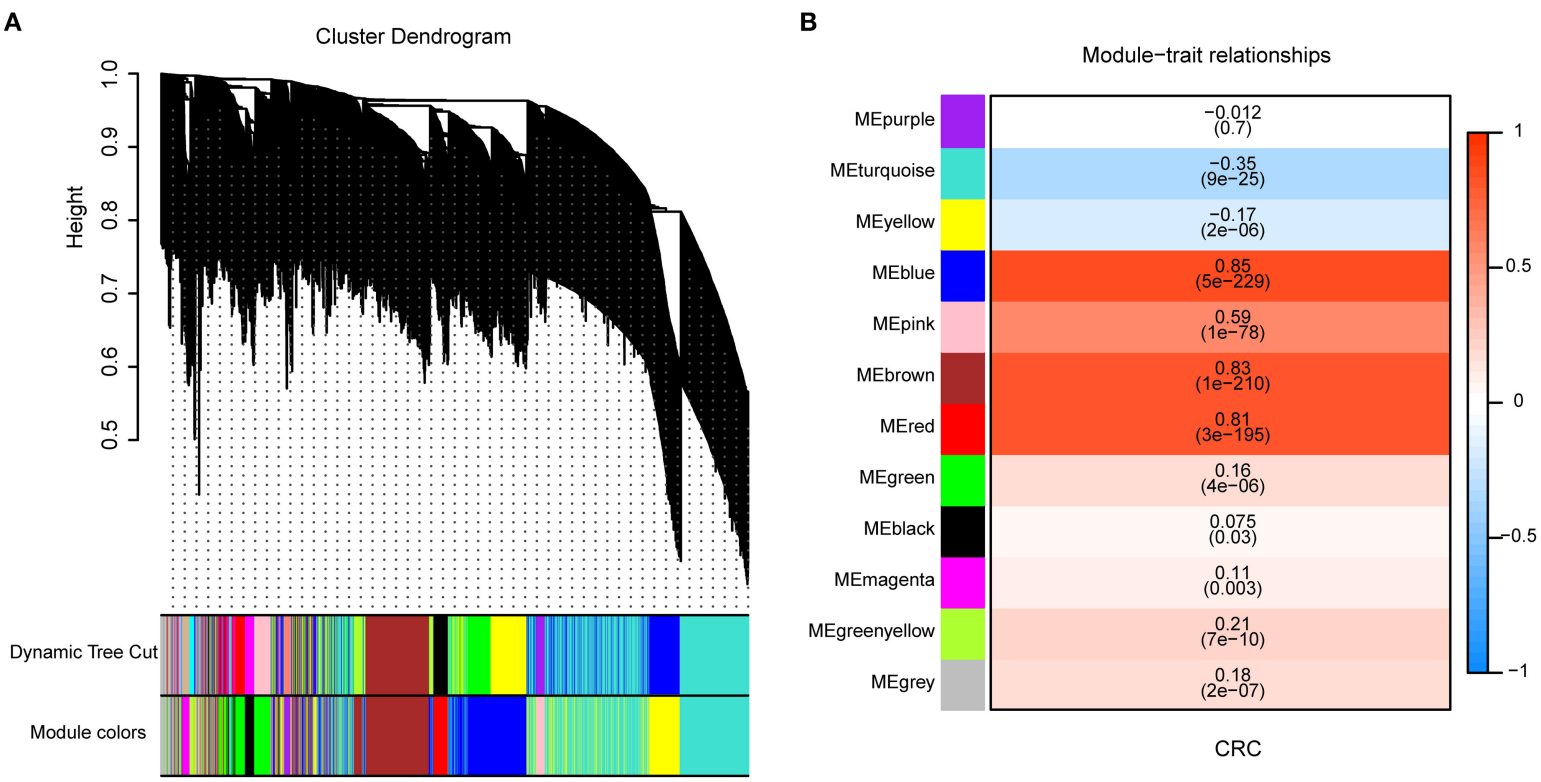
Identification of genes associated with Colorectal cancer (CRC) by Weighted Correlation Network Analysis (WGCNA). **(A)** Clustering dendrogram of 6,442 genes and 12 merged modules from 820 samples. **(B)** Correlation between modules and CRC scores. The upper row in each cell indicates the correlation coefficient ranging from – 1 to 1 of the correlation between a certain module and CRC. The lower row in each cell indicates the *p*-value.

To identify genes associated with CRC, we collected 820 samples (471 tumor and 349 normal tissues) from the TCGA and GTEx databases to do WGCNA. When the soft thresholding was set at seven and the scale-free topology fit index reached 0.85, 12 modules were identified through a one-step network construction method and are shown in different colors (Figure 2A). Based on the cutoff of the highest absolute correlation values and *p* < 0.05, the blue module was screened as being significantly related to CRC (Figure 2B, Supplementary Table 1). Finally, a total of 801 genes in the blue module (Supplementary Table 2) were identified as being significantly related to CRC based on the thresholds of the correlation of module-membership value > 0.5 and CRC-correlation value > 0.5.

### Establishing and Validating the Five-Gene Based Prognostic Signature

**Figure 3:**
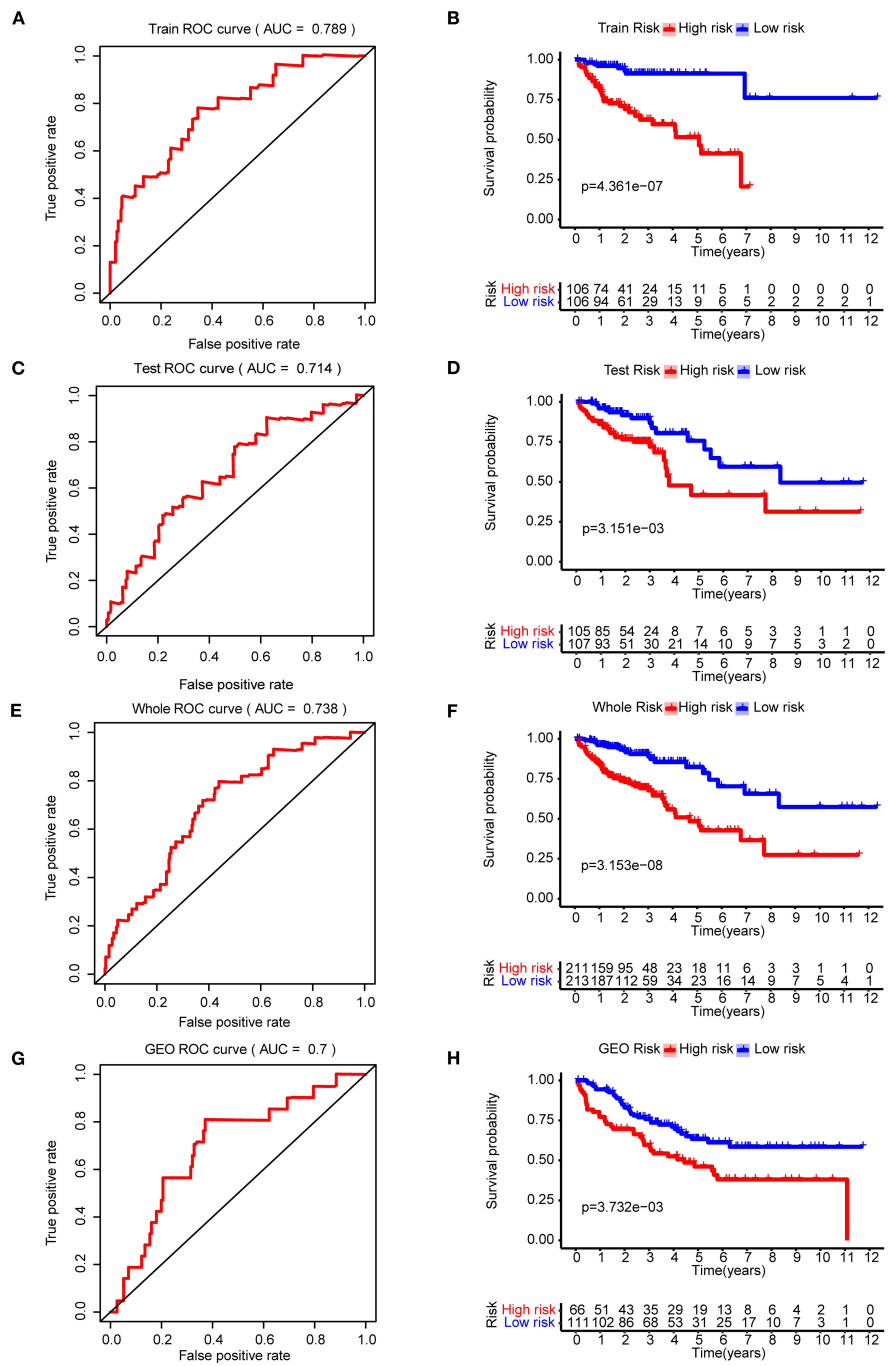
The construction and validation of the five-gene prognostic signature in CRC. Time-dependent ROC analysis and Kaplan–Meier curve of five-gene signature in training set **(A,B)**, testing dataset **(C,D)**, the whole dataset **(E,F)**, and the GSE17536 cohort **(G,H)**. ROC, receiver operating characteristic.

Four-hundred-and-twenty-four patients (Supplementary Table 3) with a survival time >1 month and 801 CRC related genes were included to construct the prognostic model. The whole data set was randomly separated into a training set (*n* = 212) and a testing set (*n* = 212) using the R package caret. The baseline characteristics are summarized in Supplementary Tables 4, 5, respectively. The clinical parameters were not significantly different from the training set and testing set. We found that 26 genes were significantly related to the overall survival using the Univariate Cox regression model (*p* < 0.05). The five-gene prognostic signature was then built using the Lasso-penalized Cox analysis. The prognostic signature included five hub genes: PGM2, PODXL, RHNO1, SCD, and SEPHS1 (Supplementary Table 6). The risk score = 0.032757 × the expression value of PODXL + 0.020453 × the expression value of RHNO1 + 0.002886 × the expression value of SCD + 0.05689 × the expression value of SEPHS1 – 0.14089 × the expression value of PGM2. Depending on the medium risk score in the training set, the patients were separated into a high- and low-risk groups. In order to assess the prognostic capacity of the five-gene signature, time-dependent ROC and Kaplan-Meier curve were performed. Similar analytic methods were employed in the testing set, the whole set, and the GSE17536 cohort to assess the performance of the five-gene prognostic model. The area under the ROC curve (AUC) for overall survival was 0.789, 0.714, 0.738, and 0.7 for the training set (Figure 3A), testing set (Figure 3C), the whole set (Figure 3E), and the GSE17536 cohort (Figure 3G), respectively. Moreover, the high-risk group was significantly associated with a poorer overall survival compared to the low-risk group in the training set, test set, the whole set, and the GSE17536 cohort (all *p* < 0.05, Figures 3B,D,F,H). Furthermore, we found that all five hub genes were significantly higher expressed in the CRC group than in the normal group, using the DEGs analysis (*p* < 0.05, Supplementary Table 7).

### Potential Transcription Regulatory Mechanism of Five Hub Genes

**Figure 4:**
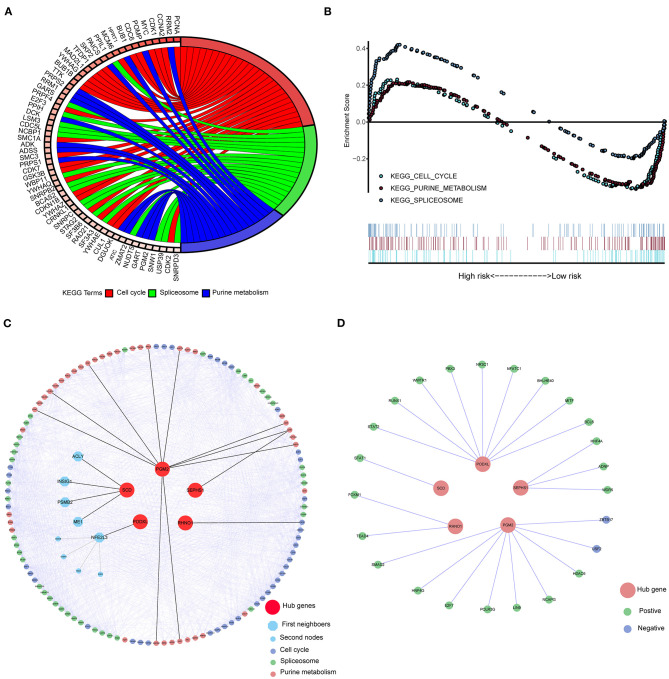
The signaling pathways of five hub genes implicated in CRC were evaluated by the KEGG pathway enrichment analysis. **(A)** The enriched pathways of CRC related genes in KOBAS 3.0 (*p* < e^−8^) with the KEGG over-representation test. **(B)** The shared pathways of GSEA between high- and low-risk groups according to the threshold of FDR < 25% and *p* < 0.05. **(C)** The molecular connections among five hub genes and key pathways through PPI and shortest path analysis. **(D)** Five Hub genes interacted with differently expressed TFs. KEGG, Kyto Encyclopedia of Genes, and Genomes; TFs, Transcription factors; PPI, Protein-protein interaction; GSEA, Gene Set Enrichment Analysis.

Eight-hundred-and-one CRC related genes were significantly enriched in three pathways (Figure 4A) using the KEGG over-representation test in the KOBAS 3.0 database: “Cell cycle,” “Spliceosome,” and “Purine metabolism” KEGG pathways. Simultaneously, according to the results of GESA with the cut-off of FDR < 25% and *p* < 0.05, 12 and 64 pathways were enriched in the CRC group and the normal group (Supplementary Table 8), respectively. Three shared pathways including “Cell Cycle,” “Purine metabolism,” and “Spliceosome” KEGG pathways (Figure 4B) were identified by the two pathway enrichment methods. We constructed the PPI network to further explore the connections among five hub genes and core genes involved in the three key KEGG pathways. There were 881 nodes and 14,520 edges in the PPI network. We then performed the shortest-path analysis based on the PPI network. As showed in Figure 4C, PGM2, SEPHS1, and RHNO1 could directly interact with the core genes involved in the three-key pathways, SCD1 and PODXL should, through other genes, crosstalk with the three-key pathways. Moreover, to explore regulatory links between transcription factors (TF) and five hub genes, we downloaded 318 TFs related to cancer (Supplementary Table 9). We then screened 119 differentially expressed TFs in CRC (|logFC| > 1 and *p* < 0.05, Supplementary Table 10). Subsequently, we employed the co-expression analysis to identify the expressed relationships between TFs and five hub genes based on the cutoff of the absolute value of correlation > 0.35 and *p* < 0.05 (Supplementary Table 11). As shown in Figure 4D, TFs might regulate the expression of hub genes (Green indicated positively regulate, Blue suggested negatively regulate).

### Potential Drug-Repurposing Based on the Hub Genes for CRC

**Figure 5:**
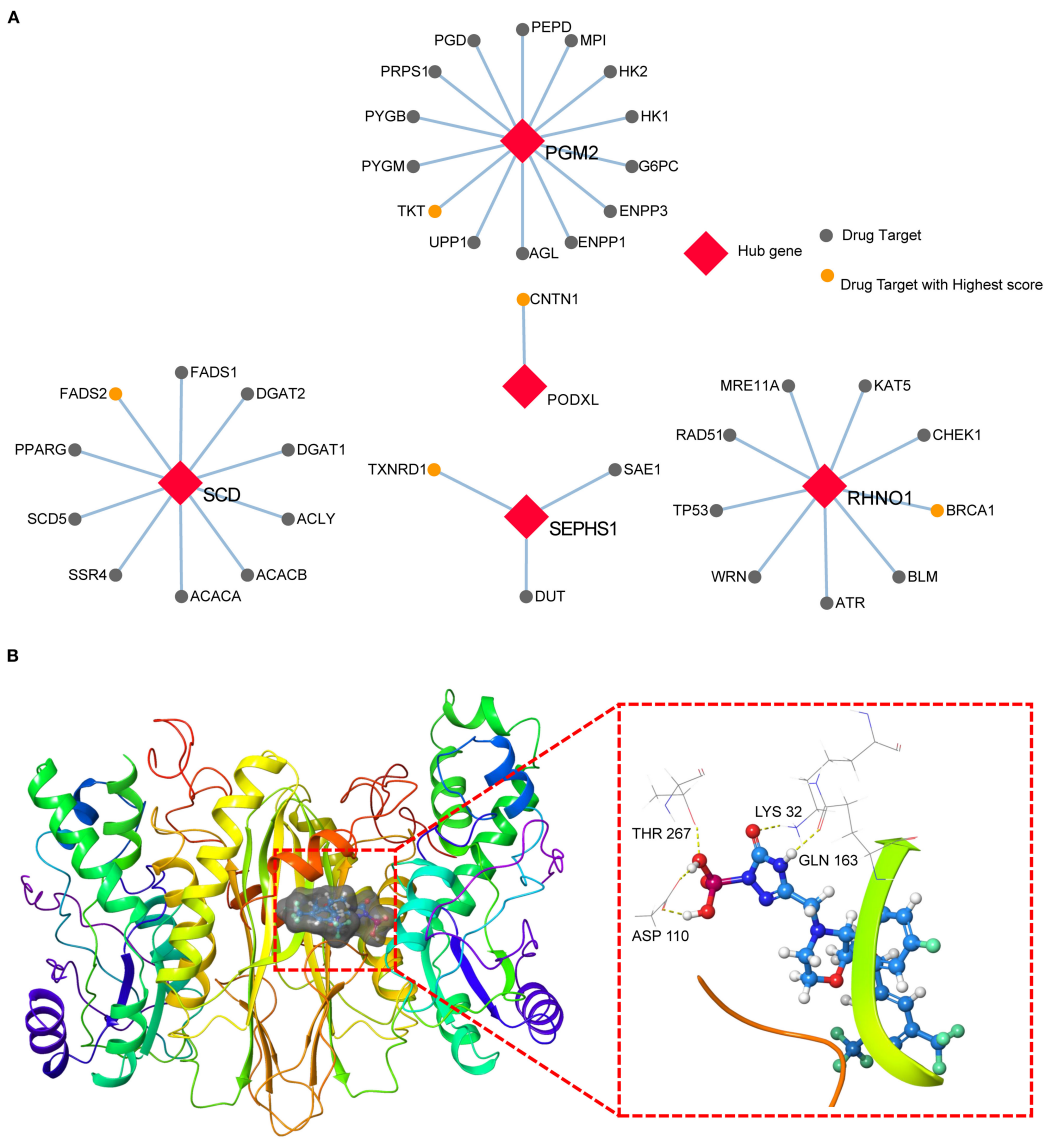
Potential drug-repurposing based on the hub genes for CRC. **(A)** The PPI network of five hub genes and targets of drugs from the Binding DB database. **(B)** The ZINC000003939013-SEPHS1 docking complex.

To explore the possibilities of potential drug-repurposing based on the hub genes for CRC, we constructed the PPI using the hub genes and targets of drugs in the Binding DB database. The cutoff of PPI was set at a score > 0.7. The most probable targets of drugs for PGM2, PODXL, RHNO1, SCD, and SEPHS1 were TKT, CNTN1, BRCA1, FADS2, and TXNRD1, respectively (Figure 5A, Supplementary Table 12). Moreover, we also screened the potential agent-repositioning depending on the 2,106 approved drugs by the FDA using structure-based virtual screening. According to the docking score, the potential drugs for PGM2 (Supplementary Table 13), PODXL (Supplementary Table 14), RHNO1 (Supplementary Table 15), SCD (Supplementary Table 16), and SEPHS1 (Supplementary Table 17) were ZINC0000000020240, ZINC000003927870, ZINC000001530948, ZINC000003830947, and ZINC000003939013, respectively. Furthermore, according to the subcellular location in the UniProt database, only SEPHS1 was in cell membrane, which was more likely to be a drug target. We then performed molecular docking between SEPHS1 and ZINC000003939013 and the ligand-protein complex had five H-bonds (Figure 5B).

## Discussion

CRC is believed to bring great challenges to, and have important impacts on, current public health. There is, therefore, currently an urgent need to identify more accurate prognostic models for CRC patients. However, previous studies have mostly focused on a single biomarker (Liu et al., 2018; Dong et al., 2019; Jary et al., 2020) or the results have been generated from small sample sizes (Huang et al., 2020; Yang et al., 2020) or have been based on DEGs (Huang et al., 2020; Yang et al., 2020), which might lower the performance of the prognostic biomarkers. In the present study, a five-gene signature was identified and validated based on the TCGA, GTEx, and GSE17536 cohort with a large sample seize (Figure 1). Furthermore, we explored potential drug repurposing, targeting five hub genes using two methods, which might expand possible therapeutic strategies and improve the prognosis of CRC patients. Finally, we explored the potential mechanism of five hub genes involved in CRC using pathway enrichment, PPI, and the shortest pathway and co-expression analysis.

In the present study, we found five hub genes (PGM2, PODXL, RHNO1, SCD, and SEPHS1) using WGCNA and Cox regression analysis. The five-gene signature model could efficiently stratify patient's overall survival and its efficacy was validated in the TCGA and the GSE17536 cohort, indicating a robust high prognostic value of the signature, especially when compared with the previous single biomarker (Dong et al., 2019; Jary et al., 2020). Moreover, other multigene-based models were mostly based on the differently expressed genes (DEGs) (Huang et al., 2020; Yang et al., 2020). It is well-known that DEGs have been screened using an artificially set threshold, which would exclude some important prognostic genes before constructing the prognostic model and which would eventually lower the performance of the model. In the present study, the prognostic signatures in CRC were screened by WGCNA, an unsupervised analysis method. Altogether, we established a robust five-gene prognostic model to guide the outcome of CRC.

We found that the five hub genes were all upregulated in CRC patients (*p* < 0.05). Moreover, the high expression of PODXL, RHNO1, SCD, and SEPHS1 were related to a poor prognosis in CRC (*p* < 0.05). The downregulated PGM2 was associated with the poor outcome in CRC, which was line with the formula of risk score. Several previous studies have revealed that PODXL, RHNO1, and SCD play an important role in CRC cancer. For example, overexpression of PODXL was reported to increase CRCs aggressive and metastatic capabilities and is associated with poor survival (Larsson et al., 2013; Kaprio et al., 2014). Moreover, upregulated PODXL might be crucial in the initiation of colorectal carcinogenesis by the disruption of the multigene network system regulating cell adhesion and the cytoskeleton (Naishiro et al., 2005). High expression of SCD was required for tumor development in mice by regulating synthesis of oleate in the enterocytes and by maintaining fatty acid homeostasis (Ducheix et al., 2018). Upregulated SCD could cause the epithelial-mesenchymal transition program in CRC cells (Sanchez-Martinez et al., 2015). Moreover, inhibition of the expression of SCD could induce cell death in CRC stem cells (Potze et al., 2016). High expression of SEPHS1 is related to the poor outcome of CRC (Choi et al., 2011). Although PGM2 and RHNO1 have not been reported in CRC, they are involved in other tumor developments (Marshall et al., 1979; Kim et al., 2010). Altogether, our findings are consistent with the results of previous studies showing that the five hub genes are functionally important for the prognosis of CRC.

It is a well-established fact that the development of a new drug is a long, complex, and costly process. Therefore, drug-repurposing has attracted a lot of attention, and reduces the need for additional toxicological experiments (Sleire et al., 2017). Recent studies have shown that Thalidomide and Raloxifene have already been approved for treatment of myeloma (Luo et al., 2017) and breast cancer (Pinsky et al., 2018), respectively. So, we wanted to take a step from the analysis of establishing gene prognostic models in CRC. We then used two methods to screen the potential drug-repurposing of five hub genes in CRC. In one method, we constructed the PPI using hub genes and targets of drugs in the Binding DB database—in an indirect manner. In the other method we directly found the potential drug repurposing using structure-based virtual screening and molecular docking. We hope to provide a faster and cheaper strategy for expanding the arsenal of approved cancer drugs.

To provide insight into the molecular mechanism of the five hub genes involved in CRC, we first performed the KEGG pathway enrichment using KOBAS 3.0 and GSEA. Three shared pathways were identified by the pathway enrichment analysis: “Cell cycle,” “Purine metabolism,” and “Spliceosome” KEGG pathways. Second, we further obtained the interactions among hub genes and three-key pathways (Figure 4) through PPI and shortest pathway analysis. Depending on the KEGG database, “Cell cycle,” “Purine metabolism,” and “Spliceosome” KEGG pathways were related to Cell growth and death, and Metabolism and Transcription, respectively. We then speculated that the three pathways might be involved in the regulation of cell growth, cell cycle progression, and metastasis in CRC. Moreover, recent studies have demonstrated that these pathways play an important role in CRC (Buolamwini, 2000; Pedley and Benkovic, 2017; Mabonga and Kappo, 2019). Altogether, we indicated that five hub genes might be involved in CRC through three-key pathways. Accumulating evidence suggests that Purine metabolism and Spliceosome are fundamental and necessary for tumor cell proliferation (Di Virgilio, 2012; Camici et al., 2019), which have been suggested to be associated with Cell cycle. In line with previous studies, we suggest that the three pathways might crosstalk with each other through five hub genes in CRC. However, large numbers of verification experiments are still needed in the future.

Simultaneously, we explored the upstream mechanism of five hub genes in CRC. Several TFs were co-expression with five hub genes. Based on previous studies (Muller and Rao, 2010; Mei et al., 2017; Lambert et al., 2018), TFs have been suggested to be involved in various physiological pathways including “Cell cycle,” “Purine metabolism,” and “Spliceosome” KEGG pathways in CRC. Altogether, the results might partly explain the upstream mechanism of five hub genes in CRC. However, further well-designed experiments are required to prove this hypothesis.

## Conclusion

In summary, we established that a signature of five genes corrected with progression and prognosis in CRC, depends on the gene expression profile datasets (TCGA, GTEx, and GEO databases) and bioinformatics analysis. Moreover, we confirmed the molecular details of connections among hub genes (PGM2, PODXL, RHNO1, SCD, and SEPHS1) and three key pathways (“Cell cycle,” “Purine metabolism,” and “Spliceosome”) and a co-expression relationship between hub genes and TFs to obtain a better understanding of molecular mechanisms involved in CRC. Furthermore, we screened the potential agent-repurposing based on the hub genes for CRC according to the Binding DB and ZINC15 databases. Altogether, we constructed a five-gene signature to significantly distinguish overall survival of CRC and found the potential drug-repurposing, which may improve the outcome of CRC in the future.

## Data Availability Statement

The datasets presented in this study can be found in online repositories. The names of the repository/repositories and accession number(s) can be found in the article/Supplementary Material.

## Author Contributions

QW and FY conceived the project, designed the study, and drafted the manuscript. LT directed the study. SC and LL collected the public data. HZ revised the manuscript. All authors read and approved the final manuscript.

## Conflict of Interest

The authors declare that the research was conducted in the absence of any commercial or financial relationships that could be construed as a potential conflict of interest.
