# Supplementary material for: Identification of a Five-Gene Prognostic Model and Its Potential Drug Repurposing in Colorectal Cancer Based on TCGA, GTEx and GEO Databases
Source: Front Genet. 2021 Jan 18;11:622659. doi: 10.3389/fgene.2020.622659 (PMC7848190; doi:10.3389/fgene.2020.622659)
Supplement: Supplementary file 1 [file Table_1.DOCX]

**SUPLEMENTAL DATA**

**Identification of a five-gene prognostic model and its potential molecular mechanism in Colorectal cancer based on TCGA, GTEx and GEO Databases**

Feng Yang^1^, Shaoyi Cai^1^, Li Ling^1^, Haiji Zhang^1^, Liang Tao^1*^, Qin Wang^1*^

*1. Zhongshan School of Medicine, Sun Yat-Sen University, Guangzhou, 510080, China.*

**Postal address:** NO. 74, Zhongshan Second Road, Guangzhou 510080, China

*Correspondence author:

Liang Tao, PhD, Zhongshan School of Medicine, Sun Yat-Sen University, Guangzhou, 510080,

China. E-mail: taol@mail.sysu.edu.cn.

Qin Wang, PhD, Zhongshan School of Medicine, Sun Yat-Sen University, Guangzhou, 510080,

China. E-mail: wangqin6@mail.sysu.edu.cn.

**Supplementary tables:**

**Table S1**. The correlations between module eigengenes and clinical traits.

| Module | Trait | PersonCorrelationValue | Pvalue |
| --- | --- | --- | --- |
| MEblack | CRC | 0.075427 | 0.030798 |
| MEblue | CRC | 0.849156 | 5.32E-229 |
| MEbrown | CRC | 0.831055 | 1.31E-210 |
| MEgreen | CRC | 0.161176 | 3.51E-06 |
| MEgreenyellow | CRC | 0.213112 | 7.08E-10 |
| MEgrey | CRC | 0.181703 | 1.62E-07 |
| MEmagenta | CRC | 0.105072 | 0.002591 |
| MEpink | CRC | 0.592019 | 1.05E-78 |
| MEpurple | CRC | -0.01183 | 0.735278 |
| MEred | CRC | 0.81417 | 2.52E-195 |
| MEturquoise | CRC | -0.34803 | 9.24E-25 |
| MEyellow | CRC | -0.16616 | 1.72E-06 |

**Table S2**. Gene significantly related to Colorectal cancer.

| Symbol | geneModuleMembership | geneTraitCor |
| --- | --- | --- |
| ARL8B | 0.718860195 | 0.527331064 |
| DESI2 | 0.864542575 | 0.775965436 |
| NCAPG | 0.839058368 | 0.791950136 |
| CENPH | 0.814421226 | 0.799728963 |
| ATP6AP2 | 0.798423701 | 0.697748866 |
| INTS13 | 0.833263707 | 0.770949888 |
| NOX1 | 0.574382737 | 0.589662825 |
| RNF5 | 0.619293954 | 0.544379258 |
| MPLKIP | 0.779391404 | 0.78720386 |
| RMI2 | 0.710176798 | 0.762722412 |
| POGK | 0.819350266 | 0.783112087 |
| RPF1 | 0.809562677 | 0.672169903 |
| EIF3I | 0.756295417 | 0.807188929 |
| NIF3L1 | 0.804838039 | 0.80690598 |
| MZT1 | 0.871272961 | 0.788250361 |
| DDX21 | 0.896772937 | 0.795591158 |
| MORF4L2 | 0.769305196 | 0.652142478 |
| FBXO28 | 0.851376951 | 0.71653029 |
| PDIA3 | 0.699600291 | 0.648839036 |
| SLPI | 0.506985794 | 0.57105342 |
| RBM12 | 0.889706508 | 0.797965836 |
| ZNF664 | 0.798161738 | 0.672576217 |
| CXCL8 | 0.562482053 | 0.664391064 |
| UBE2J1 | 0.799091559 | 0.661983714 |
| INSIG1 | 0.647407132 | 0.657274939 |
| EIF2AK2 | 0.838726023 | 0.753139931 |
| ZDHHC20 | 0.858129705 | 0.771850827 |
| ABCC1 | 0.581056349 | 0.6928759 |
| STAT1 | 0.666322185 | 0.648468262 |
| NEK2 | 0.857758692 | 0.831667353 |
| FZD6 | 0.809588528 | 0.74987711 |
| PBK | 0.767399516 | 0.731028007 |
| MAIP1 | 0.797989499 | 0.713355698 |
| ZBTB33 | 0.864584006 | 0.801018831 |
| MOB1A | 0.851591599 | 0.689482348 |
| SLBP | 0.867038879 | 0.815930252 |
| GNL3 | 0.741683107 | 0.690207651 |
| GANAB | 0.68015995 | 0.741367906 |
| MRFAP1L1 | 0.615693934 | 0.527020767 |
| S100A6 | 0.625546187 | 0.697434625 |
| MRPS16 | 0.776526063 | 0.723461891 |
| PPIH | 0.731746989 | 0.7605135 |
| DLGAP5 | 0.842097037 | 0.793676547 |
| MTIF2 | 0.82901353 | 0.705546988 |
| CUL1 | 0.759615519 | 0.727061223 |
| RACGAP1 | 0.847473973 | 0.824664502 |
| TTK | 0.885191139 | 0.799291533 |
| USP14 | 0.707306744 | 0.612250367 |
| GARS | 0.769015188 | 0.785567285 |
| MACC1 | 0.78701059 | 0.797442918 |
| ASF1A | 0.862764955 | 0.71332992 |
| PBDC1 | 0.743354364 | 0.72073768 |
| SNX7 | 0.744410296 | 0.603397702 |
| TNPO1 | 0.830109949 | 0.691918546 |
| ALG5 | 0.72483336 | 0.720151285 |
| APP | 0.620025305 | 0.622468797 |
| SMARCA5 | 0.786348509 | 0.593050531 |
| UBTD2 | 0.819904695 | 0.676640772 |
| UGGT1 | 0.844243565 | 0.802256204 |
| HSPA5 | 0.72591753 | 0.728143104 |
| POLR2K | 0.687103894 | 0.522579732 |
| DPY19L1 | 0.817850317 | 0.784985751 |
| URI1 | 0.670611219 | 0.55873697 |
| P4HA1 | 0.715882147 | 0.702721372 |
| CNOT6 | 0.864508069 | 0.763602621 |
| TMEM14A | 0.794955966 | 0.733351081 |
| SEPHS1 | 0.749213818 | 0.785279526 |
| NMD3 | 0.840093886 | 0.724090965 |
| RNF6 | 0.796983168 | 0.72575725 |
| DKC1 | 0.807495363 | 0.824668204 |
| STK39 | 0.805547989 | 0.717306246 |
| TOP2A | 0.876571289 | 0.801746888 |
| TMEM181 | 0.790177706 | 0.680873082 |
| CNOT9 | 0.837705244 | 0.837388572 |
| HNRNPAB | 0.736058167 | 0.755692839 |
| ARL1 | 0.731830455 | 0.587306751 |
| ALDH1B1 | 0.578905594 | 0.614099169 |
| JPT2 | 0.865032473 | 0.853508406 |
| LYAR | 0.802767713 | 0.779674283 |
| SMIM15 | 0.879690172 | 0.699176768 |
| CENPA | 0.808144454 | 0.812304113 |
| DAD1 | 0.729400009 | 0.713745991 |
| MYO1C | 0.666062459 | 0.651959894 |
| TFAM | 0.879763683 | 0.701127172 |
| NUDT5 | 0.675031706 | 0.644274702 |
| INTS7 | 0.850511619 | 0.850887056 |
| CPD | 0.799040266 | 0.731267776 |
| CDV3 | 0.751558893 | 0.62702934 |
| YWHAE | 0.611710244 | 0.575719066 |
| SDE2 | 0.874710821 | 0.752139115 |
| PSPC1 | 0.610110182 | 0.559741392 |
| CDC6 | 0.808786772 | 0.808183153 |
| PSME3 | 0.773986192 | 0.743235437 |
| CAPRIN1 | 0.868345926 | 0.772569267 |
| RFC5 | 0.807854536 | 0.785824945 |
| CENPF | 0.683260111 | 0.649507367 |
| PPP4R3B | 0.806922465 | 0.596379441 |
| SRPRB | 0.811516514 | 0.825730942 |
| PPP3R1 | 0.904656584 | 0.750833756 |
| COA7 | 0.855054799 | 0.804015621 |
| ZMAT2 | 0.70089902 | 0.599724645 |
| PSMD1 | 0.631999926 | 0.574955797 |
| MRPS35 | 0.877387166 | 0.79245347 |
| MRM2 | 0.765030415 | 0.807714975 |
| ETF1 | 0.788358185 | 0.624936143 |
| WDR43 | 0.935703808 | 0.847996755 |
| EZH2 | 0.794165774 | 0.809314229 |
| TPX2 | 0.81959004 | 0.824318988 |
| MCMBP | 0.795215013 | 0.686419509 |
| CNOT11 | 0.872252451 | 0.810467708 |
| PNPT1 | 0.866213719 | 0.785005901 |
| MYC | 0.64340864 | 0.706194731 |
| TOMM20 | 0.857051058 | 0.800298243 |
| KIF2A | 0.828615186 | 0.732979287 |
| RRP1B | 0.838878786 | 0.835844355 |
| UNG | 0.808526504 | 0.787072356 |
| BAG5 | 0.839060768 | 0.755337743 |
| UBD | 0.516930278 | 0.586712151 |
| APOO | 0.74921533 | 0.693229874 |
| CSNK2A1 | 0.750775758 | 0.689465363 |
| VAMP3 | 0.703093525 | 0.579113594 |
| GNPNAT1 | 0.849635309 | 0.769455152 |
| LRR1 | 0.708458289 | 0.583994854 |
| GOLT1B | 0.801418484 | 0.67654919 |
| TMEM33 | 0.871011751 | 0.789121676 |
| DCUN1D5 | 0.818519136 | 0.806214294 |
| ACLY | 0.782500563 | 0.823605659 |
| HSP90AB1 | 0.830340256 | 0.836016635 |
| TMEM185B | 0.770190943 | 0.848445252 |
| CANX | 0.822327752 | 0.721638113 |
| LAPTM4A | 0.711440691 | 0.602347587 |
| DDX50 | 0.750133191 | 0.659780099 |
| CPOX | 0.80511847 | 0.814173717 |
| IARS2 | 0.844393721 | 0.754456826 |
| LAMP2 | 0.712508682 | 0.625557391 |
| RP2 | 0.814188729 | 0.695301399 |
| ACTL6A | 0.782416533 | 0.74245725 |
| PLAA | 0.851357454 | 0.773045741 |
| PPM1H | 0.771596114 | 0.825222084 |
| TSN | 0.933117648 | 0.799716372 |
| SMC1A | 0.729592129 | 0.705338399 |
| CBFB | 0.882419852 | 0.831714187 |
| TSPAN12 | 0.578904722 | 0.501019335 |
| RAD51AP1 | 0.871412259 | 0.783991572 |
| PTPN11 | 0.704005512 | 0.589675983 |
| FAM91A1 | 0.79770547 | 0.697571044 |
| USP39 | 0.654910642 | 0.67869227 |
| STMP1 | 0.771056995 | 0.668787415 |
| CCT8 | 0.816264557 | 0.749308204 |
| SUDS3 | 0.821352437 | 0.700476611 |
| RCC2 | 0.79678879 | 0.862387316 |
| GAR1 | 0.729397865 | 0.766966159 |
| PRPF4 | 0.807147822 | 0.855130593 |
| YRDC | 0.79867913 | 0.815807238 |
| XRCC6 | 0.711744146 | 0.642776311 |
| TMEM14C | 0.674734739 | 0.648712662 |
| PAK1IP1 | 0.871624659 | 0.805673379 |
| FAM136A | 0.818655712 | 0.804668419 |
| GUF1 | 0.845425696 | 0.737988459 |
| FAM84B | 0.796148335 | 0.793100907 |
| LTV1 | 0.762191737 | 0.655482092 |
| ERH | 0.782263494 | 0.756990358 |
| STAG2 | 0.770227656 | 0.631588987 |
| RAB7A | 0.813101797 | 0.734900491 |
| DARS | 0.793358294 | 0.6826482 |
| ARHGAP18 | 0.839988842 | 0.736999179 |
| MRPS10 | 0.910300697 | 0.789022405 |
| UBE3C | 0.719306823 | 0.669157165 |
| CASP3 | 0.802972593 | 0.67553679 |
| TESC | 0.501822613 | 0.652559608 |
| MTPN | 0.856935697 | 0.745148487 |
| PUS7 | 0.845382623 | 0.816885675 |
| IMPAD1 | 0.795579413 | 0.675485403 |
| SMC3 | 0.841504369 | 0.703336673 |
| RIPK2 | 0.769128623 | 0.803439875 |
| RPS12 | 0.735310001 | 0.771557677 |
| NBDY | 0.633724272 | 0.677315043 |
| UTP14A | 0.759058759 | 0.769947885 |
| HS2ST1 | 0.869505252 | 0.778621831 |
| FTL | 0.669847716 | 0.684133448 |
| LRRC42 | 0.684560959 | 0.720926865 |
| YTHDF3 | 0.793599717 | 0.618355101 |
| DFFA | 0.743897104 | 0.785783829 |
| ALDH9A1 | 0.678383553 | 0.551751842 |
| AC005288.1 | 0.859692808 | 0.75893663 |
| FOPNL | 0.892818281 | 0.773022648 |
| KLHDC10 | 0.817642349 | 0.757524155 |
| COA6 | 0.691579931 | 0.64134162 |
| CDC5L | 0.914675934 | 0.787707505 |
| MGME1 | 0.770947646 | 0.778547445 |
| GATC | 0.849402575 | 0.804316517 |
| RFC3 | 0.854454523 | 0.815051103 |
| SMARCC1 | 0.845158714 | 0.821104349 |
| ANP32B | 0.723826423 | 0.727194114 |
| NOL7 | 0.780903642 | 0.707979352 |
| PPIL1 | 0.865260476 | 0.842096698 |
| BFAR | 0.698079675 | 0.681100769 |
| XRCC5 | 0.900277937 | 0.786619327 |
| POF1B | 0.79389558 | 0.718332537 |
| SNX4 | 0.817810843 | 0.629848749 |
| UBQLN1 | 0.800030583 | 0.659768558 |
| VPS35 | 0.806568548 | 0.631983557 |
| FARSB | 0.881708439 | 0.833499782 |
| EIF2AK1 | 0.805591484 | 0.81569141 |
| KARS | 0.779120258 | 0.780122636 |
| TGOLN2 | 0.666232942 | 0.575820436 |
| CEBPZ | 0.731672434 | 0.530972045 |
| MRPS9 | 0.711260816 | 0.602755207 |
| FAM111B | 0.82064445 | 0.780970946 |
| MAGT1 | 0.859626128 | 0.76231107 |
| COQ10B | 0.69402999 | 0.518240678 |
| HNRNPH2 | 0.801829895 | 0.715550722 |
| GNPAT | 0.671631438 | 0.607041188 |
| NDFIP1 | 0.722467941 | 0.574134802 |
| OCRL | 0.629710392 | 0.553176969 |
| RRM2 | 0.820816596 | 0.799985978 |
| AP1AR | 0.81243204 | 0.653625823 |
| EIF4EBP2 | 0.671683784 | 0.520301924 |
| KIAA1147 | 0.70586624 | 0.675510764 |
| DHX9 | 0.78304876 | 0.718223155 |
| PLBD1 | 0.651842049 | 0.673878241 |
| OSTC | 0.839194833 | 0.728325052 |
| RAB6A | 0.725874697 | 0.50943743 |
| AGPAT5 | 0.788418754 | 0.709891695 |
| SLC30A6 | 0.923145343 | 0.800804905 |
| KCTD20 | 0.767274436 | 0.672619118 |
| GPN1 | 0.719320706 | 0.71084152 |
| KDM7A | 0.677803189 | 0.607435174 |
| RAB10 | 0.906759008 | 0.766468321 |
| YARS2 | 0.769790262 | 0.797094541 |
| PGK1 | 0.609745491 | 0.638216361 |
| GTF2H3 | 0.910976607 | 0.80444735 |
| NDC1 | 0.906855305 | 0.813138078 |
| XPO5 | 0.740503231 | 0.776485479 |
| RARS2 | 0.781136407 | 0.679842362 |
| NCAPG2 | 0.834149417 | 0.824721306 |
| NAA50 | 0.717882912 | 0.52429314 |
| RB1 | 0.779070138 | 0.674479259 |
| PSMD10 | 0.767360401 | 0.738507686 |
| NCAPD2 | 0.768055661 | 0.803263901 |
| PMS2P1 | 0.771080774 | 0.791149402 |
| PSMD5 | 0.852866285 | 0.730875808 |
| TXLNG | 0.779579902 | 0.73754358 |
| TRIAP1 | 0.813898523 | 0.787837644 |
| MAP2K1 | 0.594236486 | 0.507052756 |
| SSR1 | 0.757770878 | 0.663870312 |
| HSPA13 | 0.826055067 | 0.694792685 |
| MCM6 | 0.832768492 | 0.834565224 |
| PPP1R14BP3 | 0.696964177 | 0.704688348 |
| ANLN | 0.863922756 | 0.810376485 |
| NOLC1 | 0.834297615 | 0.800365516 |
| PGM2 | 0.718656908 | 0.577164586 |
| ASNSD1 | 0.909953965 | 0.785680309 |
| EBNA1BP2 | 0.578657133 | 0.554656327 |
| FNDC3A | 0.664897916 | 0.525622216 |
| PRR11 | 0.839900951 | 0.823860623 |
| DDAH1 | 0.800914418 | 0.71986888 |
| SYPL1 | 0.788325822 | 0.670330095 |
| REEP5 | 0.74210273 | 0.636053924 |
| CIAO1 | 0.766189728 | 0.802054456 |
| CMTM6 | 0.8645407 | 0.763189447 |
| GSPT1 | 0.776347341 | 0.63229002 |
| ZW10 | 0.877543499 | 0.821176814 |
| CTNNB1 | 0.74418068 | 0.756996953 |
| HOXB-AS4 | 0.61065446 | 0.588235842 |
| EAF1 | 0.88886562 | 0.786941505 |
| AP3M1 | 0.830532369 | 0.64314818 |
| PTMAP4 | 0.696136318 | 0.726707624 |
| PA2G4 | 0.753714191 | 0.720796583 |
| HEATR3 | 0.808993505 | 0.766889668 |
| ATG5 | 0.843191406 | 0.680882492 |
| NCOA7 | 0.683479116 | 0.634504398 |
| PDCL3 | 0.620438082 | 0.564304324 |
| MKI67 | 0.765771755 | 0.753349739 |
| SLC25A32 | 0.845526146 | 0.753649582 |
| DRG1 | 0.751540666 | 0.764756091 |
| POLR1B | 0.854288173 | 0.862347786 |
| BROX | 0.796181752 | 0.674433467 |
| MAD2L1 | 0.821921818 | 0.757536082 |
| CCT6A | 0.802383642 | 0.754363121 |
| ARL6IP5 | 0.677214993 | 0.521153826 |
| YWHAQ | 0.824195514 | 0.684633283 |
| HNRNPF | 0.834052094 | 0.823962702 |
| PSMC2 | 0.752399086 | 0.757527557 |
| ATP6V1G1 | 0.849943853 | 0.752543012 |
| HSPA9 | 0.789046238 | 0.721302134 |
| GLO1 | 0.903025676 | 0.785096614 |
| MRPL39 | 0.782500757 | 0.693766329 |
| XPOT | 0.835525357 | 0.763878157 |
| RRP36 | 0.54868177 | 0.551957518 |
| ITGA2 | 0.806457793 | 0.805814255 |
| PDZD8 | 0.804656694 | 0.771238326 |
| STT3A | 0.598138507 | 0.584035542 |
| NDUFB6 | 0.650321809 | 0.583763848 |
| DIMT1 | 0.7968358 | 0.655261514 |
| PERP | 0.788023817 | 0.800788326 |
| SRPK1 | 0.857557576 | 0.770878058 |
| SS18L2 | 0.727600829 | 0.729076095 |
| RLIM | 0.824506817 | 0.797349061 |
| BMS1 | 0.777864017 | 0.800548557 |
| PPP1R8 | 0.821806088 | 0.75297853 |
| ZBTB2 | 0.850838361 | 0.773404415 |
| CERS6 | 0.806628635 | 0.732346242 |
| ALG8 | 0.697894379 | 0.660784991 |
| ANKRD22 | 0.745321808 | 0.736710676 |
| CKS2 | 0.802962669 | 0.809221791 |
| MMGT1 | 0.827451226 | 0.704501785 |
| MRPL9 | 0.617977329 | 0.616806378 |
| CDK2 | 0.574358288 | 0.514811295 |
| USP9X | 0.626959341 | 0.569237235 |
| ZMPSTE24 | 0.881524603 | 0.68144348 |
| CRK | 0.603783617 | 0.503077947 |
| ATP6V1A | 0.829080815 | 0.714059693 |
| AC092718.4 | 0.803610473 | 0.8148252 |
| RPL41P5 | 0.615416863 | 0.584344196 |
| CRNKL1 | 0.749672823 | 0.678562916 |
| MIR196A1 | 0.612267714 | 0.59913783 |
| NMI | 0.632619395 | 0.552555628 |
| LRRC59 | 0.766892397 | 0.788144322 |
| NRAS | 0.860265139 | 0.709569901 |
| MRPL15 | 0.779315063 | 0.757156312 |
| SELENOI | 0.894013016 | 0.813342595 |
| MTMR6 | 0.811411525 | 0.689392825 |
| TNFRSF10A | 0.674534451 | 0.763892232 |
| ERLEC1 | 0.842027389 | 0.729486077 |
| PPP2R5A | 0.7826533 | 0.615711556 |
| ELF1 | 0.739910425 | 0.626060752 |
| TSFM | 0.521561438 | 0.509818037 |
| COPZ1 | 0.802467665 | 0.800040195 |
| SNORD14A | 0.703826886 | 0.72985132 |
| CCNA2 | 0.86087912 | 0.810744618 |
| PCNA | 0.758984802 | 0.789580129 |
| KDM1A | 0.583420715 | 0.638066752 |
| AKIRIN1 | 0.735178569 | 0.550660568 |
| MTHFD2 | 0.705126076 | 0.67554508 |
| AP2B1 | 0.738496747 | 0.726303342 |
| SRP9 | 0.787190312 | 0.621453757 |
| AC099850.3 | 0.878886508 | 0.818844006 |
| EEF1B2 | 0.612848221 | 0.613272508 |
| CHCHD4 | 0.612591949 | 0.566447728 |
| AC010326.3 | 0.716586247 | 0.72859959 |
| KIF11 | 0.881680219 | 0.805039197 |
| PNPO | 0.772767558 | 0.808848561 |
| PSMD14 | 0.674077157 | 0.653892553 |
| CEACAM6 | 0.629768599 | 0.670009579 |
| HIBADH | 0.761920994 | 0.709371 |
| G3BP1 | 0.8437598 | 0.749262337 |
| ACSL4 | 0.661595504 | 0.612265922 |
| SKIL | 0.73166619 | 0.673686393 |
| PABPC1 | 0.769903952 | 0.78124656 |
| LSM3 | 0.819361417 | 0.742722778 |
| EIF2S3 | 0.787260104 | 0.765267775 |
| RPS4X | 0.757803053 | 0.794680007 |
| ABCE1 | 0.887113854 | 0.756212881 |
| GOLGA7 | 0.730769643 | 0.625570346 |
| PPT1 | 0.752261796 | 0.694726692 |
| AC016596.2 | 0.623637281 | 0.618372049 |
| MSMO1 | 0.714730796 | 0.632736393 |
| ADAR | 0.633311584 | 0.596371981 |
| MRPL30 | 0.901414913 | 0.820311229 |
| PSMB2 | 0.748905656 | 0.798877261 |
| PRDX1 | 0.709186484 | 0.684669244 |
| AC092338.1 | 0.559256992 | 0.556140896 |
| PSMD12 | 0.846623942 | 0.740479949 |
| TTC1 | 0.688525608 | 0.617735892 |
| ILF2 | 0.831287015 | 0.830903941 |
| GLCE | 0.749615739 | 0.705173575 |
| DTL | 0.858457173 | 0.816088772 |
| TIPRL | 0.872508507 | 0.700463965 |
| ATP1B1 | 0.648182095 | 0.616703954 |
| HSP90AA1 | 0.761482162 | 0.679972524 |
| KIF5B | 0.743775176 | 0.622363648 |
| XRN2 | 0.836480098 | 0.774054001 |
| MARS2 | 0.801203129 | 0.821094713 |
| CLDN2 | 0.579307396 | 0.676019246 |
| SORD | 0.686256687 | 0.762057658 |
| TMEM209 | 0.874741922 | 0.780420511 |
| MRPL19 | 0.844500197 | 0.66645219 |
| DGUOK | 0.649143573 | 0.654672645 |
| PRPS2 | 0.84866092 | 0.78008105 |
| CENPW | 0.759496561 | 0.795042281 |
| NDUFA1 | 0.598131592 | 0.577881497 |
| CYB5B | 0.545674366 | 0.504379016 |
| KPNA2 | 0.845533758 | 0.834638836 |
| API5 | 0.881669537 | 0.695968671 |
| ARL6IP1 | 0.862796709 | 0.757674559 |
| GINS1 | 0.806745612 | 0.800590962 |
| SRP72 | 0.862997989 | 0.72396252 |
| CD55 | 0.567828524 | 0.604465976 |
| YES1 | 0.779332458 | 0.624965262 |
| AC005912.1 | 0.558231865 | 0.587503229 |
| C5orf15 | 0.799143471 | 0.693758842 |
| KYAT3 | 0.738012332 | 0.623770913 |
| SPAST | 0.921294727 | 0.750178535 |
| SMC2 | 0.89573389 | 0.792682349 |
| MELK | 0.847322071 | 0.827333566 |
| NUDT19 | 0.813179047 | 0.818007785 |
| GSK3B | 0.83941249 | 0.789114641 |
| HACD2 | 0.830735493 | 0.652378438 |
| IQGAP3 | 0.691245725 | 0.810035147 |
| SNTB1 | 0.732789974 | 0.778249717 |
| NUFIP2 | 0.776138306 | 0.678050482 |
| ATIC | 0.581217577 | 0.638382977 |
| TYMS | 0.56996439 | 0.581388237 |
| CCT2 | 0.754651401 | 0.75998483 |
| VAMP7 | 0.830155803 | 0.68548657 |
| DTX3L | 0.799850679 | 0.734715728 |
| HMMR | 0.836113585 | 0.760971089 |
| TOMM22 | 0.805012497 | 0.768487724 |
| RPF2 | 0.758441251 | 0.654674798 |
| ECT2 | 0.888652201 | 0.816234908 |
| VKORC1L1 | 0.859266219 | 0.821061063 |
| TFDP1 | 0.766643623 | 0.783319749 |
| ITCH | 0.79386646 | 0.659885495 |
| MEST | 0.775963604 | 0.770915241 |
| PODXL | 0.530007869 | 0.586210118 |
| OLA1 | 0.644171788 | 0.506795416 |
| CLDN12 | 0.738082575 | 0.724816564 |
| PAICS | 0.873494337 | 0.807669632 |
| VRK1 | 0.788780045 | 0.715238642 |
| PCMT1 | 0.664042683 | 0.564102031 |
| NAA20 | 0.646713278 | 0.640060671 |
| CISD2 | 0.849749559 | 0.77949308 |
| AC026401.3 | 0.607132611 | 0.658110789 |
| ZC3H15 | 0.912881084 | 0.767623739 |
| CDKN3 | 0.763700068 | 0.757652314 |
| CENPU | 0.771863118 | 0.720284949 |
| CARNMT1 | 0.878519162 | 0.795346521 |
| GPC4 | 0.569041577 | 0.565703864 |
| POMP | 0.803152809 | 0.787383057 |
| TCF19 | 0.714887707 | 0.792499988 |
| RPE | 0.862660357 | 0.740114998 |
| SQLE | 0.726454942 | 0.710131428 |
| UBLCP1 | 0.758747631 | 0.567637748 |
| MRPL18 | 0.753844414 | 0.717345948 |
| GDI2 | 0.884567031 | 0.766412251 |
| PAFAH1B2 | 0.86601839 | 0.772190215 |
| TGDS | 0.824073221 | 0.791256543 |
| NBN | 0.713656142 | 0.54346527 |
| HSPA4 | 0.805555393 | 0.703824963 |
| AGMAT | 0.748764237 | 0.767217577 |
| IWS1 | 0.70160275 | 0.622570158 |
| STT3B | 0.877201552 | 0.779933575 |
| PRKCI | 0.838094069 | 0.682312211 |
| SMS | 0.819926454 | 0.774728948 |
| MTDH | 0.866914748 | 0.764820522 |
| NUSAP1 | 0.842755067 | 0.787872852 |
| CDH17 | 0.627893489 | 0.526433293 |
| PPIL4 | 0.832055607 | 0.630107684 |
| CAAP1 | 0.812031146 | 0.638496585 |
| MET | 0.818577795 | 0.82353715 |
| CDKN2AIP | 0.771534165 | 0.638933478 |
| MFAP1 | 0.683061379 | 0.608436429 |
| UFL1 | 0.81254193 | 0.609165648 |
| EBPL | 0.759059044 | 0.752787074 |
| RAB1A | 0.814544745 | 0.626386236 |
| TFB2M | 0.856619794 | 0.791407683 |
| SPTLC1 | 0.805757281 | 0.671074605 |
| SNW1 | 0.698184111 | 0.573189451 |
| GRPEL2 | 0.886573769 | 0.820336689 |
| SKP2 | 0.885767485 | 0.804456089 |
| RPS6KA3 | 0.728456818 | 0.627304929 |
| NOP14 | 0.803621231 | 0.842834471 |
| RAC1 | 0.762725017 | 0.699804386 |
| PLEKHS1 | 0.623677718 | 0.708597877 |
| SUMO1 | 0.799256834 | 0.5856821 |
| ARHGAP11A | 0.83315488 | 0.753938315 |
| ATAD2 | 0.868640447 | 0.809004439 |
| CDK1 | 0.868266777 | 0.786612102 |
| YME1L1 | 0.833585087 | 0.616370346 |
| KPNA4 | 0.843104885 | 0.716340478 |
| HSPD1 | 0.806796772 | 0.733627792 |
| PMAIP1 | 0.766296482 | 0.774644115 |
| DCK | 0.876052851 | 0.709692436 |
| ZNF146 | 0.841870401 | 0.735766461 |
| PCLAF | 0.621250465 | 0.589210409 |
| VHL | 0.832368509 | 0.780883047 |
| DARS2 | 0.845170455 | 0.765339582 |
| ARFGAP3 | 0.573945557 | 0.522817266 |
| ADAM9 | 0.616706248 | 0.51524351 |
| BZW2 | 0.827713438 | 0.781401428 |
| CKAP2 | 0.889614549 | 0.821123469 |
| CPSF3 | 0.843460228 | 0.837998158 |
| SF3A3 | 0.79795501 | 0.776789162 |
| CXCL1 | 0.581653125 | 0.697667586 |
| NARS2 | 0.82896672 | 0.776184883 |
| CDCA7 | 0.803607454 | 0.773427026 |
| SCD | 0.707759782 | 0.754792383 |
| LMNB1 | 0.815792434 | 0.799304396 |
| PWP1 | 0.830445286 | 0.821559001 |
| C1GALT1 | 0.830847236 | 0.718347433 |
| PRR13P5 | 0.59559608 | 0.57212068 |
| STRN | 0.833782282 | 0.712635773 |
| UFM1 | 0.746926443 | 0.652950036 |
| CLIC1 | 0.804074831 | 0.85284647 |
| RAD21 | 0.761190482 | 0.584466265 |
| ERO1A | 0.788461794 | 0.764390716 |
| TIMM23 | 0.888285807 | 0.846691959 |
| AGPS | 0.871718414 | 0.716652098 |
| NUP43 | 0.89538399 | 0.841918199 |
| MRPL1 | 0.711043384 | 0.573183986 |
| PSME1 | 0.542884263 | 0.565694224 |
| ARCN1 | 0.818203222 | 0.695497234 |
| AIMP1 | 0.8295534 | 0.653167037 |
| HDGF | 0.764044526 | 0.773658248 |
| ZKSCAN1 | 0.654452102 | 0.625282241 |
| MTX2 | 0.836398627 | 0.725074744 |
| YBX1 | 0.763354897 | 0.716018197 |
| GTPBP4 | 0.5680062 | 0.530283522 |
| MMADHC | 0.816275655 | 0.646143937 |
| KPNA3 | 0.839533254 | 0.727490701 |
| RMND1 | 0.823471875 | 0.776469307 |
| RHOA | 0.814523424 | 0.715534595 |
| TFCP2 | 0.825947236 | 0.775586443 |
| PNO1 | 0.855422062 | 0.806705801 |
| ATP6V1C1 | 0.735157398 | 0.653678434 |
| RAP2C | 0.741784114 | 0.62241228 |
| SKA3 | 0.84015907 | 0.824561914 |
| TRMT10C | 0.878865524 | 0.791016618 |
| THRAP3 | 0.713312287 | 0.605906738 |
| NUF2 | 0.827236496 | 0.774117232 |
| C1GALT1C1 | 0.822853675 | 0.772167382 |
| TFRC | 0.72047396 | 0.661035936 |
| TMEM97 | 0.809354262 | 0.814983778 |
| CEP55 | 0.85794564 | 0.816615042 |
| RSL24D1 | 0.700698572 | 0.548450896 |
| TGS1 | 0.847571413 | 0.815953297 |
| DEK | 0.804434335 | 0.668081393 |
| TM9SF3 | 0.812686793 | 0.7120122 |
| YWHAZ | 0.663771417 | 0.514053319 |
| STAM | 0.878296842 | 0.806144091 |
| PI4K2B | 0.865640135 | 0.74055414 |
| LYPLA1 | 0.790139779 | 0.677622231 |
| CXCL3 | 0.578687792 | 0.6872421 |
| BUD13 | 0.774838801 | 0.766490619 |
| MMP12 | 0.576465811 | 0.645170977 |
| PPP6C | 0.844607012 | 0.683677162 |
| BUB1 | 0.862146855 | 0.833413119 |
| CXCL10 | 0.559707345 | 0.624633181 |
| CCDC43 | 0.896462832 | 0.777295526 |
| RRN3 | 0.838554913 | 0.718722127 |
| TRUB1 | 0.909929961 | 0.739182628 |
| SPC25 | 0.857070409 | 0.820536062 |
| CDK7 | 0.79404646 | 0.752396336 |
| GGCT | 0.78421815 | 0.725213167 |
| KDELR2 | 0.820050773 | 0.780047762 |
| EIF3J | 0.795663641 | 0.622149915 |
| CHAMP1 | 0.812839565 | 0.71682831 |
| GFPT1 | 0.792417188 | 0.694211671 |
| WDR45B | 0.686170191 | 0.736737156 |
| MTERF3 | 0.754814739 | 0.728208293 |
| ARF4 | 0.773759498 | 0.651383475 |
| MCTS1 | 0.610854169 | 0.569027013 |
| TMEM123 | 0.817467951 | 0.717579153 |
| YTHDF2 | 0.817794534 | 0.723845001 |
| CAPZA1 | 0.854577991 | 0.723934271 |
| SYAP1 | 0.847707588 | 0.7624767 |
| DLAT | 0.784980744 | 0.595020179 |
| ADK | 0.772219166 | 0.73298584 |
| NIP7 | 0.862625717 | 0.826689797 |
| NUDCD1 | 0.81684448 | 0.774822707 |
| KDELC2 | 0.718674269 | 0.595385591 |
| PUM3 | 0.818995105 | 0.777180263 |
| MRPL45 | 0.819009335 | 0.786911981 |
| ARFIP1 | 0.75383432 | 0.547353163 |
| HPRT1 | 0.877946212 | 0.807437033 |
| C6orf62 | 0.843508857 | 0.727055951 |
| TXNDC9 | 0.764521396 | 0.565349367 |
| RRM1 | 0.850296135 | 0.793705435 |
| VBP1 | 0.882856801 | 0.738908373 |
| LRP11 | 0.830942419 | 0.813049904 |
| TMEM184C | 0.834700648 | 0.676882895 |
| SLC39A6 | 0.709117531 | 0.706283917 |
| ME1 | 0.633013989 | 0.659005799 |
| SNRPD3 | 0.569035886 | 0.568355152 |
| FANCI | 0.819338018 | 0.799185707 |
| TSEN15 | 0.733298196 | 0.65874053 |
| SYNCRIP | 0.836982609 | 0.726019959 |
| SET | 0.80667821 | 0.707938629 |
| MCUR1 | 0.737434567 | 0.684078703 |
| MAL2 | 0.807394468 | 0.713329391 |
| PSPH | 0.736312494 | 0.785326252 |
| PTMA | 0.591753984 | 0.551990677 |
| TAF9 | 0.624340862 | 0.58863511 |
| ABI1 | 0.855108999 | 0.722038473 |
| NUDT21 | 0.896213856 | 0.763586981 |
| RPL7L1 | 0.747664658 | 0.666260773 |
| MIS18A | 0.825181328 | 0.81163809 |
| GTF2E2 | 0.594096659 | 0.629686668 |
| EPRS | 0.848348538 | 0.774209021 |
| DSCC1 | 0.825485646 | 0.807709407 |
| LARP1 | 0.719770128 | 0.739706952 |
| ETS2 | 0.714076388 | 0.732808924 |
| LIN7C | 0.786665642 | 0.676774783 |
| NEBL | 0.798378194 | 0.814300283 |
| BAZ1B | 0.780024479 | 0.744471277 |
| TRAM1 | 0.806384617 | 0.682001704 |
| SNORA33 | 0.595403092 | 0.607811774 |
| UHMK1 | 0.797809717 | 0.658226601 |
| ADSS | 0.874475481 | 0.759069661 |
| BRCC3 | 0.853033227 | 0.772891407 |
| RALA | 0.880022844 | 0.801737779 |
| CEMIP | 0.666531137 | 0.785316515 |
| YWHAG | 0.855618742 | 0.823198947 |
| UBA2 | 0.626128757 | 0.503202711 |
| UBE2K | 0.787734752 | 0.631307727 |
| USP10 | 0.812183557 | 0.761311643 |
| COMMD8 | 0.799004217 | 0.640952767 |
| MRPL50 | 0.892002317 | 0.804151023 |
| COPB1 | 0.821299775 | 0.707078984 |
| EI24 | 0.800894757 | 0.722556421 |
| UTP18 | 0.851402885 | 0.811262728 |
| MSH2 | 0.881630152 | 0.799563164 |
| HOXB9 | 0.579954899 | 0.638113133 |
| STRAP | 0.865957589 | 0.775630567 |
| PTBP3 | 0.876740225 | 0.786289069 |
| PPA1 | 0.713381478 | 0.686010023 |
| NIFK | 0.861291484 | 0.761127757 |
| UBXN4 | 0.820225785 | 0.667160411 |
| BRI3BP | 0.812791084 | 0.756586683 |
| LMAN1 | 0.705567915 | 0.610709781 |
| TRIM2 | 0.755775958 | 0.692131857 |
| PGRMC1 | 0.81172746 | 0.723342563 |
| DNAJA1 | 0.797755517 | 0.758861854 |
| TXN | 0.810163822 | 0.733711523 |
| NQO1 | 0.570411463 | 0.590651417 |
| TXNDC12 | 0.866560132 | 0.832121267 |
| TNKS2 | 0.745189137 | 0.639239206 |
| EIF3A | 0.685542372 | 0.601984185 |
| NOL11 | 0.734934562 | 0.611772584 |
| TSR1 | 0.628425608 | 0.655599153 |
| BTBD1 | 0.785141847 | 0.605241299 |
| SSR3 | 0.671395568 | 0.535087134 |
| VMA21 | 0.806232394 | 0.815129052 |
| HSP90B1 | 0.714687122 | 0.618126672 |
| HINT3 | 0.800979192 | 0.640499012 |
| PDIA6 | 0.697984289 | 0.694258173 |
| PTGES3 | 0.838963369 | 0.704965224 |
| YY1 | 0.69455754 | 0.624650047 |
| NOL10 | 0.905293495 | 0.825970531 |
| AVEN | 0.631281177 | 0.671459047 |
| EMC7 | 0.664150628 | 0.604679142 |
| PSMG1 | 0.564134186 | 0.548445523 |
| LPGAT1 | 0.78865464 | 0.746620945 |
| SLC30A9 | 0.717617341 | 0.5151902 |
| CHRAC1 | 0.679505033 | 0.613226531 |
| TMPO | 0.85491809 | 0.742527831 |
| TMED7 | 0.746870561 | 0.580846269 |
| ATE1 | 0.817417405 | 0.635553816 |
| GSTO1 | 0.576297017 | 0.599467336 |
| EIF1AX | 0.76404761 | 0.637167383 |
| SELENOF | 0.820388866 | 0.679698968 |
| SKA2 | 0.675380983 | 0.519432235 |
| TTC27 | 0.862654712 | 0.812977303 |
| LGR5 | 0.598300569 | 0.698265129 |
| TUBB | 0.724332005 | 0.746091207 |
| RARS | 0.872806664 | 0.776913331 |
| NFE2L3 | 0.835693369 | 0.838258557 |
| PQLC1 | 0.742657787 | 0.694631498 |
| CRIPT | 0.825573608 | 0.691691605 |
| SMG7 | 0.746241353 | 0.699933443 |
| USP1 | 0.903784363 | 0.730127986 |
| DNAJC15 | 0.681248707 | 0.633289589 |
| MRPL3 | 0.913033953 | 0.834013995 |
| GOLIM4 | 0.722570193 | 0.643912079 |
| CMPK1 | 0.777140901 | 0.632391802 |
| SPRED1 | 0.742222881 | 0.658054952 |
| DENR | 0.925970449 | 0.798686501 |
| MRPL47 | 0.847226421 | 0.755767592 |
| EPSTI1 | 0.618126488 | 0.639867747 |
| HOXB8 | 0.529486996 | 0.640122274 |
| NXT2 | 0.841424204 | 0.740079305 |
| GRPEL1 | 0.613828869 | 0.590660603 |
| MIPEP | 0.730751846 | 0.757439955 |
| CKAP5 | 0.726188397 | 0.697513635 |
| RIOK1 | 0.821917976 | 0.750172282 |
| C1orf43 | 0.797850931 | 0.766238463 |
| PSAT1 | 0.715476291 | 0.77264893 |
| SINHCAF | 0.839476214 | 0.748203376 |
| CBX3 | 0.755039578 | 0.673267485 |
| TWISTNB | 0.904385708 | 0.804129449 |
| ODC1 | 0.776752994 | 0.791311378 |
| SLC38A1 | 0.709968678 | 0.58386388 |
| DNAJC3 | 0.806785963 | 0.727560347 |
| CREG1 | 0.73462277 | 0.644004952 |
| NCBP1 | 0.882685761 | 0.777577115 |
| KBTBD2 | 0.76010688 | 0.750850906 |
| IER3IP1 | 0.790280326 | 0.647777948 |
| SPAG5 | 0.584791465 | 0.610050054 |
| TOMM70 | 0.906999712 | 0.758491643 |
| RIDA | 0.697497782 | 0.592080838 |
| YIPF5 | 0.757198783 | 0.618781809 |
| DDX1 | 0.772568179 | 0.637702139 |
| ENOPH1 | 0.868634681 | 0.866293043 |
| PPP1R15B | 0.810268545 | 0.674553631 |
| RAD23B | 0.913297428 | 0.820075873 |
| RPS6 | 0.578799036 | 0.643983001 |
| TAF7 | 0.732050224 | 0.656400718 |
| ARMC1 | 0.851583845 | 0.69079795 |
| STARD7 | 0.880139704 | 0.784677987 |
| TBL1XR1 | 0.690946361 | 0.543411776 |
| POLR2D | 0.847397973 | 0.859358147 |
| TAP1 | 0.545242082 | 0.639547122 |
| RHNO1 | 0.809115177 | 0.788847208 |
| CPNE3 | 0.830973792 | 0.719169478 |
| SPRYD7 | 0.731555377 | 0.68129724 |
| BCAS2 | 0.829740859 | 0.676356244 |
| GTF3A | 0.577178091 | 0.622909297 |
| CNBP | 0.786059062 | 0.665398754 |
| SLC12A2 | 0.748384295 | 0.7659478 |
| CCL20 | 0.613804803 | 0.618565643 |
| ARL5B | 0.828865832 | 0.739163562 |
| CHMP2B | 0.770270944 | 0.560955459 |
| FAM199X | 0.836973306 | 0.750001477 |
| TMEM126A | 0.650935089 | 0.57693169 |
| NUDT15 | 0.854236346 | 0.776980893 |
| GTF3C6 | 0.630611929 | 0.538312463 |
| SF3B6 | 0.818097309 | 0.732286175 |
| PPP4R2 | 0.827797021 | 0.627358652 |
| HACD3 | 0.84819622 | 0.823507148 |
| DDX18 | 0.844650985 | 0.674977116 |
| IER3 | 0.600674498 | 0.683092417 |
| IRF2BP2 | 0.555673676 | 0.570790887 |
| HMGN4 | 0.861148385 | 0.751697557 |
| RAB5A | 0.732405927 | 0.547408769 |
| SNRPC | 0.709375944 | 0.772425558 |
| IPO5 | 0.601256968 | 0.5538683 |
| CDC123 | 0.834443683 | 0.816767171 |
| RMI1 | 0.883410595 | 0.74668269 |
| LURAP1L | 0.532925647 | 0.512764124 |
| ANP32E | 0.767994699 | 0.679483538 |
| PHLDA1 | 0.656786113 | 0.744937714 |
| AL035661.1 | 0.623839192 | 0.617470415 |
| PLAU | 0.610701386 | 0.743050266 |
| TMEM128 | 0.675749031 | 0.59209648 |
| RAB14 | 0.826163736 | 0.666192339 |
| NRBF2 | 0.78165231 | 0.654825727 |
| TAGLN2 | 0.746374044 | 0.839517646 |
| LIF | 0.518620306 | 0.70448693 |
| CDKN1B | 0.722693557 | 0.593272179 |
| WASHC5 | 0.785087765 | 0.716518857 |
| MRTO4 | 0.745968067 | 0.812266127 |
| VDAC1 | 0.831914807 | 0.759843446 |
| KHDRBS1 | 0.91364636 | 0.803331481 |
| STK26 | 0.881114968 | 0.753043726 |
| MFSD14B | 0.888339188 | 0.79545244 |
| PRPS1 | 0.713324695 | 0.750612329 |
| CCT3 | 0.756984972 | 0.784857638 |
| ESYT2 | 0.624096467 | 0.542534797 |
| RANBP2 | 0.673354622 | 0.53709605 |
| VPS26A | 0.882900558 | 0.6531376 |
| TOP1 | 0.85404505 | 0.7573692 |
| HTATSF1 | 0.715604106 | 0.690839007 |
| RFWD3 | 0.861483217 | 0.864467543 |
| IARS | 0.769425186 | 0.743440622 |
| CD46 | 0.650124879 | 0.604188094 |
| TIMM10B | 0.78362265 | 0.670615359 |
| TMED2 | 0.873876947 | 0.769543267 |
| YEATS4 | 0.849868552 | 0.787002609 |
| TAF11 | 0.754662583 | 0.66040161 |
| E2F3 | 0.86098154 | 0.836785576 |
| HSPA8 | 0.750307957 | 0.718776939 |
| MFSD14A | 0.775695527 | 0.622378333 |
| LACTB2 | 0.747176864 | 0.719805295 |
| SLC25A15 | 0.776441146 | 0.809177368 |
| AGGF1 | 0.895629866 | 0.756820989 |
| WBP11 | 0.82261748 | 0.779030401 |
| SNRPB2 | 0.684315329 | 0.611987261 |
| TBC1D4 | 0.790263896 | 0.74468129 |
| AZIN1 | 0.849063078 | 0.742007867 |
| GMCL1 | 0.888155733 | 0.724757812 |
| BRK1 | 0.652358586 | 0.596952334 |
| MMP9 | 0.548609384 | 0.645997241 |
| JPH1 | 0.763188704 | 0.806948226 |
| MMP1 | 0.549254118 | 0.65762765 |
| ACTR2 | 0.853391418 | 0.706333692 |
| IFNAR1 | 0.784570287 | 0.678023289 |
| CWC22 | 0.82406695 | 0.658734221 |
| SCAMP1 | 0.82317054 | 0.666268281 |
| ARMT1 | 0.888323934 | 0.709102032 |
| ATAD1 | 0.736804471 | 0.549498176 |
| STK38 | 0.659416158 | 0.561053331 |
| GART | 0.682260618 | 0.633544976 |
| NOP58 | 0.729642906 | 0.653382134 |
| NUP37 | 0.740873685 | 0.743105518 |
| NUP153 | 0.847909879 | 0.729590099 |
| FUNDC1 | 0.765061966 | 0.753589117 |
| ABHD10 | 0.836547645 | 0.776471179 |
| USO1 | 0.797738839 | 0.629618491 |
| ROCK2 | 0.704791626 | 0.604454759 |
| IRS2 | 0.549876636 | 0.589540181 |
| PDS5A | 0.808432228 | 0.64210004 |
| TMEM87B | 0.812587641 | 0.679289016 |
| LRPPRC | 0.817362486 | 0.686537349 |
| PUM2 | 0.790880038 | 0.661234033 |
| BUB1B | 0.812991972 | 0.782940836 |
| MRPS31 | 0.670250808 | 0.616202764 |
| KDM1B | 0.832826427 | 0.743306127 |
| POLE3 | 0.84182195 | 0.80439753 |
| CCT4 | 0.850005287 | 0.813910281 |
| IPO7 | 0.867719435 | 0.742634249 |

Table S3. The clinical data of the whole colorectal cancer from TCGA cohort.

| id | futime | fustat |
| --- | --- | --- |
| TCGA-AA-3842 | 3.084932 | alive |
| TCGA-F4-6806 | 3.452055 | alive |
| TCGA-A6-A56B | 4.687671 | dead |
| TCGA-CM-5863 | 1.252055 | alive |
| TCGA-QG-A5Z2 | 2.608219 | alive |
| TCGA-CM-6168 | 1.082192 | alive |
| TCGA-A6-3810 | 3.043836 | alive |
| TCGA-CM-4750 | 0.668493 | alive |
| TCGA-AZ-6600 | 1.008219 | dead |
| TCGA-5M-AATE | 3.287671 | alive |
| TCGA-AA-3496 | 0.084932 | alive |
| TCGA-AA-3930 | 0.167123 | dead |
| TCGA-AA-A01V | 0.084932 | alive |
| TCGA-CM-5349 | 2.506849 | alive |
| TCGA-AA-3519 | 0.756164 | alive |
| TCGA-AA-3970 | 3.00274 | alive |
| TCGA-A6-2675 | 3.619178 | alive |
| TCGA-AA-3841 | 3.079452 | alive |
| TCGA-AA-3713 | 1.586301 | alive |
| TCGA-AA-A02R | 1.835616 | dead |
| TCGA-AA-3821 | 0.084932 | alive |
| TCGA-D5-6920 | 1.032877 | alive |
| TCGA-A6-6650 | 1.717808 | alive |
| TCGA-AA-3517 | 3.249315 | alive |
| TCGA-AA-3494 | 0.084932 | alive |
| TCGA-AA-3538 | 2.167123 | alive |
| TCGA-CM-6680 | 1.00274 | alive |
| TCGA-QG-A5Z1 | 0.70137 | dead |
| TCGA-AA-3514 | 0.084932 | alive |
| TCGA-NH-A8F8 | 1.4 | dead |
| TCGA-F4-6809 | 1.10411 | dead |
| TCGA-AA-3851 | 2.756164 | alive |
| TCGA-AD-6889 | 6.936986 | dead |
| TCGA-CM-5344 | 1.835616 | alive |
| TCGA-AA-3663 | 0.580822 | alive |
| TCGA-AA-3860 | 2.589041 | alive |
| TCGA-A6-4107 | 2.70411 | alive |
| TCGA-F4-6807 | 3.586301 | alive |
| TCGA-AA-A010 | 2.915068 | alive |
| TCGA-A6-5661 | 2.794521 | alive |
| TCGA-DM-A28A | 2.205479 | dead |
| TCGA-5M-AAT4 | 0.134247 | dead |
| TCGA-AA-3516 | 1.084932 | dead |
| TCGA-AA-3692 | 3 | dead |
| TCGA-AA-A00D | 1.583562 | alive |
| TCGA-F4-6855 | 3.950685 | alive |
| TCGA-AA-A01I | 2.583562 | alive |
| TCGA-DM-A285 | 0.490411 | dead |
| TCGA-AZ-6599 | 0.564384 | dead |
| TCGA-F4-6704 | 0.128767 | alive |
| TCGA-A6-5662 | 1.967123 | alive |
| TCGA-G4-6625 | 7.649315 | alive |
| TCGA-F4-6460 | 2.663014 | dead |
| TCGA-DM-A1DB | 3.693151 | dead |
| TCGA-G4-6310 | 5.30137 | alive |
| TCGA-5M-AAT6 | 0.794521 | dead |
| TCGA-AA-A01Q | 0.084932 | alive |
| TCGA-AZ-6603 | 2.463014 | dead |
| TCGA-CM-5860 | 2.668493 | alive |
| TCGA-D5-5538 | 4.550685 | dead |
| TCGA-AA-3950 | 2 | alive |
| TCGA-AA-A02Y | 3.331507 | alive |
| TCGA-AA-3488 | 0.419178 | dead |
| TCGA-F4-6570 | 0.515068 | dead |
| TCGA-G4-6322 | 2.169863 | alive |
| TCGA-AA-3680 | 0.917808 | dead |
| TCGA-A6-6782 | 1.690411 | alive |
| TCGA-NH-A50U | 0.915068 | dead |
| TCGA-A6-2677 | 2.027397 | dead |
| TCGA-A6-6653 | 2.032877 | alive |
| TCGA-AA-3710 | 2.249315 | alive |
| TCGA-D5-6923 | 1.035616 | alive |
| TCGA-AA-3973 | 1.087671 | alive |
| TCGA-A6-6781 | 1.638356 | alive |
| TCGA-A6-2679 | 3.742466 | alive |
| TCGA-CK-5912 | 4.090411 | dead |
| TCGA-G4-6321 | 1.841096 | alive |
| TCGA-F4-6856 | 2.942466 | alive |
| TCGA-AA-A01X | 2.167123 | alive |
| TCGA-CK-6751 | 1.419178 | alive |
| TCGA-A6-4105 | 1.210959 | dead |
| TCGA-DM-A28C | 6.780822 | dead |
| TCGA-DM-A28E | 9.994521 | alive |
| TCGA-AA-3495 | 3.087671 | alive |
| TCGA-CK-4952 | 1.30137 | alive |
| TCGA-AA-3660 | 6.506849 | alive |
| TCGA-A6-2674 | 3.646575 | alive |
| TCGA-AA-A01T | 2.753425 | alive |
| TCGA-F4-6805 | 2.868493 | alive |
| TCGA-CM-4743 | 1.920548 | alive |
| TCGA-A6-6138 | 1.876712 | alive |
| TCGA-CK-4951 | 5.846575 | dead |
| TCGA-F4-6463 | 2.978082 | alive |
| TCGA-AA-3548 | 2.832877 | alive |
| TCGA-D5-6538 | 1.427397 | alive |
| TCGA-AY-5543 | 2.750685 | alive |
| TCGA-AA-A00R | 0.082192 | alive |
| TCGA-AY-4070 | 1.358904 | dead |
| TCGA-D5-6930 | 1.112329 | alive |
| TCGA-AA-3831 | 1.49863 | alive |
| TCGA-A6-2676 | 3.575342 | dead |
| TCGA-G4-6627 | 5.958904 | alive |
| TCGA-AA-3982 | 2.252055 | alive |
| TCGA-CM-4744 | 1.668493 | alive |
| TCGA-D5-6898 | 0.627397 | alive |
| TCGA-AA-A00F | 2.835616 | alive |
| TCGA-CA-6717 | 1.063014 | alive |
| TCGA-G4-6314 | 2.994521 | alive |
| TCGA-A6-5656 | 2.742466 | alive |
| TCGA-AA-A01S | 0.084932 | alive |
| TCGA-AD-5900 | 1.013699 | alive |
| TCGA-A6-6142 | 2.090411 | alive |
| TCGA-DM-A1HB | 11.30411 | alive |
| TCGA-AA-3502 | 2.917808 | alive |
| TCGA-AA-3866 | 1.419178 | alive |
| TCGA-CA-6719 | 1.191781 | alive |
| TCGA-A6-3808 | 2.778082 | alive |
| TCGA-AA-3532 | 2.416438 | alive |
| TCGA-CM-6161 | 1.252055 | alive |
| TCGA-AZ-6607 | 0.265753 | dead |
| TCGA-AA-3685 | 3.087671 | alive |
| TCGA-CA-5254 | 1.057534 | alive |
| TCGA-AA-3955 | 1.747945 | alive |
| TCGA-AA-3715 | 1.586301 | dead |
| TCGA-D5-6929 | 1.117808 | alive |
| TCGA-CM-4746 | 3.084932 | alive |
| TCGA-AA-3848 | 0.838356 | dead |
| TCGA-AA-3977 | 2.084932 | alive |
| TCGA-D5-6927 | 0.786301 | alive |
| TCGA-AA-3939 | 1.082192 | alive |
| TCGA-AZ-4615 | 2.745205 | alive |
| TCGA-AZ-4684 | 5.416438 | alive |
| TCGA-D5-6541 | 1.29863 | alive |
| TCGA-AA-3864 | 4.416438 | alive |
| TCGA-AA-3531 | 2.835616 | alive |
| TCGA-AA-3510 | 5.331507 | alive |
| TCGA-CM-6162 | 1 | alive |
| TCGA-A6-6141 | 0.69863 | alive |
| TCGA-AA-3979 | 2 | alive |
| TCGA-CA-5256 | 1.038356 | alive |
| TCGA-A6-5659 | 2.536986 | alive |
| TCGA-AA-3858 | 2.589041 | alive |
| TCGA-AA-A01Z | 3.084932 | alive |
| TCGA-D5-6924 | 1.191781 | alive |
| TCGA-A6-6649 | 2.013699 | alive |
| TCGA-A6-6654 | 1.989041 | alive |
| TCGA-AZ-6606 | 0.978082 | dead |
| TCGA-AA-3867 | 2.00274 | alive |
| TCGA-D5-5541 | 4.660274 | alive |
| TCGA-A6-5657 | 2.635616 | alive |
| TCGA-AA-A017 | 1.252055 | alive |
| TCGA-AA-A00L | 3.169863 | alive |
| TCGA-DM-A0X9 | 9.975342 | alive |
| TCGA-CA-6716 | 1.016438 | alive |
| TCGA-A6-2681 | 3.8 | alive |
| TCGA-AA-3869 | 2.252055 | dead |
| TCGA-AA-3678 | 3.917808 | alive |
| TCGA-AA-A01C | 1.252055 | alive |
| TCGA-AA-A00K | 1.50411 | alive |
| TCGA-D5-6926 | 0.753425 | alive |
| TCGA-A6-2682 | 1.161644 | dead |
| TCGA-AY-6386 | 1.484932 | alive |
| TCGA-AA-3976 | 2.167123 | alive |
| TCGA-AA-3815 | 2.753425 | alive |
| TCGA-AA-3994 | 2.252055 | alive |
| TCGA-CM-4747 | 2.084932 | alive |
| TCGA-G4-6317 | 3 | alive |
| TCGA-AA-3560 | 1.665753 | alive |
| TCGA-AA-3666 | 0.167123 | dead |
| TCGA-CA-6715 | 1.049315 | alive |
| TCGA-CK-4948 | 12.33425 | alive |
| TCGA-DM-A28H | 9.756164 | alive |
| TCGA-F4-6703 | 3.989041 | alive |
| TCGA-G4-6588 | 2.180822 | alive |
| TCGA-AZ-6605 | 0.435616 | dead |
| TCGA-AA-3968 | 1.832877 | alive |
| TCGA-RU-A8FL | 3.224658 | alive |
| TCGA-AA-3975 | 2.838356 | alive |
| TCGA-CM-6170 | 1.252055 | alive |
| TCGA-AZ-4323 | 0.117808 | dead |
| TCGA-AA-3552 | 1.084932 | dead |
| TCGA-AA-A02K | 1.167123 | dead |
| TCGA-AA-3664 | 4.50137 | alive |
| TCGA-CK-6748 | 0.167123 | alive |
| TCGA-AD-6548 | 1.780822 | alive |
| TCGA-AA-A00U | 1.419178 | alive |
| TCGA-D5-6532 | 1.520548 | alive |
| TCGA-AZ-4614 | 0.471233 | dead |
| TCGA-CM-6678 | 0.917808 | alive |
| TCGA-AA-A02F | 3.331507 | alive |
| TCGA-AA-3956 | 2.835616 | alive |
| TCGA-NH-A6GB | 1.30411 | alive |
| TCGA-AA-3542 | 1.082192 | alive |
| TCGA-AA-3667 | 1.167123 | alive |
| TCGA-AA-3522 | 3.087671 | alive |
| TCGA-DM-A1D8 | 1.049315 | dead |
| TCGA-AZ-4616 | 0.427397 | dead |
| TCGA-AA-3819 | 2.084932 | alive |
| TCGA-CA-5255 | 1.030137 | alive |
| TCGA-AA-3949 | 2.167123 | alive |
| TCGA-D5-6531 | 1.479452 | alive |
| TCGA-G4-6315 | 5.158904 | alive |
| TCGA-CM-5864 | 1.252055 | alive |
| TCGA-AA-3947 | 2.750685 | alive |
| TCGA-DM-A280 | 0.646575 | dead |
| TCGA-D5-5539 | 1.632877 | alive |
| TCGA-AA-3681 | 0.49863 | alive |
| TCGA-CM-6165 | 1.336986 | alive |
| TCGA-AA-3544 | 1.167123 | alive |
| TCGA-D5-6534 | 3.605479 | alive |
| TCGA-AD-6899 | 0.482192 | dead |
| TCGA-DM-A28G | 5.065753 | dead |
| TCGA-CM-5861 | 1.252055 | alive |
| TCGA-D5-5537 | 3.783562 | dead |
| TCGA-AA-3524 | 3.00274 | alive |
| TCGA-AA-A00Q | 3.50137 | alive |
| TCGA-AA-3489 | 0.586301 | dead |
| TCGA-AA-3952 | 0.167123 | dead |
| TCGA-AY-A8YK | 1.569863 | alive |
| TCGA-G4-6320 | 2.20274 | alive |
| TCGA-A6-A5ZU | 0.80274 | alive |
| TCGA-D5-6536 | 1.487671 | alive |
| TCGA-AD-6964 | 0.906849 | dead |
| TCGA-A6-6780 | 1.676712 | alive |
| TCGA-T9-A92H | 0.991781 | alive |
| TCGA-DM-A28F | 2.99726 | dead |
| TCGA-AA-3526 | 1.589041 | alive |
| TCGA-A6-2678 | 3.523288 | alive |
| TCGA-CM-5862 | 0.419178 | dead |
| TCGA-QG-A5YX | 2.747945 | alive |
| TCGA-AA-3812 | 2.920548 | alive |
| TCGA-D5-6932 | 0.947945 | alive |
| TCGA-A6-2685 | 3.10411 | alive |
| TCGA-AA-3980 | 0.663014 | alive |
| TCGA-AA-3556 | 1.917808 | alive |
| TCGA-4T-AA8H | 1.054795 | alive |
| TCGA-DM-A28K | 8.186301 | alive |
| TCGA-AA-A01P | 3.172603 | dead |
| TCGA-AA-A00O | 2.252055 | alive |
| TCGA-AA-3662 | 0.50411 | alive |
| TCGA-AA-A02H | 0.167123 | dead |
| TCGA-D5-6539 | 1.041096 | alive |
| TCGA-G4-6628 | 6.641096 | alive |
| TCGA-AZ-4308 | 9.106849 | alive |
| TCGA-DM-A288 | 1.169863 | dead |
| TCGA-AA-3941 | 2 | alive |
| TCGA-AA-3870 | 2.49863 | alive |
| TCGA-AA-3673 | 4.169863 | alive |
| TCGA-NH-A6GA | 0.827397 | dead |
| TCGA-AA-3811 | 0.838356 | dead |
| TCGA-AA-A01R | 2.917808 | alive |
| TCGA-D5-6931 | 1 | alive |
| TCGA-AA-3549 | 1.750685 | alive |
| TCGA-AA-3554 | 1.49589 | alive |
| TCGA-D5-6537 | 0.4 | dead |
| TCGA-SS-A7HO | 5.010959 | alive |
| TCGA-A6-6137 | 2.257534 | alive |
| TCGA-G4-6303 | 5.487671 | dead |
| TCGA-AY-A54L | 1.438356 | alive |
| TCGA-DM-A1D0 | 10.88767 | alive |
| TCGA-CK-4950 | 7.120548 | alive |
| TCGA-G4-6295 | 0.69589 | alive |
| TCGA-D5-6922 | 0.843836 | alive |
| TCGA-A6-6652 | 2.057534 | alive |
| TCGA-G4-6294 | 2.350685 | dead |
| TCGA-DM-A28M | 7.931507 | alive |
| TCGA-AZ-6601 | 8.334247 | dead |
| TCGA-CM-6167 | 1.249315 | alive |
| TCGA-CM-4751 | 2.252055 | alive |
| TCGA-AD-6895 | 2.090411 | alive |
| TCGA-AA-3555 | 2.49589 | alive |
| TCGA-CM-6169 | 1.084932 | alive |
| TCGA-CK-5913 | 4.276712 | alive |
| TCGA-A6-2686 | 3.084932 | dead |
| TCGA-AA-3972 | 4.249315 | alive |
| TCGA-AU-6004 | 2.257534 | alive |
| TCGA-AA-3518 | 0.084932 | alive |
| TCGA-D5-6529 | 1.682192 | alive |
| TCGA-CM-4752 | 1.084932 | alive |
| TCGA-CM-5348 | 1.915068 | alive |
| TCGA-A6-6651 | 1.813699 | alive |
| TCGA-AD-A5EK | 1.369863 | alive |
| TCGA-AA-3509 | 5.246575 | alive |
| TCGA-DM-A1HA | 7.123288 | alive |
| TCGA-AA-3971 | 1.339726 | alive |
| TCGA-A6-2684 | 3.087671 | alive |
| TCGA-G4-6323 | 1.147945 | alive |
| TCGA-A6-5660 | 2.432877 | alive |
| TCGA-F4-6808 | 2.805479 | alive |
| TCGA-AD-6888 | 1.293151 | dead |
| TCGA-CM-6166 | 1.832877 | alive |
| TCGA-AA-3861 | 2.50411 | alive |
| TCGA-AY-A71X | 1.610959 | alive |
| TCGA-NH-A8F7 | 1.487671 | alive |
| TCGA-AD-6965 | 2.205479 | alive |
| TCGA-QG-A5YV | 3.564384 | alive |
| TCGA-AA-3534 | 2.416438 | alive |
| TCGA-QL-A97D | 1.824658 | alive |
| TCGA-AY-A69D | 1.487671 | alive |
| TCGA-G4-6299 | 6.213699 | alive |
| TCGA-G4-6311 | 3.284932 | alive |
| TCGA-AZ-5407 | 7.350685 | alive |
| TCGA-F4-6461 | 0.926027 | dead |
| TCGA-DM-A0XD | 2.035616 | dead |
| TCGA-AZ-4315 | 4.865753 | alive |
| TCGA-G4-6297 | 6.865753 | alive |
| TCGA-AA-A01D | 0.915068 | dead |
| TCGA-CM-6164 | 2.419178 | alive |
| TCGA-AA-3833 | 1.328767 | alive |
| TCGA-AA-3506 | 4.835616 | alive |
| TCGA-AZ-6598 | 4.117808 | dead |
| TCGA-AZ-4682 | 1.863014 | dead |
| TCGA-NH-A6GC | 1.065753 | alive |
| TCGA-D5-6535 | 1.260274 | alive |
| TCGA-D5-7000 | 0.854795 | alive |
| TCGA-AA-3862 | 2.50411 | alive |
| TCGA-DM-A1D4 | 7.728767 | dead |
| TCGA-AA-A02J | 0.419178 | dead |
| TCGA-AA-3688 | 1.583562 | alive |
| TCGA-CM-6676 | 0.923288 | alive |
| TCGA-A6-A567 | 5.153425 | dead |
| TCGA-CM-6172 | 0.917808 | alive |
| TCGA-A6-2672 | 3.887671 | alive |
| TCGA-CM-6163 | 1.169863 | alive |
| TCGA-CK-4947 | 1.463014 | alive |
| TCGA-CM-5868 | 1.419178 | alive |
| TCGA-AA-3986 | 1.589041 | alive |
| TCGA-AD-6890 | 2.043836 | alive |
| TCGA-AA-3561 | 1.161644 | alive |
| TCGA-AA-A029 | 4.331507 | alive |
| TCGA-DM-A1D6 | 1.561644 | dead |
| TCGA-CM-6674 | 1.079452 | alive |
| TCGA-AA-A02W | 3.416438 | alive |
| TCGA-AA-3562 | 1.665753 | alive |
| TCGA-CK-6747 | 2.246575 | alive |
| TCGA-CM-6171 | 1.169863 | alive |
| TCGA-AA-A02E | 0.246575 | dead |
| TCGA-A6-2671 | 3.646575 | dead |
| TCGA-AY-6197 | 1.786301 | alive |
| TCGA-A6-5665 | 1.838356 | alive |
| TCGA-DM-A282 | 11.59726 | alive |
| TCGA-CM-6679 | 0.838356 | alive |
| TCGA-AA-3855 | 2.671233 | alive |
| TCGA-A6-A566 | 2.076712 | dead |
| TCGA-G4-6586 | 2.983562 | alive |
| TCGA-CK-5916 | 1.761644 | dead |
| TCGA-AA-3989 | 0.663014 | dead |
| TCGA-AA-3966 | 0.167123 | alive |
| TCGA-CM-6677 | 0.923288 | alive |
| TCGA-A6-2683 | 1.380822 | dead |
| TCGA-AA-3520 | 2.00274 | alive |
| TCGA-NH-A50T | 1.515068 | alive |
| TCGA-AA-3877 | 2.583562 | alive |
| TCGA-AA-3837 | 3.249315 | alive |
| TCGA-DM-A1D9 | 11.69863 | alive |
| TCGA-AA-A00A | 3.169863 | alive |
| TCGA-AA-3818 | 0.082192 | dead |
| TCGA-3L-AA1B | 1.30137 | alive |
| TCGA-G4-6293 | 11.09863 | alive |
| TCGA-D5-6530 | 1.70137 | alive |
| TCGA-AA-A024 | 3.254795 | dead |
| TCGA-AA-3675 | 3.920548 | alive |
| TCGA-AZ-5403 | 5.232877 | dead |
| TCGA-DM-A1D7 | 1.109589 | dead |
| TCGA-CK-5914 | 1.832877 | alive |
| TCGA-DM-A0XF | 3.183562 | dead |
| TCGA-AA-3856 | 0.082192 | alive |
| TCGA-A6-A565 | 1.353425 | dead |
| TCGA-CA-5796 | 1.032877 | alive |
| TCGA-AA-3553 | 2 | alive |
| TCGA-AA-3696 | 0.419178 | dead |
| TCGA-QG-A5YW | 2.454795 | alive |
| TCGA-A6-3807 | 2.887671 | alive |
| TCGA-AZ-6608 | 0.161644 | dead |
| TCGA-G4-6304 | 4.468493 | alive |
| TCGA-AA-A01F | 2.668493 | alive |
| TCGA-NH-A50V | 1.610959 | alive |
| TCGA-A6-3809 | 2.728767 | alive |
| TCGA-CA-6718 | 0.838356 | dead |
| TCGA-A6-5666 | 2.726027 | alive |
| TCGA-AA-3854 | 3.00274 | alive |
| TCGA-A6-6140 | 2.010959 | alive |
| TCGA-AA-3511 | 0.580822 | alive |
| TCGA-AA-A01G | 1 | alive |
| TCGA-AA-3530 | 1.589041 | alive |
| TCGA-D5-6928 | 0.969863 | alive |
| TCGA-D5-6540 | 1.345205 | alive |
| TCGA-CM-5341 | 2.421918 | alive |
| TCGA-F4-6569 | 2.978082 | alive |
| TCGA-AA-A004 | 1.161644 | alive |
| TCGA-A6-5664 | 1.841096 | alive |
| TCGA-G4-6306 | 3.723288 | alive |
| TCGA-AA-A00J | 1.50411 | alive |
| TCGA-WS-AB45 | 5.835616 | alive |
| TCGA-A6-2680 | 2.926027 | alive |
| TCGA-A6-5667 | 2.430137 | alive |
| TCGA-AU-3779 | 1.208219 | alive |
| TCGA-A6-6648 | 2.09863 | alive |
| TCGA-AA-A03J | 3.413699 | alive |
| TCGA-AA-3655 | 5.084932 | alive |
| TCGA-AA-3844 | 1.243836 | alive |
| TCGA-AD-6963 | 2.284932 | alive |
| TCGA-D5-5540 | 4.673973 | alive |
| TCGA-CM-6675 | 1.087671 | alive |
| TCGA-AA-3697 | 7.087671 | alive |
| TCGA-DM-A1DA | 0.624658 | dead |
| TCGA-AA-A00Z | 1.832877 | alive |
| TCGA-AA-A00N | 0.334247 | dead |
| TCGA-CA-5797 | 1.049315 | alive |
| TCGA-AA-3543 | 0.082192 | alive |
| TCGA-CM-4748 | 2.169863 | alive |
| TCGA-G4-6307 | 4.586301 | alive |
| TCGA-AA-3875 | 1.50411 | alive |
| TCGA-AD-6901 | 1.868493 | dead |
| TCGA-AA-3846 | 1.419178 | alive |
| TCGA-AA-3679 | 1.252055 | alive |
| TCGA-AA-A00W | 1.249315 | alive |
| TCGA-D5-6533 | 2.123288 | alive |
| TCGA-AA-A01K | 2.583562 | alive |
| TCGA-G4-6309 | 7.123288 | alive |
| TCGA-4N-A93T | 0.4 | alive |
| TCGA-AA-A00E | 2.50137 | alive |
| TCGA-F4-6459 | 0.717808 | dead |
| TCGA-AZ-4313 | 6.328767 | alive |

**Table S4**. The clinical data of the training set from TCGA cohort.

| id | futime | fustat |
| --- | --- | --- |
| TCGA-F4-6806 | 3.452054795 | 0 |
| TCGA-CM-5863 | 1.252054795 | 0 |
| TCGA-QG-A5Z2 | 2.608219178 | 0 |
| TCGA-CM-4750 | 0.668493151 | 0 |
| TCGA-AZ-6600 | 1.008219178 | 1 |
| TCGA-AA-3930 | 0.167123288 | 1 |
| TCGA-AA-A01V | 0.084931507 | 0 |
| TCGA-AA-3841 | 3.079452055 | 0 |
| TCGA-AA-A02R | 1.835616438 | 1 |
| TCGA-AA-3821 | 0.084931507 | 0 |
| TCGA-D5-6920 | 1.032876712 | 0 |
| TCGA-A6-6650 | 1.717808219 | 0 |
| TCGA-AA-3517 | 3.249315068 | 0 |
| TCGA-AA-3538 | 2.167123288 | 0 |
| TCGA-CM-6680 | 1.002739726 | 0 |
| TCGA-QG-A5Z1 | 0.701369863 | 1 |
| TCGA-AA-3514 | 0.084931507 | 0 |
| TCGA-NH-A8F8 | 1.4 | 1 |
| TCGA-AD-6889 | 6.936986301 | 1 |
| TCGA-AA-3663 | 0.580821918 | 0 |
| TCGA-AA-3860 | 2.589041096 | 0 |
| TCGA-F4-6807 | 3.58630137 | 0 |
| TCGA-DM-A28A | 2.205479452 | 1 |
| TCGA-AA-A00D | 1.583561644 | 0 |
| TCGA-F4-6855 | 3.950684932 | 0 |
| TCGA-AA-A01I | 2.583561644 | 0 |
| TCGA-AZ-6599 | 0.564383562 | 1 |
| TCGA-F4-6460 | 2.663013699 | 1 |
| TCGA-G4-6310 | 5.301369863 | 0 |
| TCGA-5M-AAT6 | 0.794520548 | 1 |
| TCGA-AZ-6603 | 2.463013699 | 1 |
| TCGA-AA-A02Y | 3.331506849 | 0 |
| TCGA-AA-3488 | 0.419178082 | 1 |
| TCGA-G4-6322 | 2.169863014 | 0 |
| TCGA-AA-3680 | 0.917808219 | 1 |
| TCGA-A6-6782 | 1.690410959 | 0 |
| TCGA-A6-2677 | 2.02739726 | 1 |
| TCGA-AA-3710 | 2.249315068 | 0 |
| TCGA-AA-3973 | 1.087671233 | 0 |
| TCGA-CK-5912 | 4.090410959 | 1 |
| TCGA-F4-6856 | 2.942465753 | 0 |
| TCGA-AA-A01X | 2.167123288 | 0 |
| TCGA-DM-A28C | 6.780821918 | 1 |
| TCGA-AA-3495 | 3.087671233 | 0 |
| TCGA-AA-3660 | 6.506849315 | 0 |
| TCGA-A6-2674 | 3.646575342 | 0 |
| TCGA-A6-6138 | 1.876712329 | 0 |
| TCGA-AY-5543 | 2.750684932 | 0 |
| TCGA-AA-3982 | 2.252054795 | 0 |
| TCGA-A6-6142 | 2.090410959 | 0 |
| TCGA-DM-A1HB | 11.30410959 | 0 |
| TCGA-AA-3866 | 1.419178082 | 0 |
| TCGA-CA-6719 | 1.191780822 | 0 |
| TCGA-A6-3808 | 2.778082192 | 0 |
| TCGA-AA-3532 | 2.416438356 | 0 |
| TCGA-CM-6161 | 1.252054795 | 0 |
| TCGA-AZ-6607 | 0.265753425 | 1 |
| TCGA-AA-3685 | 3.087671233 | 0 |
| TCGA-CA-5254 | 1.057534247 | 0 |
| TCGA-D5-6929 | 1.117808219 | 0 |
| TCGA-AA-3848 | 0.838356164 | 1 |
| TCGA-AA-3977 | 2.084931507 | 0 |
| TCGA-AZ-4615 | 2.745205479 | 0 |
| TCGA-AZ-4684 | 5.416438356 | 0 |
| TCGA-AA-3510 | 5.331506849 | 0 |
| TCGA-CM-6162 | 1 | 0 |
| TCGA-A6-6141 | 0.698630137 | 0 |
| TCGA-AA-3979 | 2 | 0 |
| TCGA-CA-5256 | 1.038356164 | 0 |
| TCGA-AA-3858 | 2.589041096 | 0 |
| TCGA-AA-A01Z | 3.084931507 | 0 |
| TCGA-A6-6649 | 2.01369863 | 0 |
| TCGA-AZ-6606 | 0.978082192 | 1 |
| TCGA-AA-3867 | 2.002739726 | 0 |
| TCGA-AA-A00L | 3.169863014 | 0 |
| TCGA-AA-3678 | 3.917808219 | 0 |
| TCGA-AA-A01C | 1.252054795 | 0 |
| TCGA-A6-2682 | 1.161643836 | 1 |
| TCGA-AA-3994 | 2.252054795 | 0 |
| TCGA-G4-6317 | 3 | 0 |
| TCGA-AA-3560 | 1.665753425 | 0 |
| TCGA-AA-3666 | 0.167123288 | 1 |
| TCGA-CK-4948 | 12.33424658 | 0 |
| TCGA-F4-6703 | 3.989041096 | 0 |
| TCGA-G4-6588 | 2.180821918 | 0 |
| TCGA-AZ-6605 | 0.435616438 | 1 |
| TCGA-RU-A8FL | 3.224657534 | 0 |
| TCGA-AA-3975 | 2.838356164 | 0 |
| TCGA-CM-6170 | 1.252054795 | 0 |
| TCGA-AA-3552 | 1.084931507 | 1 |
| TCGA-AA-A02K | 1.167123288 | 1 |
| TCGA-AA-3664 | 4.501369863 | 0 |
| TCGA-CK-6748 | 0.167123288 | 0 |
| TCGA-AA-A00U | 1.419178082 | 0 |
| TCGA-D5-6532 | 1.520547945 | 0 |
| TCGA-AZ-4614 | 0.471232877 | 1 |
| TCGA-CM-6678 | 0.917808219 | 0 |
| TCGA-AA-3542 | 1.082191781 | 0 |
| TCGA-AA-3667 | 1.167123288 | 0 |
| TCGA-AA-3522 | 3.087671233 | 0 |
| TCGA-DM-A1D8 | 1.049315068 | 1 |
| TCGA-AZ-4616 | 0.42739726 | 1 |
| TCGA-AA-3819 | 2.084931507 | 0 |
| TCGA-CA-5255 | 1.030136986 | 0 |
| TCGA-AA-3949 | 2.167123288 | 0 |
| TCGA-D5-6531 | 1.479452055 | 0 |
| TCGA-AA-3947 | 2.750684932 | 0 |
| TCGA-AA-3681 | 0.498630137 | 0 |
| TCGA-AA-3544 | 1.167123288 | 0 |
| TCGA-D5-6534 | 3.605479452 | 0 |
| TCGA-AD-6899 | 0.482191781 | 1 |
| TCGA-DM-A28G | 5.065753425 | 1 |
| TCGA-AA-3524 | 3.002739726 | 0 |
| TCGA-AA-3489 | 0.58630137 | 1 |
| TCGA-AA-3952 | 0.167123288 | 1 |
| TCGA-G4-6320 | 2.202739726 | 0 |
| TCGA-A6-A5ZU | 0.802739726 | 0 |
| TCGA-A6-6780 | 1.676712329 | 0 |
| TCGA-T9-A92H | 0.991780822 | 0 |
| TCGA-A6-2678 | 3.523287671 | 0 |
| TCGA-QG-A5YX | 2.747945205 | 0 |
| TCGA-A6-2685 | 3.104109589 | 0 |
| TCGA-AA-3980 | 0.663013699 | 0 |
| TCGA-AA-3556 | 1.917808219 | 0 |
| TCGA-4T-AA8H | 1.054794521 | 0 |
| TCGA-AA-A01P | 3.17260274 | 1 |
| TCGA-AA-3662 | 0.504109589 | 0 |
| TCGA-G4-6628 | 6.64109589 | 0 |
| TCGA-DM-A288 | 1.169863014 | 1 |
| TCGA-AA-3941 | 2 | 0 |
| TCGA-AA-3673 | 4.169863014 | 0 |
| TCGA-NH-A6GA | 0.82739726 | 1 |
| TCGA-AA-A01R | 2.917808219 | 0 |
| TCGA-AA-3549 | 1.750684932 | 0 |
| TCGA-D5-6537 | 0.4 | 1 |
| TCGA-SS-A7HO | 5.010958904 | 0 |
| TCGA-AY-A54L | 1.438356164 | 0 |
| TCGA-D5-6922 | 0.843835616 | 0 |
| TCGA-A6-6652 | 2.057534247 | 0 |
| TCGA-DM-A28M | 7.931506849 | 0 |
| TCGA-AA-3972 | 4.249315068 | 0 |
| TCGA-CM-5348 | 1.915068493 | 0 |
| TCGA-A6-6651 | 1.81369863 | 0 |
| TCGA-AA-3509 | 5.246575342 | 0 |
| TCGA-DM-A1HA | 7.123287671 | 0 |
| TCGA-AA-3861 | 2.504109589 | 0 |
| TCGA-NH-A8F7 | 1.487671233 | 0 |
| TCGA-AD-6965 | 2.205479452 | 0 |
| TCGA-QG-A5YV | 3.564383562 | 0 |
| TCGA-AA-3534 | 2.416438356 | 0 |
| TCGA-QL-A97D | 1.824657534 | 0 |
| TCGA-AY-A69D | 1.487671233 | 0 |
| TCGA-AZ-5407 | 7.350684932 | 0 |
| TCGA-DM-A0XD | 2.035616438 | 1 |
| TCGA-AZ-4315 | 4.865753425 | 0 |
| TCGA-AA-3506 | 4.835616438 | 0 |
| TCGA-AZ-6598 | 4.117808219 | 1 |
| TCGA-NH-A6GC | 1.065753425 | 0 |
| TCGA-D5-7000 | 0.854794521 | 0 |
| TCGA-AA-3688 | 1.583561644 | 0 |
| TCGA-CM-6676 | 0.923287671 | 0 |
| TCGA-A6-A567 | 5.153424658 | 1 |
| TCGA-CM-6172 | 0.917808219 | 0 |
| TCGA-A6-2672 | 3.887671233 | 0 |
| TCGA-CM-6163 | 1.169863014 | 0 |
| TCGA-CK-4947 | 1.463013699 | 0 |
| TCGA-AA-3986 | 1.589041096 | 0 |
| TCGA-AA-3561 | 1.161643836 | 0 |
| TCGA-AA-3562 | 1.665753425 | 0 |
| TCGA-CM-6679 | 0.838356164 | 0 |
| TCGA-AA-3855 | 2.671232877 | 0 |
| TCGA-A6-A566 | 2.076712329 | 1 |
| TCGA-CK-5916 | 1.761643836 | 1 |
| TCGA-CM-6677 | 0.923287671 | 0 |
| TCGA-NH-A50T | 1.515068493 | 0 |
| TCGA-AA-3837 | 3.249315068 | 0 |
| TCGA-AA-A00A | 3.169863014 | 0 |
| TCGA-3L-AA1B | 1.301369863 | 0 |
| TCGA-D5-6530 | 1.701369863 | 0 |
| TCGA-DM-A1D7 | 1.109589041 | 1 |
| TCGA-CK-5914 | 1.832876712 | 0 |
| TCGA-CA-5796 | 1.032876712 | 0 |
| TCGA-AA-3553 | 2 | 0 |
| TCGA-AA-3696 | 0.419178082 | 1 |
| TCGA-AZ-6608 | 0.161643836 | 1 |
| TCGA-A6-3809 | 2.728767123 | 0 |
| TCGA-CA-6718 | 0.838356164 | 1 |
| TCGA-A6-5666 | 2.726027397 | 0 |
| TCGA-AA-A01G | 1 | 0 |
| TCGA-AA-3530 | 1.589041096 | 0 |
| TCGA-D5-6928 | 0.969863014 | 0 |
| TCGA-D5-6540 | 1.345205479 | 0 |
| TCGA-CM-5341 | 2.421917808 | 0 |
| TCGA-A6-5664 | 1.84109589 | 0 |
| TCGA-WS-AB45 | 5.835616438 | 0 |
| TCGA-AU-3779 | 1.208219178 | 0 |
| TCGA-A6-6648 | 2.098630137 | 0 |
| TCGA-AA-3655 | 5.084931507 | 0 |
| TCGA-AD-6963 | 2.284931507 | 0 |
| TCGA-D5-5540 | 4.673972603 | 0 |
| TCGA-CM-6675 | 1.087671233 | 0 |
| TCGA-AA-A00Z | 1.832876712 | 0 |
| TCGA-AA-3543 | 0.082191781 | 0 |
| TCGA-CM-4748 | 2.169863014 | 0 |
| TCGA-AA-3846 | 1.419178082 | 0 |
| TCGA-AA-3679 | 1.252054795 | 0 |
| TCGA-D5-6533 | 2.123287671 | 0 |
| TCGA-G4-6309 | 7.123287671 | 0 |
| TCGA-4N-A93T | 0.4 | 0 |
| TCGA-AA-A00E | 2.501369863 | 0 |
| TCGA-F4-6459 | 0.717808219 | 1 |
| TCGA-AZ-4313 | 6.328767123 | 0 |

**Table S5**. The clinical data of the testing set from TCGA cohort.

| id | futime | fustat |
| --- | --- | --- |
| TCGA-AA-3842 | 3.0849315 | alive |
| TCGA-A6-A56B | 4.6876712 | dead |
| TCGA-CM-6168 | 1.0821918 | alive |
| TCGA-A6-3810 | 3.0438356 | alive |
| TCGA-5M-AATE | 3.2876712 | alive |
| TCGA-AA-3496 | 0.0849315 | alive |
| TCGA-CM-5349 | 2.5068493 | alive |
| TCGA-AA-3519 | 0.7561644 | alive |
| TCGA-AA-3970 | 3.0027397 | alive |
| TCGA-A6-2675 | 3.6191781 | alive |
| TCGA-AA-3713 | 1.5863014 | alive |
| TCGA-AA-3494 | 0.0849315 | alive |
| TCGA-F4-6809 | 1.1041096 | dead |
| TCGA-AA-3851 | 2.7561644 | alive |
| TCGA-CM-5344 | 1.8356164 | alive |
| TCGA-A6-4107 | 2.7041096 | alive |
| TCGA-AA-A010 | 2.9150685 | alive |
| TCGA-A6-5661 | 2.7945205 | alive |
| TCGA-5M-AAT4 | 0.1342466 | dead |
| TCGA-AA-3516 | 1.0849315 | dead |
| TCGA-AA-3692 | 3 | dead |
| TCGA-DM-A285 | 0.490411 | dead |
| TCGA-F4-6704 | 0.1287671 | alive |
| TCGA-A6-5662 | 1.9671233 | alive |
| TCGA-G4-6625 | 7.6493151 | alive |
| TCGA-DM-A1DB | 3.6931507 | dead |
| TCGA-AA-A01Q | 0.0849315 | alive |
| TCGA-CM-5860 | 2.6684932 | alive |
| TCGA-D5-5538 | 4.5506849 | dead |
| TCGA-AA-3950 | 2 | alive |
| TCGA-F4-6570 | 0.5150685 | dead |
| TCGA-NH-A50U | 0.9150685 | dead |
| TCGA-A6-6653 | 2.0328767 | alive |
| TCGA-D5-6923 | 1.0356164 | alive |
| TCGA-A6-6781 | 1.6383562 | alive |
| TCGA-A6-2679 | 3.7424658 | alive |
| TCGA-G4-6321 | 1.8410959 | alive |
| TCGA-CK-6751 | 1.4191781 | alive |
| TCGA-A6-4105 | 1.2109589 | dead |
| TCGA-DM-A28E | 9.9945205 | alive |
| TCGA-CK-4952 | 1.3013699 | alive |
| TCGA-AA-A01T | 2.7534247 | alive |
| TCGA-F4-6805 | 2.8684932 | alive |
| TCGA-CM-4743 | 1.9205479 | alive |
| TCGA-CK-4951 | 5.8465753 | dead |
| TCGA-F4-6463 | 2.9780822 | alive |
| TCGA-AA-3548 | 2.8328767 | alive |
| TCGA-D5-6538 | 1.4273973 | alive |
| TCGA-AA-A00R | 0.0821918 | alive |
| TCGA-AY-4070 | 1.3589041 | dead |
| TCGA-D5-6930 | 1.1123288 | alive |
| TCGA-AA-3831 | 1.4986301 | alive |
| TCGA-A6-2676 | 3.5753425 | dead |
| TCGA-G4-6627 | 5.9589041 | alive |
| TCGA-CM-4744 | 1.6684932 | alive |
| TCGA-D5-6898 | 0.6273973 | alive |
| TCGA-AA-A00F | 2.8356164 | alive |
| TCGA-CA-6717 | 1.0630137 | alive |
| TCGA-G4-6314 | 2.9945205 | alive |
| TCGA-A6-5656 | 2.7424658 | alive |
| TCGA-AA-A01S | 0.0849315 | alive |
| TCGA-AD-5900 | 1.0136986 | alive |
| TCGA-AA-3502 | 2.9178082 | alive |
| TCGA-AA-3955 | 1.7479452 | alive |
| TCGA-AA-3715 | 1.5863014 | dead |
| TCGA-CM-4746 | 3.0849315 | alive |
| TCGA-D5-6927 | 0.7863014 | alive |
| TCGA-AA-3939 | 1.0821918 | alive |
| TCGA-D5-6541 | 1.2986301 | alive |
| TCGA-AA-3864 | 4.4164384 | alive |
| TCGA-AA-3531 | 2.8356164 | alive |
| TCGA-A6-5659 | 2.5369863 | alive |
| TCGA-D5-6924 | 1.1917808 | alive |
| TCGA-A6-6654 | 1.9890411 | alive |
| TCGA-D5-5541 | 4.660274 | alive |
| TCGA-A6-5657 | 2.6356164 | alive |
| TCGA-AA-A017 | 1.2520548 | alive |
| TCGA-DM-A0X9 | 9.9753425 | alive |
| TCGA-CA-6716 | 1.0164384 | alive |
| TCGA-A6-2681 | 3.8 | alive |
| TCGA-AA-3869 | 2.2520548 | dead |
| TCGA-AA-A00K | 1.5041096 | alive |
| TCGA-D5-6926 | 0.7534247 | alive |
| TCGA-AY-6386 | 1.4849315 | alive |
| TCGA-AA-3976 | 2.1671233 | alive |
| TCGA-AA-3815 | 2.7534247 | alive |
| TCGA-CM-4747 | 2.0849315 | alive |
| TCGA-CA-6715 | 1.0493151 | alive |
| TCGA-DM-A28H | 9.7561644 | alive |
| TCGA-AA-3968 | 1.8328767 | alive |
| TCGA-AZ-4323 | 0.1178082 | dead |
| TCGA-AD-6548 | 1.7808219 | alive |
| TCGA-AA-A02F | 3.3315068 | alive |
| TCGA-AA-3956 | 2.8356164 | alive |
| TCGA-NH-A6GB | 1.3041096 | alive |
| TCGA-G4-6315 | 5.1589041 | alive |
| TCGA-CM-5864 | 1.2520548 | alive |
| TCGA-DM-A280 | 0.6465753 | dead |
| TCGA-D5-5539 | 1.6328767 | alive |
| TCGA-CM-6165 | 1.3369863 | alive |
| TCGA-CM-5861 | 1.2520548 | alive |
| TCGA-D5-5537 | 3.7835616 | dead |
| TCGA-AA-A00Q | 3.5013699 | alive |
| TCGA-AY-A8YK | 1.569863 | alive |
| TCGA-D5-6536 | 1.4876712 | alive |
| TCGA-AD-6964 | 0.9068493 | dead |
| TCGA-DM-A28F | 2.9972603 | dead |
| TCGA-AA-3526 | 1.5890411 | alive |
| TCGA-CM-5862 | 0.4191781 | dead |
| TCGA-AA-3812 | 2.9205479 | alive |
| TCGA-D5-6932 | 0.9479452 | alive |
| TCGA-DM-A28K | 8.1863014 | alive |
| TCGA-AA-A00O | 2.2520548 | alive |
| TCGA-AA-A02H | 0.1671233 | dead |
| TCGA-D5-6539 | 1.0410959 | alive |
| TCGA-AZ-4308 | 9.1068493 | alive |
| TCGA-AA-3870 | 2.4986301 | alive |
| TCGA-AA-3811 | 0.8383562 | dead |
| TCGA-D5-6931 | 1 | alive |
| TCGA-AA-3554 | 1.4958904 | alive |
| TCGA-A6-6137 | 2.2575342 | alive |
| TCGA-G4-6303 | 5.4876712 | dead |
| TCGA-DM-A1D0 | 10.887671 | alive |
| TCGA-CK-4950 | 7.1205479 | alive |
| TCGA-G4-6295 | 0.6958904 | alive |
| TCGA-G4-6294 | 2.3506849 | dead |
| TCGA-AZ-6601 | 8.3342466 | dead |
| TCGA-CM-6167 | 1.2493151 | alive |
| TCGA-CM-4751 | 2.2520548 | alive |
| TCGA-AD-6895 | 2.090411 | alive |
| TCGA-AA-3555 | 2.4958904 | alive |
| TCGA-CM-6169 | 1.0849315 | alive |
| TCGA-CK-5913 | 4.2767123 | alive |
| TCGA-A6-2686 | 3.0849315 | dead |
| TCGA-AU-6004 | 2.2575342 | alive |
| TCGA-AA-3518 | 0.0849315 | alive |
| TCGA-D5-6529 | 1.6821918 | alive |
| TCGA-CM-4752 | 1.0849315 | alive |
| TCGA-AD-A5EK | 1.369863 | alive |
| TCGA-AA-3971 | 1.339726 | alive |
| TCGA-A6-2684 | 3.0876712 | alive |
| TCGA-G4-6323 | 1.1479452 | alive |
| TCGA-A6-5660 | 2.4328767 | alive |
| TCGA-F4-6808 | 2.8054795 | alive |
| TCGA-AD-6888 | 1.2931507 | dead |
| TCGA-CM-6166 | 1.8328767 | alive |
| TCGA-AY-A71X | 1.6109589 | alive |
| TCGA-G4-6299 | 6.2136986 | alive |
| TCGA-G4-6311 | 3.2849315 | alive |
| TCGA-F4-6461 | 0.9260274 | dead |
| TCGA-G4-6297 | 6.8657534 | alive |
| TCGA-AA-A01D | 0.9150685 | dead |
| TCGA-CM-6164 | 2.4191781 | alive |
| TCGA-AA-3833 | 1.3287671 | alive |
| TCGA-AZ-4682 | 1.8630137 | dead |
| TCGA-D5-6535 | 1.260274 | alive |
| TCGA-AA-3862 | 2.5041096 | alive |
| TCGA-DM-A1D4 | 7.7287671 | dead |
| TCGA-AA-A02J | 0.4191781 | dead |
| TCGA-CM-5868 | 1.4191781 | alive |
| TCGA-AD-6890 | 2.0438356 | alive |
| TCGA-AA-A029 | 4.3315068 | alive |
| TCGA-DM-A1D6 | 1.5616438 | dead |
| TCGA-CM-6674 | 1.0794521 | alive |
| TCGA-AA-A02W | 3.4164384 | alive |
| TCGA-CK-6747 | 2.2465753 | alive |
| TCGA-CM-6171 | 1.169863 | alive |
| TCGA-AA-A02E | 0.2465753 | dead |
| TCGA-A6-2671 | 3.6465753 | dead |
| TCGA-AY-6197 | 1.7863014 | alive |
| TCGA-A6-5665 | 1.8383562 | alive |
| TCGA-DM-A282 | 11.59726 | alive |
| TCGA-G4-6586 | 2.9835616 | alive |
| TCGA-AA-3989 | 0.6630137 | dead |
| TCGA-AA-3966 | 0.1671233 | alive |
| TCGA-A6-2683 | 1.3808219 | dead |
| TCGA-AA-3520 | 2.0027397 | alive |
| TCGA-AA-3877 | 2.5835616 | alive |
| TCGA-DM-A1D9 | 11.69863 | alive |
| TCGA-AA-3818 | 0.0821918 | dead |
| TCGA-G4-6293 | 11.09863 | alive |
| TCGA-AA-A024 | 3.2547945 | dead |
| TCGA-AA-3675 | 3.9205479 | alive |
| TCGA-AZ-5403 | 5.2328767 | dead |
| TCGA-DM-A0XF | 3.1835616 | dead |
| TCGA-AA-3856 | 0.0821918 | alive |
| TCGA-A6-A565 | 1.3534247 | dead |
| TCGA-QG-A5YW | 2.4547945 | alive |
| TCGA-A6-3807 | 2.8876712 | alive |
| TCGA-G4-6304 | 4.4684932 | alive |
| TCGA-AA-A01F | 2.6684932 | alive |
| TCGA-NH-A50V | 1.6109589 | alive |
| TCGA-AA-3854 | 3.0027397 | alive |
| TCGA-A6-6140 | 2.0109589 | alive |
| TCGA-AA-3511 | 0.5808219 | alive |
| TCGA-F4-6569 | 2.9780822 | alive |
| TCGA-AA-A004 | 1.1616438 | alive |
| TCGA-G4-6306 | 3.7232877 | alive |
| TCGA-AA-A00J | 1.5041096 | alive |
| TCGA-A6-2680 | 2.9260274 | alive |
| TCGA-A6-5667 | 2.430137 | alive |
| TCGA-AA-A03J | 3.4136986 | alive |
| TCGA-AA-3844 | 1.2438356 | alive |
| TCGA-AA-3697 | 7.0876712 | alive |
| TCGA-DM-A1DA | 0.6246575 | dead |
| TCGA-AA-A00N | 0.3342466 | dead |
| TCGA-CA-5797 | 1.0493151 | alive |
| TCGA-G4-6307 | 4.5863014 | alive |
| TCGA-AA-3875 | 1.5041096 | alive |
| TCGA-AD-6901 | 1.8684932 | dead |
| TCGA-AA-A00W | 1.2493151 | alive |
| TCGA-AA-A01K | 2.5835616 | alive |

**Table S6.** The results of five hub genes by Lasso-penalized Cox analysis.

| Id | HR | HR.95L | HR.95H | pvalue |
| --- | --- | --- | --- | --- |
| PGM2 | 0.887738 | 0.82334 | 0.957172 | 0.00194 |
| PODXL | 1.021488 | 1.008154 | 1.034997 | 0.001517 |
| SCD | 1.002118 | 1.000509 | 1.003729 | 0.009842 |
| SEPHS1 | 1.068459 | 1.017845 | 1.121589 | 0.007488 |
| RHNO1 | 1.017404 | 1.006011 | 1.028926 | 0.002673 |

**Table S7.** The expression of five hub genes in CRC compared to normal**.**

| ID | logFC | pValue | fdr |
| --- | --- | --- | --- |
| PGM2 | 0.681848 | 2.45E-63 | 2.63E-63 |
| PODXL | 1.117152 | 8.42E-73 | 9.75E-73 |
| SCD | 4.10469 | 1.71E-121 | 1.00E-120 |
| SEPHS1 | 0.977045 | 1.19E-116 | 4.65E-116 |
| RHNO1 | 1.426189 | 2.58E-114 | 8.75E-114 |

**Table S8**. The results of Gene Set Analysis (GSEA).

| Number | GS follow link to MSigDB | SIZE | ES | NES | NOM p-val | FDR q-val | FWER p-val | RANK AT MAX | LEADING EDGE |
| --- | --- | --- | --- | --- | --- | --- | --- | --- | --- |
| 1 | KEGG_BASAL_CELL_CARCINOMA | 55 | 0.66 | 2.03 | 0 | 0.001 | 0.002 | 9157 | tags=60%, list=17%, signal=72% |
| 2 | KEGG_NOTCH_SIGNALING_PATHWAY | 47 | 0.59 | 1.76 | 0.002 | 0.035 | 0.133 | 7158 | tags=51%, list=13%, signal=59% |
| 3 | KEGG_GLYCOSAMINOGLYCAN_BIOSYNTHESIS_CHONDROITIN_SULFATE | 22 | 0.68 | 1.75 | 0.002 | 0.026 | 0.148 | 6707 | tags=50%, list=12%, signal=57% |
| 4 | KEGG_HEDGEHOG_SIGNALING_PATHWAY | 56 | 0.54 | 1.66 | 0.011 | 0.053 | 0.351 | 5358 | tags=39%, list=10%, signal=43% |
| 5 | KEGG_RIBOSOME | 87 | 0.46 | 1.52 | 0.016 | 0.168 | 0.812 | 8823 | tags=43%, list=16%, signal=51% |
| 6 | KEGG_AXON_GUIDANCE | 129 | 0.43 | 1.49 | 0.006 | 0.177 | 0.868 | 9229 | tags=42%, list=17%, signal=50% |
| 7 | KEGG_ECM_RECEPTOR_INTERACTION | 84 | 0.46 | 1.49 | 0.009 | 0.152 | 0.868 | 14398 | tags=57%, list=26%, signal=77% |
| 8 | KEGG_MELANOGENESIS | 101 | 0.44 | 1.49 | 0.009 | 0.138 | 0.882 | 9157 | tags=46%, list=17%, signal=55% |
| 9 | KEGG_SPLICEOSOME | 127 | 0.42 | 1.46 | 0.011 | 0.157 | 0.929 | 5806 | tags=30%, list=11%, signal=33% |
| 10 | KEGG_DILATED_CARDIOMYOPATHY | 89 | 0.44 | 1.44 | 0.017 | 0.163 | 0.951 | 10557 | tags=44%, list=19%, signal=54% |
| 11 | KEGG_VASCULAR_SMOOTH_MUSCLE_CONTRACTION | 115 | 0.41 | 1.39 | 0.022 | 0.223 | 0.979 | 9391 | tags=40%, list=17%, signal=48% |
| 12 | KEGG_WNT_SIGNALING_PATHWAY | 150 | 0.39 | 1.38 | 0.014 | 0.231 | 0.99 | 9157 | tags=39%, list=17%, signal=47% |
| 1 | KEGG_AMINO_SUGAR_AND_NUCLEOTIDE_SUGAR_METABOLISM | 43 | -0.66 | -2.08 | 0 | 0.002 | 0.001 | 4252 | tags=49%, list=8%, signal=53% |
| 2 | KEGG_FATTY_ACID_METABOLISM | 42 | -0.65 | -2.07 | 0 | 0.001 | 0.001 | 4510 | tags=43%, list=8%, signal=47% |
| 3 | KEGG_APOPTOSIS | 87 | -0.57 | -2.07 | 0 | 0.001 | 0.001 | 4598 | tags=47%, list=8%, signal=51% |
| 4 | KEGG_VALINE_LEUCINE_AND_ISOLEUCINE_DEGRADATION | 44 | -0.64 | -2.03 | 0 | 0.001 | 0.002 | 5047 | tags=52%, list=9%, signal=57% |
| 5 | KEGG_PROTEIN_EXPORT | 24 | -0.73 | -1.98 | 0 | 0.002 | 0.004 | 3974 | tags=50%, list=7%, signal=54% |
| 6 | KEGG_NOD_LIKE_RECEPTOR_SIGNALING_PATHWAY | 62 | -0.56 | -1.93 | 0 | 0.003 | 0.01 | 5447 | tags=45%, list=10%, signal=50% |
| 7 | KEGG_NATURAL_KILLER_CELL_MEDIATED_CYTOTOXICITY | 132 | -0.5 | -1.9 | 0 | 0.003 | 0.011 | 4598 | tags=30%, list=8%, signal=32% |
| 8 | KEGG_SPHINGOLIPID_METABOLISM | 39 | -0.59 | -1.85 | 0 | 0.007 | 0.029 | 3555 | tags=38%, list=6%, signal=41% |
| 9 | KEGG_CYTOKINE_CYTOKINE_RECEPTOR_INTERACTION | 263 | -0.44 | -1.83 | 0 | 0.008 | 0.035 | 7324 | tags=32%, list=13%, signal=36% |
| 10 | KEGG_PROTEASOME | 46 | -0.56 | -1.83 | 0 | 0.007 | 0.035 | 3319 | tags=37%, list=6%, signal=39% |
| 11 | KEGG_TOLL_LIKE_RECEPTOR_SIGNALING_PATHWAY | 102 | -0.49 | -1.82 | 0 | 0.008 | 0.042 | 4917 | tags=33%, list=9%, signal=37% |
| 12 | KEGG_OOCYTE_MEIOSIS | 112 | -0.48 | -1.79 | 0 | 0.01 | 0.06 | 4432 | tags=32%, list=8%, signal=35% |
| 13 | KEGG_ALZHEIMERS_DISEASE | 165 | -0.45 | -1.77 | 0 | 0.012 | 0.076 | 5842 | tags=33%, list=11%, signal=37% |
| 14 | KEGG_STARCH_AND_SUCROSE_METABOLISM | 52 | -0.54 | -1.75 | 0 | 0.014 | 0.095 | 8965 | tags=38%, list=16%, signal=46% |
| 15 | KEGG_ASCORBATE_AND_ALDARATE_METABOLISM | 25 | -0.61 | -1.74 | 0.005 | 0.015 | 0.108 | 8965 | tags=48%, list=16%, signal=57% |
| 16 | KEGG_AMYOTROPHIC_LATERAL_SCLEROSIS_ALS | 52 | -0.51 | -1.68 | 0 | 0.025 | 0.19 | 4321 | tags=31%, list=8%, signal=33% |
| 17 | KEGG_CITRATE_CYCLE_TCA_CYCLE | 31 | -0.57 | -1.66 | 0.003 | 0.03 | 0.233 | 4459 | tags=42%, list=8%, signal=46% |
| 18 | KEGG_PROPANOATE_METABOLISM | 33 | -0.55 | -1.65 | 0.012 | 0.03 | 0.243 | 4510 | tags=45%, list=8%, signal=49% |
| 19 | KEGG_PARKINSONS_DISEASE | 128 | -0.43 | -1.65 | 0 | 0.03 | 0.255 | 5813 | tags=34%, list=11%, signal=37% |
| 20 | KEGG_LONG_TERM_POTENTIATION | 70 | -0.48 | -1.64 | 0 | 0.029 | 0.263 | 4196 | tags=30%, list=8%, signal=32% |
| 21 | KEGG_P53_SIGNALING_PATHWAY | 68 | -0.47 | -1.64 | 0.003 | 0.029 | 0.266 | 3912 | tags=31%, list=7%, signal=33% |
| 22 | KEGG_UBIQUITIN_MEDIATED_PROTEOLYSIS | 134 | -0.43 | -1.63 | 0 | 0.029 | 0.28 | 4184 | tags=33%, list=8%, signal=35% |
| 23 | KEGG_RETINOL_METABOLISM | 64 | -0.48 | -1.63 | 0 | 0.028 | 0.287 | 10601 | tags=45%, list=19%, signal=56% |
| 24 | KEGG_DRUG_METABOLISM_CYTOCHROME_P450 | 71 | -0.46 | -1.6 | 0.003 | 0.037 | 0.378 | 9110 | tags=41%, list=17%, signal=49% |
| 25 | KEGG_INTESTINAL_IMMUNE_NETWORK_FOR_IGA_PRODUCTION | 45 | -0.5 | -1.6 | 0.008 | 0.036 | 0.381 | 10571 | tags=40%, list=19%, signal=49% |
| 26 | KEGG_NITROGEN_METABOLISM | 23 | -0.57 | -1.57 | 0.017 | 0.046 | 0.469 | 5066 | tags=39%, list=9%, signal=43% |
| 27 | KEGG_CHEMOKINE_SIGNALING_PATHWAY | 188 | -0.4 | -1.57 | 0 | 0.044 | 0.469 | 5702 | tags=29%, list=10%, signal=33% |
| 28 | KEGG_GRAFT_VERSUS_HOST_DISEASE | 37 | -0.51 | -1.56 | 0.008 | 0.046 | 0.492 | 4425 | tags=30%, list=8%, signal=32% |
| 29 | KEGG_T_CELL_RECEPTOR_SIGNALING_PATHWAY | 108 | -0.42 | -1.56 | 0.003 | 0.045 | 0.496 | 4917 | tags=34%, list=9%, signal=38% |
| 30 | KEGG_PEROXISOME | 78 | -0.44 | -1.56 | 0 | 0.045 | 0.511 | 6340 | tags=42%, list=11%, signal=48% |
| 31 | KEGG_OXIDATIVE_PHOSPHORYLATION | 131 | -0.41 | -1.56 | 0.003 | 0.043 | 0.512 | 5813 | tags=31%, list=11%, signal=34% |
| 32 | KEGG_LEISHMANIA_INFECTION | 69 | -0.44 | -1.54 | 0.006 | 0.047 | 0.552 | 4617 | tags=29%, list=8%, signal=32% |
| 33 | KEGG_O_GLYCAN_BIOSYNTHESIS | 30 | -0.54 | -1.54 | 0.028 | 0.047 | 0.563 | 6457 | tags=43%, list=12%, signal=49% |
| 34 | KEGG_ONE_CARBON_POOL_BY_FOLATE | 17 | -0.61 | -1.52 | 0.031 | 0.052 | 0.604 | 3985 | tags=59%, list=7%, signal=63% |
| 35 | KEGG_FRUCTOSE_AND_MANNOSE_METABOLISM | 33 | -0.5 | -1.52 | 0.024 | 0.054 | 0.63 | 4252 | tags=36%, list=8%, signal=39% |
| 36 | KEGG_GLYCOLYSIS_GLUCONEOGENESIS | 62 | -0.44 | -1.49 | 0.005 | 0.065 | 0.714 | 4001 | tags=29%, list=7%, signal=31% |
| 37 | KEGG_CYTOSOLIC_DNA_SENSING_PATHWAY | 55 | -0.44 | -1.49 | 0.011 | 0.064 | 0.719 | 3241 | tags=25%, list=6%, signal=27% |
| 38 | KEGG_RIG_I_LIKE_RECEPTOR_SIGNALING_PATHWAY | 71 | -0.42 | -1.49 | 0.023 | 0.063 | 0.723 | 4932 | tags=28%, list=9%, signal=31% |
| 39 | KEGG_GALACTOSE_METABOLISM | 26 | -0.54 | -1.49 | 0.034 | 0.063 | 0.731 | 2658 | tags=27%, list=5%, signal=28% |
| 40 | KEGG_HUNTINGTONS_DISEASE | 180 | -0.37 | -1.49 | 0.003 | 0.062 | 0.734 | 5919 | tags=31%, list=11%, signal=34% |
| 41 | KEGG_JAK_STAT_SIGNALING_PATHWAY | 155 | -0.38 | -1.48 | 0 | 0.062 | 0.743 | 7324 | tags=29%, list=13%, signal=33% |
| 42 | KEGG_TYPE_I_DIABETES_MELLITUS | 41 | -0.47 | -1.48 | 0.047 | 0.065 | 0.764 | 4136 | tags=24%, list=7%, signal=26% |
| 43 | KEGG_EPITHELIAL_CELL_SIGNALING_IN_HELICOBACTER_PYLORI_INFECTION | 68 | -0.42 | -1.47 | 0.014 | 0.064 | 0.766 | 6281 | tags=32%, list=11%, signal=36% |
| 44 | KEGG_HEMATOPOIETIC_CELL_LINEAGE | 85 | -0.41 | -1.47 | 0.003 | 0.065 | 0.779 | 9309 | tags=33%, list=17%, signal=40% |
| 45 | KEGG_AMINOACYL_TRNA_BIOSYNTHESIS | 41 | -0.47 | -1.46 | 0.02 | 0.065 | 0.784 | 6581 | tags=39%, list=12%, signal=44% |
| 46 | KEGG_ETHER_LIPID_METABOLISM | 33 | -0.48 | -1.46 | 0.046 | 0.067 | 0.804 | 5903 | tags=36%, list=11%, signal=41% |
| 47 | KEGG_BASAL_TRANSCRIPTION_FACTORS | 35 | -0.47 | -1.44 | 0.035 | 0.073 | 0.835 | 5298 | tags=40%, list=10%, signal=44% |
| 48 | KEGG_PENTOSE_AND_GLUCURONATE_INTERCONVERSIONS | 28 | -0.5 | -1.43 | 0.047 | 0.077 | 0.855 | 8965 | tags=36%, list=16%, signal=43% |
| 49 | KEGG_N_GLYCAN_BIOSYNTHESIS | 46 | -0.45 | -1.43 | 0.039 | 0.077 | 0.863 | 3008 | tags=28%, list=5%, signal=30% |
| 50 | KEGG_B_CELL_RECEPTOR_SIGNALING_PATHWAY | 75 | -0.4 | -1.42 | 0.019 | 0.081 | 0.89 | 4598 | tags=31%, list=8%, signal=33% |
| 51 | KEGG_CELL_CYCLE | 123 | -0.37 | -1.42 | 0.006 | 0.086 | 0.913 | 5545 | tags=33%, list=10%, signal=36% |
| 52 | KEGG_CYSTEINE_AND_METHIONINE_METABOLISM | 34 | -0.48 | -1.41 | 0.042 | 0.086 | 0.917 | 6356 | tags=41%, list=12%, signal=47% |
| 53 | KEGG_BUTANOATE_METABOLISM | 34 | -0.48 | -1.41 | 0.043 | 0.086 | 0.923 | 5170 | tags=35%, list=9%, signal=39% |
| 55 | KEGG_PURINE_METABOLISM | 158 | -0.36 | -1.39 | 0.007 | 0.092 | 0.943 | 4648 | tags=25%, list=8%, signal=28% |
| 56 | KEGG_NEUROTROPHIN_SIGNALING_PATHWAY | 126 | -0.37 | -1.39 | 0.007 | 0.092 | 0.944 | 5215 | tags=29%, list=9%, signal=32% |
| 57 | KEGG_METABOLISM_OF_XENOBIOTICS_BY_CYTOCHROME_P450 | 69 | -0.4 | -1.39 | 0.029 | 0.09 | 0.944 | 8965 | tags=36%, list=16%, signal=43% |
| 63 | KEGG_LONG_TERM_DEPRESSION | 70 | -0.39 | -1.36 | 0.042 | 0.104 | 0.973 | 4196 | tags=24%, list=8%, signal=26% |
| 64 | KEGG_COLORECTAL_CANCER | 61 | -0.4 | -1.36 | 0.037 | 0.103 | 0.973 | 4917 | tags=33%, list=9%, signal=36% |
| 65 | KEGG_MAPK_SIGNALING_PATHWAY | 266 | -0.33 | -1.35 | 0.005 | 0.103 | 0.976 | 4968 | tags=24%, list=9%, signal=26% |
| 67 | KEGG_LYSOSOME | 121 | -0.35 | -1.35 | 0.016 | 0.103 | 0.977 | 5820 | tags=31%, list=11%, signal=35% |
| 68 | KEGG_PROGESTERONE_MEDIATED_OOCYTE_MATURATION | 85 | -0.38 | -1.34 | 0.024 | 0.107 | 0.981 | 5240 | tags=28%, list=9%, signal=31% |
| 71 | KEGG_FC_EPSILON_RI_SIGNALING_PATHWAY | 79 | -0.37 | -1.32 | 0.041 | 0.116 | 0.99 | 5907 | tags=33%, list=11%, signal=37% |
| 74 | KEGG_TGF_BETA_SIGNALING_PATHWAY | 84 | -0.36 | -1.3 | 0.046 | 0.13 | 0.997 | 3185 | tags=23%, list=6%, signal=24% |
| 75 | KEGG_PROSTATE_CANCER | 89 | -0.36 | -1.28 | 0.043 | 0.144 | 0.999 | 4598 | tags=28%, list=8%, signal=31% |

**Table S9**. The transcription factors (TF) related to cancer.

| Transcription factor |
| --- |
| ADNP |
| AFF4 |
| AR |
| ARID3A |
| ARNT |
| ARNTL |
| ASCL1 |
| ASH2L |
| ATF1 |
| ATF2 |
| ATF3 |
| ATF4 |
| ATF7 |
| BACH1 |
| BACH2 |
| BATF |
| BCL11A |
| BCL3 |
| BCL6 |
| BDP1 |
| BHLHE40 |
| BMI1 |
| BRCA1 |
| BRD1 |
| BRD2 |
| BRD3 |
| BRD4 |
| BRF1 |
| BRF2 |
| C17orf96 |
| CBFB |
| CBX2 |
| CBX3 |
| CBX5 |
| CBX7 |
| CBX8 |
| CDK2 |
| CDK7 |
| CDK8 |
| CDK9 |
| CDX2 |
| CEBPA |
| CEBPB |
| CENPA |
| CHD1 |
| CHD2 |
| CHD7 |
| CHD8 |
| CIITA |
| CPSF3L |
| CREBBP |
| CTNNB1 |
| CUX1 |
| DNMT1 |
| DNMT3A |
| DYRK1A |
| E2F1 |
| E2F3 |
| E2F4 |
| E2F6 |
| E2F7 |
| EBF1 |
| EED |
| EGR1 |
| EGR2 |
| EHF |
| EHMT2 |
| ELF1 |
| ELF5 |
| ELK1 |
| ELK4 |
| ELL2 |
| EMX1 |
| EOMES |
| EP400 |
| EPAS1 |
| EPO |
| ERCC6 |
| ERG |
| ESR1 |
| ESRRA |
| ETS1 |
| ETV1 |
| EZH1 |
| EZH2 |
| FLI1 |
| FOS |
| FOSL1 |
| FOSL2 |
| FOXA1 |
| FOXA2 |
| FOXK1 |
| FOXM1 |
| FOXO1 |
| FOXO3 |
| FOXP1 |
| FOXP2 |
| FOXP3 |
| GABPA |
| GATA2 |
| GATA3 |
| GATA4 |
| GATA6 |
| GATAD1 |
| GREB1 |
| GRHL2 |
| GTF2B |
| GTF2F1 |
| GTF2I |
| H2AFX |
| HCFC1 |
| HDAC1 |
| HDAC2 |
| HDAC3 |
| HDAC6 |
| HEY1 |
| HIF1A |
| HIRA |
| HNF1B |
| HNF4A |
| HNF4G |
| HOXA9 |
| HOXB13 |
| HOXB7 |
| HOXC11 |
| HOXC9 |
| HSF1 |
| HSF2 |
| IKZF1 |
| IRF1 |
| IRF3 |
| IRF4 |
| IRF5 |
| JARID2 |
| JMJD1C |
| JMJD6 |
| JUN |
| JUNB |
| JUND |
| KAT2B |
| KAT5 |
| KDM1A |
| KDM2B |
| KDM3A |
| KDM4C |
| KDM5A |
| KDM5B |
| KDM5C |
| KDM6B |
| KLF11 |
| KLF4 |
| KLF5 |
| LEF1 |
| LHX2 |
| LIN9 |
| LMNA |
| LMNB1 |
| LMO2 |
| LYL1 |
| MAF |
| MAFF |
| MAFK |
| MAX |
| MAZ |
| MBD2 |
| MBD3 |
| MECP2 |
| MED12 |
| MEF2A |
| MEF2B |
| MEF2C |
| MEIS1 |
| MITF |
| MXI1 |
| MYB |
| MYBL2 |
| MYC |
| MYH11 |
| NANOG |
| NCAPG |
| NCOR2 |
| NFATC1 |
| NFE2 |
| NFIC |
| NFYA |
| NFYB |
| NIPBL |
| NOTCH1 |
| NR1H2 |
| NR2C2 |
| NR2F1 |
| NR2F2 |
| NR3C1 |
| NR4A1 |
| NR5A2 |
| NRF1 |
| OGT |
| PAF1 |
| PAX3 |
| PAX5 |
| PAX6 |
| PBX1 |
| PBX3 |
| PDX1 |
| PHF8 |
| PIAS1 |
| PML |
| POLR2B |
| POLR3A |
| POLR3D |
| POLR3G |
| POU2F1 |
| POU5F1 |
| PPARD |
| PPARG |
| PRDM1 |
| PRKDC |
| RAG1 |
| RARA |
| RARG |
| RB1 |
| RBBP5 |
| RBL2 |
| RBP2 |
| RBPJ |
| RCOR1 |
| RELA |
| RFX2 |
| RFX5 |
| RING1 |
| RNF2 |
| RUNX1 |
| RUNX1T1 |
| RXRA |
| RXRG |
| RYBP |
| SALL4 |
| SAP30 |
| SCML2 |
| SETDB1 |
| SF1 |
| SFMBT1 |
| SFPQ |
| SIN3A |
| SIRT6 |
| SIX5 |
| SMAD1 |
| SMAD2 |
| SMAD3 |
| SMAD4 |
| SMARCA4 |
| SMARCB1 |
| SMARCC1 |
| SMARCC2 |
| SMC1A |
| SMC3 |
| SNAI2 |
| SNAPC2 |
| SNAPC4 |
| SOX17 |
| SOX2 |
| SOX4 |
| SOX9 |
| SP2 |
| SPDEF |
| SPIB |
| SRC |
| SREBF1 |
| SREBF2 |
| SRF |
| SSRP1 |
| STAT1 |
| STAT2 |
| STAT3 |
| STAT4 |
| STAT5A |
| STAT5B |
| STAT6 |
| SUMO1 |
| SUMO2 |
| SUPT5H |
| TAF1 |
| TAL1 |
| TAT |
| TBL1XR1 |
| TBP |
| TCF12 |
| TCF21 |
| TCF7 |
| TCF7L1 |
| TCF7L2 |
| TEAD1 |
| TEAD4 |
| TERF1 |
| TERF2 |
| TET2 |
| TFAP2A |
| TFAP2C |
| THAP11 |
| TP53 |
| TP63 |
| TP73 |
| TRIM28 |
| TTF2 |
| UBTF |
| USF1 |
| USF2 |
| VDR |
| VEZF1 |
| WDR5 |
| WHSC1 |
| WWTR1 |
| XBP1 |
| XRN2 |
| YAP1 |
| YY1 |
| ZBTB17 |
| ZBTB33 |

**Table S10**. The differentially expressed Transcription factors between colorectal cancer and normal.

| gene | conMean | treatMean | logFC | pValue | fdr |
| --- | --- | --- | --- | --- | --- |
| ADNP | 11.84598 | 28.327685 | 1.2578151 | 2.65E-122 | 2.38E-120 |
| AR | 1.1604311 | 0.5638704 | -1.041225 | 1.64E-77 | 7.99E-77 |
| ARID3A | 2.1854065 | 9.9386267 | 2.1851449 | 1.37E-104 | 2.02E-103 |
| ARNTL | 7.9071669 | 2.2385084 | -1.820623 | 1.36E-110 | 3.09E-109 |
| ATF7 | 7.1194763 | 2.8381362 | -1.326827 | 7.25E-94 | 6.34E-93 |
| BATF | 1.889878 | 7.7141712 | 2.029218 | 3.24E-72 | 1.39E-71 |
| BCL6 | 44.321712 | 4.7453395 | -3.22343 | 1.81E-91 | 1.39E-90 |
| BHLHE40 | 32.442949 | 72.172136 | 1.153537 | 4.83E-57 | 1.57E-56 |
| BRCA1 | 1.7896377 | 5.034486 | 1.492177 | 2.64E-118 | 1.29E-116 |
| BRF1 | 8.6399789 | 2.1773399 | -1.988461 | 2.82E-88 | 1.92E-87 |
| CBFB | 6.4893564 | 23.440517 | 1.8528571 | 1.25E-127 | 4.28E-125 |
| CBX2 | 0.7357603 | 4.5818386 | 2.6386189 | 3.49E-116 | 1.32E-114 |
| CBX3 | 34.227221 | 73.683413 | 1.1061957 | 8.93E-90 | 6.44E-89 |
| CBX5 | 4.9031734 | 11.457555 | 1.2245116 | 1.21E-97 | 1.24E-96 |
| CBX7 | 32.886598 | 3.6872744 | -3.156873 | 2.31E-124 | 3.22E-122 |
| CBX8 | 1.5454682 | 6.017982 | 1.9612358 | 1.39E-120 | 9.58E-119 |
| CDK7 | 7.7453398 | 17.377325 | 1.1658056 | 1.33E-109 | 2.75E-108 |
| CDX2 | 32.950962 | 79.204996 | 1.2652709 | 1.19E-46 | 3.27E-46 |
| CEBPA | 9.977544 | 23.657381 | 1.2455337 | 9.11E-53 | 2.75E-52 |
| CENPA | 1.3279019 | 8.8068151 | 2.7294718 | 2.31E-125 | 4.15E-123 |
| CHD2 | 18.350155 | 7.2351132 | -1.342705 | 1.25E-92 | 1.01E-91 |
| CTNNB1 | 55.389395 | 132.73541 | 1.2608715 | 4.36E-115 | 1.48E-113 |
| E2F1 | 2.2466302 | 19.191811 | 3.0946562 | 9.64E-124 | 1.18E-121 |
| E2F3 | 2.5549486 | 8.4863956 | 1.7318576 | 5.60E-129 | 3.89E-126 |
| E2F7 | 0.5565212 | 2.3900454 | 2.1025293 | 9.27E-127 | 2.52E-124 |
| EBF1 | 2.6317868 | 1.0200824 | -1.367357 | 9.83E-84 | 5.71E-83 |
| EHF | 14.208385 | 47.206838 | 1.7322533 | 1.44E-64 | 5.32E-64 |
| ELF1 | 15.726008 | 33.421711 | 1.0876331 | 5.59E-80 | 2.91E-79 |
| ELF5 | 0.3859791 | 1.4720319 | 1.9312143 | 2.32E-33 | 5.05E-33 |
| ELK1 | 7.3490496 | 20.209468 | 1.4594018 | 3.81E-121 | 2.85E-119 |
| EZH1 | 18.545952 | 5.8823523 | -1.656639 | 3.03E-108 | 5.63E-107 |
| EZH2 | 3.1153852 | 12.01029 | 1.9467885 | 2.41E-121 | 1.86E-119 |
| FLI1 | 4.3769154 | 1.7543351 | -1.31899 | 1.01E-104 | 1.51E-103 |
| FOSL1 | 3.8885822 | 13.534077 | 1.7992804 | 4.41E-72 | 1.88E-71 |
| FOXA2 | 2.7528795 | 24.673099 | 3.1639256 | 3.37E-119 | 1.85E-117 |
| FOXM1 | 3.2311558 | 19.175211 | 2.5691202 | 3.31E-120 | 2.12E-118 |
| FOXP2 | 6.5890673 | 0.7807391 | -3.077162 | 2.63E-111 | 6.36E-110 |
| FOXP3 | 0.633855 | 2.5833412 | 2.0270135 | 8.24E-110 | 1.74E-108 |
| GATA6 | 5.8983909 | 16.163658 | 1.4543603 | 9.72E-87 | 6.25E-86 |
| GRHL2 | 2.7540549 | 13.723877 | 2.3170589 | 7.93E-122 | 6.53E-120 |
| GTF2I | 34.084592 | 5.378872 | -2.663744 | 2.03E-87 | 1.34E-86 |
| H2AFX | 12.284123 | 70.045413 | 2.5114958 | 5.67E-118 | 2.67E-116 |
| HDAC6 | 34.561004 | 7.1650731 | -2.270092 | 2.02E-99 | 2.26E-98 |
| HNF4A | 19.356784 | 60.071029 | 1.6338301 | 4.62E-65 | 1.72E-64 |
| HNF4G | 5.1505161 | 11.811035 | 1.1973465 | 1.76E-54 | 5.47E-54 |
| HOXB7 | 10.000034 | 30.249384 | 1.5969009 | 5.03E-76 | 2.36E-75 |
| HOXC11 | 0.40867 | 1.1661456 | 1.5127395 | 5.30E-15 | 7.08E-15 |
| JARID2 | 3.0516016 | 6.5313996 | 1.0978256 | 1.21E-102 | 1.61E-101 |
| JMJD1C | 15.172549 | 7.5634051 | -1.004356 | 2.51E-84 | 1.49E-83 |
| JMJD6 | 20.453197 | 7.5456232 | -1.438614 | 1.70E-80 | 8.99E-80 |
| KAT2B | 6.8911492 | 3.278484 | -1.071716 | 5.36E-86 | 3.36E-85 |
| KAT5 | 24.007346 | 11.897011 | -1.012877 | 3.45E-105 | 5.28E-104 |
| KDM4C | 6.919193 | 3.2296372 | -1.099232 | 1.58E-95 | 1.48E-94 |
| KLF5 | 45.771277 | 162.74241 | 1.8300758 | 1.42E-75 | 6.58E-75 |
| LEF1 | 1.7134037 | 4.5829819 | 1.4194215 | 1.70E-70 | 7.06E-70 |
| LIN9 | 1.3026734 | 3.7702284 | 1.5331765 | 3.16E-111 | 7.60E-110 |
| LMNB1 | 7.113073 | 38.193667 | 2.4247886 | 6.62E-118 | 3.09E-116 |
| MAFK | 30.401755 | 13.990805 | -1.119676 | 2.47E-55 | 7.79E-55 |
| MAZ | 66.948941 | 32.356668 | -1.048998 | 1.53E-86 | 9.81E-86 |
| MECP2 | 8.6984441 | 3.7994611 | -1.194963 | 3.18E-91 | 2.43E-90 |
| MEF2B | 1.7192778 | 0.6949009 | -1.306924 | 6.50E-95 | 5.95E-94 |
| MEF2C | 5.5627652 | 1.8370423 | -1.598417 | 1.99E-106 | 3.30E-105 |
| MEIS1 | 34.500742 | 1.3901139 | -4.633352 | 2.19E-118 | 1.08E-116 |
| MITF | 6.3748896 | 1.6575869 | -1.943316 | 5.81E-68 | 2.29E-67 |
| MXI1 | 26.491136 | 12.573412 | -1.075134 | 1.54E-78 | 7.71E-78 |
| MYB | 6.4795747 | 21.452823 | 1.7271965 | 2.80E-83 | 1.61E-82 |
| MYBL2 | 3.0821994 | 58.560399 | 4.2478933 | 2.84E-127 | 8.96E-125 |
| MYC | 29.9242 | 153.00542 | 2.3541982 | 4.40E-106 | 7.12E-105 |
| MYH11 | 1362.4922 | 24.321916 | -5.807847 | 1.11E-111 | 2.78E-110 |
| NCAPG | 1.2978159 | 7.270568 | 2.4859823 | 1.80E-122 | 1.68E-120 |
| NFATC1 | 4.442233 | 1.4907931 | -1.575205 | 2.03E-81 | 1.10E-80 |
| NFIC | 26.176253 | 10.626949 | -1.300531 | 1.46E-45 | 3.93E-45 |
| NFYA | 4.6725466 | 13.584612 | 1.5396925 | 3.30E-121 | 2.50E-119 |
| NFYB | 22.095946 | 8.7941638 | -1.329163 | 2.62E-48 | 7.36E-48 |
| NIPBL | 15.35555 | 7.6218259 | -1.010552 | 8.77E-89 | 6.09E-88 |
| NR3C1 | 5.563877 | 2.63442 | -1.078605 | 8.00E-73 | 3.48E-72 |
| NR4A1 | 111.63338 | 21.195989 | -2.396905 | 1.53E-44 | 4.04E-44 |
| PBX1 | 19.067952 | 3.0832803 | -2.628612 | 5.55E-112 | 1.43E-110 |
| PBX3 | 17.128969 | 4.2153557 | -2.022712 | 4.04E-53 | 1.23E-52 |
| PDX1 | 0.4708821 | 11.870522 | 4.6558736 | 3.40E-128 | 1.52E-125 |
| PML | 20.389217 | 10.07219 | -1.017429 | 7.06E-56 | 2.25E-55 |
| POLR3A | 2.9424383 | 5.9364442 | 1.0125869 | 1.39E-116 | 5.52E-115 |
| POLR3G | 1.055792 | 2.5170753 | 1.2534227 | 3.83E-75 | 1.75E-74 |
| RBBP5 | 3.3053629 | 9.3579828 | 1.5013889 | 3.94E-112 | 1.03E-110 |
| RBP2 | 2.8495952 | 9.4186849 | 1.7247686 | 2.33E-63 | 8.41E-63 |
| RFX2 | 8.4459417 | 1.5949027 | -2.40479 | 6.06E-90 | 4.40E-89 |
| RNF2 | 3.171519 | 7.2442984 | 1.191672 | 3.82E-103 | 5.19E-102 |
| RUNX1 | 3.8347799 | 7.8293575 | 1.0297502 | 1.20E-79 | 6.20E-79 |
| RUNX1T1 | 3.0209434 | 0.7015168 | -2.10645 | 1.82E-102 | 2.40E-101 |
| RXRG | 1.9463698 | 0.4326854 | -2.169396 | 1.35E-125 | 2.66E-123 |
| SALL4 | 0.4876513 | 1.7339611 | 1.8301498 | 1.56E-108 | 2.96E-107 |
| SIX5 | 11.056039 | 3.5149325 | -1.653266 | 8.04E-83 | 4.53E-82 |
| SMAD2 | 17.073682 | 3.0653414 | -2.477655 | 1.26E-108 | 2.42E-107 |
| SMAD4 | 14.522308 | 5.5121231 | -1.397591 | 8.75E-110 | 1.84E-108 |
| SMARCB1 | 22.295576 | 48.46084 | 1.120062 | 2.07E-105 | 3.22E-104 |
| SMARCC1 | 11.62292 | 34.820409 | 1.5829606 | 3.30E-125 | 5.51E-123 |
| SMC1A | 9.2631168 | 21.909107 | 1.2419611 | 1.17E-99 | 1.33E-98 |
| SMC3 | 9.9084692 | 22.186101 | 1.1629221 | 1.07E-93 | 9.22E-93 |
| SNAPC4 | 3.4361592 | 7.344211 | 1.0958106 | 1.54E-103 | 2.15E-102 |
| SOX17 | 3.5842221 | 1.3821336 | -1.374763 | 1.62E-45 | 4.35E-45 |
| SOX4 | 4.2415735 | 40.957337 | 3.2714504 | 1.45E-130 | 2.18E-127 |
| SOX9 | 6.388224 | 97.591843 | 3.9332738 | 6.22E-131 | 1.40E-127 |
| SPIB | 7.597398 | 1.1969507 | -2.666142 | 2.49E-14 | 3.29E-14 |
| STAT1 | 17.630705 | 56.622304 | 1.6832803 | 1.71E-93 | 1.45E-92 |
| STAT2 | 37.016515 | 16.105151 | -1.200647 | 7.79E-85 | 4.71E-84 |
| TAL1 | 1.5069152 | 0.6774207 | -1.153474 | 5.89E-105 | 8.92E-104 |
| TCF21 | 7.1304243 | 1.5110328 | -2.238453 | 3.12E-121 | 2.37E-119 |
| TCF7 | 2.1375037 | 8.074191 | 1.9173908 | 3.28E-103 | 4.49E-102 |
| TCF7L1 | 9.9493392 | 2.112833 | -2.235422 | 6.77E-102 | 8.62E-101 |
| TEAD4 | 4.3361404 | 18.840254 | 2.119335 | 9.85E-108 | 1.76E-106 |
| TP53 | 10.53076 | 40.391722 | 1.9394501 | 4.81E-83 | 2.74E-82 |
| TP73 | 0.4765205 | 1.560677 | 1.7115619 | 2.35E-97 | 2.38E-96 |
| USF2 | 57.196033 | 25.544153 | -1.162922 | 4.50E-95 | 4.14E-94 |
| WDR5 | 10.702635 | 26.87519 | 1.3283089 | 2.06E-120 | 1.38E-118 |
| WWTR1 | 28.803151 | 5.0464375 | -2.512889 | 5.39E-72 | 2.30E-71 |
| XBP1 | 42.482292 | 0.3756643 | -6.821274 | 1.04E-93 | 8.95E-93 |
| XRN2 | 15.065399 | 49.502029 | 1.7162488 | 2.70E-120 | 1.78E-118 |
| ZBTB17 | 15.563225 | 6.9645948 | -1.16003 | 9.87E-92 | 7.71E-91 |
| ZBTB33 | 2.4718046 | 10.694464 | 2.1132275 | 2.23E-123 | 2.52E-121 |

**Table S11**. The correlation between the differentially expressed transcription factors and hub genes.

| TF | immuneGene | cor | pvalue | Regulation |
| --- | --- | --- | --- | --- |
| ADNP | SEPHS1 | 0.377479855 | 1.39E-08 | postive |
| BCL6 | PODXL | 0.405105606 | 8.88E-10 | postive |
| BHLHE40 | PODXL | 0.430509964 | 5.63E-11 | postive |
| E2F7 | PGM2 | 0.42939708 | 6.38E-11 | postive |
| FOXM1 | RHNO1 | 0.920068661 | 2.04E-87 | postive |
| HDAC6 | PGM2 | -0.359927992 | 6.99E-08 | negative |
| HNF4A | SEPHS1 | 0.350992453 | 1.54E-07 | postive |
| HNF4G | PGM2 | 0.426665331 | 8.67E-11 | postive |
| LIN9 | PGM2 | 0.39557865 | 2.36E-09 | postive |
| MITF | PODXL | 0.534229831 | 4.83E-17 | postive |
| NCAPG | PGM2 | 0.567550296 | 1.79E-19 | postive |
| NFATC1 | PODXL | 0.392232999 | 3.30E-09 | postive |
| NR3C1 | PODXL | 0.499735372 | 8.57E-15 | postive |
| PBX3 | PODXL | 0.407654523 | 6.80E-10 | postive |
| POLR3G | PGM2 | 0.429377559 | 6.40E-11 | postive |
| RUNX1 | PODXL | 0.412158085 | 4.23E-10 | postive |
| SMAD2 | PGM2 | 0.431701984 | 4.92E-11 | postive |
| STAT1 | SCD | 0.369120818 | 3.03E-08 | postive |
| STAT2 | PODXL | 0.352261435 | 1.38E-07 | postive |
| TEAD4 | RHNO1 | 0.876679582 | 1.15E-68 | postive |
| USF2 | PGM2 | -0.412838717 | 3.93E-10 | negative |
| WDR5 | SEPHS1 | 0.423668336 | 1.21E-10 | postive |
| WWTR1 | PODXL | 0.408438755 | 6.27E-10 | postive |
| ZBTB17 | PGM2 | -0.443783893 | 1.21E-11 | negative |

Table S12. The possibilities of potential drug-repurposing based on the hub genes.

| Hub gene | BindingDB Reactant_set_id | UniProt (SwissProt) Entry Name of Target Chain |
| --- | --- | --- |
| PODXL | 50267803 | CNTN1_HUMAN |
| SEPHS1 | 50115363 | TXNRD1_HUMAN |
| SEPHS2 | 50115369 | TXNRD1_HUMAN |
| SEPHS3 | 50115392 | TXNRD1_HUMAN |
| SEPHS4 | 50115403 | TXNRD1_HUMAN |
| SEPHS5 | 50115410 | TXNRD1_HUMAN |
| SEPHS6 | 50115416 | TXNRD1_HUMAN |
| SEPHS7 | 50115423 | TXNRD1_HUMAN |
| SEPHS8 | 50115445 | TXNRD1_HUMAN |
| SEPHS9 | 50115447 | TXNRD1_HUMAN |
| SEPHS10 | 50115450 | TXNRD1_HUMAN |
| SEPHS11 | 50115451 | TXNRD1_HUMAN |
| SEPHS12 | 50508544 | TXNRD1_HUMAN |
| SEPHS13 | 50570318 | TXNRD1_HUMAN |
| RHNO1 | 50503615 | BRCA1_HUMAN |
| RHNO2 | 50503616 | BRCA1_HUMAN |
| RHNO3 | 50503617 | BRCA1_HUMAN |
| RHNO4 | 50643780 | BRCA1_HUMAN |
| RHNO5 | 50643781 | BRCA1_HUMAN |
| RHNO6 | 50643782 | BRCA1_HUMAN |
| RHNO7 | 50643783 | BRCA1_HUMAN |
| RHNO8 | 50643784 | BRCA1_HUMAN |
| RHNO9 | 50643785 | BRCA1_HUMAN |
| RHNO10 | 50643786 | BRCA1_HUMAN |
| RHNO11 | 50643787 | BRCA1_HUMAN |
| RHNO12 | 50643788 | BRCA1_HUMAN |
| RHNO13 | 50643789 | BRCA1_HUMAN |
| RHNO14 | 50643790 | BRCA1_HUMAN |
| RHNO15 | 50643791 | BRCA1_HUMAN |
| RHNO16 | 50643792 | BRCA1_HUMAN |
| RHNO17 | 50643793 | BRCA1_HUMAN |
| RHNO18 | 50643794 | BRCA1_HUMAN |
| RHNO19 | 50643795 | BRCA1_HUMAN |
| RHNO20 | 50643796 | BRCA1_HUMAN |
| RHNO21 | 50643797 | BRCA1_HUMAN |
| RHNO22 | 50643798 | BRCA1_HUMAN |
| RHNO23 | 50643799 | BRCA1_HUMAN |
| RHNO24 | 50643800 | BRCA1_HUMAN |
| RHNO25 | 50643801 | BRCA1_HUMAN |
| RHNO26 | 50927401 | BRCA1_HUMAN |
| RHNO27 | 50927402 | BRCA1_HUMAN |
| RHNO28 | 50927403 | BRCA1_HUMAN |
| RHNO29 | 50927404 | BRCA1_HUMAN |
| RHNO30 | 50927405 | BRCA1_HUMAN |
| RHNO31 | 50927406 | BRCA1_HUMAN |
| RHNO32 | 50927407 | BRCA1_HUMAN |
| RHNO33 | 50927408 | BRCA1_HUMAN |
| RHNO34 | 50927409 | BRCA1_HUMAN |
| RHNO35 | 50927410 | BRCA1_HUMAN |
| RHNO36 | 50927411 | BRCA1_HUMAN |
| RHNO37 | 50927412 | BRCA1_HUMAN |
| RHNO38 | 50927413 | BRCA1_HUMAN |
| RHNO39 | 50927414 | BRCA1_HUMAN |
| RHNO40 | 50927415 | BRCA1_HUMAN |
| SCD | 50536786 | FADS2_HUMAN |
| SCD | 50563478 | FADS2_HUMAN |
| SCD | 50713869 | FADS2_HUMAN |
| SCD | 50717444 | FADS2_HUMAN |
| SCD | 50957861 | FADS2_HUMAN |
| SCD | 50957862 | FADS2_HUMAN |
| SCD | 50957863 | FADS2_HUMAN |
| SCD | 50957864 | FADS2_HUMAN |
| SCD | 50957865 | FADS2_HUMAN |
| PGM2 | 50408507 | TKT_HUMAN |
| PGM3 | 50408508 | TKT_HUMAN |
| PGM4 | 50408509 | TKT_HUMAN |
| PGM5 | 50408510 | TKT_HUMAN |
| PGM6 | 50408511 | TKT_HUMAN |
| PGM7 | 50408512 | TKT_HUMAN |
| PGM8 | 50408513 | TKT_HUMAN |
| PGM9 | 50408514 | TKT_HUMAN |
| PGM10 | 50408515 | TKT_HUMAN |
| PGM11 | 50408516 | TKT_HUMAN |
| PGM12 | 50408517 | TKT_HUMAN |
| PGM13 | 50408518 | TKT_HUMAN |
| PGM14 | 50408519 | TKT_HUMAN |
| PGM15 | 50408520 | TKT_HUMAN |
| PGM16 | 50408521 | TKT_HUMAN |
| PGM17 | 50408522 | TKT_HUMAN |
| PGM18 | 50408523 | TKT_HUMAN |
| PGM19 | 50431478 | TKT_HUMAN |
| PGM20 | 50860105 | TKT_HUMAN |
| PGM21 | 50860106 | TKT_HUMAN |
| PGM22 | 50860107 | TKT_HUMAN |
| PGM23 | 50860108 | TKT_HUMAN |
| PGM24 | 50860109 | TKT_HUMAN |
| PGM25 | 50860110 | TKT_HUMAN |
| PGM26 | 50860111 | TKT_HUMAN |
| PGM27 | 50860112 | TKT_HUMAN |
| PGM28 | 50860113 | TKT_HUMAN |
| PGM29 | 50860114 | TKT_HUMAN |
| PGM30 | 50860115 | TKT_HUMAN |
| PGM31 | 50860116 | TKT_HUMAN |
| PGM32 | 50860117 | TKT_HUMAN |
| PGM33 | 50860118 | TKT_HUMAN |
| PGM34 | 50860119 | TKT_HUMAN |
| PGM35 | 50860120 | TKT_HUMAN |
| PGM36 | 50860121 | TKT_HUMAN |
| PGM37 | 50860122 | TKT_HUMAN |
| PGM38 | 50860123 | TKT_HUMAN |
| PGM39 | 50860124 | TKT_HUMAN |
| PGM40 | 50860125 | TKT_HUMAN |
| PGM41 | 50860126 | TKT_HUMAN |
| PGM42 | 50860127 | TKT_HUMAN |
| PGM43 | 50860128 | TKT_HUMAN |
| PGM44 | 50860129 | TKT_HUMAN |
| PGM45 | 50860130 | TKT_HUMAN |
| PGM46 | 50860131 | TKT_HUMAN |
| PGM47 | 50860132 | TKT_HUMAN |
| PGM48 | 50860133 | TKT_HUMAN |
| PGM49 | 50860134 | TKT_HUMAN |
| PGM50 | 50860135 | TKT_HUMAN |
| PGM51 | 50860136 | TKT_HUMAN |
| PGM52 | 50860137 | TKT_HUMAN |
| PGM53 | 50860138 | TKT_HUMAN |
| PGM54 | 50860139 | TKT_HUMAN |
| PGM55 | 50860140 | TKT_HUMAN |
| PGM56 | 50860141 | TKT_HUMAN |
| PGM57 | 50860142 | TKT_HUMAN |
| PGM58 | 50860143 | TKT_HUMAN |
| PGM59 | 50860144 | TKT_HUMAN |
| PGM60 | 50860145 | TKT_HUMAN |
| PGM61 | 50860146 | TKT_HUMAN |
| PGM62 | 50860147 | TKT_HUMAN |
| PGM63 | 50860148 | TKT_HUMAN |
| PGM64 | 50860149 | TKT_HUMAN |
| PGM65 | 50860150 | TKT_HUMAN |
| PGM66 | 50860151 | TKT_HUMAN |
| PGM67 | 50860152 | TKT_HUMAN |
| PGM68 | 50860153 | TKT_HUMAN |
| PGM69 | 50860154 | TKT_HUMAN |
| PGM70 | 50860155 | TKT_HUMAN |
| PGM71 | 50860156 | TKT_HUMAN |
| PGM72 | 50860157 | TKT_HUMAN |
| PGM73 | 50860158 | TKT_HUMAN |
| PGM74 | 50860159 | TKT_HUMAN |
| PGM75 | 50860160 | TKT_HUMAN |
| PGM76 | 50860480 | TKT_HUMAN |
| PGM77 | 50860481 | TKT_HUMAN |
| PGM78 | 50860482 | TKT_HUMAN |
| PGM79 | 50860483 | TKT_HUMAN |
| PGM80 | 50860484 | TKT_HUMAN |
| PGM81 | 50860485 | TKT_HUMAN |
| PGM82 | 50860486 | TKT_HUMAN |
| PGM83 | 50860487 | TKT_HUMAN |
| PGM84 | 50860488 | TKT_HUMAN |
| PGM85 | 50860489 | TKT_HUMAN |
| PGM86 | 50860490 | TKT_HUMAN |
| PGM87 | 50860491 | TKT_HUMAN |
| PGM88 | 50860492 | TKT_HUMAN |
| PGM89 | 50860493 | TKT_HUMAN |
| PGM90 | 50860494 | TKT_HUMAN |
| PGM91 | 50860495 | TKT_HUMAN |
| PGM92 | 50860496 | TKT_HUMAN |
| PGM93 | 50860497 | TKT_HUMAN |
| PGM94 | 50860498 | TKT_HUMAN |
| PGM95 | 50860499 | TKT_HUMAN |
| PGM96 | 50860500 | TKT_HUMAN |
| PGM97 | 50860501 | TKT_HUMAN |
| PGM98 | 50860502 | TKT_HUMAN |
| PGM99 | 50860503 | TKT_HUMAN |
| PGM100 | 50860504 | TKT_HUMAN |
| PGM101 | 50860505 | TKT_HUMAN |
| PGM102 | 50860506 | TKT_HUMAN |
| PGM103 | 50860507 | TKT_HUMAN |
| PGM104 | 50860508 | TKT_HUMAN |
| PGM105 | 50860509 | TKT_HUMAN |
| PGM106 | 50860510 | TKT_HUMAN |

**Table S13**. The results of structure-based virtual screening for PGM2.

| Title | Docking score |
| --- | --- |
| ZINC000000020240 | -9.524 |
| ZINC000000004785 | -9.181 |
| ZINC000000006157 | -9.053 |
| ZINC000000011012 | -8.991 |
| ZINC000000896634 | -8.853 |
| ZINC000000000903 | -8.791 |
| ZINC000036294079 | -8.748 |
| ZINC000036294079 | -8.746 |
| ZINC000000002191 | -8.721 |
| ZINC000000000128 | -8.672 |
| ZINC000000020243 | -8.646 |
| ZINC000000025958 | -8.614 |
| ZINC000000105216 | -8.586 |
| ZINC000001690324 | -8.568 |
| ZINC000001690324 | -8.568 |
| ZINC000000056646 | -8.556 |
| ZINC000000005560 | -8.552 |
| ZINC000000008667 | -8.526 |
| ZINC000000896731 | -8.51 |
| ZINC000000896731 | -8.509 |
| ZINC000001530764 | -8.506 |
| ZINC000000007782 | -8.504 |
| ZINC000000002279 | -8.501 |
| ZINC000001999487 | -8.498 |
| ZINC000005162311 | -8.49 |
| ZINC000006467621 | -8.434 |
| ZINC000013537284 | -8.433 |
| ZINC000034220093 | -8.391 |
| ZINC000035342789 | -8.355 |
| ZINC000005733652 | -8.327 |
| ZINC000005733652 | -8.327 |
| ZINC000005733652 | -8.301 |
| ZINC000005733652 | -8.301 |
| ZINC000002570817 | -8.3 |
| ZINC000000020221 | -8.298 |
| ZINC000000006427 | -8.282 |
| ZINC000000057534 | -8.275 |
| ZINC000000004321 | -8.272 |
| ZINC000001853550 | -8.259 |
| ZINC000000000850 | -8.256 |
| ZINC000000000850 | -8.255 |
| ZINC000003812974 | -8.204 |
| ZINC000013512456 | -8.165 |
| ZINC000003831139 | -8.113 |
| ZINC000000057435 | -8.09 |
| ZINC000000002272 | -8.078 |
| ZINC000000001145 | -8.032 |
| ZINC000000001145 | -8.021 |
| ZINC000003604264 | -8.02 |
| ZINC000000006251 | -7.977 |
| ZINC000000006251 | -7.977 |
| ZINC000000968233 | -7.976 |
| ZINC000001530636 | -7.949 |
| ZINC000008466459 | -7.929 |
| ZINC000000136138 | -7.927 |
| ZINC000000136138 | -7.925 |
| ZINC000000001408 | -7.909 |
| ZINC000000018635 | -7.904 |
| ZINC000000020241 | -7.881 |
| ZINC000003979899 | -7.867 |
| ZINC000000035804 | -7.823 |
| ZINC000000014864 | -7.812 |
| ZINC000003775644 | -7.8 |
| ZINC000000057464 | -7.797 |
| ZINC000000083315 | -7.788 |
| ZINC000019875504 | -7.787 |
| ZINC000001530948 | -7.761 |
| ZINC000019875504 | -7.758 |
| ZINC000006733300 | -7.75 |
| ZINC000043207851 | -7.732 |
| ZINC000000896703 | -7.729 |
| ZINC000000000655 | -7.707 |
| ZINC000001530741 | -7.699 |
| ZINC000018203737 | -7.686 |
| ZINC000018203737 | -7.686 |
| ZINC000000014257 | -7.677 |
| ZINC000013512456 | -7.674 |
| ZINC000000074836 | -7.646 |
| ZINC000000006156 | -7.632 |
| ZINC000004640636 | -7.625 |
| ZINC000000020259 | -7.622 |
| ZINC000001997125 | -7.613 |
| ZINC000000001758 | -7.598 |
| ZINC000000001382 | -7.576 |
| ZINC000000000061 | -7.571 |
| ZINC000000097996 | -7.567 |
| ZINC000000085733 | -7.563 |
| ZINC000000120286 | -7.563 |
| ZINC000001530637 | -7.561 |
| ZINC000003875368 | -7.559 |
| ZINC000003875368 | -7.559 |
| ZINC000000403609 | -7.553 |
| ZINC000000056556 | -7.546 |
| ZINC000000608101 | -7.544 |
| ZINC000001530638 | -7.532 |
| ZINC000003818808 | -7.53 |
| ZINC000000057532 | -7.528 |
| ZINC000013537284 | -7.528 |
| ZINC000012414057 | -7.518 |
| ZINC000000897385 | -7.512 |
| ZINC000000057206 | -7.496 |
| ZINC000000402909 | -7.494 |
| ZINC000000057206 | -7.489 |
| ZINC000003874498 | -7.458 |
| ZINC000001530803 | -7.434 |
| ZINC000003629271 | -7.431 |
| ZINC000000057533 | -7.429 |
| ZINC000022010375 | -7.429 |
| ZINC000022010375 | -7.429 |
| ZINC000022010375 | -7.429 |
| ZINC000000000271 | -7.416 |
| ZINC000000113398 | -7.408 |
| ZINC000003842753 | -7.408 |
| ZINC000003842753 | -7.408 |
| ZINC000009302239 | -7.378 |
| ZINC000004097225 | -7.377 |
| ZINC000012466082 | -7.374 |
| ZINC000252678020 | -7.374 |
| ZINC000000001958 | -7.373 |
| ZINC000006021033 | -7.371 |
| ZINC000006036847 | -7.368 |
| ZINC000012503076 | -7.362 |
| ZINC000000012346 | -7.36 |
| ZINC000004392649 | -7.357 |
| ZINC000018203737 | -7.352 |
| ZINC000018203737 | -7.352 |
| ZINC000034051848 | -7.333 |
| ZINC000000001331 | -7.332 |
| ZINC000001530725 | -7.323 |
| ZINC000003775644 | -7.322 |
| ZINC000022010387 | -7.321 |
| ZINC000022010387 | -7.321 |
| ZINC000022010387 | -7.321 |
| ZINC000000000323 | -7.311 |
| ZINC000000057255 | -7.311 |
| ZINC000100299039 | -7.288 |
| ZINC000003871541 | -7.284 |
| ZINC000000057278 | -7.276 |
| ZINC000003813010 | -7.275 |
| ZINC000003813010 | -7.275 |
| ZINC000000896819 | -7.275 |
| ZINC000003872605 | -7.273 |
| ZINC000000596881 | -7.272 |
| ZINC000003830990 | -7.272 |
| ZINC000003872605 | -7.271 |
| ZINC000035999642 | -7.267 |
| ZINC000017146904 | -7.266 |
| ZINC000034051848 | -7.263 |
| ZINC000035999642 | -7.262 |
| ZINC000003806413 | -7.261 |
| ZINC000001530636 | -7.26 |
| ZINC000019702309 | -7.249 |
| ZINC000001997127 | -7.249 |
| ZINC000000001644 | -7.245 |
| ZINC000022116608 | -7.241 |
| ZINC000003806413 | -7.238 |
| ZINC000012466082 | -7.238 |
| ZINC000252678020 | -7.238 |
| ZINC000000968263 | -7.238 |
| ZINC000003812869 | -7.237 |
| ZINC000003812869 | -7.227 |
| ZINC000001482094 | -7.214 |
| ZINC000003812865 | -7.207 |
| ZINC000000057464 | -7.207 |
| ZINC000000004009 | -7.204 |
| ZINC000000121541 | -7.189 |
| ZINC000000005895 | -7.188 |
| ZINC000001530947 | -7.185 |
| ZINC000018203737 | -7.181 |
| ZINC000018203737 | -7.181 |
| ZINC000012360535 | -7.179 |
| ZINC000004632106 | -7.179 |
| ZINC000004632106 | -7.178 |
| ZINC000000001145 | -7.167 |
| ZINC000000968330 | -7.164 |
| ZINC000022010379 | -7.163 |
| ZINC000022010379 | -7.163 |
| ZINC000022010379 | -7.163 |
| ZINC000000001145 | -7.162 |
| ZINC000000001464 | -7.15 |
| ZINC000000034157 | -7.148 |
| ZINC000000002647 | -7.148 |
| ZINC000000049153 | -7.146 |
| ZINC000002522648 | -7.142 |
| ZINC000004632106 | -7.141 |
| ZINC000000643055 | -7.141 |
| ZINC000022010382 | -7.132 |
| ZINC000022010382 | -7.132 |
| ZINC000022010382 | -7.132 |
| ZINC000000000431 | -7.123 |
| ZINC000000000575 | -7.115 |
| ZINC000000006310 | -7.11 |
| ZINC000000113382 | -7.109 |
| ZINC000000002101 | -7.108 |
| ZINC000000014360 | -7.105 |
| ZINC000003779042 | -7.104 |
| ZINC000000895199 | -7.095 |
| ZINC000006021043 | -7.094 |
| ZINC000000113382 | -7.094 |
| ZINC000000967597 | -7.091 |
| ZINC000003798734 | -7.079 |
| ZINC000000119717 | -7.071 |
| ZINC000000004840 | -7.063 |
| ZINC000012360535 | -7.062 |
| ZINC000003960338 | -7.056 |
| ZINC000000006481 | -7.054 |
| ZINC000004632106 | -7.052 |
| ZINC000000001931 | -7.043 |
| ZINC000000001931 | -7.043 |
| ZINC000000968305 | -7.038 |
| ZINC000000968305 | -7.038 |
| ZINC000008403947 | -7.037 |
| ZINC000019168887 | -7.029 |
| ZINC000000014007 | -7.022 |
| ZINC000000001735 | -7.003 |
| ZINC000003802690 | -7.003 |
| ZINC000000001655 | -7.002 |
| ZINC000000113428 | -6.998 |
| ZINC000000020231 | -6.985 |
| ZINC000009224016 | -6.984 |
| ZINC000009224016 | -6.984 |
| ZINC000000007673 | -6.982 |
| ZINC000100009280 | -6.976 |
| ZINC000100009280 | -6.976 |
| ZINC000016929327 | -6.973 |
| ZINC000000002216 | -6.97 |
| ZINC000000002216 | -6.97 |
| ZINC000000056427 | -6.97 |
| ZINC000003781664 | -6.948 |
| ZINC000021303210 | -6.945 |
| ZINC000003940470 | -6.94 |
| ZINC000001690604 | -6.934 |
| ZINC000000608101 | -6.931 |
| ZINC000000643055 | -6.93 |
| ZINC000018279854 | -6.929 |
| ZINC000017146904 | -6.927 |
| ZINC000001842633 | -6.92 |
| ZINC000100014475 | -6.915 |
| ZINC000002169830 | -6.914 |
| ZINC000000018087 | -6.911 |
| ZINC000100009278 | -6.874 |
| ZINC000100009278 | -6.874 |
| ZINC000001530977 | -6.874 |
| ZINC000001530977 | -6.874 |
| ZINC000001530977 | -6.874 |
| ZINC000000001148 | -6.867 |
| ZINC000035024346 | -6.866 |
| ZINC000019594599 | -6.859 |
| ZINC000019594599 | -6.859 |
| ZINC000019594599 | -6.859 |
| ZINC000008855117 | -6.856 |
| ZINC000000000693 | -6.854 |
| ZINC000008855117 | -6.852 |
| ZINC000001530776 | -6.849 |
| ZINC000000001984 | -6.847 |
| ZINC000000001728 | -6.843 |
| ZINC000004640636 | -6.842 |
| ZINC000008855117 | -6.837 |
| ZINC000008855117 | -6.837 |
| ZINC000001481910 | -6.836 |
| ZINC000003872055 | -6.833 |
| ZINC000001530817 | -6.833 |
| ZINC000001530817 | -6.833 |
| ZINC000001035331 | -6.83 |
| ZINC000000020255 | -6.828 |
| ZINC000000020245 | -6.827 |
| ZINC000003952881 | -6.824 |
| ZINC000000001132 | -6.819 |
| ZINC000000000931 | -6.807 |
| ZINC000000000931 | -6.807 |
| ZINC000000518554 | -6.804 |
| ZINC000000033882 | -6.802 |
| ZINC000000003876 | -6.79 |
| ZINC000000002043 | -6.785 |
| ZINC000000002043 | -6.785 |
| ZINC000000897291 | -6.784 |
| ZINC000000002055 | -6.78 |
| ZINC000000002055 | -6.78 |
| ZINC000003872520 | -6.779 |
| ZINC000000388081 | -6.771 |
| ZINC000000388081 | -6.77 |
| ZINC000000388081 | -6.768 |
| ZINC000000001899 | -6.768 |
| ZINC000084757007 | -6.767 |
| ZINC000000002273 | -6.76 |
| ZINC000000002041 | -6.759 |
| ZINC000000005423 | -6.755 |
| ZINC000012661824 | -6.754 |
| ZINC000021303210 | -6.74 |
| ZINC000000057001 | -6.735 |
| ZINC000013597823 | -6.731 |
| ZINC000001543475 | -6.73 |
| ZINC000001543475 | -6.73 |
| ZINC000001543475 | -6.73 |
| ZINC000002015035 | -6.728 |
| ZINC000002015035 | -6.728 |
| ZINC000013597823 | -6.728 |
| ZINC000002548959 | -6.727 |
| ZINC000001530654 | -6.721 |
| ZINC000001530654 | -6.721 |
| ZINC000000000373 | -6.712 |
| ZINC000000020228 | -6.709 |
| ZINC000000266964 | -6.709 |
| ZINC000000020228 | -6.707 |
| ZINC000000897385 | -6.702 |
| ZINC000000403010 | -6.701 |
| ZINC000000968303 | -6.695 |
| ZINC000000403010 | -6.69 |
| ZINC000013129998 | -6.682 |
| ZINC000000057624 | -6.676 |
| ZINC000000001979 | -6.67 |
| ZINC000001481833 | -6.668 |
| ZINC000000015515 | -6.665 |
| ZINC000000002281 | -6.665 |
| ZINC000000013156 | -6.665 |
| ZINC000000000507 | -6.664 |
| ZINC000000001011 | -6.66 |
| ZINC000000000507 | -6.653 |
| ZINC000000039089 | -6.647 |
| ZINC000001530652 | -6.644 |
| ZINC000001530652 | -6.644 |
| ZINC000001482184 | -6.642 |
| ZINC000018043251 | -6.641 |
| ZINC000003812944 | -6.64 |
| ZINC000000113404 | -6.639 |
| ZINC000000113426 | -6.637 |
| ZINC000034781704 | -6.635 |
| ZINC000000000215 | -6.631 |
| ZINC000000000215 | -6.631 |
| ZINC000000057513 | -6.628 |
| ZINC000000057513 | -6.628 |
| ZINC000000000746 | -6.625 |
| ZINC000001482049 | -6.625 |
| ZINC000001482049 | -6.625 |
| ZINC000004228257 | -6.624 |
| ZINC000000897258 | -6.624 |
| ZINC000000105196 | -6.623 |
| ZINC000000897258 | -6.617 |
| ZINC000007997897 | -6.616 |
| ZINC000007997897 | -6.616 |
| ZINC000007997897 | -6.616 |
| ZINC000000002159 | -6.607 |
| ZINC000003861768 | -6.601 |
| ZINC000004228257 | -6.595 |
| ZINC000000896569 | -6.591 |
| ZINC000003830569 | -6.59 |
| ZINC000004095696 | -6.58 |
| ZINC000004475353 | -6.575 |
| ZINC000000020230 | -6.574 |
| ZINC000003872738 | -6.574 |
| ZINC000003795098 | -6.571 |
| ZINC000000003642 | -6.57 |
| ZINC000000391812 | -6.561 |
| ZINC000000391812 | -6.561 |
| ZINC000000391812 | -6.561 |
| ZINC000000020783 | -6.561 |
| ZINC000004216238 | -6.559 |
| ZINC000000057341 | -6.552 |
| ZINC000000596881 | -6.55 |
| ZINC000013597823 | -6.544 |
| ZINC000013597823 | -6.544 |
| ZINC000000895302 | -6.537 |
| ZINC000000001084 | -6.535 |
| ZINC000000896709 | -6.527 |
| ZINC000000004778 | -6.516 |
| ZINC000000508068 | -6.514 |
| ZINC000000607971 | -6.502 |
| ZINC000000057313 | -6.502 |
| ZINC000022002218 | -6.499 |
| ZINC000022002218 | -6.499 |
| ZINC000000137884 | -6.496 |
| ZINC000013585233 | -6.484 |
| ZINC000095452610 | -6.484 |
| ZINC000001530599 | -6.48 |
| ZINC000000403079 | -6.477 |
| ZINC000000403079 | -6.472 |
| ZINC000008214651 | -6.47 |
| ZINC000002847375 | -6.456 |
| ZINC000002847375 | -6.456 |
| ZINC000001639567 | -6.453 |
| ZINC000000113355 | -6.446 |
| ZINC000022002214 | -6.446 |
| ZINC000022002214 | -6.446 |
| ZINC000013550868 | -6.445 |
| ZINC000001530569 | -6.442 |
| ZINC000000001341 | -6.435 |
| ZINC000000001341 | -6.433 |
| ZINC000051133897 | -6.432 |
| ZINC000004258316 | -6.432 |
| ZINC000000004076 | -6.42 |
| ZINC000004228258 | -6.411 |
| ZINC000004228258 | -6.411 |
| ZINC000004228258 | -6.41 |
| ZINC000019632633 | -6.405 |
| ZINC000019632633 | -6.405 |
| ZINC000019632633 | -6.405 |
| ZINC000003798064 | -6.404 |
| ZINC000003873296 | -6.395 |
| ZINC000001530570 | -6.391 |
| ZINC000000056652 | -6.39 |
| ZINC000022056030 | -6.386 |
| ZINC000022056030 | -6.386 |
| ZINC000022056030 | -6.386 |
| ZINC000000001281 | -6.373 |
| ZINC000000020257 | -6.372 |
| ZINC000000056568 | -6.368 |
| ZINC000001536779 | -6.368 |
| ZINC000018099446 | -6.362 |
| ZINC000100019007 | -6.362 |
| ZINC000000896711 | -6.359 |
| ZINC000100070937 | -6.359 |
| ZINC000100032379 | -6.357 |
| ZINC000100032379 | -6.357 |
| ZINC000003927870 | -6.351 |
| ZINC000019702309 | -6.348 |
| ZINC000000057340 | -6.33 |
| ZINC000000601250 | -6.32 |
| ZINC000001482164 | -6.319 |
| ZINC000000020237 | -6.319 |
| ZINC000000020237 | -6.319 |
| ZINC000001576892 | -6.31 |
| ZINC000000000469 | -6.305 |
| ZINC000000001795 | -6.303 |
| ZINC000003872277 | -6.299 |
| ZINC000019166991 | -6.288 |
| ZINC000007997568 | -6.286 |
| ZINC000007997568 | -6.286 |
| ZINC000000388462 | -6.284 |
| ZINC000084843283 | -6.266 |
| ZINC000000007601 | -6.264 |
| ZINC000000020231 | -6.263 |
| ZINC000000156395 | -6.261 |
| ZINC000000005878 | -6.261 |
| ZINC000013537284 | -6.256 |
| ZINC000000119344 | -6.251 |
| ZINC000000895360 | -6.248 |
| ZINC000000013156 | -6.241 |
| ZINC000000113446 | -6.237 |
| ZINC000000403011 | -6.223 |
| ZINC000000896666 | -6.21 |
| ZINC000003806262 | -6.206 |
| ZINC000003806262 | -6.206 |
| ZINC000000000053 | -6.206 |
| ZINC000001530938 | -6.205 |
| ZINC000001530938 | -6.205 |
| ZINC000000075008 | -6.204 |
| ZINC000000075008 | -6.204 |
| ZINC000000901736 | -6.201 |
| ZINC000001633887 | -6.198 |
| ZINC000005179119 | -6.182 |
| ZINC000003927870 | -6.18 |
| ZINC000003927870 | -6.18 |
| ZINC000000001267 | -6.18 |
| ZINC000013537284 | -6.18 |
| ZINC000002015928 | -6.166 |
| ZINC000000057147 | -6.163 |
| ZINC000008015016 | -6.149 |
| ZINC000008015016 | -6.147 |
| ZINC000008015016 | -6.147 |
| ZINC000000394284 | -6.144 |
| ZINC000000896918 | -6.133 |
| ZINC000000008492 | -6.132 |
| ZINC000000001505 | -6.13 |
| ZINC000000001798 | -6.123 |
| ZINC000000001798 | -6.123 |
| ZINC000000001798 | -6.123 |
| ZINC000001547851 | -6.116 |
| ZINC000100007011 | -6.11 |
| ZINC000000599734 | -6.103 |
| ZINC000003830813 | -6.095 |
| ZINC000003830813 | -6.095 |
| ZINC000000403011 | -6.084 |
| ZINC000000000905 | -6.074 |
| ZINC000000001554 | -6.073 |
| ZINC000002561203 | -6.068 |
| ZINC000003823492 | -6.066 |
| ZINC000000057062 | -6.066 |
| ZINC000033943508 | -6.057 |
| ZINC000001542002 | -6.048 |
| ZINC000012360535 | -6.042 |
| ZINC000003830986 | -6.037 |
| ZINC000003830813 | -6.032 |
| ZINC000087515509 | -6.028 |
| ZINC000000057146 | -6.025 |
| ZINC000000896484 | -6.019 |
| ZINC000000896484 | -6.019 |
| ZINC000000001706 | -6.017 |
| ZINC000003813042 | -6.016 |
| ZINC000100005670 | -6.007 |
| ZINC000095452610 | -6.002 |
| ZINC000000895154 | -6.001 |
| ZINC000000895154 | -5.998 |
| ZINC000000000922 | -5.986 |
| ZINC000002005550 | -5.985 |
| ZINC000022010649 | -5.975 |
| ZINC000000000565 | -5.975 |
| ZINC000000000565 | -5.975 |
| ZINC000003798247 | -5.961 |
| ZINC000001530599 | -5.959 |
| ZINC000001530756 | -5.95 |
| ZINC000000004166 | -5.94 |
| ZINC000000001411 | -5.938 |
| ZINC000000395010 | -5.925 |
| ZINC000000001792 | -5.918 |
| ZINC000002005550 | -5.91 |
| ZINC000003956788 | -5.907 |
| ZINC000000389149 | -5.898 |
| ZINC000002539827 | -5.888 |
| ZINC000006409735 | -5.886 |
| ZINC000006409735 | -5.886 |
| ZINC000002539827 | -5.883 |
| ZINC000000896463 | -5.877 |
| ZINC000001883067 | -5.875 |
| ZINC000004228257 | -5.862 |
| ZINC000001530555 | -5.841 |
| ZINC000013585233 | -5.837 |
| ZINC000013585233 | -5.837 |
| ZINC000001531009 | -5.829 |
| ZINC000001531009 | -5.829 |
| ZINC000001531009 | -5.826 |
| ZINC000000897244 | -5.821 |
| ZINC000000003911 | -5.814 |
| ZINC000034676245 | -5.813 |
| ZINC000034676245 | -5.813 |
| ZINC000000001688 | -5.805 |
| ZINC000000001688 | -5.802 |
| ZINC000013545634 | -5.797 |
| ZINC000013545634 | -5.792 |
| ZINC000012358719 | -5.787 |
| ZINC000000901791 | -5.786 |
| ZINC000004658290 | -5.782 |
| ZINC000001698306 | -5.777 |
| ZINC000001530863 | -5.776 |
| ZINC000001530716 | -5.776 |
| ZINC000000056653 | -5.772 |
| ZINC000000000353 | -5.769 |
| ZINC000003794711 | -5.766 |
| ZINC000003794711 | -5.766 |
| ZINC000003794711 | -5.766 |
| ZINC000000121480 | -5.765 |
| ZINC000000968345 | -5.76 |
| ZINC000000004413 | -5.756 |
| ZINC000000004413 | -5.756 |
| ZINC000000968345 | -5.74 |
| ZINC000000896663 | -5.733 |
| ZINC000000056645 | -5.727 |
| ZINC000006661227 | -5.723 |
| ZINC000006661227 | -5.723 |
| ZINC000006661227 | -5.723 |
| ZINC000001530816 | -5.716 |
| ZINC000000388081 | -5.716 |
| ZINC000000388081 | -5.716 |
| ZINC000000388081 | -5.712 |
| ZINC000000000456 | -5.711 |
| ZINC000000000456 | -5.711 |
| ZINC000000000456 | -5.711 |
| ZINC000006627681 | -5.711 |
| ZINC000001530816 | -5.705 |
| ZINC000000002005 | -5.703 |
| ZINC000000004319 | -5.697 |
| ZINC000000156792 | -5.693 |
| ZINC000000006226 | -5.691 |
| ZINC000008101109 | -5.684 |
| ZINC000008101109 | -5.684 |
| ZINC000008101109 | -5.684 |
| ZINC000000004319 | -5.675 |
| ZINC000000049154 | -5.673 |
| ZINC000100071256 | -5.663 |
| ZINC000000002009 | -5.661 |
| ZINC000004658290 | -5.659 |
| ZINC000006627681 | -5.655 |
| ZINC000006382803 | -5.651 |
| ZINC000003830891 | -5.651 |
| ZINC000002539827 | -5.647 |
| ZINC000001482197 | -5.646 |
| ZINC000003830999 | -5.638 |
| ZINC000000001681 | -5.637 |
| ZINC000000001681 | -5.637 |
| ZINC000004258316 | -5.612 |
| ZINC000004676424 | -5.611 |
| ZINC000006382803 | -5.608 |
| ZINC000000897322 | -5.589 |
| ZINC000096006009 | -5.577 |
| ZINC000003831474 | -5.575 |
| ZINC000006661227 | -5.57 |
| ZINC000003651680 | -5.568 |
| ZINC000006661227 | -5.566 |
| ZINC000006661227 | -5.566 |
| ZINC000000113410 | -5.562 |
| ZINC000000896523 | -5.561 |
| ZINC000003651680 | -5.56 |
| ZINC000000000882 | -5.55 |
| ZINC000000000882 | -5.549 |
| ZINC000096006009 | -5.548 |
| ZINC000000002176 | -5.548 |
| ZINC000003830999 | -5.547 |
| ZINC000000020251 | -5.522 |
| ZINC000000000242 | -5.502 |
| ZINC000000000242 | -5.502 |
| ZINC000001533877 | -5.501 |
| ZINC000001533877 | -5.501 |
| ZINC000001533877 | -5.501 |
| ZINC000001533877 | -5.501 |
| ZINC000000895154 | -5.499 |
| ZINC000000896698 | -5.497 |
| ZINC000000896698 | -5.497 |
| ZINC000000000711 | -5.493 |
| ZINC000007997952 | -5.488 |
| ZINC000007997952 | -5.488 |
| ZINC000003918138 | -5.486 |
| ZINC000019166988 | -5.461 |
| ZINC000000120319 | -5.461 |
| ZINC000000120319 | -5.461 |
| ZINC000003831475 | -5.454 |
| ZINC000012503151 | -5.451 |
| ZINC000253917094 | -5.451 |
| ZINC000003803652 | -5.449 |
| ZINC000000537957 | -5.446 |
| ZINC000053084692 | -5.445 |
| ZINC000001530806 | -5.445 |
| ZINC000053084692 | -5.445 |
| ZINC000001187543 | -5.444 |
| ZINC000001530568 | -5.438 |
| ZINC000001843047 | -5.435 |
| ZINC000003830339 | -5.433 |
| ZINC000003776875 | -5.416 |
| ZINC000001530599 | -5.41 |
| ZINC000001530599 | -5.409 |
| ZINC000013545636 | -5.395 |
| ZINC000000009342 | -5.374 |
| ZINC000000009342 | -5.374 |
| ZINC000013545636 | -5.374 |
| ZINC000013298313 | -5.373 |
| ZINC000003874950 | -5.373 |
| ZINC000003830347 | -5.369 |
| ZINC000003830347 | -5.369 |
| ZINC000005133329 | -5.365 |
| ZINC000000049154 | -5.359 |
| ZINC000001530567 | -5.351 |
| ZINC000000895099 | -5.344 |
| ZINC000005133378 | -5.343 |
| ZINC000000895457 | -5.338 |
| ZINC000000000490 | -5.333 |
| ZINC000003645145 | -5.333 |
| ZINC000001530737 | -5.33 |
| ZINC000001530737 | -5.33 |
| ZINC000001530736 | -5.33 |
| ZINC000001530736 | -5.33 |
| ZINC000000000122 | -5.328 |
| ZINC000000000122 | -5.328 |
| ZINC000000154964 | -5.308 |
| ZINC000000154964 | -5.308 |
| ZINC000000154964 | -5.308 |
| ZINC000000005152 | -5.305 |
| ZINC000003874950 | -5.298 |
| ZINC000100061056 | -5.296 |
| ZINC000038212689 | -5.294 |
| ZINC000038212689 | -5.287 |
| ZINC000000896546 | -5.286 |
| ZINC000000896546 | -5.286 |
| ZINC000013298313 | -5.279 |
| ZINC000000896543 | -5.276 |
| ZINC000000000882 | -5.273 |
| ZINC000000000882 | -5.27 |
| ZINC000002016258 | -5.267 |
| ZINC000004340269 | -5.235 |
| ZINC000004340269 | -5.235 |
| ZINC000012503156 | -5.232 |
| ZINC000006827695 | -5.231 |
| ZINC000006827695 | -5.231 |
| ZINC000006827695 | -5.231 |
| ZINC000001533877 | -5.23 |
| ZINC000001533877 | -5.23 |
| ZINC000003801919 | -5.225 |
| ZINC000003801919 | -5.225 |
| ZINC000000113442 | -5.223 |
| ZINC000003801919 | -5.199 |
| ZINC000000014037 | -5.196 |
| ZINC000000089763 | -5.19 |
| ZINC000000897002 | -5.189 |
| ZINC000000897002 | -5.189 |
| ZINC000000897002 | -5.189 |
| ZINC000000089763 | -5.188 |
| ZINC000000001115 | -5.185 |
| ZINC000100006770 | -5.179 |
| ZINC000001530710 | -5.173 |
| ZINC000003818726 | -5.137 |
| ZINC000001530600 | -5.126 |
| ZINC000001530600 | -5.122 |
| ZINC000001530600 | -5.122 |
| ZINC000000002028 | -5.121 |
| ZINC000001530713 | -5.111 |
| ZINC000004097392 | -5.102 |
| ZINC000001846431 | -5.096 |
| ZINC000004658552 | -5.083 |
| ZINC000003818726 | -5.079 |
| ZINC000001530810 | -5.076 |
| ZINC000002000707 | -5.065 |
| ZINC000008034121 | -5.061 |
| ZINC000011680943 | -5.058 |
| ZINC000001530912 | -5.051 |
| ZINC000001530912 | -5.051 |
| ZINC000001530912 | -5.051 |
| ZINC000011680943 | -5.048 |
| ZINC000001530713 | -5.047 |
| ZINC000001530427 | -5.047 |
| ZINC000001530427 | -5.047 |
| ZINC000001530427 | -5.047 |
| ZINC000001532517 | -5.044 |
| ZINC000003581355 | -5.043 |
| ZINC000001849548 | -5.035 |
| ZINC000000004949 | -4.997 |
| ZINC000003812862 | -4.987 |
| ZINC000003812862 | -4.987 |
| ZINC000001530805 | -4.977 |
| ZINC000100009383 | -4.968 |
| ZINC000000114124 | -4.966 |
| ZINC000003589203 | -4.965 |
| ZINC000003831477 | -4.956 |
| ZINC000001530929 | -4.949 |
| ZINC000001530929 | -4.949 |
| ZINC000001530572 | -4.944 |
| ZINC000002016257 | -4.907 |
| ZINC000001633889 | -4.893 |
| ZINC000001530701 | -4.882 |
| ZINC000001530703 | -4.867 |
| ZINC000004658603 | -4.859 |
| ZINC000004658603 | -4.859 |
| ZINC000001530641 | -4.853 |
| ZINC000017285872 | -4.844 |
| ZINC000003812862 | -4.843 |
| ZINC000000599985 | -4.83 |
| ZINC000004097426 | -4.827 |
| ZINC000004097426 | -4.827 |
| ZINC000004097426 | -4.827 |
| ZINC000000010164 | -4.826 |
| ZINC000000010164 | -4.826 |
| ZINC000053022902 | -4.795 |
| ZINC000053022902 | -4.795 |
| ZINC000001532529 | -4.772 |
| ZINC000004658553 | -4.765 |
| ZINC000000000607 | -4.758 |
| ZINC000003008621 | -4.753 |
| ZINC000000599985 | -4.728 |
| ZINC000001554588 | -4.697 |
| ZINC000004577910 | -4.687 |
| ZINC000002539702 | -4.671 |
| ZINC000003831051 | -4.666 |
| ZINC000000057512 | -4.665 |
| ZINC000003831050 | -4.649 |
| ZINC000000000471 | -4.625 |
| ZINC000000000494 | -4.607 |
| ZINC000000897288 | -4.605 |
| ZINC000000020244 | -4.595 |
| ZINC000000020244 | -4.595 |
| ZINC000001530728 | -4.592 |
| ZINC000001530728 | -4.592 |
| ZINC000008034120 | -4.591 |
| ZINC000003830314 | -4.58 |
| ZINC000001532526 | -4.574 |
| ZINC000019144216 | -4.566 |
| ZINC000019144216 | -4.566 |
| ZINC000018089317 | -4.565 |
| ZINC000000113415 | -4.562 |
| ZINC000000155531 | -4.554 |
| ZINC000000155531 | -4.554 |
| ZINC000000901555 | -4.552 |
| ZINC000003809490 | -4.541 |
| ZINC000000114127 | -4.537 |
| ZINC000034676245 | -4.536 |
| ZINC000003079342 | -4.5 |
| ZINC000001530575 | -4.499 |
| ZINC000008214514 | -4.484 |
| ZINC000000000905 | -4.481 |
| ZINC000003079340 | -4.477 |
| ZINC000001531008 | -4.462 |
| ZINC000002019953 | -4.405 |
| ZINC000002019953 | -4.39 |
| ZINC000000155905 | -4.37 |
| ZINC000006827693 | -4.369 |
| ZINC000006827693 | -4.369 |
| ZINC000001530703 | -4.366 |
| ZINC000000001982 | -4.362 |
| ZINC000012859773 | -4.353 |
| ZINC000003782550 | -4.341 |
| ZINC000000000083 | -4.334 |
| ZINC000003782550 | -4.329 |
| ZINC000003831551 | -4.324 |
| ZINC000001532728 | -4.301 |
| ZINC000001543873 | -4.251 |
| ZINC000001543873 | -4.251 |
| ZINC000001530862 | -4.243 |
| ZINC000005224188 | -4.205 |
| ZINC000003079337 | -4.187 |
| ZINC000008101126 | -4.161 |
| ZINC000019594594 | -4.15 |
| ZINC000019594594 | -4.15 |
| ZINC000001530717 | -4.145 |
| ZINC000000968255 | -4.115 |
| ZINC000003079336 | -4.104 |
| ZINC000003831040 | -4.1 |
| ZINC000001530940 | -4.097 |
| ZINC000001530940 | -4.097 |
| ZINC000000895042 | -4.094 |
| ZINC000052971887 | -4.076 |
| ZINC000052971887 | -4.074 |
| ZINC000000901552 | -4.043 |
| ZINC000001532522 | -4.01 |
| ZINC000000895103 | -3.988 |
| ZINC000000000257 | -3.954 |
| ZINC000000895032 | -3.893 |
| ZINC000001530718 | -3.87 |
| ZINC000002019954 | -3.79 |
| ZINC000002019954 | -3.777 |
| ZINC000018115268 | -3.774 |
| ZINC000018115268 | -3.774 |
| ZINC000001530283 | -3.739 |
| ZINC000004658562 | -3.716 |
| ZINC000001529425 | -3.669 |
| ZINC000001530811 | -3.667 |
| ZINC000001530811 | -3.667 |
| ZINC000000895081 | -3.641 |
| ZINC000000016154 | -3.641 |
| ZINC000019364225 | -3.581 |
| ZINC000019364225 | -3.581 |
| ZINC000000901061 | -3.512 |
| ZINC000001530762 | -3.485 |
| ZINC000001530762 | -3.485 |
| ZINC000003843378 | -3.396 |
| ZINC000004658560 | -3.353 |
| ZINC000001530951 | -3.318 |
| ZINC000001482113 | -3.255 |
| ZINC000013973998 | -3.222 |
| ZINC000001530703 | -3.154 |
| ZINC000001530703 | -3.15 |
| ZINC000004658557 | -3.108 |
| ZINC000000895034 | -2.99 |
| ZINC000000895034 | -2.989 |
| ZINC000003929508 | -2.985 |
| ZINC000000895318 | -2.941 |
| ZINC000008214625 | -2.929 |
| ZINC000100017856 | -2.709 |
| ZINC000100017856 | -2.709 |
| ZINC000008214573 | -2.683 |
| ZINC000000895316 | -2.656 |
| ZINC000100037020 | -2.632 |
| ZINC000100037020 | -2.632 |
| ZINC000100037020 | -2.632 |
| ZINC000001554392 | -2.615 |
| ZINC000001554392 | -2.613 |
| ZINC000000403618 | -2.59 |
| ZINC000001554010 | -2.585 |
| ZINC000003798750 | -2.558 |
| ZINC000018115268 | -2.53 |
| ZINC000018115268 | -2.53 |
| ZINC000001532179 | -2.512 |
| ZINC000001530950 | -2.466 |
| ZINC000000896695 | -2.452 |
| ZINC000000896695 | -2.447 |
| ZINC000001532805 | -2.402 |
| ZINC000000895048 | -2.306 |
| ZINC000006845860 | -2.266 |
| ZINC000003808779 | -2.159 |
| ZINC000000901159 | -2.127 |
| ZINC000001530820 | -2.079 |
| ZINC000001530303 | -1.712 |
| ZINC000019364219 | -1.681 |
| ZINC000019364219 | -1.673 |
| ZINC000019363537 | -1.447 |
| ZINC000019363537 | -1.44 |
| ZINC000017285869 | -1.4 |
| ZINC000003977777 | -1.205 |
| ZINC000019364242 | -1.125 |
| ZINC000019364242 | -1.125 |
| ZINC000001531036 | -1.039 |
| ZINC000008437287 | -0.282 |
| ZINC000001532525 | 0.051 |
| ZINC000000896409 | 1.336 |
| ZINC000000896409 | 1.336 |
| ZINC000001530635 | 2.286 |

**Table S14**. The results of structure-based virtual screening for PODXL.

| Title | Docking score |
| --- | --- |
| ZINC000003927870 | -8.092 |
| ZINC000003927870 | -8.092 |
| ZINC000003927870 | -8.086 |
| ZINC000027990463 | -7.285 |
| ZINC000001530974 | -7.216 |
| ZINC000001529323 | -7.193 |
| ZINC000022448097 | -7.083 |
| ZINC000022448097 | -7.083 |
| ZINC000022448097 | -7.083 |
| ZINC000003956788 | -7.048 |
| ZINC000000527386 | -7.032 |
| ZINC000002016037 | -6.96 |
| ZINC000003952881 | -6.921 |
| ZINC000008855117 | -6.916 |
| ZINC000005133378 | -6.906 |
| ZINC000009212428 | -6.899 |
| ZINC000008855117 | -6.858 |
| ZINC000009212428 | -6.818 |
| ZINC000009212428 | -6.817 |
| ZINC000003812865 | -6.812 |
| ZINC000100296832 | -6.807 |
| ZINC000100296832 | -6.807 |
| ZINC000100006770 | -6.791 |
| ZINC000012360535 | -6.77 |
| ZINC000000014864 | -6.75 |
| ZINC000002005305 | -6.73 |
| ZINC000001530973 | -6.722 |
| ZINC000013512456 | -6.702 |
| ZINC000100006264 | -6.697 |
| ZINC000000897385 | -6.68 |
| ZINC000000000856 | -6.679 |
| ZINC000000896731 | -6.671 |
| ZINC000000896731 | -6.663 |
| ZINC000009212428 | -6.654 |
| ZINC000009212428 | -6.652 |
| ZINC000029319828 | -6.628 |
| ZINC000001997127 | -6.627 |
| ZINC000011616925 | -6.627 |
| ZINC000001530922 | -6.62 |
| ZINC000001530922 | -6.62 |
| ZINC000001530922 | -6.62 |
| ZINC000003782818 | -6.616 |
| ZINC000003782818 | -6.616 |
| ZINC000031274852 | -6.608 |
| ZINC000031274852 | -6.608 |
| ZINC000003831490 | -6.597 |
| ZINC000008577218 | -6.582 |
| ZINC000000607971 | -6.57 |
| ZINC000003873921 | -6.55 |
| ZINC000019632917 | -6.546 |
| ZINC000000403011 | -6.544 |
| ZINC000013512456 | -6.527 |
| ZINC000003831490 | -6.522 |
| ZINC000000121541 | -6.521 |
| ZINC000000403011 | -6.508 |
| ZINC000001530636 | -6.508 |
| ZINC000000001341 | -6.482 |
| ZINC000000001341 | -6.482 |
| ZINC000000895360 | -6.481 |
| ZINC000003812862 | -6.476 |
| ZINC000003812862 | -6.476 |
| ZINC000014210876 | -6.475 |
| ZINC000003940470 | -6.474 |
| ZINC000003604264 | -6.472 |
| ZINC000003812862 | -6.467 |
| ZINC000049643479 | -6.461 |
| ZINC000014210876 | -6.458 |
| ZINC000084589076 | -6.417 |
| ZINC000000000850 | -6.409 |
| ZINC000003872566 | -6.406 |
| ZINC000000000850 | -6.403 |
| ZINC000000001735 | -6.402 |
| ZINC000000596881 | -6.402 |
| ZINC000018203737 | -6.4 |
| ZINC000018203737 | -6.4 |
| ZINC000000057624 | -6.395 |
| ZINC000000897085 | -6.391 |
| ZINC000000601250 | -6.389 |
| ZINC000002005305 | -6.363 |
| ZINC000003801919 | -6.363 |
| ZINC000003801919 | -6.363 |
| ZINC000003801919 | -6.36 |
| ZINC000000968303 | -6.354 |
| ZINC000000643055 | -6.35 |
| ZINC000003831139 | -6.34 |
| ZINC000000895154 | -6.294 |
| ZINC000018279854 | -6.287 |
| ZINC000003806262 | -6.283 |
| ZINC000003806262 | -6.283 |
| ZINC000003872520 | -6.275 |
| ZINC000003807804 | -6.269 |
| ZINC000000137884 | -6.267 |
| ZINC000000538065 | -6.265 |
| ZINC000000538065 | -6.265 |
| ZINC000002525885 | -6.26 |
| ZINC000002525885 | -6.26 |
| ZINC000004228258 | -6.258 |
| ZINC000019419017 | -6.252 |
| ZINC000000000853 | -6.249 |
| ZINC000100070937 | -6.233 |
| ZINC000000538658 | -6.227 |
| ZINC000000643138 | -6.226 |
| ZINC000000602128 | -6.223 |
| ZINC000003830813 | -6.218 |
| ZINC000003830813 | -6.218 |
| ZINC000003831586 | -6.218 |
| ZINC000001543475 | -6.211 |
| ZINC000001543475 | -6.211 |
| ZINC000001543475 | -6.211 |
| ZINC000003779042 | -6.205 |
| ZINC000003830813 | -6.203 |
| ZINC000003873295 | -6.192 |
| ZINC000000895154 | -6.184 |
| ZINC000000608101 | -6.182 |
| ZINC000009212427 | -6.173 |
| ZINC000001997125 | -6.171 |
| ZINC000013585233 | -6.149 |
| ZINC000001530947 | -6.144 |
| ZINC000001552908 | -6.142 |
| ZINC000003802690 | -6.135 |
| ZINC000000011012 | -6.126 |
| ZINC000000592419 | -6.109 |
| ZINC000004228258 | -6.108 |
| ZINC000004228258 | -6.108 |
| ZINC000000607986 | -6.106 |
| ZINC000000607986 | -6.105 |
| ZINC000002005305 | -6.101 |
| ZINC000000004009 | -6.098 |
| ZINC000001547851 | -6.095 |
| ZINC000002005305 | -6.095 |
| ZINC000009212427 | -6.092 |
| ZINC000001481815 | -6.09 |
| ZINC000001481815 | -6.09 |
| ZINC000003810860 | -6.088 |
| ZINC000000000905 | -6.087 |
| ZINC000001530764 | -6.083 |
| ZINC000000001145 | -6.07 |
| ZINC000000001145 | -6.068 |
| ZINC000002005305 | -6.064 |
| ZINC000000004840 | -6.064 |
| ZINC000085537017 | -6.064 |
| ZINC000085537017 | -6.061 |
| ZINC000085537017 | -6.061 |
| ZINC000022851765 | -6.055 |
| ZINC000022851765 | -6.055 |
| ZINC000022851765 | -6.055 |
| ZINC000000006427 | -6.052 |
| ZINC000000968274 | -6.045 |
| ZINC000000897089 | -6.04 |
| ZINC000008015016 | -6.04 |
| ZINC000000596881 | -6.036 |
| ZINC000001531009 | -6.035 |
| ZINC000001531009 | -6.034 |
| ZINC000001531009 | -6.034 |
| ZINC000000000271 | -6.034 |
| ZINC000000013156 | -6.022 |
| ZINC000028973446 | -6.022 |
| ZINC000001530948 | -6.018 |
| ZINC000004658290 | -6.018 |
| ZINC000019418959 | -6.015 |
| ZINC000019418959 | -6.015 |
| ZINC000019418959 | -6.015 |
| ZINC000001530974 | -6.014 |
| ZINC000003874185 | -6.013 |
| ZINC000002539827 | -6.006 |
| ZINC000002539827 | -6.003 |
| ZINC000000113404 | -6.001 |
| ZINC000001530636 | -5.995 |
| ZINC000003831477 | -5.994 |
| ZINC000009212427 | -5.993 |
| ZINC000000968301 | -5.993 |
| ZINC000009212427 | -5.992 |
| ZINC000003830449 | -5.99 |
| ZINC000000002055 | -5.988 |
| ZINC000000002055 | -5.988 |
| ZINC000000002041 | -5.984 |
| ZINC000035653009 | -5.982 |
| ZINC000008015016 | -5.981 |
| ZINC000008015016 | -5.981 |
| ZINC000000001084 | -5.978 |
| ZINC000000000431 | -5.976 |
| ZINC000002510358 | -5.975 |
| ZINC000003798064 | -5.973 |
| ZINC000002510358 | -5.969 |
| ZINC000000113382 | -5.967 |
| ZINC000000113382 | -5.965 |
| ZINC000001550477 | -5.96 |
| ZINC000000004321 | -5.954 |
| ZINC000004658290 | -5.949 |
| ZINC000019419017 | -5.948 |
| ZINC000019419017 | -5.948 |
| ZINC000000537805 | -5.945 |
| ZINC000002005305 | -5.939 |
| ZINC000000020230 | -5.939 |
| ZINC000000000575 | -5.938 |
| ZINC000002539827 | -5.933 |
| ZINC000000057147 | -5.932 |
| ZINC000000001958 | -5.926 |
| ZINC000002539702 | -5.926 |
| ZINC000003871541 | -5.926 |
| ZINC000004099200 | -5.921 |
| ZINC000000402830 | -5.907 |
| ZINC000000402830 | -5.907 |
| ZINC000012360535 | -5.905 |
| ZINC000013818943 | -5.903 |
| ZINC000000075126 | -5.901 |
| ZINC000005819214 | -5.9 |
| ZINC000013831130 | -5.9 |
| ZINC000013831130 | -5.898 |
| ZINC000009212427 | -5.896 |
| ZINC000000001850 | -5.894 |
| ZINC000004658553 | -5.881 |
| ZINC000019364229 | -5.879 |
| ZINC000019364229 | -5.879 |
| ZINC000019364229 | -5.879 |
| ZINC000084757007 | -5.879 |
| ZINC000003830990 | -5.877 |
| ZINC000002548959 | -5.875 |
| ZINC000000057254 | -5.872 |
| ZINC000000057254 | -5.872 |
| ZINC000001530741 | -5.871 |
| ZINC000006827695 | -5.87 |
| ZINC000006827695 | -5.87 |
| ZINC000006827695 | -5.87 |
| ZINC000000968336 | -5.866 |
| ZINC000000085733 | -5.859 |
| ZINC000004099200 | -5.855 |
| ZINC000019364230 | -5.855 |
| ZINC000019364230 | -5.855 |
| ZINC000019364230 | -5.855 |
| ZINC000003775644 | -5.85 |
| ZINC000038212689 | -5.85 |
| ZINC000038212689 | -5.848 |
| ZINC000003800475 | -5.847 |
| ZINC000001530788 | -5.845 |
| ZINC000000896543 | -5.839 |
| ZINC000000000096 | -5.836 |
| ZINC000000018635 | -5.828 |
| ZINC000008214418 | -5.825 |
| ZINC000009212427 | -5.824 |
| ZINC000003873296 | -5.823 |
| ZINC000000006157 | -5.822 |
| ZINC000002570895 | -5.817 |
| ZINC000000020245 | -5.807 |
| ZINC000100009383 | -5.803 |
| ZINC000053022902 | -5.803 |
| ZINC000053022902 | -5.803 |
| ZINC000000000469 | -5.8 |
| ZINC000013298313 | -5.799 |
| ZINC000000896546 | -5.796 |
| ZINC000000896546 | -5.795 |
| ZINC000001846431 | -5.795 |
| ZINC000003873371 | -5.792 |
| ZINC000003873371 | -5.792 |
| ZINC000008101109 | -5.782 |
| ZINC000008101109 | -5.782 |
| ZINC000008101109 | -5.782 |
| ZINC000003960338 | -5.776 |
| ZINC000000967597 | -5.774 |
| ZINC000004658552 | -5.773 |
| ZINC000000013156 | -5.772 |
| ZINC000006382803 | -5.771 |
| ZINC000000089763 | -5.77 |
| ZINC000070466416 | -5.769 |
| ZINC000000000882 | -5.766 |
| ZINC000000000882 | -5.766 |
| ZINC000000089763 | -5.763 |
| ZINC000000901736 | -5.76 |
| ZINC000000897291 | -5.759 |
| ZINC000035801098 | -5.759 |
| ZINC000035801098 | -5.755 |
| ZINC000000000128 | -5.755 |
| ZINC000100296828 | -5.754 |
| ZINC000100296828 | -5.754 |
| ZINC000001481833 | -5.753 |
| ZINC000000020255 | -5.749 |
| ZINC000003823492 | -5.744 |
| ZINC000000002101 | -5.737 |
| ZINC000000601316 | -5.737 |
| ZINC000000896740 | -5.736 |
| ZINC000000608101 | -5.734 |
| ZINC000000000449 | -5.733 |
| ZINC000006745272 | -5.732 |
| ZINC000000020220 | -5.722 |
| ZINC000000105196 | -5.722 |
| ZINC000003812984 | -5.722 |
| ZINC000003812984 | -5.722 |
| ZINC000002000707 | -5.719 |
| ZINC000000601250 | -5.718 |
| ZINC000000002299 | -5.716 |
| ZINC000000002299 | -5.716 |
| ZINC000000002299 | -5.716 |
| ZINC000001035331 | -5.713 |
| ZINC000003824921 | -5.712 |
| ZINC000043100953 | -5.707 |
| ZINC000043100953 | -5.707 |
| ZINC000000896595 | -5.706 |
| ZINC000043100953 | -5.705 |
| ZINC000000020220 | -5.703 |
| ZINC000000057313 | -5.703 |
| ZINC000004632106 | -5.702 |
| ZINC000001530611 | -5.701 |
| ZINC000003842753 | -5.701 |
| ZINC000003830961 | -5.699 |
| ZINC000003842753 | -5.694 |
| ZINC000006733300 | -5.693 |
| ZINC000053084692 | -5.693 |
| ZINC000053084692 | -5.69 |
| ZINC000000004028 | -5.689 |
| ZINC000000004028 | -5.689 |
| ZINC000000901791 | -5.684 |
| ZINC000005844792 | -5.675 |
| ZINC000000004785 | -5.671 |
| ZINC000003813003 | -5.669 |
| ZINC000005844792 | -5.669 |
| ZINC000000000746 | -5.667 |
| ZINC000000020241 | -5.66 |
| ZINC000003827556 | -5.659 |
| ZINC000000001281 | -5.658 |
| ZINC000004632106 | -5.658 |
| ZINC000000074836 | -5.658 |
| ZINC000001187543 | -5.657 |
| ZINC000019632912 | -5.654 |
| ZINC000003875368 | -5.649 |
| ZINC000003875368 | -5.649 |
| ZINC000000056652 | -5.648 |
| ZINC000000039089 | -5.647 |
| ZINC000000002281 | -5.643 |
| ZINC000001482049 | -5.637 |
| ZINC000001482049 | -5.636 |
| ZINC000000097996 | -5.635 |
| ZINC000003776633 | -5.633 |
| ZINC000003776633 | -5.633 |
| ZINC000000075008 | -5.633 |
| ZINC000000075008 | -5.633 |
| ZINC000000009689 | -5.633 |
| ZINC000000001132 | -5.632 |
| ZINC000000002272 | -5.632 |
| ZINC000003803652 | -5.631 |
| ZINC000004095696 | -5.629 |
| ZINC000001530695 | -5.627 |
| ZINC000001530695 | -5.627 |
| ZINC000001530695 | -5.627 |
| ZINC000006661227 | -5.627 |
| ZINC000006661227 | -5.627 |
| ZINC000006661227 | -5.627 |
| ZINC000000113426 | -5.627 |
| ZINC000000606383 | -5.627 |
| ZINC000004228257 | -5.626 |
| ZINC000000001979 | -5.621 |
| ZINC000001530555 | -5.617 |
| ZINC000022448696 | -5.614 |
| ZINC000022448696 | -5.614 |
| ZINC000022448696 | -5.614 |
| ZINC000022448696 | -5.614 |
| ZINC000022448696 | -5.614 |
| ZINC000003819138 | -5.614 |
| ZINC000000002043 | -5.613 |
| ZINC000000002043 | -5.613 |
| ZINC000003830391 | -5.613 |
| ZINC000011681563 | -5.61 |
| ZINC000011681563 | -5.61 |
| ZINC000029319828 | -5.603 |
| ZINC000000005560 | -5.603 |
| ZINC000000120319 | -5.602 |
| ZINC000000049153 | -5.602 |
| ZINC000000120319 | -5.601 |
| ZINC000000006156 | -5.601 |
| ZINC000019632670 | -5.598 |
| ZINC000019632670 | -5.598 |
| ZINC000019364222 | -5.596 |
| ZINC000019364222 | -5.596 |
| ZINC000019364222 | -5.596 |
| ZINC000000896918 | -5.594 |
| ZINC000003607120 | -5.591 |
| ZINC000004693575 | -5.59 |
| ZINC000014879992 | -5.589 |
| ZINC000003607120 | -5.588 |
| ZINC000003589203 | -5.588 |
| ZINC000003830391 | -5.586 |
| ZINC000002519740 | -5.584 |
| ZINC000002519740 | -5.584 |
| ZINC000002519740 | -5.584 |
| ZINC000018043251 | -5.583 |
| ZINC000000001261 | -5.583 |
| ZINC000004676424 | -5.581 |
| ZINC000100005670 | -5.573 |
| ZINC000013597823 | -5.564 |
| ZINC000013597823 | -5.563 |
| ZINC000000537957 | -5.562 |
| ZINC000012503291 | -5.561 |
| ZINC000034636383 | -5.554 |
| ZINC000003830891 | -5.551 |
| ZINC000101489663 | -5.548 |
| ZINC000029571072 | -5.546 |
| ZINC000029571072 | -5.542 |
| ZINC000000896703 | -5.535 |
| ZINC000002599970 | -5.533 |
| ZINC000000643143 | -5.531 |
| ZINC000002599970 | -5.529 |
| ZINC000000007782 | -5.525 |
| ZINC000006021043 | -5.52 |
| ZINC000001530806 | -5.52 |
| ZINC000003809490 | -5.519 |
| ZINC000003872605 | -5.518 |
| ZINC000000895081 | -5.517 |
| ZINC000011679756 | -5.514 |
| ZINC000000004724 | -5.513 |
| ZINC000006467621 | -5.513 |
| ZINC000003812913 | -5.513 |
| ZINC000001533877 | -5.512 |
| ZINC000001533877 | -5.512 |
| ZINC000003872605 | -5.51 |
| ZINC000000001688 | -5.507 |
| ZINC000000001688 | -5.507 |
| ZINC000003791297 | -5.506 |
| ZINC000001533877 | -5.499 |
| ZINC000001533877 | -5.499 |
| ZINC000001533877 | -5.499 |
| ZINC000001533877 | -5.499 |
| ZINC000003812851 | -5.499 |
| ZINC000001690324 | -5.498 |
| ZINC000001690324 | -5.498 |
| ZINC000003986735 | -5.496 |
| ZINC000003798247 | -5.496 |
| ZINC000003812851 | -5.495 |
| ZINC000003986735 | -5.491 |
| ZINC000003986735 | -5.491 |
| ZINC000004213946 | -5.486 |
| ZINC000043207238 | -5.486 |
| ZINC000000001655 | -5.483 |
| ZINC000011679756 | -5.482 |
| ZINC000008034121 | -5.48 |
| ZINC000035999642 | -5.48 |
| ZINC000035999642 | -5.473 |
| ZINC000021297660 | -5.473 |
| ZINC000021297660 | -5.473 |
| ZINC000006021033 | -5.473 |
| ZINC000084843283 | -5.473 |
| ZINC000000001644 | -5.472 |
| ZINC000004213946 | -5.47 |
| ZINC000001530601 | -5.468 |
| ZINC000004632106 | -5.466 |
| ZINC000004632106 | -5.466 |
| ZINC000003831474 | -5.463 |
| ZINC000018203737 | -5.462 |
| ZINC000018203737 | -5.462 |
| ZINC000008403947 | -5.461 |
| ZINC000000538621 | -5.459 |
| ZINC000000538621 | -5.459 |
| ZINC000000005823 | -5.458 |
| ZINC000000002273 | -5.457 |
| ZINC000035653007 | -5.455 |
| ZINC000001540998 | -5.453 |
| ZINC000002020233 | -5.45 |
| ZINC000001532517 | -5.447 |
| ZINC000000402909 | -5.447 |
| ZINC000000057146 | -5.444 |
| ZINC000003872931 | -5.444 |
| ZINC000003872931 | -5.444 |
| ZINC000002570817 | -5.443 |
| ZINC000000005423 | -5.441 |
| ZINC000003800706 | -5.44 |
| ZINC000003812863 | -5.437 |
| ZINC000252679615 | -5.426 |
| ZINC000252679615 | -5.426 |
| ZINC000002568036 | -5.426 |
| ZINC000002568036 | -5.426 |
| ZINC000007997966 | -5.426 |
| ZINC000007997966 | -5.426 |
| ZINC000000538564 | -5.423 |
| ZINC000000003642 | -5.423 |
| ZINC000000033882 | -5.422 |
| ZINC000003813042 | -5.42 |
| ZINC000004216238 | -5.42 |
| ZINC000003795098 | -5.417 |
| ZINC000000020251 | -5.417 |
| ZINC000007997568 | -5.417 |
| ZINC000007997568 | -5.417 |
| ZINC000011681534 | -5.413 |
| ZINC000000004893 | -5.413 |
| ZINC000000004893 | -5.413 |
| ZINC000000004893 | -5.413 |
| ZINC000001530803 | -5.412 |
| ZINC000000075126 | -5.408 |
| ZINC000000537822 | -5.408 |
| ZINC000000537822 | -5.408 |
| ZINC000011681534 | -5.406 |
| ZINC000019144216 | -5.405 |
| ZINC000019144216 | -5.405 |
| ZINC000000113428 | -5.403 |
| ZINC000001482184 | -5.401 |
| ZINC000013597823 | -5.4 |
| ZINC000001850377 | -5.399 |
| ZINC000003807804 | -5.399 |
| ZINC000000968257 | -5.395 |
| ZINC000000895302 | -5.394 |
| ZINC000000007295 | -5.393 |
| ZINC000000001758 | -5.391 |
| ZINC000003860453 | -5.39 |
| ZINC000013597823 | -5.39 |
| ZINC000000005823 | -5.389 |
| ZINC000013986658 | -5.383 |
| ZINC000013986658 | -5.383 |
| ZINC000012402836 | -5.378 |
| ZINC000012360535 | -5.374 |
| ZINC000003871703 | -5.373 |
| ZINC000000057533 | -5.371 |
| ZINC000000896819 | -5.368 |
| ZINC000058581064 | -5.367 |
| ZINC000000968263 | -5.367 |
| ZINC000000004448 | -5.366 |
| ZINC000002015928 | -5.363 |
| ZINC000035342789 | -5.36 |
| ZINC000001534965 | -5.359 |
| ZINC000000057253 | -5.358 |
| ZINC000000057253 | -5.358 |
| ZINC000001493878 | -5.357 |
| ZINC000013298313 | -5.356 |
| ZINC000005162311 | -5.355 |
| ZINC000019632917 | -5.35 |
| ZINC000051951647 | -5.349 |
| ZINC000000000416 | -5.346 |
| ZINC000000056646 | -5.346 |
| ZINC000034676245 | -5.346 |
| ZINC000034676245 | -5.346 |
| ZINC000000403010 | -5.344 |
| ZINC000000000416 | -5.344 |
| ZINC000000008667 | -5.34 |
| ZINC000001530805 | -5.34 |
| ZINC000000403010 | -5.34 |
| ZINC000003830579 | -5.337 |
| ZINC000000388081 | -5.333 |
| ZINC000003873160 | -5.331 |
| ZINC000003873160 | -5.331 |
| ZINC000000388081 | -5.329 |
| ZINC000006716957 | -5.328 |
| ZINC000003830999 | -5.323 |
| ZINC000003871967 | -5.322 |
| ZINC000001690604 | -5.321 |
| ZINC000019228902 | -5.32 |
| ZINC000019228902 | -5.32 |
| ZINC000019228902 | -5.32 |
| ZINC000022010387 | -5.32 |
| ZINC000022010387 | -5.32 |
| ZINC000022010387 | -5.32 |
| ZINC000000057206 | -5.317 |
| ZINC000000057206 | -5.316 |
| ZINC000000895199 | -5.314 |
| ZINC000008034120 | -5.311 |
| ZINC000000388081 | -5.307 |
| ZINC000022002214 | -5.306 |
| ZINC000022002214 | -5.306 |
| ZINC000000001011 | -5.305 |
| ZINC000000388462 | -5.305 |
| ZINC000003831040 | -5.303 |
| ZINC000003871978 | -5.295 |
| ZINC000000000607 | -5.294 |
| ZINC000000119344 | -5.294 |
| ZINC000006409735 | -5.293 |
| ZINC000006409735 | -5.293 |
| ZINC000001842633 | -5.291 |
| ZINC000009212428 | -5.291 |
| ZINC000000606383 | -5.29 |
| ZINC000008466459 | -5.287 |
| ZINC000003808779 | -5.287 |
| ZINC000022059268 | -5.285 |
| ZINC000000020231 | -5.284 |
| ZINC000001530713 | -5.282 |
| ZINC000019166991 | -5.282 |
| ZINC000001530427 | -5.28 |
| ZINC000001530427 | -5.28 |
| ZINC000001530427 | -5.28 |
| ZINC000006627681 | -5.275 |
| ZINC000001853550 | -5.273 |
| ZINC000002169830 | -5.271 |
| ZINC000001530968 | -5.269 |
| ZINC000001530968 | -5.269 |
| ZINC000012466082 | -5.267 |
| ZINC000252678020 | -5.267 |
| ZINC000002015035 | -5.266 |
| ZINC000002015035 | -5.266 |
| ZINC000030691760 | -5.265 |
| ZINC000001530639 | -5.263 |
| ZINC000003813010 | -5.257 |
| ZINC000003798734 | -5.255 |
| ZINC000001530599 | -5.247 |
| ZINC000100036536 | -5.246 |
| ZINC000100036536 | -5.246 |
| ZINC000001530599 | -5.245 |
| ZINC000012466082 | -5.244 |
| ZINC000252678020 | -5.244 |
| ZINC000000004778 | -5.244 |
| ZINC000012503068 | -5.243 |
| ZINC000003813010 | -5.241 |
| ZINC000000599985 | -5.24 |
| ZINC000016159083 | -5.24 |
| ZINC000016159083 | -5.24 |
| ZINC000003979899 | -5.238 |
| ZINC000000968275 | -5.235 |
| ZINC000003830569 | -5.235 |
| ZINC000000002216 | -5.234 |
| ZINC000000005878 | -5.233 |
| ZINC000003776970 | -5.233 |
| ZINC000000002216 | -5.232 |
| ZINC000100016084 | -5.23 |
| ZINC000003818726 | -5.23 |
| ZINC000019796155 | -5.229 |
| ZINC000019796155 | -5.229 |
| ZINC000000897258 | -5.227 |
| ZINC000003776970 | -5.225 |
| ZINC000000034157 | -5.224 |
| ZINC000001530600 | -5.224 |
| ZINC000001530600 | -5.224 |
| ZINC000000001317 | -5.221 |
| ZINC000000000061 | -5.221 |
| ZINC000000020228 | -5.219 |
| ZINC000000897258 | -5.219 |
| ZINC000000020228 | -5.219 |
| ZINC000000000931 | -5.216 |
| ZINC000000000931 | -5.216 |
| ZINC000001550499 | -5.215 |
| ZINC000052957434 | -5.215 |
| ZINC000052957434 | -5.215 |
| ZINC000000000882 | -5.212 |
| ZINC000000000882 | -5.212 |
| ZINC000001532526 | -5.212 |
| ZINC000100299039 | -5.21 |
| ZINC000003875259 | -5.21 |
| ZINC000003875259 | -5.21 |
| ZINC000019875504 | -5.207 |
| ZINC000003871923 | -5.203 |
| ZINC000019875504 | -5.2 |
| ZINC000001530688 | -5.2 |
| ZINC000009224016 | -5.197 |
| ZINC000009224016 | -5.197 |
| ZINC000001530728 | -5.195 |
| ZINC000001530728 | -5.195 |
| ZINC000000008492 | -5.195 |
| ZINC000100055899 | -5.194 |
| ZINC000009164421 | -5.193 |
| ZINC000004640636 | -5.189 |
| ZINC000004640636 | -5.189 |
| ZINC000100007011 | -5.189 |
| ZINC000000538275 | -5.187 |
| ZINC000000538275 | -5.187 |
| ZINC000019364224 | -5.187 |
| ZINC000019364224 | -5.187 |
| ZINC000019364224 | -5.187 |
| ZINC000005844788 | -5.185 |
| ZINC000005844788 | -5.185 |
| ZINC000001530694 | -5.185 |
| ZINC000001530930 | -5.184 |
| ZINC000000000903 | -5.182 |
| ZINC000000155269 | -5.181 |
| ZINC000000155269 | -5.181 |
| ZINC000000020231 | -5.18 |
| ZINC000000896968 | -5.179 |
| ZINC000004228257 | -5.178 |
| ZINC000000895457 | -5.178 |
| ZINC000000001331 | -5.175 |
| ZINC000000004448 | -5.175 |
| ZINC000001530930 | -5.173 |
| ZINC000003831165 | -5.17 |
| ZINC000013682481 | -5.17 |
| ZINC000003812867 | -5.167 |
| ZINC000001530886 | -5.163 |
| ZINC000000004166 | -5.162 |
| ZINC000000001706 | -5.162 |
| ZINC000022010379 | -5.162 |
| ZINC000022010379 | -5.162 |
| ZINC000022010379 | -5.162 |
| ZINC000013585233 | -5.161 |
| ZINC000000057435 | -5.16 |
| ZINC000000643153 | -5.159 |
| ZINC000004658603 | -5.158 |
| ZINC000004658603 | -5.158 |
| ZINC000000006016 | -5.156 |
| ZINC000000006016 | -5.156 |
| ZINC000001542113 | -5.156 |
| ZINC000001542113 | -5.156 |
| ZINC000000002279 | -5.155 |
| ZINC000095452610 | -5.154 |
| ZINC000019362735 | -5.153 |
| ZINC000019362735 | -5.153 |
| ZINC000019362735 | -5.153 |
| ZINC000000000507 | -5.153 |
| ZINC000004475353 | -5.152 |
| ZINC000019702309 | -5.152 |
| ZINC000013585233 | -5.152 |
| ZINC000003812863 | -5.152 |
| ZINC000000105216 | -5.151 |
| ZINC000000049154 | -5.148 |
| ZINC000000000856 | -5.147 |
| ZINC000000000507 | -5.146 |
| ZINC000072318121 | -5.144 |
| ZINC000072318121 | -5.144 |
| ZINC000022059930 | -5.142 |
| ZINC000008855117 | -5.141 |
| ZINC000000002028 | -5.141 |
| ZINC000008855117 | -5.141 |
| ZINC000000509440 | -5.14 |
| ZINC000000006251 | -5.139 |
| ZINC000000006251 | -5.139 |
| ZINC000000001795 | -5.136 |
| ZINC000000388081 | -5.136 |
| ZINC000014961096 | -5.134 |
| ZINC000000002191 | -5.134 |
| ZINC000000014360 | -5.133 |
| ZINC000001530814 | -5.131 |
| ZINC000001530814 | -5.131 |
| ZINC000001530814 | -5.131 |
| ZINC000000009073 | -5.129 |
| ZINC000000009073 | -5.129 |
| ZINC000000009073 | -5.129 |
| ZINC000000083315 | -5.129 |
| ZINC000001999441 | -5.126 |
| ZINC000000388081 | -5.126 |
| ZINC000034676245 | -5.126 |
| ZINC000000000509 | -5.125 |
| ZINC000000000509 | -5.125 |
| ZINC000000000509 | -5.125 |
| ZINC000003812306 | -5.125 |
| ZINC000000388081 | -5.124 |
| ZINC000000896958 | -5.12 |
| ZINC000006382803 | -5.114 |
| ZINC000001999441 | -5.113 |
| ZINC000003818726 | -5.113 |
| ZINC000003872931 | -5.111 |
| ZINC000003872931 | -5.111 |
| ZINC000000056647 | -5.11 |
| ZINC000016929327 | -5.109 |
| ZINC000000003876 | -5.109 |
| ZINC000000403566 | -5.108 |
| ZINC000000968330 | -5.105 |
| ZINC000000057001 | -5.102 |
| ZINC000028467879 | -5.096 |
| ZINC000028467879 | -5.096 |
| ZINC000000491073 | -5.096 |
| ZINC000000491073 | -5.096 |
| ZINC000000491073 | -5.096 |
| ZINC000000056556 | -5.096 |
| ZINC000100032379 | -5.094 |
| ZINC000000000905 | -5.093 |
| ZINC000000007601 | -5.09 |
| ZINC000000006481 | -5.089 |
| ZINC000006661227 | -5.089 |
| ZINC000006661227 | -5.089 |
| ZINC000006661227 | -5.089 |
| ZINC000001530939 | -5.089 |
| ZINC000001530600 | -5.087 |
| ZINC000013129998 | -5.086 |
| ZINC000008214692 | -5.085 |
| ZINC000000537931 | -5.084 |
| ZINC000000121480 | -5.082 |
| ZINC000003873160 | -5.079 |
| ZINC000001543916 | -5.078 |
| ZINC000001543916 | -5.077 |
| ZINC000003873160 | -5.077 |
| ZINC000000020221 | -5.071 |
| ZINC000003812869 | -5.07 |
| ZINC000100032379 | -5.063 |
| ZINC000001883067 | -5.061 |
| ZINC000001886617 | -5.059 |
| ZINC000003812944 | -5.058 |
| ZINC000001530760 | -5.056 |
| ZINC000001530776 | -5.056 |
| ZINC000035328014 | -5.055 |
| ZINC000000020248 | -5.052 |
| ZINC000000057513 | -5.051 |
| ZINC000000057513 | -5.051 |
| ZINC000003982483 | -5.05 |
| ZINC000003982483 | -5.05 |
| ZINC000003982483 | -5.05 |
| ZINC000000113355 | -5.049 |
| ZINC000001530697 | -5.044 |
| ZINC000001530697 | -5.044 |
| ZINC000001530697 | -5.044 |
| ZINC000013682481 | -5.043 |
| ZINC000003830500 | -5.043 |
| ZINC000001530617 | -5.042 |
| ZINC000000000346 | -5.041 |
| ZINC000000000922 | -5.04 |
| ZINC000008214514 | -5.04 |
| ZINC000013537284 | -5.039 |
| ZINC000028973441 | -5.039 |
| ZINC000003812869 | -5.039 |
| ZINC000000896740 | -5.036 |
| ZINC000000001899 | -5.036 |
| ZINC000000001148 | -5.034 |
| ZINC000000057062 | -5.033 |
| ZINC000000014257 | -5.03 |
| ZINC000012503099 | -5.03 |
| ZINC000012503099 | -5.03 |
| ZINC000001530652 | -5.029 |
| ZINC000001530652 | -5.029 |
| ZINC000000622123 | -5.026 |
| ZINC000000968330 | -5.025 |
| ZINC000006036847 | -5.021 |
| ZINC000003830999 | -5.02 |
| ZINC000000001931 | -5.019 |
| ZINC000000001931 | -5.019 |
| ZINC000000968273 | -5.017 |
| ZINC000013550868 | -5.015 |
| ZINC000003830500 | -5.014 |
| ZINC000003830986 | -5.012 |
| ZINC000000002009 | -5.011 |
| ZINC000001531008 | -5.011 |
| ZINC000003651680 | -5.008 |
| ZINC000019796080 | -5.008 |
| ZINC000019796080 | -5.008 |
| ZINC000019796080 | -5.008 |
| ZINC000002847375 | -5.008 |
| ZINC000002847375 | -5.008 |
| ZINC000019796018 | -5.006 |
| ZINC000019796018 | -5.006 |
| ZINC000019796018 | -5.006 |
| ZINC000003651680 | -5.005 |
| ZINC000013537284 | -5.004 |
| ZINC000003831475 | -4.999 |
| ZINC000000001115 | -4.998 |
| ZINC000013545634 | -4.996 |
| ZINC000013545634 | -4.994 |
| ZINC000003964126 | -4.993 |
| ZINC000000020244 | -4.992 |
| ZINC000000020244 | -4.992 |
| ZINC000000601305 | -4.992 |
| ZINC000001481910 | -4.99 |
| ZINC000049637509 | -4.989 |
| ZINC000052716421 | -4.989 |
| ZINC000052716421 | -4.989 |
| ZINC000001530713 | -4.982 |
| ZINC000003929508 | -4.978 |
| ZINC000003792417 | -4.978 |
| ZINC000000015515 | -4.978 |
| ZINC000001850376 | -4.974 |
| ZINC000000968233 | -4.974 |
| ZINC000000056427 | -4.972 |
| ZINC000001849548 | -4.97 |
| ZINC000000044027 | -4.97 |
| ZINC000003079340 | -4.968 |
| ZINC000000403079 | -4.966 |
| ZINC000001536779 | -4.964 |
| ZINC000003830441 | -4.963 |
| ZINC000043202140 | -4.963 |
| ZINC000003830993 | -4.961 |
| ZINC000003812983 | -4.961 |
| ZINC000003812983 | -4.961 |
| ZINC000000000693 | -4.96 |
| ZINC000000403079 | -4.96 |
| ZINC000000005895 | -4.959 |
| ZINC000000897322 | -4.956 |
| ZINC000013537284 | -4.951 |
| ZINC000043202140 | -4.951 |
| ZINC000000895032 | -4.95 |
| ZINC000003782818 | -4.95 |
| ZINC000003782818 | -4.95 |
| ZINC000003830993 | -4.949 |
| ZINC000000897408 | -4.948 |
| ZINC000003873296 | -4.938 |
| ZINC000008101126 | -4.937 |
| ZINC000014210876 | -4.937 |
| ZINC000014210876 | -4.937 |
| ZINC000021303210 | -4.937 |
| ZINC000049637509 | -4.936 |
| ZINC000001843099 | -4.932 |
| ZINC000007997897 | -4.93 |
| ZINC000007997897 | -4.93 |
| ZINC000007997897 | -4.93 |
| ZINC000001530973 | -4.923 |
| ZINC000017146904 | -4.922 |
| ZINC000001995484 | -4.918 |
| ZINC000000005151 | -4.918 |
| ZINC000000005151 | -4.918 |
| ZINC000000599985 | -4.916 |
| ZINC000003872994 | -4.914 |
| ZINC000030691727 | -4.914 |
| ZINC000000010164 | -4.913 |
| ZINC000000010164 | -4.913 |
| ZINC000002019954 | -4.912 |
| ZINC000084668739 | -4.91 |
| ZINC000000057341 | -4.905 |
| ZINC000000537795 | -4.904 |
| ZINC000000601229 | -4.904 |
| ZINC000000601229 | -4.904 |
| ZINC000000601229 | -4.904 |
| ZINC000000057278 | -4.903 |
| ZINC000000000373 | -4.901 |
| ZINC000002019954 | -4.898 |
| ZINC000000896634 | -4.894 |
| ZINC000001542392 | -4.884 |
| ZINC000002522648 | -4.882 |
| ZINC000001995484 | -4.879 |
| ZINC000000056645 | -4.878 |
| ZINC000005179119 | -4.876 |
| ZINC000000538621 | -4.876 |
| ZINC000001530710 | -4.875 |
| ZINC000000968305 | -4.871 |
| ZINC000000968305 | -4.871 |
| ZINC000000136138 | -4.871 |
| ZINC000000896698 | -4.869 |
| ZINC000000896698 | -4.869 |
| ZINC000253476027 | -4.867 |
| ZINC000100015775 | -4.867 |
| ZINC000008220878 | -4.867 |
| ZINC000008220878 | -4.867 |
| ZINC000000518554 | -4.867 |
| ZINC000003786192 | -4.867 |
| ZINC000000136138 | -4.863 |
| ZINC000000002159 | -4.862 |
| ZINC000001530618 | -4.859 |
| ZINC000003978005 | -4.859 |
| ZINC000003978005 | -4.859 |
| ZINC000003978005 | -4.859 |
| ZINC000000000456 | -4.858 |
| ZINC000000000456 | -4.858 |
| ZINC000000000456 | -4.858 |
| ZINC000004097426 | -4.852 |
| ZINC000004097426 | -4.852 |
| ZINC000004097426 | -4.852 |
| ZINC000019362737 | -4.851 |
| ZINC000019362737 | -4.851 |
| ZINC000019362737 | -4.851 |
| ZINC000000020257 | -4.849 |
| ZINC000000113398 | -4.848 |
| ZINC000003831151 | -4.844 |
| ZINC000000120286 | -4.844 |
| ZINC000004228257 | -4.841 |
| ZINC000002008866 | -4.84 |
| ZINC000003831531 | -4.838 |
| ZINC000003831531 | -4.838 |
| ZINC000005224188 | -4.837 |
| ZINC000001530707 | -4.837 |
| ZINC000006627681 | -4.831 |
| ZINC000000000973 | -4.83 |
| ZINC000005733652 | -4.83 |
| ZINC000005733652 | -4.83 |
| ZINC000005733652 | -4.829 |
| ZINC000005733652 | -4.829 |
| ZINC000000896455 | -4.829 |
| ZINC000000000973 | -4.829 |
| ZINC000003794601 | -4.829 |
| ZINC000001530652 | -4.829 |
| ZINC000001530652 | -4.829 |
| ZINC000003806413 | -4.829 |
| ZINC000003806413 | -4.826 |
| ZINC000000020243 | -4.823 |
| ZINC000001548097 | -4.815 |
| ZINC000001548097 | -4.815 |
| ZINC000040899447 | -4.815 |
| ZINC000064033452 | -4.809 |
| ZINC000001530283 | -4.807 |
| ZINC000000000053 | -4.806 |
| ZINC000000901061 | -4.806 |
| ZINC000000967566 | -4.806 |
| ZINC000000020259 | -4.805 |
| ZINC000000119717 | -4.797 |
| ZINC000000113410 | -4.796 |
| ZINC000019361042 | -4.793 |
| ZINC000019361042 | -4.793 |
| ZINC000027428713 | -4.792 |
| ZINC000000156395 | -4.789 |
| ZINC000027428713 | -4.783 |
| ZINC000003807917 | -4.783 |
| ZINC000000897256 | -4.781 |
| ZINC000003872277 | -4.778 |
| ZINC000000897256 | -4.777 |
| ZINC000021982937 | -4.777 |
| ZINC000021982937 | -4.777 |
| ZINC000095619100 | -4.774 |
| ZINC000095619100 | -4.774 |
| ZINC000000897240 | -4.771 |
| ZINC000000897240 | -4.771 |
| ZINC000000897240 | -4.771 |
| ZINC000019702309 | -4.771 |
| ZINC000003918138 | -4.77 |
| ZINC000022010375 | -4.77 |
| ZINC000022010375 | -4.77 |
| ZINC000022010375 | -4.77 |
| ZINC000003775644 | -4.765 |
| ZINC000100014475 | -4.764 |
| ZINC000034220093 | -4.76 |
| ZINC000001530637 | -4.76 |
| ZINC000003938482 | -4.755 |
| ZINC000004215234 | -4.749 |
| ZINC000018203737 | -4.747 |
| ZINC000018203737 | -4.747 |
| ZINC000021303210 | -4.746 |
| ZINC000000154964 | -4.742 |
| ZINC000000154964 | -4.742 |
| ZINC000000154964 | -4.742 |
| ZINC000001530862 | -4.742 |
| ZINC000000537791 | -4.741 |
| ZINC000000896484 | -4.739 |
| ZINC000000896484 | -4.739 |
| ZINC000051133897 | -4.738 |
| ZINC000003932831 | -4.734 |
| ZINC000000901555 | -4.725 |
| ZINC000012503177 | -4.714 |
| ZINC000003938686 | -4.714 |
| ZINC000085540219 | -4.713 |
| ZINC000018516586 | -4.711 |
| ZINC000001530706 | -4.71 |
| ZINC000003938686 | -4.709 |
| ZINC000003795819 | -4.708 |
| ZINC000003795819 | -4.708 |
| ZINC000000001408 | -4.706 |
| ZINC000000968327 | -4.706 |
| ZINC000000002005 | -4.703 |
| ZINC000001576892 | -4.701 |
| ZINC000003798537 | -4.7 |
| ZINC000004340269 | -4.699 |
| ZINC000001996784 | -4.697 |
| ZINC000000001145 | -4.697 |
| ZINC000000001145 | -4.696 |
| ZINC000003830713 | -4.695 |
| ZINC000001530599 | -4.694 |
| ZINC000004474443 | -4.694 |
| ZINC000000004319 | -4.694 |
| ZINC000012503151 | -4.693 |
| ZINC000253917094 | -4.693 |
| ZINC000000004319 | -4.693 |
| ZINC000096006009 | -4.693 |
| ZINC000043206370 | -4.693 |
| ZINC000004474443 | -4.691 |
| ZINC000096006009 | -4.689 |
| ZINC000001542392 | -4.689 |
| ZINC000000508068 | -4.687 |
| ZINC000000968327 | -4.687 |
| ZINC000000000740 | -4.685 |
| ZINC000000967520 | -4.678 |
| ZINC000028957444 | -4.677 |
| ZINC000013520815 | -4.668 |
| ZINC000012503156 | -4.666 |
| ZINC000000020250 | -4.661 |
| ZINC000004340269 | -4.661 |
| ZINC000000395010 | -4.66 |
| ZINC000000000506 | -4.66 |
| ZINC000000895042 | -4.659 |
| ZINC000004258316 | -4.658 |
| ZINC000001530725 | -4.655 |
| ZINC000006827693 | -4.653 |
| ZINC000006827693 | -4.653 |
| ZINC000000000242 | -4.651 |
| ZINC000000000242 | -4.651 |
| ZINC000001530654 | -4.648 |
| ZINC000001530654 | -4.648 |
| ZINC000014210457 | -4.648 |
| ZINC000003875259 | -4.645 |
| ZINC000018099446 | -4.645 |
| ZINC000100019007 | -4.645 |
| ZINC000003875259 | -4.642 |
| ZINC000012661824 | -4.638 |
| ZINC000019632706 | -4.634 |
| ZINC000003812974 | -4.633 |
| ZINC000003781664 | -4.632 |
| ZINC000003794711 | -4.629 |
| ZINC000003794711 | -4.629 |
| ZINC000003794711 | -4.629 |
| ZINC000000897385 | -4.627 |
| ZINC000000643055 | -4.624 |
| ZINC000003782550 | -4.618 |
| ZINC000003782550 | -4.617 |
| ZINC000000056653 | -4.615 |
| ZINC000001482113 | -4.615 |
| ZINC000026985532 | -4.615 |
| ZINC000026985532 | -4.615 |
| ZINC000026985532 | -4.615 |
| ZINC000000538483 | -4.612 |
| ZINC000000538483 | -4.612 |
| ZINC000000000083 | -4.61 |
| ZINC000000968310 | -4.607 |
| ZINC000000968310 | -4.607 |
| ZINC000000968310 | -4.607 |
| ZINC000084441937 | -4.607 |
| ZINC000003872738 | -4.606 |
| ZINC000000005823 | -4.604 |
| ZINC000001482094 | -4.603 |
| ZINC000005764759 | -4.6 |
| ZINC000001530599 | -4.599 |
| ZINC000003201907 | -4.593 |
| ZINC000003201907 | -4.593 |
| ZINC000006094354 | -4.592 |
| ZINC000006094354 | -4.592 |
| ZINC000095452610 | -4.592 |
| ZINC000000584092 | -4.592 |
| ZINC000003831282 | -4.591 |
| ZINC000003787097 | -4.589 |
| ZINC000003831051 | -4.589 |
| ZINC000000601283 | -4.583 |
| ZINC000000001681 | -4.582 |
| ZINC000000001681 | -4.582 |
| ZINC000001482164 | -4.578 |
| ZINC000000001798 | -4.574 |
| ZINC000000001798 | -4.574 |
| ZINC000000001798 | -4.574 |
| ZINC000253476025 | -4.57 |
| ZINC000100015780 | -4.57 |
| ZINC000000001505 | -4.569 |
| ZINC000100036924 | -4.565 |
| ZINC000000968328 | -4.56 |
| ZINC000030691754 | -4.559 |
| ZINC000003873936 | -4.557 |
| ZINC000003873936 | -4.557 |
| ZINC000001895505 | -4.552 |
| ZINC000000020240 | -4.55 |
| ZINC000001489478 | -4.55 |
| ZINC000022116608 | -4.549 |
| ZINC000034051848 | -4.549 |
| ZINC000000000323 | -4.548 |
| ZINC000000537964 | -4.546 |
| ZINC000100071256 | -4.544 |
| ZINC000003831551 | -4.543 |
| ZINC000000057464 | -4.541 |
| ZINC000003830339 | -4.538 |
| ZINC000019156872 | -4.535 |
| ZINC000019156872 | -4.535 |
| ZINC000019156872 | -4.535 |
| ZINC000003989268 | -4.535 |
| ZINC000096006023 | -4.535 |
| ZINC000013545636 | -4.534 |
| ZINC000036294079 | -4.533 |
| ZINC000036294079 | -4.531 |
| ZINC000084758479 | -4.531 |
| ZINC000084758479 | -4.531 |
| ZINC000000000655 | -4.529 |
| ZINC000013545636 | -4.529 |
| ZINC000022116608 | -4.528 |
| ZINC000040430143 | -4.528 |
| ZINC000034051848 | -4.525 |
| ZINC000000391812 | -4.521 |
| ZINC000000391812 | -4.521 |
| ZINC000000391812 | -4.521 |
| ZINC000012503076 | -4.513 |
| ZINC000001489478 | -4.513 |
| ZINC000003784120 | -4.51 |
| ZINC000000020253 | -4.51 |
| ZINC000000057532 | -4.509 |
| ZINC000000538550 | -4.5 |
| ZINC000000538550 | -4.5 |
| ZINC000000057512 | -4.499 |
| ZINC000021982937 | -4.493 |
| ZINC000021982937 | -4.493 |
| ZINC000021982937 | -4.493 |
| ZINC000000014037 | -4.489 |
| ZINC000004474405 | -4.484 |
| ZINC000000006300 | -4.483 |
| ZINC000000007673 | -4.48 |
| ZINC000116473771 | -4.478 |
| ZINC000072267023 | -4.476 |
| ZINC000072267023 | -4.476 |
| ZINC000000000490 | -4.473 |
| ZINC000001530621 | -4.471 |
| ZINC000003978006 | -4.471 |
| ZINC000001530621 | -4.471 |
| ZINC000000010163 | -4.469 |
| ZINC000000010163 | -4.469 |
| ZINC000003874715 | -4.469 |
| ZINC000004468778 | -4.468 |
| ZINC000003830716 | -4.466 |
| ZINC000003830716 | -4.466 |
| ZINC000003806063 | -4.464 |
| ZINC000000004949 | -4.464 |
| ZINC000052971887 | -4.462 |
| ZINC000000895034 | -4.462 |
| ZINC000003927198 | -4.459 |
| ZINC000003830842 | -4.458 |
| ZINC000000113442 | -4.458 |
| ZINC000000896711 | -4.457 |
| ZINC000014768621 | -4.456 |
| ZINC000003953037 | -4.455 |
| ZINC000100061056 | -4.454 |
| ZINC000116473771 | -4.452 |
| ZINC000052971887 | -4.451 |
| ZINC000000057255 | -4.451 |
| ZINC000000607971 | -4.449 |
| ZINC000012358719 | -4.448 |
| ZINC000001532728 | -4.448 |
| ZINC000004474682 | -4.448 |
| ZINC000003881958 | -4.447 |
| ZINC000116473771 | -4.445 |
| ZINC000003818808 | -4.441 |
| ZINC000003953037 | -4.44 |
| ZINC000000020237 | -4.439 |
| ZINC000000020237 | -4.439 |
| ZINC000003973334 | -4.438 |
| ZINC000000003911 | -4.437 |
| ZINC000000538621 | -4.433 |
| ZINC000000057534 | -4.433 |
| ZINC000000895034 | -4.432 |
| ZINC000000005823 | -4.431 |
| ZINC000003918453 | -4.431 |
| ZINC000000895154 | -4.428 |
| ZINC000053683345 | -4.428 |
| ZINC000016052277 | -4.425 |
| ZINC000002036848 | -4.421 |
| ZINC000002036848 | -4.421 |
| ZINC000003814395 | -4.421 |
| ZINC000116473771 | -4.421 |
| ZINC000003830276 | -4.417 |
| ZINC000003814422 | -4.414 |
| ZINC000060325170 | -4.412 |
| ZINC000001999487 | -4.411 |
| ZINC000000006226 | -4.404 |
| ZINC000003875484 | -4.401 |
| ZINC000022447798 | -4.401 |
| ZINC000022447798 | -4.401 |
| ZINC000035328014 | -4.4 |
| ZINC000022010382 | -4.4 |
| ZINC000022010382 | -4.4 |
| ZINC000022010382 | -4.4 |
| ZINC000005133329 | -4.398 |
| ZINC000000538312 | -4.397 |
| ZINC000000538312 | -4.397 |
| ZINC000003875483 | -4.396 |
| ZINC000003875483 | -4.396 |
| ZINC000003875483 | -4.396 |
| ZINC000003875342 | -4.395 |
| ZINC000000001554 | -4.395 |
| ZINC000000006310 | -4.393 |
| ZINC000003875980 | -4.392 |
| ZINC000001530886 | -4.392 |
| ZINC000000000596 | -4.391 |
| ZINC000000000596 | -4.391 |
| ZINC000000000565 | -4.39 |
| ZINC000000000565 | -4.39 |
| ZINC000035024346 | -4.389 |
| ZINC000001538857 | -4.389 |
| ZINC000003801163 | -4.389 |
| ZINC000001530689 | -4.389 |
| ZINC000001530689 | -4.389 |
| ZINC000004097392 | -4.385 |
| ZINC000000968345 | -4.384 |
| ZINC000100009280 | -4.38 |
| ZINC000100009280 | -4.38 |
| ZINC000000968345 | -4.379 |
| ZINC000019796087 | -4.376 |
| ZINC000019796087 | -4.376 |
| ZINC000019796087 | -4.376 |
| ZINC000003831417 | -4.368 |
| ZINC000011677857 | -4.366 |
| ZINC000013587680 | -4.366 |
| ZINC000000643114 | -4.365 |
| ZINC000222731806 | -4.362 |
| ZINC000001530638 | -4.361 |
| ZINC000000002647 | -4.352 |
| ZINC000022010649 | -4.35 |
| ZINC000003976838 | -4.348 |
| ZINC000003976838 | -4.348 |
| ZINC000003938751 | -4.345 |
| ZINC000022065398 | -4.344 |
| ZINC000013912394 | -4.342 |
| ZINC000000896569 | -4.341 |
| ZINC000001530929 | -4.338 |
| ZINC000001530929 | -4.337 |
| ZINC000000020783 | -4.336 |
| ZINC000019168887 | -4.336 |
| ZINC000003922770 | -4.334 |
| ZINC000043763856 | -4.332 |
| ZINC000004258316 | -4.332 |
| ZINC000019144226 | -4.331 |
| ZINC000003831405 | -4.331 |
| ZINC000003831405 | -4.331 |
| ZINC000000056568 | -4.329 |
| ZINC000000057340 | -4.327 |
| ZINC000000000471 | -4.327 |
| ZINC000001843099 | -4.325 |
| ZINC000095616601 | -4.323 |
| ZINC000018516586 | -4.321 |
| ZINC000003876069 | -4.316 |
| ZINC000000001464 | -4.314 |
| ZINC000001530817 | -4.313 |
| ZINC000001530817 | -4.313 |
| ZINC000003876069 | -4.311 |
| ZINC000019796158 | -4.311 |
| ZINC000019796158 | -4.311 |
| ZINC000022116612 | -4.307 |
| ZINC000000000122 | -4.302 |
| ZINC000000000122 | -4.302 |
| ZINC000003794794 | -4.302 |
| ZINC000095616601 | -4.3 |
| ZINC000003993846 | -4.3 |
| ZINC000000002176 | -4.3 |
| ZINC000094566093 | -4.3 |
| ZINC000003927198 | -4.299 |
| ZINC000004428529 | -4.298 |
| ZINC000118912517 | -4.298 |
| ZINC000014879992 | -4.297 |
| ZINC000014879992 | -4.297 |
| ZINC000003079342 | -4.296 |
| ZINC000002005550 | -4.295 |
| ZINC000095564694 | -4.295 |
| ZINC000095564694 | -4.295 |
| ZINC000095564694 | -4.295 |
| ZINC000000537805 | -4.294 |
| ZINC000001530977 | -4.292 |
| ZINC000001530977 | -4.292 |
| ZINC000001530977 | -4.292 |
| ZINC000000537805 | -4.292 |
| ZINC000014879992 | -4.289 |
| ZINC000002005550 | -4.286 |
| ZINC000003977981 | -4.285 |
| ZINC000003813083 | -4.285 |
| ZINC000003818808 | -4.285 |
| ZINC000000001411 | -4.282 |
| ZINC000100017856 | -4.282 |
| ZINC000100017856 | -4.282 |
| ZINC000003830957 | -4.277 |
| ZINC000003802417 | -4.271 |
| ZINC000003802417 | -4.271 |
| ZINC000000001728 | -4.268 |
| ZINC000003876068 | -4.267 |
| ZINC000001530975 | -4.266 |
| ZINC000001530975 | -4.266 |
| ZINC000003876068 | -4.266 |
| ZINC000000155905 | -4.263 |
| ZINC000000113446 | -4.256 |
| ZINC000003930376 | -4.256 |
| ZINC000000896663 | -4.253 |
| ZINC000006716957 | -4.253 |
| ZINC000003830453 | -4.252 |
| ZINC000003813088 | -4.248 |
| ZINC000002561203 | -4.246 |
| ZINC000019144231 | -4.245 |
| ZINC000004097286 | -4.242 |
| ZINC000011617039 | -4.241 |
| ZINC000008214614 | -4.241 |
| ZINC000019632834 | -4.24 |
| ZINC000001530761 | -4.236 |
| ZINC000003872055 | -4.234 |
| ZINC000019144231 | -4.234 |
| ZINC000001571009 | -4.233 |
| ZINC000033965961 | -4.232 |
| ZINC000033965961 | -4.232 |
| ZINC000033965961 | -4.232 |
| ZINC000033965961 | -4.229 |
| ZINC000033965961 | -4.229 |
| ZINC000002016037 | -4.229 |
| ZINC000003964325 | -4.227 |
| ZINC000000001267 | -4.226 |
| ZINC000004658562 | -4.225 |
| ZINC000001639567 | -4.224 |
| ZINC000019632834 | -4.221 |
| ZINC000019632834 | -4.221 |
| ZINC000000012346 | -4.22 |
| ZINC000018089317 | -4.219 |
| ZINC000116473771 | -4.211 |
| ZINC000003861768 | -4.21 |
| ZINC000004693574 | -4.209 |
| ZINC000000049154 | -4.208 |
| ZINC000052509463 | -4.204 |
| ZINC000003874498 | -4.203 |
| ZINC000001530912 | -4.201 |
| ZINC000001530912 | -4.201 |
| ZINC000001530912 | -4.201 |
| ZINC000118912393 | -4.2 |
| ZINC000000601301 | -4.199 |
| ZINC000008214402 | -4.197 |
| ZINC000008214402 | -4.197 |
| ZINC000085540215 | -4.197 |
| ZINC000000001792 | -4.196 |
| ZINC000003823475 | -4.192 |
| ZINC000003823475 | -4.192 |
| ZINC000003823475 | -4.192 |
| ZINC000003819392 | -4.188 |
| ZINC000004658560 | -4.185 |
| ZINC000095616599 | -4.181 |
| ZINC000001530569 | -4.181 |
| ZINC000030691736 | -4.18 |
| ZINC000012859773 | -4.179 |
| ZINC000000002688 | -4.179 |
| ZINC000003812988 | -4.178 |
| ZINC000003812988 | -4.178 |
| ZINC000003874950 | -4.177 |
| ZINC000001552174 | -4.176 |
| ZINC000410428674 | -4.174 |
| ZINC000036520252 | -4.174 |
| ZINC000001546066 | -4.17 |
| ZINC000052509463 | -4.169 |
| ZINC000013537284 | -4.169 |
| ZINC000000156792 | -4.167 |
| ZINC000000895099 | -4.166 |
| ZINC000052509366 | -4.165 |
| ZINC000000000215 | -4.164 |
| ZINC000000000215 | -4.164 |
| ZINC000003776875 | -4.164 |
| ZINC000001485935 | -4.158 |
| ZINC000003806104 | -4.156 |
| ZINC000033943508 | -4.156 |
| ZINC000085537014 | -4.154 |
| ZINC000085537014 | -4.154 |
| ZINC000003629271 | -4.153 |
| ZINC000013831141 | -4.151 |
| ZINC000000896666 | -4.151 |
| ZINC000000896463 | -4.146 |
| ZINC000019632633 | -4.141 |
| ZINC000019632633 | -4.141 |
| ZINC000019632633 | -4.141 |
| ZINC000118912450 | -4.138 |
| ZINC000004097427 | -4.136 |
| ZINC000004097427 | -4.136 |
| ZINC000000968328 | -4.136 |
| ZINC000022002218 | -4.132 |
| ZINC000022002218 | -4.132 |
| ZINC000004693574 | -4.131 |
| ZINC000004468780 | -4.128 |
| ZINC000001542392 | -4.123 |
| ZINC000000896755 | -4.122 |
| ZINC000100017856 | -4.122 |
| ZINC000100017856 | -4.122 |
| ZINC000003813078 | -4.121 |
| ZINC000004392649 | -4.114 |
| ZINC000084400879 | -4.113 |
| ZINC000084400879 | -4.113 |
| ZINC000084400879 | -4.113 |
| ZINC000000901552 | -4.108 |
| ZINC000003820029 | -4.107 |
| ZINC000001843047 | -4.107 |
| ZINC000001530567 | -4.105 |
| ZINC000001843099 | -4.105 |
| ZINC000004097305 | -4.104 |
| ZINC000096942202 | -4.1 |
| ZINC000000000751 | -4.1 |
| ZINC000000000751 | -4.1 |
| ZINC000000000751 | -4.1 |
| ZINC000012503187 | -4.096 |
| ZINC000003939013 | -4.095 |
| ZINC000095626706 | -4.095 |
| ZINC000052509366 | -4.093 |
| ZINC000001530816 | -4.092 |
| ZINC000001530816 | -4.092 |
| ZINC000000389149 | -4.089 |
| ZINC000000016154 | -4.089 |
| ZINC000012404516 | -4.089 |
| ZINC000012404516 | -4.089 |
| ZINC000003938746 | -4.087 |
| ZINC000003938746 | -4.087 |
| ZINC000003938746 | -4.087 |
| ZINC000084441937 | -4.087 |
| ZINC000084441937 | -4.087 |
| ZINC000000597013 | -4.082 |
| ZINC000000597013 | -4.082 |
| ZINC000009302239 | -4.08 |
| ZINC000100037020 | -4.08 |
| ZINC000100037020 | -4.08 |
| ZINC000100037020 | -4.08 |
| ZINC000004217732 | -4.077 |
| ZINC000003871701 | -4.074 |
| ZINC000000897288 | -4.073 |
| ZINC000000266964 | -4.07 |
| ZINC000000895103 | -4.069 |
| ZINC000003830212 | -4.068 |
| ZINC000003830212 | -4.068 |
| ZINC000007997952 | -4.068 |
| ZINC000007997952 | -4.068 |
| ZINC000004577910 | -4.065 |
| ZINC000003955219 | -4.064 |
| ZINC000003977764 | -4.064 |
| ZINC000003977978 | -4.056 |
| ZINC000000596731 | -4.053 |
| ZINC000000596731 | -4.053 |
| ZINC000012503187 | -4.05 |
| ZINC000004097476 | -4.047 |
| ZINC000100009278 | -4.043 |
| ZINC000100009278 | -4.043 |
| ZINC000085540223 | -4.043 |
| ZINC000000537805 | -4.042 |
| ZINC000001542199 | -4.041 |
| ZINC000012503187 | -4.04 |
| ZINC000008552123 | -4.037 |
| ZINC000008552123 | -4.037 |
| ZINC000008552123 | -4.037 |
| ZINC000013648755 | -4.03 |
| ZINC000013648755 | -4.028 |
| ZINC000001611274 | -4.028 |
| ZINC000003873295 | -4.026 |
| ZINC000001530716 | -4.025 |
| ZINC000001611274 | -4.025 |
| ZINC000028232750 | -4.024 |
| ZINC000028232750 | -4.023 |
| ZINC000028232750 | -4.02 |
| ZINC000002008310 | -4.007 |
| ZINC000003830347 | -4.005 |
| ZINC000003830347 | -4.005 |
| ZINC000028639340 | -4.003 |
| ZINC000000394284 | -4.001 |
| ZINC000003830405 | -3.998 |
| ZINC000003831404 | -3.995 |
| ZINC000003831404 | -3.995 |
| ZINC000003991624 | -3.988 |
| ZINC000003079337 | -3.987 |
| ZINC000035902489 | -3.986 |
| ZINC000100017856 | -3.986 |
| ZINC000100017856 | -3.986 |
| ZINC000000035804 | -3.985 |
| ZINC000084758235 | -3.979 |
| ZINC000087515509 | -3.979 |
| ZINC000000001773 | -3.978 |
| ZINC000000001773 | -3.978 |
| ZINC000000001773 | -3.978 |
| ZINC000003831050 | -3.978 |
| ZINC000003798757 | -3.976 |
| ZINC000000057464 | -3.973 |
| ZINC000003581355 | -3.97 |
| ZINC000001554010 | -3.969 |
| ZINC000003843198 | -3.959 |
| ZINC000072316335 | -3.953 |
| ZINC000022116612 | -3.953 |
| ZINC000072316335 | -3.951 |
| ZINC000038140873 | -3.948 |
| ZINC000001530575 | -3.946 |
| ZINC000000537791 | -3.945 |
| ZINC000100070954 | -3.943 |
| ZINC000000599734 | -3.942 |
| ZINC000003875357 | -3.941 |
| ZINC000000000711 | -3.938 |
| ZINC000019632618 | -3.937 |
| ZINC000019632618 | -3.937 |
| ZINC000019632618 | -3.937 |
| ZINC000001530654 | -3.936 |
| ZINC000001530654 | -3.936 |
| ZINC000043207851 | -3.935 |
| ZINC000003830314 | -3.935 |
| ZINC000000968255 | -3.933 |
| ZINC000000025958 | -3.929 |
| ZINC000003874950 | -3.929 |
| ZINC000003799072 | -3.924 |
| ZINC000003816287 | -3.921 |
| ZINC000011616882 | -3.921 |
| ZINC000035902489 | -3.917 |
| ZINC000000001984 | -3.915 |
| ZINC000000114124 | -3.915 |
| ZINC000095616600 | -3.914 |
| ZINC000085205451 | -3.911 |
| ZINC000011677837 | -3.91 |
| ZINC000000601317 | -3.909 |
| ZINC000001530751 | -3.908 |
| ZINC000001530751 | -3.908 |
| ZINC000096942201 | -3.905 |
| ZINC000014210642 | -3.903 |
| ZINC000014768621 | -3.903 |
| ZINC000004214700 | -3.902 |
| ZINC000004214700 | -3.902 |
| ZINC000001530863 | -3.901 |
| ZINC000001536109 | -3.898 |
| ZINC000019594599 | -3.895 |
| ZINC000019594599 | -3.895 |
| ZINC000019594599 | -3.895 |
| ZINC000003812989 | -3.892 |
| ZINC000003812989 | -3.892 |
| ZINC000003981610 | -3.889 |
| ZINC000014210642 | -3.885 |
| ZINC000001554588 | -3.878 |
| ZINC000003913937 | -3.868 |
| ZINC000000001982 | -3.86 |
| ZINC000004618208 | -3.86 |
| ZINC000094566092 | -3.858 |
| ZINC000000004076 | -3.853 |
| ZINC000001530810 | -3.853 |
| ZINC000001530580 | -3.85 |
| ZINC000003875560 | -3.845 |
| ZINC000000896709 | -3.836 |
| ZINC000001542002 | -3.834 |
| ZINC000100004343 | -3.833 |
| ZINC000019166988 | -3.83 |
| ZINC000003830215 | -3.828 |
| ZINC000001530580 | -3.823 |
| ZINC000001851149 | -3.821 |
| ZINC000001851149 | -3.821 |
| ZINC000011677376 | -3.813 |
| ZINC000011677376 | -3.813 |
| ZINC000011677376 | -3.813 |
| ZINC000000897222 | -3.81 |
| ZINC000003926298 | -3.809 |
| ZINC000004393164 | -3.808 |
| ZINC000003785268 | -3.807 |
| ZINC000000607939 | -3.807 |
| ZINC000001482197 | -3.804 |
| ZINC000001530568 | -3.801 |
| ZINC000003830947 | -3.801 |
| ZINC000003833821 | -3.8 |
| ZINC000022016981 | -3.8 |
| ZINC000022016981 | -3.8 |
| ZINC000022016981 | -3.8 |
| ZINC000001530775 | -3.798 |
| ZINC000003831138 | -3.797 |
| ZINC000001698306 | -3.792 |
| ZINC000253498282 | -3.791 |
| ZINC000003782807 | -3.79 |
| ZINC000034806477 | -3.789 |
| ZINC000000000353 | -3.787 |
| ZINC000003943279 | -3.783 |
| ZINC000000538386 | -3.781 |
| ZINC000000538386 | -3.781 |
| ZINC000012468792 | -3.775 |
| ZINC000003792789 | -3.773 |
| ZINC000049933061 | -3.766 |
| ZINC000003798763 | -3.759 |
| ZINC000095626706 | -3.759 |
| ZINC000012503187 | -3.757 |
| ZINC000000000973 | -3.755 |
| ZINC000095619105 | -3.752 |
| ZINC000095619105 | -3.752 |
| ZINC000095619105 | -3.752 |
| ZINC000003875392 | -3.749 |
| ZINC000003875392 | -3.749 |
| ZINC000003875392 | -3.749 |
| ZINC000003995811 | -3.745 |
| ZINC000001532529 | -3.742 |
| ZINC000000000973 | -3.738 |
| ZINC000001530571 | -3.737 |
| ZINC000001530571 | -3.737 |
| ZINC000014261579 | -3.737 |
| ZINC000004693575 | -3.731 |
| ZINC000000114127 | -3.728 |
| ZINC000049036447 | -3.727 |
| ZINC000008214703 | -3.721 |
| ZINC000012495062 | -3.719 |
| ZINC000002522669 | -3.719 |
| ZINC000002522669 | -3.719 |
| ZINC000004097308 | -3.71 |
| ZINC000009212654 | -3.708 |
| ZINC000000968375 | -3.705 |
| ZINC000005456939 | -3.703 |
| ZINC000003830218 | -3.702 |
| ZINC000003831332 | -3.7 |
| ZINC000003830959 | -3.696 |
| ZINC000003830218 | -3.695 |
| ZINC000000000494 | -3.691 |
| ZINC000000896695 | -3.689 |
| ZINC000000896695 | -3.689 |
| ZINC000100008319 | -3.687 |
| ZINC000003787060 | -3.683 |
| ZINC000003871832 | -3.675 |
| ZINC000003871832 | -3.675 |
| ZINC000000005152 | -3.671 |
| ZINC000004097416 | -3.668 |
| ZINC000242437511 | -3.665 |
| ZINC000008214629 | -3.659 |
| ZINC000002032615 | -3.659 |
| ZINC000008214651 | -3.655 |
| ZINC000098023177 | -3.648 |
| ZINC000098023177 | -3.648 |
| ZINC000019632668 | -3.645 |
| ZINC000019632668 | -3.645 |
| ZINC000095616600 | -3.64 |
| ZINC000000537791 | -3.637 |
| ZINC000003008621 | -3.629 |
| ZINC000003833846 | -3.629 |
| ZINC000003833846 | -3.629 |
| ZINC000003833846 | -3.629 |
| ZINC000000895318 | -3.625 |
| ZINC000070466416 | -3.623 |
| ZINC000012414057 | -3.621 |
| ZINC000003875334 | -3.62 |
| ZINC000019632614 | -3.62 |
| ZINC000019632614 | -3.62 |
| ZINC000000001370 | -3.619 |
| ZINC000068247389 | -3.619 |
| ZINC000068247389 | -3.619 |
| ZINC000019632628 | -3.614 |
| ZINC000019632628 | -3.614 |
| ZINC000000000973 | -3.608 |
| ZINC000001530701 | -3.607 |
| ZINC000002019953 | -3.604 |
| ZINC000000000973 | -3.604 |
| ZINC000002019953 | -3.603 |
| ZINC000003806721 | -3.596 |
| ZINC000003806721 | -3.596 |
| ZINC000001530635 | -3.596 |
| ZINC000034608502 | -3.591 |
| ZINC000100004345 | -3.585 |
| ZINC000004095858 | -3.584 |
| ZINC000011680067 | -3.584 |
| ZINC000003917708 | -3.584 |
| ZINC000000403609 | -3.583 |
| ZINC000000601254 | -3.582 |
| ZINC000100014909 | -3.58 |
| ZINC000100014909 | -3.58 |
| ZINC000000643143 | -3.567 |
| ZINC000001530572 | -3.565 |
| ZINC000003995807 | -3.563 |
| ZINC000000018087 | -3.555 |
| ZINC000095616603 | -3.554 |
| ZINC000052509463 | -3.554 |
| ZINC000000537791 | -3.553 |
| ZINC000000113415 | -3.551 |
| ZINC000001530935 | -3.549 |
| ZINC000013540519 | -3.548 |
| ZINC000003782599 | -3.544 |
| ZINC000000897244 | -3.544 |
| ZINC000000896717 | -3.539 |
| ZINC000034781704 | -3.538 |
| ZINC000100018854 | -3.536 |
| ZINC000100018854 | -3.536 |
| ZINC000100018854 | -3.536 |
| ZINC000000000347 | -3.534 |
| ZINC000000000347 | -3.534 |
| ZINC000000000347 | -3.534 |
| ZINC000053683151 | -3.533 |
| ZINC000053683151 | -3.533 |
| ZINC000053683151 | -3.533 |
| ZINC000022448983 | -3.533 |
| ZINC000022448983 | -3.533 |
| ZINC000022448983 | -3.533 |
| ZINC000000538627 | -3.532 |
| ZINC000003079336 | -3.524 |
| ZINC000004676424 | -3.523 |
| ZINC000003995809 | -3.512 |
| ZINC000000897251 | -3.511 |
| ZINC000000897251 | -3.511 |
| ZINC000003800008 | -3.505 |
| ZINC000003800008 | -3.505 |
| ZINC000022016976 | -3.504 |
| ZINC000022016976 | -3.504 |
| ZINC000022016976 | -3.504 |
| ZINC000001530769 | -3.499 |
| ZINC000001853205 | -3.497 |
| ZINC000000000941 | -3.492 |
| ZINC000001481956 | -3.492 |
| ZINC000001481956 | -3.492 |
| ZINC000001850374 | -3.49 |
| ZINC000001996117 | -3.487 |
| ZINC000001996117 | -3.487 |
| ZINC000000009342 | -3.483 |
| ZINC000000009342 | -3.483 |
| ZINC000001530579 | -3.483 |
| ZINC000000057522 | -3.478 |
| ZINC000003830958 | -3.474 |
| ZINC000001530579 | -3.473 |
| ZINC000003938695 | -3.473 |
| ZINC000100001918 | -3.466 |
| ZINC000003816514 | -3.465 |
| ZINC000003916214 | -3.453 |
| ZINC000003916214 | -3.453 |
| ZINC000003875393 | -3.453 |
| ZINC000003875393 | -3.453 |
| ZINC000003875393 | -3.453 |
| ZINC000019144226 | -3.448 |
| ZINC000004097310 | -3.446 |
| ZINC000000968264 | -3.441 |
| ZINC000000968264 | -3.441 |
| ZINC000001530756 | -3.44 |
| ZINC000100003902 | -3.437 |
| ZINC000100003902 | -3.437 |
| ZINC000003925861 | -3.434 |
| ZINC000000001382 | -3.423 |
| ZINC000000004413 | -3.416 |
| ZINC000000004413 | -3.416 |
| ZINC000100074252 | -3.411 |
| ZINC000000601281 | -3.408 |
| ZINC000000601281 | -3.408 |
| ZINC000000537795 | -3.4 |
| ZINC000000602632 | -3.399 |
| ZINC000004212851 | -3.392 |
| ZINC000043200832 | -3.378 |
| ZINC000043200832 | -3.378 |
| ZINC000043200832 | -3.378 |
| ZINC000068153186 | -3.375 |
| ZINC000003990451 | -3.373 |
| ZINC000001530703 | -3.373 |
| ZINC000068153186 | -3.373 |
| ZINC000017146904 | -3.371 |
| ZINC000001530703 | -3.371 |
| ZINC000003812888 | -3.368 |
| ZINC000003812888 | -3.368 |
| ZINC000001530737 | -3.365 |
| ZINC000001530737 | -3.365 |
| ZINC000001530736 | -3.365 |
| ZINC000001530736 | -3.365 |
| ZINC000001532522 | -3.362 |
| ZINC000006920384 | -3.356 |
| ZINC000003831430 | -3.355 |
| ZINC000148723177 | -3.353 |
| ZINC000148723177 | -3.353 |
| ZINC000003876136 | -3.348 |
| ZINC000003938704 | -3.338 |
| ZINC000001530812 | -3.338 |
| ZINC000001530812 | -3.338 |
| ZINC000001530812 | -3.338 |
| ZINC000003938704 | -3.337 |
| ZINC000000537795 | -3.334 |
| ZINC000003941496 | -3.334 |
| ZINC000095616599 | -3.334 |
| ZINC000003920355 | -3.329 |
| ZINC000000537891 | -3.329 |
| ZINC000000537891 | -3.329 |
| ZINC000022056030 | -3.328 |
| ZINC000022056030 | -3.328 |
| ZINC000022056030 | -3.328 |
| ZINC000003784182 | -3.327 |
| ZINC000000537928 | -3.325 |
| ZINC000000537795 | -3.325 |
| ZINC000003920266 | -3.317 |
| ZINC000003843378 | -3.316 |
| ZINC000000402954 | -3.311 |
| ZINC000000402954 | -3.311 |
| ZINC000000402954 | -3.311 |
| ZINC000000002212 | -3.31 |
| ZINC000003920266 | -3.31 |
| ZINC000001530759 | -3.309 |
| ZINC000003816292 | -3.308 |
| ZINC000004215736 | -3.308 |
| ZINC000004215736 | -3.308 |
| ZINC000000538509 | -3.302 |
| ZINC000000538509 | -3.302 |
| ZINC000000897002 | -3.301 |
| ZINC000000897002 | -3.301 |
| ZINC000000897002 | -3.301 |
| ZINC000004658557 | -3.298 |
| ZINC000043194409 | -3.291 |
| ZINC000019364225 | -3.285 |
| ZINC000003951740 | -3.284 |
| ZINC000013911941 | -3.284 |
| ZINC000094566093 | -3.282 |
| ZINC000019364225 | -3.281 |
| ZINC000001539579 | -3.278 |
| ZINC000000403533 | -3.272 |
| ZINC000000403533 | -3.272 |
| ZINC000000403533 | -3.272 |
| ZINC000004175630 | -3.27 |
| ZINC000004175630 | -3.27 |
| ZINC000004212945 | -3.263 |
| ZINC000026664090 | -3.259 |
| ZINC000026664090 | -3.259 |
| ZINC000026664090 | -3.259 |
| ZINC000043450326 | -3.253 |
| ZINC000043450326 | -3.253 |
| ZINC000043450324 | -3.249 |
| ZINC000043450324 | -3.249 |
| ZINC000000538273 | -3.247 |
| ZINC000000538273 | -3.247 |
| ZINC000003815424 | -3.244 |
| ZINC000003800008 | -3.24 |
| ZINC000011726211 | -3.228 |
| ZINC000011726211 | -3.228 |
| ZINC000019203912 | -3.226 |
| ZINC000019203912 | -3.226 |
| ZINC000019203912 | -3.226 |
| ZINC000000004351 | -3.211 |
| ZINC000000004351 | -3.211 |
| ZINC000019632718 | -3.203 |
| ZINC000011615926 | -3.203 |
| ZINC000003812897 | -3.202 |
| ZINC000000014007 | -3.2 |
| ZINC000003936683 | -3.196 |
| ZINC000003936683 | -3.196 |
| ZINC000003875439 | -3.195 |
| ZINC000003813061 | -3.191 |
| ZINC000019632713 | -3.19 |
| ZINC000000608382 | -3.184 |
| ZINC000000608382 | -3.184 |
| ZINC000030691797 | -3.182 |
| ZINC000038945666 | -3.175 |
| ZINC000003816514 | -3.169 |
| ZINC000013986658 | -3.162 |
| ZINC000019796168 | -3.161 |
| ZINC000019796168 | -3.161 |
| ZINC000019796168 | -3.161 |
| ZINC000000895316 | -3.154 |
| ZINC000000968326 | -3.148 |
| ZINC000000537752 | -3.147 |
| ZINC000000537752 | -3.147 |
| ZINC000000968326 | -3.147 |
| ZINC000001530938 | -3.144 |
| ZINC000001530938 | -3.144 |
| ZINC000011680943 | -3.137 |
| ZINC000011680943 | -3.137 |
| ZINC000095619101 | -3.136 |
| ZINC000095619101 | -3.136 |
| ZINC000086040406 | -3.127 |
| ZINC000003881640 | -3.124 |
| ZINC000003812841 | -3.123 |
| ZINC000096272772 | -3.116 |
| ZINC000005752191 | -3.107 |
| ZINC000008214573 | -3.1 |
| ZINC000001535101 | -3.097 |
| ZINC000001530625 | -3.093 |
| ZINC000049783788 | -3.077 |
| ZINC000095616937 | -3.076 |
| ZINC000049783788 | -3.075 |
| ZINC000001530703 | -3.074 |
| ZINC000049783788 | -3.071 |
| ZINC000004097309 | -3.058 |
| ZINC000001530752 | -3.039 |
| ZINC000001530752 | -3.039 |
| ZINC000013986658 | -3.036 |
| ZINC000003938652 | -3.031 |
| ZINC000100378061 | -3.03 |
| ZINC000100378061 | -3.03 |
| ZINC000100378061 | -3.03 |
| ZINC000100013500 | -3.022 |
| ZINC000100013500 | -3.022 |
| ZINC000003830264 | -3.008 |
| ZINC000000591993 | -3.007 |
| ZINC000013973998 | -3.006 |
| ZINC000014263142 | -3.005 |
| ZINC000026011099 | -2.998 |
| ZINC000026011099 | -2.998 |
| ZINC000003797541 | -2.991 |
| ZINC000004097225 | -2.987 |
| ZINC000003826253 | -2.986 |
| ZINC000003645145 | -2.982 |
| ZINC000004474414 | -2.974 |
| ZINC000036766734 | -2.973 |
| ZINC000100015048 | -2.964 |
| ZINC000085537053 | -2.962 |
| ZINC000096014710 | -2.954 |
| ZINC000030690433 | -2.944 |
| ZINC000030690433 | -2.944 |
| ZINC000030690433 | -2.944 |
| ZINC000003929022 | -2.943 |
| ZINC000001530981 | -2.939 |
| ZINC000001530981 | -2.939 |
| ZINC000001530981 | -2.939 |
| ZINC000116473771 | -2.938 |
| ZINC000000607790 | -2.934 |
| ZINC000003872994 | -2.906 |
| ZINC000030691763 | -2.904 |
| ZINC000003798750 | -2.898 |
| ZINC000001280665 | -2.892 |
| ZINC000001280665 | -2.892 |
| ZINC000001280665 | -2.892 |
| ZINC000008214625 | -2.884 |
| ZINC000003830635 | -2.881 |
| ZINC000003830215 | -2.878 |
| ZINC000003830321 | -2.874 |
| ZINC000003813047 | -2.873 |
| ZINC000004102194 | -2.869 |
| ZINC000001532805 | -2.852 |
| ZINC000003871960 | -2.831 |
| ZINC000043195697 | -2.827 |
| ZINC000043195697 | -2.827 |
| ZINC000043100709 | -2.821 |
| ZINC000000403618 | -2.817 |
| ZINC000008214614 | -2.81 |
| ZINC000000621853 | -2.807 |
| ZINC000003817234 | -2.802 |
| ZINC000003817234 | -2.802 |
| ZINC000068202099 | -2.799 |
| ZINC000100022637 | -2.798 |
| ZINC000100016058 | -2.798 |
| ZINC000003781943 | -2.786 |
| ZINC000000155531 | -2.765 |
| ZINC000000155531 | -2.765 |
| ZINC000036701290 | -2.751 |
| ZINC000036701290 | -2.751 |
| ZINC000000643138 | -2.743 |
| ZINC000242437513 | -2.739 |
| ZINC000005029557 | -2.739 |
| ZINC000001530570 | -2.732 |
| ZINC000003830960 | -2.723 |
| ZINC000019364242 | -2.721 |
| ZINC000019364242 | -2.721 |
| ZINC000000643153 | -2.716 |
| ZINC000245204949 | -2.712 |
| ZINC000003875332 | -2.71 |
| ZINC000003876186 | -2.707 |
| ZINC000100022637 | -2.692 |
| ZINC000100016058 | -2.692 |
| ZINC000018115268 | -2.68 |
| ZINC000018115268 | -2.68 |
| ZINC000001530613 | -2.68 |
| ZINC000001530613 | -2.68 |
| ZINC000242437514 | -2.673 |
| ZINC000038197764 | -2.662 |
| ZINC000038197764 | -2.658 |
| ZINC000100001964 | -2.655 |
| ZINC000019594557 | -2.635 |
| ZINC000019594557 | -2.635 |
| ZINC000001530703 | -2.635 |
| ZINC000000057512 | -2.614 |
| ZINC000242437512 | -2.6 |
| ZINC000000895048 | -2.596 |
| ZINC000000389747 | -2.593 |
| ZINC000000389747 | -2.593 |
| ZINC000000389747 | -2.593 |
| ZINC000004074875 | -2.587 |
| ZINC000004074875 | -2.587 |
| ZINC000000057512 | -2.575 |
| ZINC000003931840 | -2.554 |
| ZINC000003931840 | -2.554 |
| ZINC000003882036 | -2.534 |
| ZINC000000057512 | -2.508 |
| ZINC000003920719 | -2.471 |
| ZINC000094566092 | -2.464 |
| ZINC000000538266 | -2.463 |
| ZINC000001530641 | -2.463 |
| ZINC000001542930 | -2.458 |
| ZINC000022059926 | -2.455 |
| ZINC000011615927 | -2.449 |
| ZINC000018324776 | -2.437 |
| ZINC000018324776 | -2.437 |
| ZINC000018324776 | -2.437 |
| ZINC000003985982 | -2.43 |
| ZINC000004097427 | -2.426 |
| ZINC000004097427 | -2.426 |
| ZINC000004097467 | -2.415 |
| ZINC000003876023 | -2.412 |
| ZINC000006845963 | -2.382 |
| ZINC000000000257 | -2.373 |
| ZINC000003927822 | -2.359 |
| ZINC000003927822 | -2.359 |
| ZINC000043195697 | -2.341 |
| ZINC000043195697 | -2.341 |
| ZINC000043195697 | -2.341 |
| ZINC000016052277 | -2.335 |
| ZINC000000000196 | -2.325 |
| ZINC000000000196 | -2.325 |
| ZINC000004083606 | -2.322 |
| ZINC000003915154 | -2.305 |
| ZINC000001530718 | -2.303 |
| ZINC000085205448 | -2.302 |
| ZINC000001530820 | -2.3 |
| ZINC000018115268 | -2.299 |
| ZINC000018115268 | -2.299 |
| ZINC000003831128 | -2.259 |
| ZINC000000896523 | -2.253 |
| ZINC000001530940 | -2.218 |
| ZINC000001530940 | -2.218 |
| ZINC000002016258 | -2.2 |
| ZINC000052955754 | -2.195 |
| ZINC000052955754 | -2.195 |
| ZINC000052955754 | -2.195 |
| ZINC000003927200 | -2.191 |
| ZINC000004074875 | -2.183 |
| ZINC000004074875 | -2.183 |
| ZINC000003807172 | -2.168 |
| ZINC000016052277 | -2.161 |
| ZINC000016052277 | -2.161 |
| ZINC000003861806 | -2.094 |
| ZINC000017285872 | -2.091 |
| ZINC000008035268 | -2.08 |
| ZINC000003945984 | -2.076 |
| ZINC000004629876 | -2.073 |
| ZINC000002016257 | -2.056 |
| ZINC000011615928 | -2.051 |
| ZINC000100001965 | -2.015 |
| ZINC000001612996 | -2.004 |
| ZINC000017285869 | -2.002 |
| ZINC000004474460 | -1.966 |
| ZINC000003861599 | -1.965 |
| ZINC000004655029 | -1.938 |
| ZINC000004655029 | -1.938 |
| ZINC000001319780 | -1.936 |
| ZINC000001319780 | -1.936 |
| ZINC000000621893 | -1.917 |
| ZINC000000621893 | -1.917 |
| ZINC000001529425 | -1.911 |
| ZINC000003805768 | -1.894 |
| ZINC000003805768 | -1.894 |
| ZINC000019594594 | -1.831 |
| ZINC000019594594 | -1.831 |
| ZINC000001530950 | -1.809 |
| ZINC000001530951 | -1.781 |
| ZINC000003977777 | -1.764 |
| ZINC000001530717 | -1.764 |
| ZINC000014881137 | -1.754 |
| ZINC000003992105 | -1.74 |
| ZINC000001530762 | -1.739 |
| ZINC000001530762 | -1.739 |
| ZINC000022443609 | -1.738 |
| ZINC000008214635 | -1.732 |
| ZINC000004212809 | -1.675 |
| ZINC000012484958 | -1.64 |
| ZINC000008214619 | -1.6 |
| ZINC000001530811 | -1.43 |
| ZINC000001530811 | -1.43 |
| ZINC000004474603 | -1.347 |
| ZINC000000901159 | -1.317 |
| ZINC000003920027 | -1.315 |
| ZINC000001543873 | -1.248 |
| ZINC000001543873 | -1.248 |
| ZINC000003785276 | -1.184 |
| ZINC000019363537 | -1.097 |
| ZINC000019363537 | -1.094 |
| ZINC000096903163 | -1.092 |
| ZINC000001633889 | -1.049 |
| ZINC000001633887 | -0.982 |
| ZINC000019364219 | -0.876 |
| ZINC000019364219 | -0.876 |
| ZINC000000538283 | -0.774 |
| ZINC000000538283 | -0.774 |
| ZINC000003920657 | -0.75 |
| ZINC000001532179 | -0.705 |
| ZINC000001532525 | -0.472 |
| ZINC000001530303 | -0.275 |
| ZINC000001554392 | 0.154 |
| ZINC000001554392 | 0.156 |
| ZINC000004978673 | 0.471 |
| ZINC000001531036 | 0.496 |
| ZINC000006845860 | 0.72 |
| ZINC000000896409 | 1.018 |
| ZINC000000896409 | 1.018 |
| ZINC000008437287 | 1.654 |

**Table S15**. The results of structure-based virtual screening for RHNO1.

| Title | Docking score |
| --- | --- |
| ZINC000001530948 | -9.887 |
| ZINC000005162311 | -9.872 |
| ZINC000035342789 | -9.443 |
| ZINC000001530929 | -9.397 |
| ZINC000001530929 | -9.391 |
| ZINC000001530636 | -9.157 |
| ZINC000003876069 | -9.146 |
| ZINC000003876069 | -9.114 |
| ZINC000001530947 | -9.054 |
| ZINC000100014475 | -9.019 |
| ZINC000000004724 | -8.988 |
| ZINC000000007295 | -8.95 |
| ZINC000003604264 | -8.946 |
| ZINC000000004778 | -8.891 |
| ZINC000003940470 | -8.809 |
| ZINC000013545634 | -8.805 |
| ZINC000013545634 | -8.805 |
| ZINC000000136138 | -8.8 |
| ZINC000000136138 | -8.799 |
| ZINC000001997127 | -8.785 |
| ZINC000013831141 | -8.782 |
| ZINC000003651680 | -8.667 |
| ZINC000003651680 | -8.667 |
| ZINC000000057533 | -8.649 |
| ZINC000000000850 | -8.638 |
| ZINC000013545636 | -8.624 |
| ZINC000013545636 | -8.624 |
| ZINC000002005550 | -8.591 |
| ZINC000002005550 | -8.59 |
| ZINC000000000850 | -8.589 |
| ZINC000000896731 | -8.557 |
| ZINC000000896731 | -8.548 |
| ZINC000003874950 | -8.541 |
| ZINC000000004785 | -8.532 |
| ZINC000003775644 | -8.515 |
| ZINC000003874950 | -8.51 |
| ZINC000000002279 | -8.509 |
| ZINC000000006157 | -8.498 |
| ZINC000001530930 | -8.493 |
| ZINC000001530930 | -8.493 |
| ZINC000013129998 | -8.485 |
| ZINC000000057532 | -8.476 |
| ZINC000004228257 | -8.455 |
| ZINC000000001145 | -8.449 |
| ZINC000013537284 | -8.439 |
| ZINC000004228257 | -8.436 |
| ZINC000000005560 | -8.433 |
| ZINC000100006264 | -8.432 |
| ZINC000000000128 | -8.417 |
| ZINC000000001145 | -8.394 |
| ZINC000000014257 | -8.386 |
| ZINC000000001735 | -8.374 |
| ZINC000012360535 | -8.357 |
| ZINC000001547851 | -8.34 |
| ZINC000000896595 | -8.294 |
| ZINC000035024346 | -8.291 |
| ZINC000013537284 | -8.229 |
| ZINC000003812944 | -8.222 |
| ZINC000051133897 | -8.199 |
| ZINC000009302239 | -8.189 |
| ZINC000000000655 | -8.181 |
| ZINC000013537284 | -8.179 |
| ZINC000000004448 | -8.163 |
| ZINC000001997125 | -8.162 |
| ZINC000000083315 | -8.145 |
| ZINC000000002272 | -8.144 |
| ZINC000000001958 | -8.118 |
| ZINC000000020240 | -8.11 |
| ZINC000006627681 | -8.101 |
| ZINC000003830990 | -8.087 |
| ZINC000000001084 | -8.046 |
| ZINC000003871541 | -8.04 |
| ZINC000000013156 | -8.031 |
| ZINC000000968303 | -8.022 |
| ZINC000000119717 | -8.014 |
| ZINC000000402909 | -8.01 |
| ZINC000001530776 | -8.003 |
| ZINC000003813010 | -8.002 |
| ZINC000003813010 | -7.989 |
| ZINC000003775644 | -7.983 |
| ZINC000000002191 | -7.977 |
| ZINC000000000740 | -7.974 |
| ZINC000000113428 | -7.971 |
| ZINC000000967597 | -7.969 |
| ZINC000003807917 | -7.96 |
| ZINC000000007782 | -7.959 |
| ZINC000000897244 | -7.949 |
| ZINC000003830569 | -7.946 |
| ZINC000001531009 | -7.943 |
| ZINC000001531009 | -7.943 |
| ZINC000001531009 | -7.942 |
| ZINC000000113426 | -7.939 |
| ZINC000000020250 | -7.911 |
| ZINC000000004321 | -7.902 |
| ZINC000000004448 | -7.897 |
| ZINC000001481833 | -7.894 |
| ZINC000003872520 | -7.892 |
| ZINC000000000323 | -7.878 |
| ZINC000000001979 | -7.859 |
| ZINC000018279854 | -7.848 |
| ZINC000001530725 | -7.847 |
| ZINC000000057435 | -7.819 |
| ZINC000000113398 | -7.815 |
| ZINC000000057146 | -7.812 |
| ZINC000007997568 | -7.809 |
| ZINC000007997568 | -7.809 |
| ZINC000000001370 | -7.808 |
| ZINC000003779042 | -7.805 |
| ZINC000012503099 | -7.788 |
| ZINC000012503099 | -7.788 |
| ZINC000013512456 | -7.785 |
| ZINC000013520815 | -7.781 |
| ZINC000000057534 | -7.777 |
| ZINC000003629271 | -7.75 |
| ZINC000002008310 | -7.734 |
| ZINC000002570817 | -7.728 |
| ZINC000000012346 | -7.724 |
| ZINC000018203737 | -7.71 |
| ZINC000018203737 | -7.71 |
| ZINC000000004840 | -7.703 |
| ZINC000000968305 | -7.7 |
| ZINC000000968305 | -7.7 |
| ZINC000000968233 | -7.698 |
| ZINC000002548959 | -7.696 |
| ZINC000000008492 | -7.695 |
| ZINC000000002647 | -7.684 |
| ZINC000035999642 | -7.673 |
| ZINC000000002216 | -7.661 |
| ZINC000021303210 | -7.657 |
| ZINC000000403609 | -7.651 |
| ZINC000018043251 | -7.651 |
| ZINC000006021033 | -7.645 |
| ZINC000035999642 | -7.644 |
| ZINC000000105216 | -7.64 |
| ZINC000100005670 | -7.63 |
| ZINC000001530599 | -7.628 |
| ZINC000095626706 | -7.622 |
| ZINC000003960338 | -7.619 |
| ZINC000000897291 | -7.616 |
| ZINC000001482164 | -7.614 |
| ZINC000000006310 | -7.609 |
| ZINC000000000903 | -7.606 |
| ZINC000000000271 | -7.605 |
| ZINC000000020243 | -7.595 |
| ZINC000000896634 | -7.594 |
| ZINC000003802690 | -7.591 |
| ZINC000000002216 | -7.585 |
| ZINC000000896703 | -7.58 |
| ZINC000000020228 | -7.575 |
| ZINC000000020228 | -7.57 |
| ZINC000000020221 | -7.567 |
| ZINC000000011012 | -7.565 |
| ZINC000021303210 | -7.563 |
| ZINC000000006427 | -7.543 |
| ZINC000019594599 | -7.531 |
| ZINC000019594599 | -7.531 |
| ZINC000019594599 | -7.531 |
| ZINC000008403947 | -7.531 |
| ZINC000000001145 | -7.527 |
| ZINC000000001728 | -7.523 |
| ZINC000000137884 | -7.521 |
| ZINC000018203737 | -7.519 |
| ZINC000018203737 | -7.519 |
| ZINC000000001145 | -7.514 |
| ZINC000000000431 | -7.504 |
| ZINC000000008667 | -7.501 |
| ZINC000000105196 | -7.483 |
| ZINC000000075008 | -7.475 |
| ZINC000000075008 | -7.475 |
| ZINC000000006016 | -7.473 |
| ZINC000000006016 | -7.473 |
| ZINC000018203737 | -7.468 |
| ZINC000018203737 | -7.468 |
| ZINC000000034157 | -7.468 |
| ZINC000000057464 | -7.464 |
| ZINC000000001464 | -7.463 |
| ZINC000000002159 | -7.46 |
| ZINC000000001681 | -7.451 |
| ZINC000000001681 | -7.451 |
| ZINC000009224016 | -7.449 |
| ZINC000009224016 | -7.449 |
| ZINC000000968345 | -7.443 |
| ZINC000001530599 | -7.441 |
| ZINC000000968345 | -7.441 |
| ZINC000001530599 | -7.439 |
| ZINC000001482184 | -7.426 |
| ZINC000013512456 | -7.426 |
| ZINC000013597823 | -7.425 |
| ZINC000013597823 | -7.42 |
| ZINC000001482049 | -7.403 |
| ZINC000001482049 | -7.4 |
| ZINC000000057512 | -7.399 |
| ZINC000000014864 | -7.394 |
| ZINC000000967520 | -7.388 |
| ZINC000000001132 | -7.383 |
| ZINC000000509440 | -7.37 |
| ZINC000000057147 | -7.369 |
| ZINC000008855117 | -7.366 |
| ZINC000008855117 | -7.361 |
| ZINC000001999487 | -7.356 |
| ZINC000001530625 | -7.355 |
| ZINC000000007673 | -7.355 |
| ZINC000000002055 | -7.354 |
| ZINC000000002055 | -7.354 |
| ZINC000004640636 | -7.351 |
| ZINC000003842753 | -7.35 |
| ZINC000004640636 | -7.347 |
| ZINC000000056427 | -7.343 |
| ZINC000005819214 | -7.341 |
| ZINC000003842753 | -7.341 |
| ZINC000000000061 | -7.337 |
| ZINC000006036847 | -7.335 |
| ZINC000003979899 | -7.318 |
| ZINC000000018635 | -7.318 |
| ZINC000034220093 | -7.316 |
| ZINC000000395010 | -7.311 |
| ZINC000003823492 | -7.31 |
| ZINC000003831429 | -7.305 |
| ZINC000013537284 | -7.295 |
| ZINC000000967521 | -7.293 |
| ZINC000012503076 | -7.291 |
| ZINC000004632106 | -7.288 |
| ZINC000004632106 | -7.288 |
| ZINC000000085733 | -7.282 |
| ZINC000000000215 | -7.274 |
| ZINC000000000215 | -7.274 |
| ZINC000006409735 | -7.26 |
| ZINC000006409735 | -7.257 |
| ZINC000000968273 | -7.246 |
| ZINC000100070937 | -7.239 |
| ZINC000003831430 | -7.233 |
| ZINC000004340269 | -7.229 |
| ZINC000006733300 | -7.224 |
| ZINC000000057464 | -7.221 |
| ZINC000118912393 | -7.22 |
| ZINC000003874498 | -7.218 |
| ZINC000036294079 | -7.217 |
| ZINC000004340269 | -7.217 |
| ZINC000036294079 | -7.217 |
| ZINC000084843283 | -7.214 |
| ZINC000004632106 | -7.213 |
| ZINC000004632106 | -7.21 |
| ZINC000002847375 | -7.194 |
| ZINC000002847375 | -7.194 |
| ZINC000012360535 | -7.176 |
| ZINC000000020231 | -7.175 |
| ZINC000013597823 | -7.172 |
| ZINC000004216238 | -7.17 |
| ZINC000000596881 | -7.166 |
| ZINC000000004949 | -7.164 |
| ZINC000013597823 | -7.161 |
| ZINC000000896484 | -7.161 |
| ZINC000000896484 | -7.161 |
| ZINC000000968274 | -7.149 |
| ZINC000000000122 | -7.146 |
| ZINC000000000122 | -7.146 |
| ZINC000000006481 | -7.141 |
| ZINC000016929327 | -7.14 |
| ZINC000001530803 | -7.13 |
| ZINC000013585233 | -7.13 |
| ZINC000003872738 | -7.129 |
| ZINC000000006156 | -7.12 |
| ZINC000000120286 | -7.118 |
| ZINC000000057206 | -7.111 |
| ZINC000000057253 | -7.11 |
| ZINC000000057253 | -7.11 |
| ZINC000002015928 | -7.093 |
| ZINC000000057001 | -7.093 |
| ZINC000001530710 | -7.092 |
| ZINC000000002281 | -7.09 |
| ZINC000003818726 | -7.089 |
| ZINC000003818726 | -7.087 |
| ZINC000000049153 | -7.087 |
| ZINC000000057206 | -7.084 |
| ZINC000100032379 | -7.083 |
| ZINC000000001341 | -7.083 |
| ZINC000100032379 | -7.078 |
| ZINC000000002212 | -7.073 |
| ZINC000000001382 | -7.069 |
| ZINC000003861768 | -7.067 |
| ZINC000003876068 | -7.066 |
| ZINC000000897322 | -7.056 |
| ZINC000003876068 | -7.056 |
| ZINC000003798064 | -7.05 |
| ZINC000000004009 | -7.049 |
| ZINC000003929508 | -7.047 |
| ZINC000000002273 | -7.045 |
| ZINC000003795098 | -7.042 |
| ZINC000000001281 | -7.041 |
| ZINC000022002214 | -7.038 |
| ZINC000022002214 | -7.038 |
| ZINC000022002218 | -7.028 |
| ZINC000022002218 | -7.028 |
| ZINC000006467621 | -7.013 |
| ZINC000000967566 | -7.013 |
| ZINC000000056556 | -7.012 |
| ZINC000019702309 | -7.011 |
| ZINC000000074836 | -7.006 |
| ZINC000000057341 | -6.981 |
| ZINC000000001341 | -6.98 |
| ZINC000000001644 | -6.98 |
| ZINC000012360535 | -6.979 |
| ZINC000002522648 | -6.978 |
| ZINC000000895302 | -6.975 |
| ZINC000000056652 | -6.975 |
| ZINC000000057512 | -6.975 |
| ZINC000000057340 | -6.972 |
| ZINC000000009073 | -6.958 |
| ZINC000000009073 | -6.958 |
| ZINC000000009073 | -6.958 |
| ZINC000000010164 | -6.956 |
| ZINC000000010164 | -6.956 |
| ZINC000003872277 | -6.947 |
| ZINC000000003642 | -6.944 |
| ZINC000001530621 | -6.944 |
| ZINC000001530912 | -6.941 |
| ZINC000001530912 | -6.941 |
| ZINC000001530912 | -6.941 |
| ZINC000002510358 | -6.941 |
| ZINC000000020244 | -6.94 |
| ZINC000000020244 | -6.94 |
| ZINC000002510358 | -6.935 |
| ZINC000001530621 | -6.928 |
| ZINC000001530806 | -6.925 |
| ZINC000000002043 | -6.925 |
| ZINC000000002043 | -6.925 |
| ZINC000000596881 | -6.915 |
| ZINC000000508068 | -6.914 |
| ZINC000000000746 | -6.914 |
| ZINC000000057254 | -6.906 |
| ZINC000000057254 | -6.906 |
| ZINC000000001984 | -6.901 |
| ZINC000000001655 | -6.898 |
| ZINC000008855117 | -6.892 |
| ZINC000008855117 | -6.892 |
| ZINC000003830347 | -6.891 |
| ZINC000003830347 | -6.891 |
| ZINC000000896666 | -6.887 |
| ZINC000003798247 | -6.879 |
| ZINC000001530805 | -6.876 |
| ZINC000000013156 | -6.873 |
| ZINC000000121541 | -6.861 |
| ZINC000100071256 | -6.86 |
| ZINC000000009689 | -6.859 |
| ZINC000000010163 | -6.85 |
| ZINC000000010163 | -6.85 |
| ZINC000004097225 | -6.849 |
| ZINC000000000490 | -6.845 |
| ZINC000006661227 | -6.832 |
| ZINC000006661227 | -6.832 |
| ZINC000006661227 | -6.831 |
| ZINC000000388462 | -6.83 |
| ZINC000087515509 | -6.828 |
| ZINC000004392649 | -6.818 |
| ZINC000101489663 | -6.812 |
| ZINC000000020231 | -6.81 |
| ZINC000019875504 | -6.804 |
| ZINC000019875504 | -6.803 |
| ZINC000100009280 | -6.803 |
| ZINC000100009280 | -6.803 |
| ZINC000000039089 | -6.801 |
| ZINC000000113355 | -6.8 |
| ZINC000000113382 | -6.798 |
| ZINC000001035331 | -6.794 |
| ZINC000000968263 | -6.789 |
| ZINC000000001011 | -6.789 |
| ZINC000000057313 | -6.788 |
| ZINC000000057512 | -6.771 |
| ZINC000019166988 | -6.769 |
| ZINC000004658290 | -6.764 |
| ZINC000000000471 | -6.762 |
| ZINC000000113382 | -6.761 |
| ZINC000000266964 | -6.754 |
| ZINC000000020259 | -6.751 |
| ZINC000022010387 | -6.744 |
| ZINC000022010387 | -6.744 |
| ZINC000022010387 | -6.744 |
| ZINC000001576892 | -6.742 |
| ZINC000000005878 | -6.741 |
| ZINC000022010375 | -6.739 |
| ZINC000022010375 | -6.739 |
| ZINC000022010375 | -6.739 |
| ZINC000003787060 | -6.737 |
| ZINC000017146904 | -6.732 |
| ZINC000000002041 | -6.73 |
| ZINC000022010379 | -6.725 |
| ZINC000022010379 | -6.725 |
| ZINC000022010379 | -6.725 |
| ZINC000043763856 | -6.723 |
| ZINC000000896709 | -6.721 |
| ZINC000006627681 | -6.718 |
| ZINC000000056646 | -6.716 |
| ZINC000000001982 | -6.716 |
| ZINC000000020255 | -6.716 |
| ZINC000003875368 | -6.716 |
| ZINC000003875368 | -6.716 |
| ZINC000100009278 | -6.7 |
| ZINC000100009278 | -6.7 |
| ZINC000000020230 | -6.695 |
| ZINC000003781664 | -6.689 |
| ZINC000003830339 | -6.688 |
| ZINC000000020241 | -6.686 |
| ZINC000000156395 | -6.681 |
| ZINC000019168887 | -6.681 |
| ZINC000004658290 | -6.679 |
| ZINC000000020783 | -6.673 |
| ZINC000019702309 | -6.669 |
| ZINC000000896711 | -6.667 |
| ZINC000022010382 | -6.661 |
| ZINC000022010382 | -6.661 |
| ZINC000022010382 | -6.661 |
| ZINC000000000053 | -6.659 |
| ZINC000000518554 | -6.648 |
| ZINC000019166991 | -6.644 |
| ZINC000100299039 | -6.641 |
| ZINC000000057624 | -6.64 |
| ZINC000013585233 | -6.637 |
| ZINC000000895154 | -6.634 |
| ZINC000000896698 | -6.631 |
| ZINC000000896698 | -6.631 |
| ZINC000000001706 | -6.629 |
| ZINC000001530701 | -6.627 |
| ZINC000004258316 | -6.619 |
| ZINC000000000565 | -6.609 |
| ZINC000000000565 | -6.609 |
| ZINC000000005895 | -6.601 |
| ZINC000000056653 | -6.597 |
| ZINC000084589076 | -6.591 |
| ZINC000008214651 | -6.59 |
| ZINC000001530810 | -6.587 |
| ZINC000034051848 | -6.587 |
| ZINC000034051848 | -6.583 |
| ZINC000000002028 | -6.582 |
| ZINC000013585233 | -6.58 |
| ZINC000003201907 | -6.578 |
| ZINC000003201907 | -6.578 |
| ZINC000000895199 | -6.572 |
| ZINC000000056568 | -6.569 |
| ZINC000006094354 | -6.56 |
| ZINC000006094354 | -6.56 |
| ZINC000006021043 | -6.558 |
| ZINC000003803652 | -6.557 |
| ZINC000000000575 | -6.556 |
| ZINC000000056645 | -6.545 |
| ZINC000000000373 | -6.542 |
| ZINC000003830218 | -6.538 |
| ZINC000001482197 | -6.537 |
| ZINC000001530977 | -6.529 |
| ZINC000001530977 | -6.529 |
| ZINC000001530977 | -6.529 |
| ZINC000000014037 | -6.526 |
| ZINC000038140873 | -6.516 |
| ZINC000096006009 | -6.509 |
| ZINC000096006009 | -6.509 |
| ZINC000003972949 | -6.499 |
| ZINC000003972949 | -6.499 |
| ZINC000000643055 | -6.499 |
| ZINC000000000507 | -6.498 |
| ZINC000000000507 | -6.497 |
| ZINC000000097996 | -6.496 |
| ZINC000000643055 | -6.491 |
| ZINC000000896569 | -6.489 |
| ZINC000001690604 | -6.486 |
| ZINC000005179119 | -6.485 |
| ZINC000013298313 | -6.48 |
| ZINC000003830218 | -6.48 |
| ZINC000000000693 | -6.479 |
| ZINC000002539827 | -6.477 |
| ZINC000003956788 | -6.472 |
| ZINC000000896455 | -6.471 |
| ZINC000000896918 | -6.467 |
| ZINC000002169830 | -6.465 |
| ZINC000001530741 | -6.463 |
| ZINC000000001267 | -6.459 |
| ZINC000000001931 | -6.458 |
| ZINC000000001931 | -6.458 |
| ZINC000002539827 | -6.444 |
| ZINC000004228258 | -6.436 |
| ZINC000004228258 | -6.436 |
| ZINC000004228258 | -6.433 |
| ZINC000003830999 | -6.432 |
| ZINC000008466459 | -6.431 |
| ZINC000002015035 | -6.419 |
| ZINC000002015035 | -6.419 |
| ZINC000100007011 | -6.409 |
| ZINC000013550868 | -6.407 |
| ZINC000053084692 | -6.404 |
| ZINC000000968256 | -6.404 |
| ZINC000011680943 | -6.4 |
| ZINC000000113442 | -6.397 |
| ZINC000000020253 | -6.393 |
| ZINC000003794711 | -6.384 |
| ZINC000003794711 | -6.384 |
| ZINC000003794711 | -6.384 |
| ZINC000004258316 | -6.38 |
| ZINC000000089763 | -6.378 |
| ZINC000003830999 | -6.376 |
| ZINC000000018087 | -6.374 |
| ZINC000000089763 | -6.369 |
| ZINC000001482094 | -6.365 |
| ZINC000001481910 | -6.365 |
| ZINC000000001758 | -6.362 |
| ZINC000001842633 | -6.358 |
| ZINC000000000711 | -6.356 |
| ZINC000000895154 | -6.345 |
| ZINC000000015515 | -6.33 |
| ZINC000001187543 | -6.33 |
| ZINC000053084692 | -6.323 |
| ZINC000000120319 | -6.322 |
| ZINC000019632912 | -6.321 |
| ZINC000000120319 | -6.321 |
| ZINC000052971887 | -6.314 |
| ZINC000013298313 | -6.307 |
| ZINC000000020257 | -6.304 |
| ZINC000003812933 | -6.299 |
| ZINC000052971887 | -6.296 |
| ZINC000003812865 | -6.288 |
| ZINC000002539827 | -6.286 |
| ZINC000001639567 | -6.286 |
| ZINC000000896663 | -6.28 |
| ZINC000053022902 | -6.272 |
| ZINC000053022902 | -6.272 |
| ZINC000001533877 | -6.271 |
| ZINC000001533877 | -6.271 |
| ZINC000001533877 | -6.271 |
| ZINC000001533877 | -6.271 |
| ZINC000000001795 | -6.267 |
| ZINC000012503151 | -6.264 |
| ZINC000253917094 | -6.264 |
| ZINC000000000506 | -6.254 |
| ZINC000001533877 | -6.252 |
| ZINC000001533877 | -6.252 |
| ZINC000001530703 | -6.242 |
| ZINC000011680943 | -6.239 |
| ZINC000000033882 | -6.238 |
| ZINC000000001408 | -6.237 |
| ZINC000000001411 | -6.233 |
| ZINC000000057062 | -6.224 |
| ZINC000001849548 | -6.223 |
| ZINC000000002101 | -6.216 |
| ZINC000038212689 | -6.211 |
| ZINC000038212689 | -6.211 |
| ZINC000000000905 | -6.198 |
| ZINC000000009342 | -6.194 |
| ZINC000000009342 | -6.194 |
| ZINC000000000922 | -6.193 |
| ZINC000003784120 | -6.192 |
| ZINC000001530555 | -6.19 |
| ZINC000001530637 | -6.189 |
| ZINC000006382803 | -6.189 |
| ZINC000003806262 | -6.176 |
| ZINC000003806262 | -6.176 |
| ZINC000000896546 | -6.169 |
| ZINC000000896546 | -6.169 |
| ZINC000013973998 | -6.157 |
| ZINC000000014360 | -6.157 |
| ZINC000000154964 | -6.156 |
| ZINC000000154964 | -6.156 |
| ZINC000000154964 | -6.156 |
| ZINC000000002176 | -6.152 |
| ZINC000003872605 | -6.148 |
| ZINC000003872605 | -6.144 |
| ZINC000004215736 | -6.141 |
| ZINC000004215736 | -6.141 |
| ZINC000000004166 | -6.135 |
| ZINC000001883067 | -6.115 |
| ZINC000000388081 | -6.114 |
| ZINC000000388081 | -6.114 |
| ZINC000000388081 | -6.114 |
| ZINC000000000607 | -6.114 |
| ZINC000002539702 | -6.098 |
| ZINC000012358719 | -6.097 |
| ZINC000000394284 | -6.096 |
| ZINC000000608101 | -6.083 |
| ZINC000000000353 | -6.077 |
| ZINC000003871701 | -6.076 |
| ZINC000001843047 | -6.075 |
| ZINC000000896463 | -6.06 |
| ZINC000000155269 | -6.053 |
| ZINC000000155269 | -6.053 |
| ZINC000000000853 | -6.045 |
| ZINC000000006251 | -6.044 |
| ZINC000000006251 | -6.044 |
| ZINC000000005823 | -6.039 |
| ZINC000006382803 | -6.033 |
| ZINC000002525885 | -6.032 |
| ZINC000002525885 | -6.032 |
| ZINC000000001688 | -6.031 |
| ZINC000000001505 | -6.03 |
| ZINC000000001688 | -6.03 |
| ZINC000000002005 | -6.03 |
| ZINC000000004076 | -6.028 |
| ZINC000000114124 | -6.027 |
| ZINC000006661227 | -6.016 |
| ZINC000000001792 | -6.006 |
| ZINC000095452610 | -5.991 |
| ZINC000000968330 | -5.985 |
| ZINC000003872055 | -5.975 |
| ZINC000003813042 | -5.973 |
| ZINC000000388081 | -5.972 |
| ZINC000000000469 | -5.971 |
| ZINC000000388081 | -5.969 |
| ZINC000000388081 | -5.968 |
| ZINC000000057512 | -5.957 |
| ZINC000000897002 | -5.953 |
| ZINC000000897002 | -5.953 |
| ZINC000000897002 | -5.953 |
| ZINC000004228257 | -5.951 |
| ZINC000000895360 | -5.946 |
| ZINC000000020237 | -5.946 |
| ZINC000000020237 | -5.946 |
| ZINC000034676245 | -5.937 |
| ZINC000034676245 | -5.937 |
| ZINC000000000882 | -5.926 |
| ZINC000000000882 | -5.926 |
| ZINC000017146904 | -5.924 |
| ZINC000005133329 | -5.908 |
| ZINC000000391812 | -5.908 |
| ZINC000000391812 | -5.908 |
| ZINC000000391812 | -5.908 |
| ZINC000006661227 | -5.892 |
| ZINC000006661227 | -5.892 |
| ZINC000000016154 | -5.885 |
| ZINC000004658552 | -5.88 |
| ZINC000000049154 | -5.879 |
| ZINC000000004319 | -5.866 |
| ZINC000000004319 | -5.863 |
| ZINC000001698306 | -5.861 |
| ZINC000000113446 | -5.858 |
| ZINC000000001554 | -5.847 |
| ZINC000001530816 | -5.843 |
| ZINC000000896958 | -5.839 |
| ZINC000000035804 | -5.823 |
| ZINC000001530816 | -5.818 |
| ZINC000000000494 | -5.814 |
| ZINC000000119344 | -5.796 |
| ZINC000000001798 | -5.79 |
| ZINC000000001798 | -5.79 |
| ZINC000000001798 | -5.79 |
| ZINC000022056030 | -5.788 |
| ZINC000022056030 | -5.788 |
| ZINC000022056030 | -5.788 |
| ZINC000019632633 | -5.781 |
| ZINC000019632633 | -5.781 |
| ZINC000019632633 | -5.781 |
| ZINC000000895154 | -5.774 |
| ZINC000008015016 | -5.773 |
| ZINC000000006226 | -5.771 |
| ZINC000004097392 | -5.764 |
| ZINC000001530599 | -5.764 |
| ZINC000001543475 | -5.762 |
| ZINC000001543475 | -5.762 |
| ZINC000001543475 | -5.762 |
| ZINC000002561203 | -5.729 |
| ZINC000001530737 | -5.726 |
| ZINC000001530737 | -5.726 |
| ZINC000001530736 | -5.726 |
| ZINC000001530736 | -5.726 |
| ZINC000052957434 | -5.725 |
| ZINC000052957434 | -5.725 |
| ZINC000003927870 | -5.695 |
| ZINC000003927870 | -5.695 |
| ZINC000012414057 | -5.694 |
| ZINC000000002009 | -5.682 |
| ZINC000000403079 | -5.675 |
| ZINC000018099446 | -5.674 |
| ZINC000100019007 | -5.674 |
| ZINC000000901791 | -5.671 |
| ZINC000000403079 | -5.67 |
| ZINC000001530863 | -5.662 |
| ZINC000000004413 | -5.656 |
| ZINC000000004413 | -5.656 |
| ZINC000000000905 | -5.624 |
| ZINC000000000456 | -5.624 |
| ZINC000000000456 | -5.624 |
| ZINC000000000456 | -5.624 |
| ZINC000000001899 | -5.623 |
| ZINC000000000882 | -5.62 |
| ZINC000005133378 | -5.616 |
| ZINC000000000882 | -5.614 |
| ZINC000003927870 | -5.609 |
| ZINC000001530756 | -5.607 |
| ZINC000002016257 | -5.605 |
| ZINC000003589203 | -5.6 |
| ZINC000100006770 | -5.594 |
| ZINC000000005152 | -5.592 |
| ZINC000000895099 | -5.589 |
| ZINC000000000242 | -5.588 |
| ZINC000000000242 | -5.588 |
| ZINC000001530716 | -5.588 |
| ZINC000003645145 | -5.578 |
| ZINC000000000196 | -5.578 |
| ZINC000000000196 | -5.578 |
| ZINC000003581355 | -5.562 |
| ZINC000001530862 | -5.555 |
| ZINC000004097426 | -5.549 |
| ZINC000004097426 | -5.549 |
| ZINC000004097426 | -5.549 |
| ZINC000000155531 | -5.547 |
| ZINC000000155531 | -5.547 |
| ZINC000000001115 | -5.547 |
| ZINC000000114127 | -5.539 |
| ZINC000000057255 | -5.537 |
| ZINC000003831475 | -5.534 |
| ZINC000001530812 | -5.529 |
| ZINC000001530812 | -5.529 |
| ZINC000001530812 | -5.529 |
| ZINC000001530817 | -5.51 |
| ZINC000095452610 | -5.506 |
| ZINC000001530817 | -5.505 |
| ZINC000001690324 | -5.486 |
| ZINC000001690324 | -5.486 |
| ZINC000003831282 | -5.448 |
| ZINC000003831474 | -5.435 |
| ZINC000007997952 | -5.434 |
| ZINC000007997952 | -5.434 |
| ZINC000002019954 | -5.434 |
| ZINC000001532529 | -5.429 |
| ZINC000002019954 | -5.423 |
| ZINC000003801919 | -5.422 |
| ZINC000001532526 | -5.417 |
| ZINC000000113410 | -5.41 |
| ZINC000003801919 | -5.406 |
| ZINC000003801919 | -5.406 |
| ZINC000001530703 | -5.399 |
| ZINC000001530703 | -5.397 |
| ZINC000008034120 | -5.394 |
| ZINC000000057513 | -5.392 |
| ZINC000000057513 | -5.392 |
| ZINC000095616603 | -5.36 |
| ZINC000004577910 | -5.357 |
| ZINC000019144216 | -5.336 |
| ZINC000019144216 | -5.336 |
| ZINC000000896819 | -5.325 |
| ZINC000006827695 | -5.321 |
| ZINC000006827695 | -5.321 |
| ZINC000006827695 | -5.321 |
| ZINC000008034121 | -5.319 |
| ZINC000000003911 | -5.313 |
| ZINC000000007601 | -5.302 |
| ZINC000000049154 | -5.289 |
| ZINC000001530938 | -5.281 |
| ZINC000001530938 | -5.281 |
| ZINC000100061056 | -5.276 |
| ZINC000000895081 | -5.245 |
| ZINC000000000083 | -5.244 |
| ZINC000003079342 | -5.238 |
| ZINC000000121480 | -5.221 |
| ZINC000001530638 | -5.218 |
| ZINC000004658553 | -5.2 |
| ZINC000000968328 | -5.194 |
| ZINC000000895457 | -5.191 |
| ZINC000003812862 | -5.183 |
| ZINC000003812862 | -5.183 |
| ZINC000003812862 | -5.183 |
| ZINC000001530427 | -5.175 |
| ZINC000001530427 | -5.175 |
| ZINC000001530427 | -5.175 |
| ZINC000000897288 | -5.173 |
| ZINC000003830813 | -5.165 |
| ZINC000003830813 | -5.165 |
| ZINC000003830813 | -5.165 |
| ZINC000100037020 | -5.164 |
| ZINC000100037020 | -5.164 |
| ZINC000100037020 | -5.164 |
| ZINC000012503156 | -5.162 |
| ZINC000000599985 | -5.142 |
| ZINC000003831477 | -5.13 |
| ZINC000034676245 | -5.113 |
| ZINC000003831551 | -5.107 |
| ZINC000003079340 | -5.098 |
| ZINC000004658603 | -5.096 |
| ZINC000004658603 | -5.096 |
| ZINC000000020251 | -5.093 |
| ZINC000001532728 | -5.077 |
| ZINC000086040406 | -5.076 |
| ZINC000018089317 | -5.052 |
| ZINC000003008621 | -5.05 |
| ZINC000000155905 | -5.047 |
| ZINC000001554588 | -5.044 |
| ZINC000001530572 | -5.042 |
| ZINC000003831050 | -5.036 |
| ZINC000000901736 | -5.032 |
| ZINC000001531008 | -5.027 |
| ZINC000003830314 | -5.026 |
| ZINC000001530600 | -5.014 |
| ZINC000003776875 | -4.996 |
| ZINC000003830891 | -4.992 |
| ZINC000008214514 | -4.982 |
| ZINC000001554010 | -4.975 |
| ZINC000000599985 | -4.963 |
| ZINC000001530636 | -4.954 |
| ZINC000000895032 | -4.929 |
| ZINC000001530283 | -4.928 |
| ZINC000001532517 | -4.92 |
| ZINC000000014007 | -4.897 |
| ZINC000003830500 | -4.864 |
| ZINC000003830500 | -4.86 |
| ZINC000100009383 | -4.853 |
| ZINC000008015016 | -4.852 |
| ZINC000008015016 | -4.852 |
| ZINC000008101126 | -4.84 |
| ZINC000001532522 | -4.815 |
| ZINC000001846431 | -4.768 |
| ZINC000008101109 | -4.754 |
| ZINC000008101109 | -4.754 |
| ZINC000008101109 | -4.754 |
| ZINC000001530600 | -4.719 |
| ZINC000001530600 | -4.719 |
| ZINC000005224188 | -4.696 |
| ZINC000002019953 | -4.684 |
| ZINC000012859773 | -4.657 |
| ZINC000000968301 | -4.652 |
| ZINC000003843378 | -4.649 |
| ZINC000006827693 | -4.637 |
| ZINC000006827693 | -4.637 |
| ZINC000000895042 | -4.62 |
| ZINC000003831040 | -4.606 |
| ZINC000001530703 | -4.602 |
| ZINC000002019953 | -4.595 |
| ZINC000001530641 | -4.526 |
| ZINC000000901555 | -4.507 |
| ZINC000000000257 | -4.44 |
| ZINC000003831051 | -4.406 |
| ZINC000001530713 | -4.398 |
| ZINC000001530713 | -4.39 |
| ZINC000001530950 | -4.383 |
| ZINC000000895103 | -4.344 |
| ZINC000003079336 | -4.344 |
| ZINC000000901552 | -4.295 |
| ZINC000018115268 | -4.29 |
| ZINC000018115268 | -4.29 |
| ZINC000003079337 | -4.244 |
| ZINC000002016258 | -4.24 |
| ZINC000003782550 | -4.147 |
| ZINC000003782550 | -4.146 |
| ZINC000019364225 | -4.138 |
| ZINC000019364225 | -4.125 |
| ZINC000003809490 | -4.038 |
| ZINC000000000346 | -4.019 |
| ZINC000001530940 | -3.987 |
| ZINC000001530940 | -3.987 |
| ZINC000001543873 | -3.967 |
| ZINC000001543873 | -3.967 |
| ZINC000018115268 | -3.799 |
| ZINC000018115268 | -3.799 |
| ZINC000000901061 | -3.796 |
| ZINC000004099200 | -3.779 |
| ZINC000001530811 | -3.768 |
| ZINC000001530811 | -3.768 |
| ZINC000001529425 | -3.742 |
| ZINC000004658560 | -3.726 |
| ZINC000004658562 | -3.691 |
| ZINC000002000707 | -3.684 |
| ZINC000001482113 | -3.615 |
| ZINC000003798750 | -3.59 |
| ZINC000004676424 | -3.515 |
| ZINC000000896523 | -3.398 |
| ZINC000000895034 | -3.174 |
| ZINC000000895034 | -3.172 |
| ZINC000004658557 | -3.145 |
| ZINC000008214573 | -3.026 |
| ZINC000000896695 | -2.975 |
| ZINC000000896695 | -2.969 |
| ZINC000001530717 | -2.954 |
| ZINC000000895316 | -2.869 |
| ZINC000000895318 | -2.869 |
| ZINC000000113415 | -2.848 |
| ZINC000008214625 | -2.811 |
| ZINC000000403618 | -2.79 |
| ZINC000001530303 | -2.643 |
| ZINC000003830961 | -2.638 |
| ZINC000001532805 | -2.508 |
| ZINC000019364242 | -2.433 |
| ZINC000019364242 | -2.433 |
| ZINC000001633887 | -2.255 |
| ZINC000001530820 | -2.249 |
| ZINC000000895048 | -2.202 |
| ZINC000019364219 | -2.168 |
| ZINC000019364219 | -2.163 |
| ZINC000034781704 | -2.149 |
| ZINC000001530718 | -2.046 |
| ZINC000000901159 | -1.991 |
| ZINC000001530951 | -1.966 |
| ZINC000001554392 | -1.584 |
| ZINC000001554392 | -1.565 |
| ZINC000017285872 | -1.388 |
| ZINC000017285869 | -1.179 |
| ZINC000001532525 | -1.078 |
| ZINC000000896968 | -0.978 |
| ZINC000001531036 | -0.877 |
| ZINC000019363537 | -0.438 |
| ZINC000019363537 | -0.432 |
| ZINC000004097476 | -0.111 |
| ZINC000000896409 | 1.473 |
| ZINC000000896409 | 1.473 |

**Table S16**. The results of structure-based virtual screening for SCD.

| Title | Docking score |
| --- | --- |
| ZINC000003830947 | -6.416 |
| ZINC000085540219 | -6.39 |
| ZINC000003802690 | -6.243 |
| ZINC000003830958 | -5.925 |
| ZINC000085537017 | -5.904 |
| ZINC000085537017 | -5.904 |
| ZINC000085537017 | -5.893 |
| ZINC000009164421 | -5.792 |
| ZINC000013585233 | -5.774 |
| ZINC000085540223 | -5.771 |
| ZINC000013585233 | -5.753 |
| ZINC000006827695 | -5.564 |
| ZINC000006827695 | -5.564 |
| ZINC000006827695 | -5.564 |
| ZINC000013585233 | -5.547 |
| ZINC000001530636 | -5.492 |
| ZINC000085540215 | -5.492 |
| ZINC000008214418 | -5.47 |
| ZINC000009212427 | -5.424 |
| ZINC000009212427 | -5.422 |
| ZINC000000895154 | -5.397 |
| ZINC000006382803 | -5.392 |
| ZINC000003830441 | -5.389 |
| ZINC000004228257 | -5.378 |
| ZINC000004228257 | -5.356 |
| ZINC000004228257 | -5.347 |
| ZINC000003927870 | -5.337 |
| ZINC000003927870 | -5.337 |
| ZINC000003830813 | -5.333 |
| ZINC000003939013 | -5.33 |
| ZINC000003830813 | -5.325 |
| ZINC000003830813 | -5.325 |
| ZINC000003927870 | -5.321 |
| ZINC000022016976 | -5.304 |
| ZINC000022016976 | -5.304 |
| ZINC000022016976 | -5.304 |
| ZINC000000001505 | -5.251 |
| ZINC000001531009 | -5.24 |
| ZINC000001531009 | -5.24 |
| ZINC000001531009 | -5.239 |
| ZINC000014879992 | -5.227 |
| ZINC000001543475 | -5.226 |
| ZINC000001543475 | -5.226 |
| ZINC000001543475 | -5.226 |
| ZINC000003922770 | -5.209 |
| ZINC000000895154 | -5.204 |
| ZINC000003861768 | -5.202 |
| ZINC000034676245 | -5.197 |
| ZINC000034676245 | -5.197 |
| ZINC000000895154 | -5.197 |
| ZINC000008015016 | -5.18 |
| ZINC000008015016 | -5.18 |
| ZINC000008855117 | -5.151 |
| ZINC000003812862 | -5.134 |
| ZINC000003812862 | -5.134 |
| ZINC000002539827 | -5.131 |
| ZINC000002169830 | -5.122 |
| ZINC000008855117 | -5.121 |
| ZINC000005733652 | -5.121 |
| ZINC000005733652 | -5.121 |
| ZINC000005733652 | -5.119 |
| ZINC000005733652 | -5.119 |
| ZINC000003812862 | -5.113 |
| ZINC000001035331 | -5.108 |
| ZINC000000002101 | -5.107 |
| ZINC000000643046 | -5.092 |
| ZINC000008015016 | -5.069 |
| ZINC000004228258 | -5.069 |
| ZINC000004228258 | -5.069 |
| ZINC000000518554 | -5.064 |
| ZINC000000002028 | -5.052 |
| ZINC000003831475 | -5.05 |
| ZINC000004340269 | -5.037 |
| ZINC000001482184 | -5.023 |
| ZINC000000002273 | -5.015 |
| ZINC000000001899 | -5.001 |
| ZINC000008220909 | -4.997 |
| ZINC000043100953 | -4.993 |
| ZINC000043100953 | -4.99 |
| ZINC000043100953 | -4.99 |
| ZINC000000057624 | -4.987 |
| ZINC000016052277 | -4.985 |
| ZINC000003813042 | -4.981 |
| ZINC000004228258 | -4.978 |
| ZINC000000000469 | -4.976 |
| ZINC000002539702 | -4.966 |
| ZINC000085537014 | -4.963 |
| ZINC000085537014 | -4.963 |
| ZINC000004340269 | -4.955 |
| ZINC000003775644 | -4.951 |
| ZINC000169621220 | -4.943 |
| ZINC000001530555 | -4.931 |
| ZINC000005133378 | -4.931 |
| ZINC000006382803 | -4.923 |
| ZINC000001995484 | -4.921 |
| ZINC000003989268 | -4.921 |
| ZINC000096006023 | -4.921 |
| ZINC000003806262 | -4.918 |
| ZINC000003806262 | -4.918 |
| ZINC000021982937 | -4.912 |
| ZINC000021982937 | -4.912 |
| ZINC000014210876 | -4.911 |
| ZINC000001482049 | -4.897 |
| ZINC000001482049 | -4.897 |
| ZINC000002539827 | -4.895 |
| ZINC000014210876 | -4.894 |
| ZINC000004216238 | -4.89 |
| ZINC000002539827 | -4.881 |
| ZINC000100070937 | -4.874 |
| ZINC000003860453 | -4.871 |
| ZINC000009212428 | -4.87 |
| ZINC000009212428 | -4.87 |
| ZINC000009212428 | -4.87 |
| ZINC000003830215 | -4.86 |
| ZINC000003830959 | -4.853 |
| ZINC000001995484 | -4.837 |
| ZINC000100006770 | -4.822 |
| ZINC000000000746 | -4.819 |
| ZINC000003801919 | -4.818 |
| ZINC000003801919 | -4.818 |
| ZINC000022116608 | -4.816 |
| ZINC000001533877 | -4.813 |
| ZINC000001533877 | -4.813 |
| ZINC000001547851 | -4.813 |
| ZINC000003801919 | -4.806 |
| ZINC000084441937 | -4.801 |
| ZINC000084441937 | -4.801 |
| ZINC000013298313 | -4.79 |
| ZINC000008855117 | -4.784 |
| ZINC000008855117 | -4.783 |
| ZINC000018043251 | -4.782 |
| ZINC000006467621 | -4.776 |
| ZINC000004658552 | -4.772 |
| ZINC000001533877 | -4.76 |
| ZINC000001533877 | -4.76 |
| ZINC000001533877 | -4.76 |
| ZINC000001533877 | -4.76 |
| ZINC000009212427 | -4.743 |
| ZINC000001530283 | -4.74 |
| ZINC000000039089 | -4.736 |
| ZINC000003803652 | -4.696 |
| ZINC000016929327 | -4.692 |
| ZINC000000000416 | -4.691 |
| ZINC000018279854 | -4.691 |
| ZINC000003872277 | -4.681 |
| ZINC000003795098 | -4.671 |
| ZINC000006661227 | -4.665 |
| ZINC000006661227 | -4.665 |
| ZINC000006661227 | -4.665 |
| ZINC000002015035 | -4.651 |
| ZINC000002015035 | -4.651 |
| ZINC000009212428 | -4.64 |
| ZINC000009212428 | -4.639 |
| ZINC000003842753 | -4.631 |
| ZINC000004097426 | -4.625 |
| ZINC000004097426 | -4.625 |
| ZINC000004097426 | -4.625 |
| ZINC000000002281 | -4.62 |
| ZINC000000000416 | -4.615 |
| ZINC000001543916 | -4.613 |
| ZINC000001543916 | -4.608 |
| ZINC000003830391 | -4.601 |
| ZINC000022116612 | -4.601 |
| ZINC000000013156 | -4.601 |
| ZINC000021982937 | -4.6 |
| ZINC000021982937 | -4.6 |
| ZINC000021982937 | -4.6 |
| ZINC000013298313 | -4.597 |
| ZINC000003830391 | -4.594 |
| ZINC000004658553 | -4.594 |
| ZINC000003918453 | -4.588 |
| ZINC000003830990 | -4.586 |
| ZINC000008101109 | -4.568 |
| ZINC000008101109 | -4.568 |
| ZINC000008101109 | -4.568 |
| ZINC000006661227 | -4.56 |
| ZINC000006661227 | -4.56 |
| ZINC000006661227 | -4.56 |
| ZINC000028957444 | -4.557 |
| ZINC000022116608 | -4.554 |
| ZINC000003982483 | -4.554 |
| ZINC000003982483 | -4.554 |
| ZINC000003982483 | -4.554 |
| ZINC000003813010 | -4.551 |
| ZINC000084441937 | -4.542 |
| ZINC000084668739 | -4.541 |
| ZINC000003842753 | -4.539 |
| ZINC000000000922 | -4.53 |
| ZINC000006827693 | -4.527 |
| ZINC000000049154 | -4.516 |
| ZINC000000018635 | -4.515 |
| ZINC000003629271 | -4.515 |
| ZINC000003830215 | -4.513 |
| ZINC000000895360 | -4.507 |
| ZINC000000007601 | -4.498 |
| ZINC000004658290 | -4.493 |
| ZINC000000002055 | -4.493 |
| ZINC000000002055 | -4.493 |
| ZINC000242437514 | -4.491 |
| ZINC000019419017 | -4.49 |
| ZINC000019419017 | -4.49 |
| ZINC000000388081 | -4.485 |
| ZINC000000388081 | -4.485 |
| ZINC000006627681 | -4.484 |
| ZINC000003831477 | -4.483 |
| ZINC000000896546 | -4.482 |
| ZINC000000896546 | -4.48 |
| ZINC000008214514 | -4.478 |
| ZINC000003776970 | -4.475 |
| ZINC000000388081 | -4.474 |
| ZINC000003830957 | -4.473 |
| ZINC000003872520 | -4.47 |
| ZINC000001532517 | -4.466 |
| ZINC000000001341 | -4.462 |
| ZINC000000001341 | -4.462 |
| ZINC000000156395 | -4.46 |
| ZINC000003775644 | -4.453 |
| ZINC000022059268 | -4.45 |
| ZINC000000083315 | -4.448 |
| ZINC000000020253 | -4.447 |
| ZINC000028467879 | -4.446 |
| ZINC000028467879 | -4.446 |
| ZINC000001535101 | -4.429 |
| ZINC000003776970 | -4.429 |
| ZINC000003813010 | -4.427 |
| ZINC000003808779 | -4.425 |
| ZINC000003830891 | -4.424 |
| ZINC000006827693 | -4.422 |
| ZINC000000034157 | -4.418 |
| ZINC000014879972 | -4.418 |
| ZINC000001530621 | -4.417 |
| ZINC000100071256 | -4.416 |
| ZINC000000057512 | -4.416 |
| ZINC000000001795 | -4.414 |
| ZINC000003779042 | -4.413 |
| ZINC000012360535 | -4.412 |
| ZINC000001530621 | -4.408 |
| ZINC000000057512 | -4.408 |
| ZINC000000388081 | -4.407 |
| ZINC000000000882 | -4.407 |
| ZINC000000000882 | -4.407 |
| ZINC000022116612 | -4.406 |
| ZINC000001530713 | -4.406 |
| ZINC000000388081 | -4.405 |
| ZINC000000388081 | -4.405 |
| ZINC000000896569 | -4.401 |
| ZINC000000014360 | -4.398 |
| ZINC000002005305 | -4.397 |
| ZINC000003918138 | -4.395 |
| ZINC000000020231 | -4.387 |
| ZINC000000403011 | -4.386 |
| ZINC000029319828 | -4.386 |
| ZINC000008034120 | -4.385 |
| ZINC000000403011 | -4.385 |
| ZINC000000020230 | -4.384 |
| ZINC000003872055 | -4.371 |
| ZINC000000002159 | -4.369 |
| ZINC000001530713 | -4.364 |
| ZINC000006627681 | -4.364 |
| ZINC000009212427 | -4.361 |
| ZINC000028108825 | -4.358 |
| ZINC000028108825 | -4.358 |
| ZINC000028108825 | -4.358 |
| ZINC000000001735 | -4.358 |
| ZINC000004468778 | -4.355 |
| ZINC000001530599 | -4.354 |
| ZINC000003831474 | -4.352 |
| ZINC000012360535 | -4.35 |
| ZINC000001895505 | -4.345 |
| ZINC000001690324 | -4.344 |
| ZINC000001690324 | -4.344 |
| ZINC000013818943 | -4.344 |
| ZINC000001530599 | -4.342 |
| ZINC000009212427 | -4.341 |
| ZINC000009212428 | -4.335 |
| ZINC000000000882 | -4.326 |
| ZINC000013648755 | -4.325 |
| ZINC000013648755 | -4.325 |
| ZINC000000000882 | -4.324 |
| ZINC000013520815 | -4.318 |
| ZINC000000137884 | -4.316 |
| ZINC000003798247 | -4.315 |
| ZINC000034220093 | -4.313 |
| ZINC000019168887 | -4.307 |
| ZINC000001530427 | -4.306 |
| ZINC000001530427 | -4.306 |
| ZINC000001530427 | -4.306 |
| ZINC000003812869 | -4.305 |
| ZINC000009212427 | -4.304 |
| ZINC000009302239 | -4.303 |
| ZINC000004258316 | -4.298 |
| ZINC000003827556 | -4.295 |
| ZINC000033965961 | -4.289 |
| ZINC000033965961 | -4.289 |
| ZINC000000085733 | -4.289 |
| ZINC000000538621 | -4.288 |
| ZINC000000004319 | -4.286 |
| ZINC000033965961 | -4.286 |
| ZINC000033965961 | -4.286 |
| ZINC000033965961 | -4.286 |
| ZINC000008577218 | -4.283 |
| ZINC000000538621 | -4.282 |
| ZINC000003874498 | -4.279 |
| ZINC000000004319 | -4.274 |
| ZINC000002005305 | -4.273 |
| ZINC000000001688 | -4.27 |
| ZINC000000001688 | -4.267 |
| ZINC000003830264 | -4.262 |
| ZINC000029416466 | -4.254 |
| ZINC000029416466 | -4.254 |
| ZINC000029416466 | -4.254 |
| ZINC000000000693 | -4.254 |
| ZINC000000075126 | -4.247 |
| ZINC000000895199 | -4.245 |
| ZINC000000004448 | -4.245 |
| ZINC000003914809 | -4.239 |
| ZINC000012859773 | -4.235 |
| ZINC000000537957 | -4.229 |
| ZINC000000033882 | -4.223 |
| ZINC000013597823 | -4.219 |
| ZINC000000005823 | -4.218 |
| ZINC000100009383 | -4.216 |
| ZINC000002005305 | -4.213 |
| ZINC000004095696 | -4.211 |
| ZINC000000020231 | -4.208 |
| ZINC000000000850 | -4.207 |
| ZINC000000000850 | -4.206 |
| ZINC000026985532 | -4.206 |
| ZINC000026985532 | -4.206 |
| ZINC000026985532 | -4.206 |
| ZINC000000001132 | -4.205 |
| ZINC000003871967 | -4.194 |
| ZINC000003927198 | -4.193 |
| ZINC000003823492 | -4.193 |
| ZINC000001530922 | -4.189 |
| ZINC000001530922 | -4.189 |
| ZINC000001530922 | -4.189 |
| ZINC000013550868 | -4.187 |
| ZINC000001529323 | -4.186 |
| ZINC000022448696 | -4.185 |
| ZINC000022448696 | -4.185 |
| ZINC000022448696 | -4.185 |
| ZINC000022448696 | -4.185 |
| ZINC000022448696 | -4.185 |
| ZINC000035801098 | -4.184 |
| ZINC000003830569 | -4.182 |
| ZINC000035801098 | -4.177 |
| ZINC000000266964 | -4.177 |
| ZINC000000897258 | -4.175 |
| ZINC000000403010 | -4.172 |
| ZINC000003781664 | -4.171 |
| ZINC000000005823 | -4.17 |
| ZINC000000403010 | -4.17 |
| ZINC000022447798 | -4.17 |
| ZINC000022447798 | -4.17 |
| ZINC000003812865 | -4.162 |
| ZINC000000012346 | -4.155 |
| ZINC000038212689 | -4.154 |
| ZINC000038212689 | -4.153 |
| ZINC000001530600 | -4.151 |
| ZINC000001530600 | -4.151 |
| ZINC000000057146 | -4.148 |
| ZINC000026664090 | -4.147 |
| ZINC000026664090 | -4.147 |
| ZINC000026664090 | -4.147 |
| ZINC000000020259 | -4.147 |
| ZINC000000003911 | -4.144 |
| ZINC000001530600 | -4.143 |
| ZINC000000001644 | -4.141 |
| ZINC000003782807 | -4.14 |
| ZINC000001997127 | -4.138 |
| ZINC000000056652 | -4.137 |
| ZINC000003831165 | -4.136 |
| ZINC000001997125 | -4.136 |
| ZINC000001540998 | -4.136 |
| ZINC000000006310 | -4.133 |
| ZINC000005133329 | -4.129 |
| ZINC000003798064 | -4.124 |
| ZINC000013597823 | -4.116 |
| ZINC000242437512 | -4.11 |
| ZINC000000035804 | -4.107 |
| ZINC000000120286 | -4.104 |
| ZINC000022059930 | -4.103 |
| ZINC000000000507 | -4.102 |
| ZINC000001531008 | -4.101 |
| ZINC000000007295 | -4.098 |
| ZINC000000000507 | -4.097 |
| ZINC000000538275 | -4.096 |
| ZINC000000538275 | -4.096 |
| ZINC000100036924 | -4.095 |
| ZINC000008101126 | -4.092 |
| ZINC000003914808 | -4.091 |
| ZINC000002005305 | -4.088 |
| ZINC000000901736 | -4.088 |
| ZINC000002036848 | -4.087 |
| ZINC000002036848 | -4.087 |
| ZINC000001530788 | -4.087 |
| ZINC000003913937 | -4.086 |
| ZINC000000020220 | -4.083 |
| ZINC000003813003 | -4.082 |
| ZINC000001690604 | -4.079 |
| ZINC000000004785 | -4.078 |
| ZINC000038197764 | -4.074 |
| ZINC000003830960 | -4.073 |
| ZINC000001883067 | -4.073 |
| ZINC000000001011 | -4.073 |
| ZINC000003940470 | -4.073 |
| ZINC000004658290 | -4.073 |
| ZINC000003873921 | -4.072 |
| ZINC000000057512 | -4.068 |
| ZINC000000599985 | -4.068 |
| ZINC000001530636 | -4.063 |
| ZINC000000057313 | -4.063 |
| ZINC000002000707 | -4.062 |
| ZINC000003812897 | -4.062 |
| ZINC000013597823 | -4.061 |
| ZINC000000000061 | -4.061 |
| ZINC000013597823 | -4.06 |
| ZINC000003830453 | -4.059 |
| ZINC000052971887 | -4.058 |
| ZINC000052971887 | -4.058 |
| ZINC000003786192 | -4.058 |
| ZINC000006733300 | -4.056 |
| ZINC000005162311 | -4.054 |
| ZINC000001849548 | -4.051 |
| ZINC000000538621 | -4.05 |
| ZINC000000538621 | -4.05 |
| ZINC000000895081 | -4.05 |
| ZINC000000057464 | -4.048 |
| ZINC000028232750 | -4.046 |
| ZINC000028232750 | -4.045 |
| ZINC000000008492 | -4.045 |
| ZINC000000000905 | -4.042 |
| ZINC000002005305 | -4.041 |
| ZINC000029571072 | -4.041 |
| ZINC000051133897 | -4.039 |
| ZINC000004474443 | -4.035 |
| ZINC000000901791 | -4.035 |
| ZINC000028232750 | -4.034 |
| ZINC000038197764 | -4.03 |
| ZINC000001886617 | -4.024 |
| ZINC000013129998 | -4.018 |
| ZINC000004474443 | -4.017 |
| ZINC000018516586 | -4.017 |
| ZINC000014164617 | -4.016 |
| ZINC000003813088 | -4.01 |
| ZINC000000113355 | -4.007 |
| ZINC000002599970 | -4.007 |
| ZINC000026011099 | -4.002 |
| ZINC000026011099 | -4.002 |
| ZINC000000004724 | -4.002 |
| ZINC000000967597 | -4.002 |
| ZINC000009224016 | -3.995 |
| ZINC000009224016 | -3.995 |
| ZINC000002599970 | -3.989 |
| ZINC000000001317 | -3.984 |
| ZINC000002548959 | -3.983 |
| ZINC000000009073 | -3.98 |
| ZINC000000009073 | -3.98 |
| ZINC000000009073 | -3.98 |
| ZINC000003812863 | -3.972 |
| ZINC000003812863 | -3.968 |
| ZINC000004215234 | -3.963 |
| ZINC000034676245 | -3.963 |
| ZINC000018099446 | -3.958 |
| ZINC000100019007 | -3.958 |
| ZINC000003830500 | -3.956 |
| ZINC000000002279 | -3.955 |
| ZINC000003830500 | -3.954 |
| ZINC000100007011 | -3.952 |
| ZINC000018203737 | -3.939 |
| ZINC000018203737 | -3.939 |
| ZINC000004658603 | -3.939 |
| ZINC000004658603 | -3.939 |
| ZINC000000000373 | -3.93 |
| ZINC000003861806 | -3.923 |
| ZINC000000895457 | -3.923 |
| ZINC000051951647 | -3.922 |
| ZINC000003812851 | -3.921 |
| ZINC000003914810 | -3.92 |
| ZINC000000119344 | -3.919 |
| ZINC000004213946 | -3.916 |
| ZINC000001530599 | -3.912 |
| ZINC000011679756 | -3.911 |
| ZINC000000000740 | -3.91 |
| ZINC000011679756 | -3.91 |
| ZINC000000897256 | -3.909 |
| ZINC000000895302 | -3.908 |
| ZINC000014210457 | -3.907 |
| ZINC000004213946 | -3.905 |
| ZINC000053022902 | -3.903 |
| ZINC000053022902 | -3.903 |
| ZINC000001530948 | -3.901 |
| ZINC000001493878 | -3.9 |
| ZINC000034636383 | -3.898 |
| ZINC000012360535 | -3.896 |
| ZINC000001532728 | -3.894 |
| ZINC000029571072 | -3.893 |
| ZINC000000897256 | -3.892 |
| ZINC000000016154 | -3.891 |
| ZINC000001843047 | -3.887 |
| ZINC000000002005 | -3.884 |
| ZINC000001530974 | -3.883 |
| ZINC000001187543 | -3.883 |
| ZINC000003809490 | -3.883 |
| ZINC000043450324 | -3.878 |
| ZINC000043450324 | -3.878 |
| ZINC000001530803 | -3.873 |
| ZINC000084843283 | -3.872 |
| ZINC000003927198 | -3.871 |
| ZINC000013540519 | -3.87 |
| ZINC000004258316 | -3.869 |
| ZINC000000491073 | -3.863 |
| ZINC000000491073 | -3.863 |
| ZINC000000491073 | -3.863 |
| ZINC000003604264 | -3.861 |
| ZINC000012503151 | -3.86 |
| ZINC000253917094 | -3.86 |
| ZINC000000113442 | -3.857 |
| ZINC000001481815 | -3.856 |
| ZINC000001481815 | -3.855 |
| ZINC000006036847 | -3.853 |
| ZINC000000004321 | -3.853 |
| ZINC000003798763 | -3.852 |
| ZINC000003791297 | -3.847 |
| ZINC000003812851 | -3.846 |
| ZINC000003833846 | -3.843 |
| ZINC000003833846 | -3.843 |
| ZINC000003833846 | -3.843 |
| ZINC000000000431 | -3.842 |
| ZINC000008034121 | -3.841 |
| ZINC000000011012 | -3.839 |
| ZINC000003830405 | -3.839 |
| ZINC000000113382 | -3.839 |
| ZINC000000057255 | -3.837 |
| ZINC000000113382 | -3.834 |
| ZINC000095564694 | -3.83 |
| ZINC000095564694 | -3.83 |
| ZINC000095564694 | -3.83 |
| ZINC000003785268 | -3.829 |
| ZINC000001530973 | -3.829 |
| ZINC000000968303 | -3.825 |
| ZINC000000002191 | -3.822 |
| ZINC000003814422 | -3.822 |
| ZINC000043206370 | -3.82 |
| ZINC000003819138 | -3.814 |
| ZINC000003787060 | -3.813 |
| ZINC000019419017 | -3.812 |
| ZINC000000897258 | -3.809 |
| ZINC000019632917 | -3.806 |
| ZINC000000004166 | -3.806 |
| ZINC000001554588 | -3.803 |
| ZINC000003831040 | -3.799 |
| ZINC000000020255 | -3.796 |
| ZINC000014961096 | -3.794 |
| ZINC000011615926 | -3.792 |
| ZINC000004097309 | -3.792 |
| ZINC000253476025 | -3.79 |
| ZINC000100015780 | -3.79 |
| ZINC000004212854 | -3.789 |
| ZINC000003830449 | -3.788 |
| ZINC000001489478 | -3.787 |
| ZINC000001489478 | -3.787 |
| ZINC000011681534 | -3.786 |
| ZINC000011681534 | -3.785 |
| ZINC000003802417 | -3.784 |
| ZINC000003802417 | -3.784 |
| ZINC000003914596 | -3.783 |
| ZINC000003914596 | -3.783 |
| ZINC000003914596 | -3.783 |
| ZINC000000020220 | -3.782 |
| ZINC000001481833 | -3.782 |
| ZINC000000003642 | -3.781 |
| ZINC000003876186 | -3.776 |
| ZINC000003831139 | -3.776 |
| ZINC000002015928 | -3.773 |
| ZINC000001536109 | -3.772 |
| ZINC000003806413 | -3.772 |
| ZINC000000403609 | -3.771 |
| ZINC000004474405 | -3.769 |
| ZINC000036294079 | -3.769 |
| ZINC000003806413 | -3.768 |
| ZINC000036294079 | -3.768 |
| ZINC000000537795 | -3.765 |
| ZINC000003794711 | -3.759 |
| ZINC000003794711 | -3.759 |
| ZINC000003794711 | -3.759 |
| ZINC000004640636 | -3.757 |
| ZINC000004640636 | -3.757 |
| ZINC000006858022 | -3.756 |
| ZINC000003872994 | -3.755 |
| ZINC000013537284 | -3.752 |
| ZINC000095616600 | -3.752 |
| ZINC000022448097 | -3.751 |
| ZINC000022448097 | -3.751 |
| ZINC000022448097 | -3.751 |
| ZINC000043207238 | -3.751 |
| ZINC000100005670 | -3.75 |
| ZINC000013537284 | -3.75 |
| ZINC000242437513 | -3.749 |
| ZINC000000057341 | -3.745 |
| ZINC000018203737 | -3.745 |
| ZINC000018203737 | -3.745 |
| ZINC000001530806 | -3.744 |
| ZINC000000896731 | -3.743 |
| ZINC000000896731 | -3.743 |
| ZINC000003938704 | -3.742 |
| ZINC000000001773 | -3.741 |
| ZINC000000001773 | -3.741 |
| ZINC000000001773 | -3.741 |
| ZINC000003812869 | -3.739 |
| ZINC000001482164 | -3.738 |
| ZINC000003981610 | -3.736 |
| ZINC000000001084 | -3.736 |
| ZINC000002519740 | -3.735 |
| ZINC000002519740 | -3.735 |
| ZINC000002519740 | -3.735 |
| ZINC000001530947 | -3.734 |
| ZINC000001530639 | -3.732 |
| ZINC000253476027 | -3.731 |
| ZINC000100015775 | -3.731 |
| ZINC000000057340 | -3.727 |
| ZINC000000599985 | -3.726 |
| ZINC000003875484 | -3.725 |
| ZINC000003810860 | -3.725 |
| ZINC000000389747 | -3.72 |
| ZINC000000389747 | -3.72 |
| ZINC000000389747 | -3.72 |
| ZINC000008403947 | -3.718 |
| ZINC000000001145 | -3.713 |
| ZINC000003871978 | -3.711 |
| ZINC000000001958 | -3.711 |
| ZINC000003818808 | -3.71 |
| ZINC000000005878 | -3.708 |
| ZINC000000897288 | -3.708 |
| ZINC000022016981 | -3.708 |
| ZINC000022016981 | -3.708 |
| ZINC000022016981 | -3.708 |
| ZINC000000001979 | -3.707 |
| ZINC000003830276 | -3.706 |
| ZINC000003952881 | -3.705 |
| ZINC000100296832 | -3.703 |
| ZINC000100296832 | -3.703 |
| ZINC000006021033 | -3.702 |
| ZINC000003929508 | -3.701 |
| ZINC000000001145 | -3.698 |
| ZINC000000537805 | -3.694 |
| ZINC000002005305 | -3.691 |
| ZINC000013831130 | -3.69 |
| ZINC000000007782 | -3.686 |
| ZINC000021303210 | -3.686 |
| ZINC000000004076 | -3.686 |
| ZINC000003874715 | -3.686 |
| ZINC000000000711 | -3.683 |
| ZINC000003875368 | -3.681 |
| ZINC000003875368 | -3.681 |
| ZINC000000897222 | -3.681 |
| ZINC000013682481 | -3.677 |
| ZINC000003938704 | -3.677 |
| ZINC000000074836 | -3.677 |
| ZINC000000013156 | -3.677 |
| ZINC000013831130 | -3.676 |
| ZINC000004097476 | -3.676 |
| ZINC000013682481 | -3.675 |
| ZINC000028973441 | -3.672 |
| ZINC000003800475 | -3.671 |
| ZINC000116473771 | -3.671 |
| ZINC000116473771 | -3.669 |
| ZINC000000006481 | -3.669 |
| ZINC000001996784 | -3.669 |
| ZINC000000001758 | -3.668 |
| ZINC000035342789 | -3.667 |
| ZINC000000056653 | -3.666 |
| ZINC000001846431 | -3.666 |
| ZINC000000968255 | -3.661 |
| ZINC000000020243 | -3.66 |
| ZINC000003872738 | -3.657 |
| ZINC000008214692 | -3.655 |
| ZINC000000049153 | -3.653 |
| ZINC000035024346 | -3.653 |
| ZINC000022059926 | -3.652 |
| ZINC000085205451 | -3.651 |
| ZINC000019702309 | -3.649 |
| ZINC000000403079 | -3.649 |
| ZINC000000403079 | -3.643 |
| ZINC000000509440 | -3.64 |
| ZINC000003873160 | -3.637 |
| ZINC000003873160 | -3.636 |
| ZINC000000006157 | -3.634 |
| ZINC000095626706 | -3.631 |
| ZINC000004099200 | -3.63 |
| ZINC000001542113 | -3.625 |
| ZINC000001542113 | -3.625 |
| ZINC000016052277 | -3.624 |
| ZINC000016052277 | -3.624 |
| ZINC000003875332 | -3.623 |
| ZINC000000105216 | -3.619 |
| ZINC000014879992 | -3.619 |
| ZINC000014879992 | -3.619 |
| ZINC000018203737 | -3.618 |
| ZINC000018203737 | -3.618 |
| ZINC000000896819 | -3.617 |
| ZINC000003943279 | -3.615 |
| ZINC000019632912 | -3.615 |
| ZINC000005764759 | -3.613 |
| ZINC000004097304 | -3.607 |
| ZINC000002570817 | -3.604 |
| ZINC000003781943 | -3.604 |
| ZINC000003607120 | -3.603 |
| ZINC000410428674 | -3.603 |
| ZINC000003955219 | -3.602 |
| ZINC000000113446 | -3.598 |
| ZINC000087515509 | -3.597 |
| ZINC000000000471 | -3.597 |
| ZINC000000000856 | -3.595 |
| ZINC000012358719 | -3.592 |
| ZINC000000002272 | -3.591 |
| ZINC000001530710 | -3.589 |
| ZINC000000537891 | -3.587 |
| ZINC000000537891 | -3.587 |
| ZINC000003812944 | -3.587 |
| ZINC000003812988 | -3.585 |
| ZINC000003812988 | -3.585 |
| ZINC000004468780 | -3.585 |
| ZINC000000089763 | -3.584 |
| ZINC000000105196 | -3.583 |
| ZINC000000537931 | -3.581 |
| ZINC000019166988 | -3.578 |
| ZINC000000596881 | -3.576 |
| ZINC000003607120 | -3.576 |
| ZINC000000154964 | -3.575 |
| ZINC000000154964 | -3.575 |
| ZINC000000154964 | -3.575 |
| ZINC000001530694 | -3.572 |
| ZINC000012468792 | -3.571 |
| ZINC000000000856 | -3.567 |
| ZINC000000538564 | -3.566 |
| ZINC000000591993 | -3.566 |
| ZINC000000089763 | -3.564 |
| ZINC000003953037 | -3.559 |
| ZINC000150338699 | -3.557 |
| ZINC000000004351 | -3.553 |
| ZINC000000004351 | -3.553 |
| ZINC000001530863 | -3.553 |
| ZINC000003830993 | -3.55 |
| ZINC000003830993 | -3.55 |
| ZINC000043100709 | -3.549 |
| ZINC000019702309 | -3.547 |
| ZINC000001530611 | -3.546 |
| ZINC000100378061 | -3.545 |
| ZINC000100378061 | -3.545 |
| ZINC000100378061 | -3.545 |
| ZINC000000607971 | -3.544 |
| ZINC000004099200 | -3.544 |
| ZINC000072267023 | -3.544 |
| ZINC000072267023 | -3.544 |
| ZINC000003953037 | -3.543 |
| ZINC000000000905 | -3.543 |
| ZINC000100017856 | -3.539 |
| ZINC000100017856 | -3.539 |
| ZINC000030691736 | -3.536 |
| ZINC000011726211 | -3.53 |
| ZINC000011726211 | -3.53 |
| ZINC000003871701 | -3.529 |
| ZINC000021981454 | -3.527 |
| ZINC000013537284 | -3.525 |
| ZINC000000968264 | -3.524 |
| ZINC000000968264 | -3.524 |
| ZINC000017146904 | -3.524 |
| ZINC000003875357 | -3.522 |
| ZINC000014210642 | -3.521 |
| ZINC000100032379 | -3.52 |
| ZINC000000057464 | -3.52 |
| ZINC000003830713 | -3.519 |
| ZINC000000000456 | -3.518 |
| ZINC000000000456 | -3.518 |
| ZINC000000000456 | -3.518 |
| ZINC000012503177 | -3.518 |
| ZINC000003795819 | -3.517 |
| ZINC000003795819 | -3.517 |
| ZINC000003806063 | -3.517 |
| ZINC000002510358 | -3.516 |
| ZINC000100032379 | -3.515 |
| ZINC000005179119 | -3.515 |
| ZINC000002510358 | -3.515 |
| ZINC000003079340 | -3.513 |
| ZINC000100074252 | -3.513 |
| ZINC000003814395 | -3.513 |
| ZINC000164760756 | -3.513 |
| ZINC000014879992 | -3.511 |
| ZINC000000044027 | -3.508 |
| ZINC000003818726 | -3.507 |
| ZINC000003818726 | -3.507 |
| ZINC000003875560 | -3.506 |
| ZINC000000968375 | -3.505 |
| ZINC000013512456 | -3.501 |
| ZINC000084589076 | -3.5 |
| ZINC000000000053 | -3.5 |
| ZINC000000896918 | -3.498 |
| ZINC000003938751 | -3.494 |
| ZINC000060325170 | -3.493 |
| ZINC000003872931 | -3.492 |
| ZINC000003872931 | -3.492 |
| ZINC000000000083 | -3.489 |
| ZINC000013986658 | -3.489 |
| ZINC000000000271 | -3.487 |
| ZINC000000005895 | -3.486 |
| ZINC000052716421 | -3.486 |
| ZINC000052716421 | -3.486 |
| ZINC000000001554 | -3.485 |
| ZINC000000057435 | -3.483 |
| ZINC000013986658 | -3.483 |
| ZINC000000601283 | -3.481 |
| ZINC000014210642 | -3.478 |
| ZINC000011615927 | -3.477 |
| ZINC000000121541 | -3.477 |
| ZINC000000049154 | -3.476 |
| ZINC000000005560 | -3.475 |
| ZINC000000020245 | -3.472 |
| ZINC000000009342 | -3.47 |
| ZINC000000009342 | -3.47 |
| ZINC000101489663 | -3.469 |
| ZINC000000009689 | -3.468 |
| ZINC000095619105 | -3.466 |
| ZINC000095619105 | -3.466 |
| ZINC000095619105 | -3.466 |
| ZINC000019364230 | -3.466 |
| ZINC000019364230 | -3.466 |
| ZINC000019364230 | -3.466 |
| ZINC000001530805 | -3.465 |
| ZINC000000025958 | -3.464 |
| ZINC000000113426 | -3.464 |
| ZINC000003815424 | -3.464 |
| ZINC000003807172 | -3.463 |
| ZINC000003813078 | -3.461 |
| ZINC000095616599 | -3.459 |
| ZINC000003871923 | -3.458 |
| ZINC000000968310 | -3.458 |
| ZINC000000968310 | -3.458 |
| ZINC000000968310 | -3.458 |
| ZINC000000897408 | -3.458 |
| ZINC000014768621 | -3.457 |
| ZINC000013537284 | -3.455 |
| ZINC000012495062 | -3.455 |
| ZINC000003873295 | -3.453 |
| ZINC000001530776 | -3.453 |
| ZINC000001576892 | -3.451 |
| ZINC000003986735 | -3.449 |
| ZINC000003986735 | -3.449 |
| ZINC000000018087 | -3.448 |
| ZINC000019166991 | -3.446 |
| ZINC000002525885 | -3.445 |
| ZINC000002525885 | -3.445 |
| ZINC000118912393 | -3.442 |
| ZINC000029319828 | -3.442 |
| ZINC000003813061 | -3.439 |
| ZINC000001530756 | -3.438 |
| ZINC000000001145 | -3.436 |
| ZINC000118912450 | -3.435 |
| ZINC000000601316 | -3.435 |
| ZINC000004693574 | -3.435 |
| ZINC000100006264 | -3.433 |
| ZINC000043450326 | -3.431 |
| ZINC000043450326 | -3.431 |
| ZINC000004097225 | -3.43 |
| ZINC000000001145 | -3.429 |
| ZINC000000097996 | -3.426 |
| ZINC000003807804 | -3.424 |
| ZINC000000000490 | -3.424 |
| ZINC000000000575 | -3.418 |
| ZINC000000895099 | -3.417 |
| ZINC000000896595 | -3.415 |
| ZINC000000020221 | -3.413 |
| ZINC000006409735 | -3.412 |
| ZINC000003830999 | -3.411 |
| ZINC000000057147 | -3.411 |
| ZINC000000001706 | -3.411 |
| ZINC000003830999 | -3.41 |
| ZINC000006409735 | -3.41 |
| ZINC000003807917 | -3.409 |
| ZINC000000537791 | -3.408 |
| ZINC000095452610 | -3.406 |
| ZINC000003812974 | -3.398 |
| ZINC000000395010 | -3.396 |
| ZINC000003794794 | -3.396 |
| ZINC000000001850 | -3.394 |
| ZINC000245204949 | -3.394 |
| ZINC000003798757 | -3.392 |
| ZINC000000020250 | -3.39 |
| ZINC000003823475 | -3.389 |
| ZINC000003823475 | -3.389 |
| ZINC000003823475 | -3.389 |
| ZINC000000601250 | -3.387 |
| ZINC000000537791 | -3.385 |
| ZINC000002008866 | -3.383 |
| ZINC000000006226 | -3.379 |
| ZINC000003986735 | -3.377 |
| ZINC000000896755 | -3.377 |
| ZINC000019364229 | -3.375 |
| ZINC000019364229 | -3.375 |
| ZINC000019364229 | -3.375 |
| ZINC000000121480 | -3.372 |
| ZINC000000020248 | -3.366 |
| ZINC000005224188 | -3.365 |
| ZINC000003944422 | -3.365 |
| ZINC000000601305 | -3.364 |
| ZINC000003818808 | -3.362 |
| ZINC000021303210 | -3.36 |
| ZINC000001853550 | -3.358 |
| ZINC000019796155 | -3.357 |
| ZINC000019796155 | -3.357 |
| ZINC000100037020 | -3.357 |
| ZINC000100037020 | -3.357 |
| ZINC000100037020 | -3.357 |
| ZINC000004475353 | -3.357 |
| ZINC000001843099 | -3.356 |
| ZINC000003799072 | -3.356 |
| ZINC000000004778 | -3.354 |
| ZINC000013512456 | -3.352 |
| ZINC000000000323 | -3.35 |
| ZINC000000020783 | -3.349 |
| ZINC000000643114 | -3.348 |
| ZINC000000057532 | -3.343 |
| ZINC000014210455 | -3.341 |
| ZINC000005844792 | -3.34 |
| ZINC000003941829 | -3.34 |
| ZINC000003941829 | -3.34 |
| ZINC000003871541 | -3.339 |
| ZINC000004393164 | -3.338 |
| ZINC000000056647 | -3.337 |
| ZINC000003875439 | -3.335 |
| ZINC000000897085 | -3.333 |
| ZINC000005844792 | -3.332 |
| ZINC000003941829 | -3.331 |
| ZINC000043207851 | -3.33 |
| ZINC000003831282 | -3.329 |
| ZINC000003882036 | -3.328 |
| ZINC000000000346 | -3.328 |
| ZINC000027428713 | -3.327 |
| ZINC000000897002 | -3.325 |
| ZINC000000897002 | -3.325 |
| ZINC000000897002 | -3.325 |
| ZINC000100061056 | -3.324 |
| ZINC000001481956 | -3.324 |
| ZINC000001481956 | -3.324 |
| ZINC000095616603 | -3.323 |
| ZINC000003920719 | -3.32 |
| ZINC000000968328 | -3.319 |
| ZINC000001530599 | -3.319 |
| ZINC000000008667 | -3.318 |
| ZINC000000896634 | -3.317 |
| ZINC000022010649 | -3.317 |
| ZINC000027428713 | -3.314 |
| ZINC000000602632 | -3.314 |
| ZINC000014210876 | -3.313 |
| ZINC000000896958 | -3.312 |
| ZINC000011677837 | -3.307 |
| ZINC000012503156 | -3.307 |
| ZINC000000897322 | -3.303 |
| ZINC000000004009 | -3.302 |
| ZINC000003812841 | -3.3 |
| ZINC000000897291 | -3.291 |
| ZINC000000000903 | -3.29 |
| ZINC000003917708 | -3.289 |
| ZINC000000057001 | -3.287 |
| ZINC000000001370 | -3.287 |
| ZINC000000000941 | -3.285 |
| ZINC000004632106 | -3.284 |
| ZINC000004632106 | -3.282 |
| ZINC000000006156 | -3.28 |
| ZINC000000020241 | -3.28 |
| ZINC000000001655 | -3.279 |
| ZINC000000000931 | -3.275 |
| ZINC000000000931 | -3.275 |
| ZINC000003812867 | -3.275 |
| ZINC000003782818 | -3.275 |
| ZINC000003782818 | -3.275 |
| ZINC000001530716 | -3.275 |
| ZINC000000896543 | -3.272 |
| ZINC000004474682 | -3.271 |
| ZINC000005844788 | -3.267 |
| ZINC000013986658 | -3.267 |
| ZINC000001530741 | -3.26 |
| ZINC000000388462 | -3.26 |
| ZINC000003589203 | -3.259 |
| ZINC000000014257 | -3.259 |
| ZINC000049637509 | -3.259 |
| ZINC000003995811 | -3.258 |
| ZINC000049637509 | -3.257 |
| ZINC000000002212 | -3.257 |
| ZINC000000537964 | -3.256 |
| ZINC000003831151 | -3.255 |
| ZINC000001542392 | -3.253 |
| ZINC000000538483 | -3.253 |
| ZINC000000538483 | -3.253 |
| ZINC000116473771 | -3.252 |
| ZINC000003978006 | -3.251 |
| ZINC000003995807 | -3.25 |
| ZINC000116473771 | -3.249 |
| ZINC000000002176 | -3.249 |
| ZINC000005844788 | -3.248 |
| ZINC000000002216 | -3.245 |
| ZINC000003876136 | -3.245 |
| ZINC000014210876 | -3.243 |
| ZINC000000002216 | -3.243 |
| ZINC000000901061 | -3.242 |
| ZINC000003651680 | -3.241 |
| ZINC000003873160 | -3.241 |
| ZINC000003873160 | -3.241 |
| ZINC000000003876 | -3.24 |
| ZINC000064033452 | -3.24 |
| ZINC000003651680 | -3.239 |
| ZINC000001530760 | -3.239 |
| ZINC000004658562 | -3.238 |
| ZINC000003812989 | -3.238 |
| ZINC000003812989 | -3.238 |
| ZINC000085537053 | -3.234 |
| ZINC000003920355 | -3.233 |
| ZINC000003921872 | -3.233 |
| ZINC000084757007 | -3.233 |
| ZINC000000000449 | -3.233 |
| ZINC000001530579 | -3.231 |
| ZINC000000020257 | -3.23 |
| ZINC000004212945 | -3.23 |
| ZINC000001530579 | -3.229 |
| ZINC000000897089 | -3.229 |
| ZINC000000114127 | -3.224 |
| ZINC000000005823 | -3.223 |
| ZINC000003995809 | -3.222 |
| ZINC000095616599 | -3.222 |
| ZINC000035328014 | -3.222 |
| ZINC000000601254 | -3.221 |
| ZINC000003812984 | -3.221 |
| ZINC000003812984 | -3.221 |
| ZINC000022851765 | -3.22 |
| ZINC000022851765 | -3.22 |
| ZINC000022851765 | -3.22 |
| ZINC000011616925 | -3.22 |
| ZINC000000002647 | -3.22 |
| ZINC000001530968 | -3.219 |
| ZINC000003979899 | -3.218 |
| ZINC000004693575 | -3.216 |
| ZINC000000537795 | -3.214 |
| ZINC000013545636 | -3.214 |
| ZINC000000896711 | -3.214 |
| ZINC000001530968 | -3.212 |
| ZINC000000057533 | -3.211 |
| ZINC000003871960 | -3.211 |
| ZINC000000001982 | -3.211 |
| ZINC000030691797 | -3.207 |
| ZINC000013545636 | -3.206 |
| ZINC000001999441 | -3.205 |
| ZINC000100008319 | -3.205 |
| ZINC000001999441 | -3.205 |
| ZINC000003830961 | -3.203 |
| ZINC000003806104 | -3.203 |
| ZINC000001530764 | -3.202 |
| ZINC000019875504 | -3.201 |
| ZINC000004217732 | -3.2 |
| ZINC000003873296 | -3.2 |
| ZINC000000136138 | -3.2 |
| ZINC000008552123 | -3.199 |
| ZINC000008552123 | -3.199 |
| ZINC000008552123 | -3.199 |
| ZINC000019875504 | -3.198 |
| ZINC000007997568 | -3.193 |
| ZINC000007997568 | -3.193 |
| ZINC000004097416 | -3.189 |
| ZINC000000000494 | -3.189 |
| ZINC000001530775 | -3.187 |
| ZINC000001534965 | -3.187 |
| ZINC000013831141 | -3.186 |
| ZINC000000136138 | -3.184 |
| ZINC000000968330 | -3.184 |
| ZINC000000896484 | -3.183 |
| ZINC000000896484 | -3.183 |
| ZINC000000004840 | -3.183 |
| ZINC000003797541 | -3.182 |
| ZINC000001532526 | -3.182 |
| ZINC000004676424 | -3.181 |
| ZINC000001530618 | -3.179 |
| ZINC000003875980 | -3.174 |
| ZINC000052957434 | -3.173 |
| ZINC000052957434 | -3.173 |
| ZINC000006094354 | -3.173 |
| ZINC000006094354 | -3.173 |
| ZINC000004097427 | -3.17 |
| ZINC000004097427 | -3.17 |
| ZINC000000001261 | -3.169 |
| ZINC000100014475 | -3.168 |
| ZINC000002568036 | -3.166 |
| ZINC000002568036 | -3.166 |
| ZINC000252679615 | -3.166 |
| ZINC000252679615 | -3.166 |
| ZINC000007997966 | -3.166 |
| ZINC000007997966 | -3.166 |
| ZINC000000002299 | -3.165 |
| ZINC000000002299 | -3.165 |
| ZINC000000002299 | -3.165 |
| ZINC000003787097 | -3.164 |
| ZINC000000896698 | -3.163 |
| ZINC000000896698 | -3.163 |
| ZINC000000897385 | -3.163 |
| ZINC000000120319 | -3.163 |
| ZINC000000120319 | -3.163 |
| ZINC000000113398 | -3.159 |
| ZINC000003798734 | -3.158 |
| ZINC000000537795 | -3.158 |
| ZINC000000000596 | -3.156 |
| ZINC000000000596 | -3.156 |
| ZINC000011677376 | -3.155 |
| ZINC000011677376 | -3.155 |
| ZINC000011677376 | -3.155 |
| ZINC000001843099 | -3.154 |
| ZINC000000056645 | -3.152 |
| ZINC000003816292 | -3.151 |
| ZINC000000006427 | -3.148 |
| ZINC000001530688 | -3.148 |
| ZINC000001530572 | -3.146 |
| ZINC000000607986 | -3.146 |
| ZINC000008214651 | -3.145 |
| ZINC000095551509 | -3.144 |
| ZINC000019796080 | -3.144 |
| ZINC000019796080 | -3.144 |
| ZINC000019796080 | -3.144 |
| ZINC000001530697 | -3.144 |
| ZINC000001530697 | -3.144 |
| ZINC000001530697 | -3.144 |
| ZINC000000607986 | -3.143 |
| ZINC000028973446 | -3.142 |
| ZINC000000402909 | -3.141 |
| ZINC000013587680 | -3.138 |
| ZINC000095616601 | -3.138 |
| ZINC000068247389 | -3.136 |
| ZINC000068247389 | -3.136 |
| ZINC000000895032 | -3.136 |
| ZINC000000968328 | -3.135 |
| ZINC000011615928 | -3.135 |
| ZINC000000001148 | -3.135 |
| ZINC000004214700 | -3.131 |
| ZINC000004214700 | -3.131 |
| ZINC000000968233 | -3.126 |
| ZINC000004074875 | -3.124 |
| ZINC000004074875 | -3.124 |
| ZINC000000114124 | -3.123 |
| ZINC000022010387 | -3.122 |
| ZINC000022010387 | -3.122 |
| ZINC000022010387 | -3.122 |
| ZINC000000403566 | -3.121 |
| ZINC000003830218 | -3.12 |
| ZINC000003830635 | -3.119 |
| ZINC000049841054 | -3.118 |
| ZINC000008214703 | -3.118 |
| ZINC000169621200 | -3.117 |
| ZINC000019632628 | -3.117 |
| ZINC000019632628 | -3.117 |
| ZINC000003830579 | -3.117 |
| ZINC000000896740 | -3.113 |
| ZINC000002008310 | -3.111 |
| ZINC000000897240 | -3.11 |
| ZINC000000897240 | -3.11 |
| ZINC000000897240 | -3.11 |
| ZINC000017146904 | -3.109 |
| ZINC000000156792 | -3.109 |
| ZINC000003645145 | -3.108 |
| ZINC000003833821 | -3.108 |
| ZINC000000000655 | -3.106 |
| ZINC000000608101 | -3.105 |
| ZINC000000538273 | -3.105 |
| ZINC000000538273 | -3.105 |
| ZINC000022002218 | -3.105 |
| ZINC000022002218 | -3.105 |
| ZINC000096006009 | -3.104 |
| ZINC000096006009 | -3.103 |
| ZINC000100055899 | -3.101 |
| ZINC000000015515 | -3.098 |
| ZINC000019228902 | -3.098 |
| ZINC000019228902 | -3.098 |
| ZINC000019228902 | -3.098 |
| ZINC000004632106 | -3.098 |
| ZINC000004632106 | -3.097 |
| ZINC000003875392 | -3.097 |
| ZINC000003875392 | -3.097 |
| ZINC000003875392 | -3.097 |
| ZINC000003830218 | -3.096 |
| ZINC000049643479 | -3.096 |
| ZINC000000002688 | -3.094 |
| ZINC000000007673 | -3.092 |
| ZINC000019364224 | -3.091 |
| ZINC000019364224 | -3.091 |
| ZINC000019364224 | -3.091 |
| ZINC000005752191 | -3.09 |
| ZINC000035902489 | -3.089 |
| ZINC000000968330 | -3.088 |
| ZINC000003784120 | -3.087 |
| ZINC000000075008 | -3.086 |
| ZINC000000075008 | -3.086 |
| ZINC000000075126 | -3.084 |
| ZINC000001530759 | -3.083 |
| ZINC000000621853 | -3.082 |
| ZINC000003831404 | -3.081 |
| ZINC000003831404 | -3.081 |
| ZINC000000968345 | -3.081 |
| ZINC000150338699 | -3.08 |
| ZINC000000968326 | -3.078 |
| ZINC000001482094 | -3.078 |
| ZINC000001482113 | -3.077 |
| ZINC000003831138 | -3.075 |
| ZINC000003873371 | -3.074 |
| ZINC000003873371 | -3.074 |
| ZINC000000001281 | -3.073 |
| ZINC000001530569 | -3.073 |
| ZINC000100001918 | -3.072 |
| ZINC000003816287 | -3.072 |
| ZINC000011616882 | -3.072 |
| ZINC000003830986 | -3.072 |
| ZINC000000968345 | -3.069 |
| ZINC000000000607 | -3.069 |
| ZINC000000020251 | -3.069 |
| ZINC000013986658 | -3.067 |
| ZINC000000002041 | -3.064 |
| ZINC000002016037 | -3.063 |
| ZINC000100001964 | -3.063 |
| ZINC000019632713 | -3.063 |
| ZINC000003780893 | -3.061 |
| ZINC000049783788 | -3.061 |
| ZINC000000896709 | -3.06 |
| ZINC000003813047 | -3.057 |
| ZINC000000020237 | -3.055 |
| ZINC000000020237 | -3.055 |
| ZINC000049783788 | -3.055 |
| ZINC000000901555 | -3.054 |
| ZINC000003776633 | -3.054 |
| ZINC000003776633 | -3.054 |
| ZINC000001542392 | -3.053 |
| ZINC000000002009 | -3.052 |
| ZINC000036520252 | -3.052 |
| ZINC000000113428 | -3.05 |
| ZINC000019796158 | -3.049 |
| ZINC000019796158 | -3.049 |
| ZINC000000056646 | -3.047 |
| ZINC000001530635 | -3.043 |
| ZINC000049783788 | -3.042 |
| ZINC000000056556 | -3.04 |
| ZINC000001530886 | -3.04 |
| ZINC000007997897 | -3.039 |
| ZINC000007997897 | -3.039 |
| ZINC000007997897 | -3.039 |
| ZINC000000968326 | -3.038 |
| ZINC000011677857 | -3.038 |
| ZINC000084758235 | -3.037 |
| ZINC000052509463 | -3.036 |
| ZINC000000119717 | -3.036 |
| ZINC000012503291 | -3.035 |
| ZINC000000895103 | -3.034 |
| ZINC000052509366 | -3.033 |
| ZINC000000057254 | -3.032 |
| ZINC000000057254 | -3.032 |
| ZINC000002570895 | -3.031 |
| ZINC000004658560 | -3.029 |
| ZINC000000643143 | -3.029 |
| ZINC000095452610 | -3.028 |
| ZINC000001530625 | -3.026 |
| ZINC000000896968 | -3.025 |
| ZINC000052509366 | -3.024 |
| ZINC000000000347 | -3.022 |
| ZINC000000000347 | -3.022 |
| ZINC000000000347 | -3.022 |
| ZINC000100017856 | -3.022 |
| ZINC000100017856 | -3.022 |
| ZINC000005029557 | -3.021 |
| ZINC000003831586 | -3.018 |
| ZINC000031274852 | -3.018 |
| ZINC000031274852 | -3.018 |
| ZINC000000537805 | -3.015 |
| ZINC000022443609 | -3.014 |
| ZINC000019364222 | -3.012 |
| ZINC000019364222 | -3.012 |
| ZINC000019364222 | -3.012 |
| ZINC000040899447 | -3.012 |
| ZINC000084758479 | -3.01 |
| ZINC000084758479 | -3.01 |
| ZINC000000606383 | -3.009 |
| ZINC000003977764 | -3.005 |
| ZINC000100018854 | -3.003 |
| ZINC000100018854 | -3.003 |
| ZINC000100018854 | -3.003 |
| ZINC000003843198 | -3.002 |
| ZINC000001481910 | -3.001 |
| ZINC000000537791 | -3.001 |
| ZINC000035999642 | -3 |
| ZINC000000001464 | -2.999 |
| ZINC000000004893 | -2.997 |
| ZINC000000004893 | -2.997 |
| ZINC000000004893 | -2.997 |
| ZINC000012503099 | -2.997 |
| ZINC000012503099 | -2.997 |
| ZINC000000643055 | -2.996 |
| ZINC000035999642 | -2.995 |
| ZINC000000010163 | -2.994 |
| ZINC000000010163 | -2.994 |
| ZINC000000010164 | -2.994 |
| ZINC000000010164 | -2.994 |
| ZINC000001999487 | -2.993 |
| ZINC000027990463 | -2.989 |
| ZINC000018089317 | -2.989 |
| ZINC000001530886 | -2.984 |
| ZINC000000394284 | -2.981 |
| ZINC000035328014 | -2.981 |
| ZINC000004097392 | -2.977 |
| ZINC000003938684 | -2.977 |
| ZINC000000895042 | -2.977 |
| ZINC000003956788 | -2.976 |
| ZINC000019632834 | -2.967 |
| ZINC000019632834 | -2.965 |
| ZINC000019632834 | -2.965 |
| ZINC000222731806 | -2.964 |
| ZINC000001539579 | -2.964 |
| ZINC000001530912 | -2.962 |
| ZINC000001530912 | -2.962 |
| ZINC000001530912 | -2.962 |
| ZINC000004099008 | -2.959 |
| ZINC000000000128 | -2.958 |
| ZINC000001843099 | -2.953 |
| ZINC000003951740 | -2.953 |
| ZINC000000896703 | -2.95 |
| ZINC000000968263 | -2.949 |
| ZINC000003964325 | -2.945 |
| ZINC000095616937 | -2.944 |
| ZINC000003991624 | -2.943 |
| ZINC000000020228 | -2.94 |
| ZINC000003830339 | -2.939 |
| ZINC000000020228 | -2.939 |
| ZINC000003776875 | -2.937 |
| ZINC000000004448 | -2.931 |
| ZINC000014261579 | -2.93 |
| ZINC000000968327 | -2.928 |
| ZINC000000602128 | -2.927 |
| ZINC000100009280 | -2.926 |
| ZINC000100009280 | -2.926 |
| ZINC000012661824 | -2.926 |
| ZINC000095616600 | -2.925 |
| ZINC000001550499 | -2.924 |
| ZINC000000968327 | -2.921 |
| ZINC000001530935 | -2.919 |
| ZINC000000001331 | -2.918 |
| ZINC000000527386 | -2.917 |
| ZINC000014768621 | -2.915 |
| ZINC000000001411 | -2.913 |
| ZINC000003826253 | -2.908 |
| ZINC000022010379 | -2.907 |
| ZINC000022010379 | -2.907 |
| ZINC000022010379 | -2.907 |
| ZINC000000006300 | -2.905 |
| ZINC000006021043 | -2.904 |
| ZINC000000057062 | -2.904 |
| ZINC000009212654 | -2.903 |
| ZINC000000601301 | -2.902 |
| ZINC000000643138 | -2.902 |
| ZINC000253530025 | -2.898 |
| ZINC000018516586 | -2.897 |
| ZINC000000389149 | -2.896 |
| ZINC000004097286 | -2.894 |
| ZINC000000896463 | -2.894 |
| ZINC000100299039 | -2.893 |
| ZINC000003079342 | -2.892 |
| ZINC000068153186 | -2.891 |
| ZINC000022010382 | -2.889 |
| ZINC000022010382 | -2.889 |
| ZINC000022010382 | -2.889 |
| ZINC000000014037 | -2.887 |
| ZINC000011680067 | -2.886 |
| ZINC000004693575 | -2.884 |
| ZINC000000896666 | -2.883 |
| ZINC000000391812 | -2.883 |
| ZINC000000391812 | -2.883 |
| ZINC000000391812 | -2.883 |
| ZINC000003938746 | -2.882 |
| ZINC000003938746 | -2.882 |
| ZINC000003938746 | -2.882 |
| ZINC000000607790 | -2.88 |
| ZINC000021297660 | -2.879 |
| ZINC000021297660 | -2.879 |
| ZINC000000020240 | -2.879 |
| ZINC000022002214 | -2.878 |
| ZINC000022002214 | -2.878 |
| ZINC000242437511 | -2.874 |
| ZINC000001530862 | -2.874 |
| ZINC000001536779 | -2.873 |
| ZINC000013545634 | -2.873 |
| ZINC000003830314 | -2.87 |
| ZINC000000968273 | -2.869 |
| ZINC000005819214 | -2.867 |
| ZINC000003079337 | -2.867 |
| ZINC000013911941 | -2.865 |
| ZINC000052955754 | -2.864 |
| ZINC000052955754 | -2.864 |
| ZINC000052955754 | -2.864 |
| ZINC000000000096 | -2.864 |
| ZINC000000643153 | -2.863 |
| ZINC000002847375 | -2.862 |
| ZINC000002847375 | -2.862 |
| ZINC000013545634 | -2.86 |
| ZINC000006716957 | -2.859 |
| ZINC000003782818 | -2.859 |
| ZINC000003782818 | -2.859 |
| ZINC000036766734 | -2.858 |
| ZINC000000607939 | -2.855 |
| ZINC000085205448 | -2.851 |
| ZINC000003784182 | -2.851 |
| ZINC000000005423 | -2.849 |
| ZINC000000057253 | -2.848 |
| ZINC000000057253 | -2.848 |
| ZINC000000597013 | -2.848 |
| ZINC000000597013 | -2.848 |
| ZINC000000057206 | -2.846 |
| ZINC000000057206 | -2.845 |
| ZINC000148723177 | -2.844 |
| ZINC000148723177 | -2.844 |
| ZINC000000000353 | -2.842 |
| ZINC000016052277 | -2.842 |
| ZINC000003830347 | -2.841 |
| ZINC000003830347 | -2.841 |
| ZINC000000601229 | -2.839 |
| ZINC000000601229 | -2.839 |
| ZINC000000601229 | -2.839 |
| ZINC000000538550 | -2.835 |
| ZINC000000538550 | -2.835 |
| ZINC000000113404 | -2.833 |
| ZINC000095616601 | -2.829 |
| ZINC000000004028 | -2.828 |
| ZINC000000004028 | -2.828 |
| ZINC000116473771 | -2.825 |
| ZINC000003926298 | -2.824 |
| ZINC000001530810 | -2.823 |
| ZINC000000004949 | -2.822 |
| ZINC000003872931 | -2.822 |
| ZINC000003872931 | -2.822 |
| ZINC000095619101 | -2.822 |
| ZINC000095619101 | -2.822 |
| ZINC000003831405 | -2.822 |
| ZINC000003831405 | -2.822 |
| ZINC000003920027 | -2.821 |
| ZINC000035653009 | -2.817 |
| ZINC000001482197 | -2.817 |
| ZINC000003875259 | -2.816 |
| ZINC000003875259 | -2.814 |
| ZINC000053084692 | -2.813 |
| ZINC000000001798 | -2.812 |
| ZINC000000001798 | -2.812 |
| ZINC000000001798 | -2.812 |
| ZINC000000622123 | -2.812 |
| ZINC000053084692 | -2.811 |
| ZINC000000538312 | -2.811 |
| ZINC000000538312 | -2.811 |
| ZINC000001530977 | -2.809 |
| ZINC000001530977 | -2.809 |
| ZINC000001530977 | -2.809 |
| ZINC000000006016 | -2.808 |
| ZINC000000006016 | -2.808 |
| ZINC000003831490 | -2.808 |
| ZINC000116473771 | -2.808 |
| ZINC000094566092 | -2.806 |
| ZINC000034051848 | -2.805 |
| ZINC000034051848 | -2.804 |
| ZINC000000000973 | -2.799 |
| ZINC000068153186 | -2.797 |
| ZINC000003813083 | -2.797 |
| ZINC000004097308 | -2.796 |
| ZINC000003831490 | -2.795 |
| ZINC000019156872 | -2.792 |
| ZINC000019156872 | -2.792 |
| ZINC000019156872 | -2.792 |
| ZINC000004392649 | -2.792 |
| ZINC000095626706 | -2.788 |
| ZINC000000968301 | -2.788 |
| ZINC000022065398 | -2.787 |
| ZINC000004618208 | -2.787 |
| ZINC000100016084 | -2.784 |
| ZINC000043200832 | -2.784 |
| ZINC000043200832 | -2.784 |
| ZINC000043200832 | -2.784 |
| ZINC000003801163 | -2.783 |
| ZINC000002561203 | -2.781 |
| ZINC000003993846 | -2.78 |
| ZINC000003875334 | -2.778 |
| ZINC000003812306 | -2.778 |
| ZINC000012503068 | -2.777 |
| ZINC000001530571 | -2.777 |
| ZINC000001530571 | -2.777 |
| ZINC000034806477 | -2.776 |
| ZINC000000000973 | -2.775 |
| ZINC000019418959 | -2.775 |
| ZINC000019418959 | -2.775 |
| ZINC000019418959 | -2.775 |
| ZINC000001698306 | -2.775 |
| ZINC000001552174 | -2.774 |
| ZINC000022010375 | -2.773 |
| ZINC000022010375 | -2.773 |
| ZINC000022010375 | -2.773 |
| ZINC000004693574 | -2.771 |
| ZINC000012466082 | -2.77 |
| ZINC000252678020 | -2.77 |
| ZINC000012466082 | -2.77 |
| ZINC000252678020 | -2.77 |
| ZINC000001530812 | -2.77 |
| ZINC000001530812 | -2.77 |
| ZINC000001530812 | -2.77 |
| ZINC000000155905 | -2.768 |
| ZINC000001639567 | -2.765 |
| ZINC000000896455 | -2.756 |
| ZINC000000005151 | -2.753 |
| ZINC000000005151 | -2.753 |
| ZINC000000001382 | -2.752 |
| ZINC000019632718 | -2.751 |
| ZINC000001530652 | -2.75 |
| ZINC000001530652 | -2.75 |
| ZINC000001530580 | -2.749 |
| ZINC000001530580 | -2.748 |
| ZINC000003581355 | -2.747 |
| ZINC000000020244 | -2.747 |
| ZINC000000020244 | -2.747 |
| ZINC000004175630 | -2.745 |
| ZINC000004175630 | -2.745 |
| ZINC000001552908 | -2.744 |
| ZINC000003929022 | -2.744 |
| ZINC000000057534 | -2.743 |
| ZINC000001530814 | -2.742 |
| ZINC000001530814 | -2.742 |
| ZINC000001530814 | -2.742 |
| ZINC000000901552 | -2.74 |
| ZINC000019796168 | -2.74 |
| ZINC000019796168 | -2.74 |
| ZINC000019796168 | -2.74 |
| ZINC000003977981 | -2.738 |
| ZINC000000001728 | -2.735 |
| ZINC000003782550 | -2.734 |
| ZINC000003782550 | -2.734 |
| ZINC000003920266 | -2.731 |
| ZINC000000968275 | -2.731 |
| ZINC000002020233 | -2.731 |
| ZINC000000537805 | -2.73 |
| ZINC000100004345 | -2.73 |
| ZINC000003916214 | -2.729 |
| ZINC000003916214 | -2.729 |
| ZINC000003831551 | -2.729 |
| ZINC000003831417 | -2.729 |
| ZINC000003920266 | -2.728 |
| ZINC000000537822 | -2.728 |
| ZINC000000537822 | -2.728 |
| ZINC000004102194 | -2.725 |
| ZINC000000000122 | -2.721 |
| ZINC000000000122 | -2.721 |
| ZINC000008214573 | -2.718 |
| ZINC000003792789 | -2.714 |
| ZINC000000056427 | -2.712 |
| ZINC000012404516 | -2.708 |
| ZINC000012404516 | -2.708 |
| ZINC000000001681 | -2.706 |
| ZINC000000001681 | -2.706 |
| ZINC000003831051 | -2.704 |
| ZINC000000538266 | -2.701 |
| ZINC000003875393 | -2.693 |
| ZINC000003875393 | -2.693 |
| ZINC000003875393 | -2.693 |
| ZINC000000537752 | -2.69 |
| ZINC000000537752 | -2.69 |
| ZINC000001530751 | -2.69 |
| ZINC000001530751 | -2.69 |
| ZINC000000057512 | -2.683 |
| ZINC000003872566 | -2.683 |
| ZINC000000056568 | -2.683 |
| ZINC000003960338 | -2.681 |
| ZINC000001611274 | -2.68 |
| ZINC000013912394 | -2.68 |
| ZINC000001550477 | -2.68 |
| ZINC000053683151 | -2.678 |
| ZINC000053683151 | -2.678 |
| ZINC000053683151 | -2.678 |
| ZINC000001542930 | -2.675 |
| ZINC000000895034 | -2.675 |
| ZINC000000001408 | -2.672 |
| ZINC000058581064 | -2.67 |
| ZINC000000608382 | -2.669 |
| ZINC000000608382 | -2.669 |
| ZINC000000643153 | -2.669 |
| ZINC000000895034 | -2.669 |
| ZINC000008466459 | -2.669 |
| ZINC000100009278 | -2.666 |
| ZINC000100009278 | -2.666 |
| ZINC000001530637 | -2.664 |
| ZINC000000002043 | -2.662 |
| ZINC000000002043 | -2.662 |
| ZINC000096272772 | -2.662 |
| ZINC000003831050 | -2.661 |
| ZINC000001530816 | -2.66 |
| ZINC000001530638 | -2.659 |
| ZINC000100296828 | -2.658 |
| ZINC000100296828 | -2.658 |
| ZINC000001530816 | -2.658 |
| ZINC000019362735 | -2.657 |
| ZINC000019362735 | -2.657 |
| ZINC000019362735 | -2.657 |
| ZINC000019144216 | -2.656 |
| ZINC000019144216 | -2.656 |
| ZINC000000968257 | -2.654 |
| ZINC000100036536 | -2.654 |
| ZINC000100036536 | -2.654 |
| ZINC000019594599 | -2.646 |
| ZINC000019594599 | -2.646 |
| ZINC000019594599 | -2.646 |
| ZINC000000599734 | -2.646 |
| ZINC000000538627 | -2.646 |
| ZINC000003798537 | -2.645 |
| ZINC000000001984 | -2.644 |
| ZINC000000508068 | -2.644 |
| ZINC000100004343 | -2.644 |
| ZINC000001542392 | -2.643 |
| ZINC000001530570 | -2.634 |
| ZINC000001611274 | -2.633 |
| ZINC000003816514 | -2.632 |
| ZINC000000000509 | -2.632 |
| ZINC000000000509 | -2.632 |
| ZINC000000000509 | -2.632 |
| ZINC000003782599 | -2.631 |
| ZINC000004676424 | -2.629 |
| ZINC000003816514 | -2.629 |
| ZINC000003874185 | -2.627 |
| ZINC000001530938 | -2.624 |
| ZINC000001530938 | -2.624 |
| ZINC000004658557 | -2.624 |
| ZINC000019362737 | -2.624 |
| ZINC000019362737 | -2.624 |
| ZINC000019362737 | -2.624 |
| ZINC000001485935 | -2.623 |
| ZINC000012503076 | -2.623 |
| ZINC000003819392 | -2.62 |
| ZINC000030691760 | -2.615 |
| ZINC000000001931 | -2.615 |
| ZINC000000001931 | -2.615 |
| ZINC000030691727 | -2.615 |
| ZINC000003812888 | -2.614 |
| ZINC000003812888 | -2.614 |
| ZINC000000537805 | -2.612 |
| ZINC000004629876 | -2.605 |
| ZINC000094566093 | -2.602 |
| ZINC000003861599 | -2.601 |
| ZINC000000005152 | -2.6 |
| ZINC000004097310 | -2.6 |
| ZINC000003985982 | -2.6 |
| ZINC000000537795 | -2.594 |
| ZINC000003820029 | -2.594 |
| ZINC000000005823 | -2.594 |
| ZINC000011617039 | -2.592 |
| ZINC000003876069 | -2.591 |
| ZINC000000000751 | -2.591 |
| ZINC000000000751 | -2.591 |
| ZINC000000000751 | -2.591 |
| ZINC000003876069 | -2.589 |
| ZINC000019796087 | -2.586 |
| ZINC000019796087 | -2.586 |
| ZINC000019796087 | -2.586 |
| ZINC000040430143 | -2.584 |
| ZINC000001530974 | -2.583 |
| ZINC000100017856 | -2.582 |
| ZINC000100017856 | -2.582 |
| ZINC000003964126 | -2.579 |
| ZINC000003831531 | -2.579 |
| ZINC000003831531 | -2.579 |
| ZINC000000000973 | -2.576 |
| ZINC000000000973 | -2.576 |
| ZINC000000057278 | -2.573 |
| ZINC000000968336 | -2.569 |
| ZINC000094566093 | -2.568 |
| ZINC000000014864 | -2.566 |
| ZINC000003936683 | -2.565 |
| ZINC000003936683 | -2.565 |
| ZINC000100015048 | -2.564 |
| ZINC000000000973 | -2.564 |
| ZINC000013973998 | -2.564 |
| ZINC000000000973 | -2.563 |
| ZINC000012414057 | -2.563 |
| ZINC000001530817 | -2.562 |
| ZINC000019144226 | -2.561 |
| ZINC000001530817 | -2.561 |
| ZINC000003941496 | -2.559 |
| ZINC000000001267 | -2.559 |
| ZINC000001530652 | -2.558 |
| ZINC000001530652 | -2.558 |
| ZINC000001530973 | -2.558 |
| ZINC000001554010 | -2.556 |
| ZINC000003938695 | -2.554 |
| ZINC000003973334 | -2.545 |
| ZINC000000001792 | -2.544 |
| ZINC000003978005 | -2.541 |
| ZINC000003978005 | -2.541 |
| ZINC000003978005 | -2.541 |
| ZINC000001530689 | -2.541 |
| ZINC000001530689 | -2.541 |
| ZINC000001530575 | -2.539 |
| ZINC000003812913 | -2.532 |
| ZINC000001530752 | -2.532 |
| ZINC000001530752 | -2.532 |
| ZINC000001530695 | -2.531 |
| ZINC000001530695 | -2.531 |
| ZINC000001530695 | -2.531 |
| ZINC000085555528 | -2.531 |
| ZINC000085555528 | -2.531 |
| ZINC000085555528 | -2.531 |
| ZINC000085555528 | -2.531 |
| ZINC000007997952 | -2.531 |
| ZINC000007997952 | -2.531 |
| ZINC000012503187 | -2.53 |
| ZINC000034781704 | -2.529 |
| ZINC000004074875 | -2.526 |
| ZINC000004074875 | -2.526 |
| ZINC000004577910 | -2.524 |
| ZINC000005456939 | -2.52 |
| ZINC000001530761 | -2.518 |
| ZINC000019632706 | -2.517 |
| ZINC000004097427 | -2.515 |
| ZINC000004097427 | -2.515 |
| ZINC000019144226 | -2.514 |
| ZINC000035653007 | -2.508 |
| ZINC000043202140 | -2.507 |
| ZINC000003977978 | -2.503 |
| ZINC000004212851 | -2.502 |
| ZINC000001853205 | -2.501 |
| ZINC000003873296 | -2.498 |
| ZINC000001530567 | -2.496 |
| ZINC000000537928 | -2.494 |
| ZINC000000896663 | -2.49 |
| ZINC000004641374 | -2.49 |
| ZINC000002522648 | -2.488 |
| ZINC000001532529 | -2.486 |
| ZINC000006745272 | -2.482 |
| ZINC000000006251 | -2.48 |
| ZINC000000006251 | -2.48 |
| ZINC000001530568 | -2.48 |
| ZINC000000897385 | -2.479 |
| ZINC000043195697 | -2.478 |
| ZINC000043195697 | -2.478 |
| ZINC000043195697 | -2.477 |
| ZINC000043195697 | -2.477 |
| ZINC000043195697 | -2.477 |
| ZINC000095619100 | -2.475 |
| ZINC000095619100 | -2.475 |
| ZINC000001842633 | -2.473 |
| ZINC000000000565 | -2.471 |
| ZINC000000000565 | -2.471 |
| ZINC000012402836 | -2.465 |
| ZINC000003830842 | -2.464 |
| ZINC000000057513 | -2.464 |
| ZINC000000057513 | -2.464 |
| ZINC000001546066 | -2.463 |
| ZINC000008214402 | -2.458 |
| ZINC000008214402 | -2.458 |
| ZINC000000596731 | -2.454 |
| ZINC000000596731 | -2.454 |
| ZINC000003079336 | -2.454 |
| ZINC000034608502 | -2.452 |
| ZINC000004097305 | -2.45 |
| ZINC000002005550 | -2.438 |
| ZINC000072316335 | -2.434 |
| ZINC000043194409 | -2.434 |
| ZINC000002005550 | -2.434 |
| ZINC000019203912 | -2.433 |
| ZINC000019203912 | -2.433 |
| ZINC000019203912 | -2.433 |
| ZINC000072316335 | -2.433 |
| ZINC000001530975 | -2.433 |
| ZINC000001530975 | -2.433 |
| ZINC000030691754 | -2.431 |
| ZINC000012484958 | -2.43 |
| ZINC000003915154 | -2.429 |
| ZINC000000000506 | -2.426 |
| ZINC000001532522 | -2.423 |
| ZINC000100003902 | -2.414 |
| ZINC000100003902 | -2.414 |
| ZINC000000000215 | -2.412 |
| ZINC000000000215 | -2.412 |
| ZINC000035902489 | -2.399 |
| ZINC000003876068 | -2.39 |
| ZINC000003876068 | -2.389 |
| ZINC000019632917 | -2.388 |
| ZINC000003792417 | -2.388 |
| ZINC000000601317 | -2.386 |
| ZINC000004083606 | -2.383 |
| ZINC000003992105 | -2.379 |
| ZINC000001530654 | -2.378 |
| ZINC000001530654 | -2.378 |
| ZINC000000592419 | -2.37 |
| ZINC000003871703 | -2.367 |
| ZINC000100070954 | -2.366 |
| ZINC000084400879 | -2.365 |
| ZINC000084400879 | -2.365 |
| ZINC000084400879 | -2.365 |
| ZINC000000113410 | -2.365 |
| ZINC000019796018 | -2.365 |
| ZINC000019796018 | -2.365 |
| ZINC000019796018 | -2.365 |
| ZINC000001530725 | -2.36 |
| ZINC000019361042 | -2.359 |
| ZINC000019361042 | -2.359 |
| ZINC000003874950 | -2.354 |
| ZINC000003874950 | -2.354 |
| ZINC000003930376 | -2.352 |
| ZINC000100001965 | -2.35 |
| ZINC000000537791 | -2.349 |
| ZINC000003807804 | -2.349 |
| ZINC000001530617 | -2.342 |
| ZINC000004474414 | -2.337 |
| ZINC000016159083 | -2.337 |
| ZINC000016159083 | -2.337 |
| ZINC000001530703 | -2.336 |
| ZINC000000608101 | -2.335 |
| ZINC000001530703 | -2.333 |
| ZINC000000155269 | -2.332 |
| ZINC000000155269 | -2.332 |
| ZINC000001851149 | -2.329 |
| ZINC000001851149 | -2.329 |
| ZINC000003875259 | -2.326 |
| ZINC000003875259 | -2.326 |
| ZINC000003830212 | -2.316 |
| ZINC000003830212 | -2.316 |
| ZINC000009302201 | -2.313 |
| ZINC000001530939 | -2.306 |
| ZINC000068202099 | -2.303 |
| ZINC000001530929 | -2.291 |
| ZINC000003876023 | -2.29 |
| ZINC000001530654 | -2.29 |
| ZINC000001530654 | -2.29 |
| ZINC000001538857 | -2.288 |
| ZINC000019594557 | -2.288 |
| ZINC000019594557 | -2.288 |
| ZINC000003881640 | -2.286 |
| ZINC000001530929 | -2.283 |
| ZINC000000538509 | -2.282 |
| ZINC000000538509 | -2.282 |
| ZINC000096942202 | -2.273 |
| ZINC000006716957 | -2.269 |
| ZINC000070466416 | -2.265 |
| ZINC000001530701 | -2.262 |
| ZINC000003875342 | -2.26 |
| ZINC000019632614 | -2.259 |
| ZINC000019632614 | -2.259 |
| ZINC000001530930 | -2.258 |
| ZINC000000968274 | -2.257 |
| ZINC000001530930 | -2.251 |
| ZINC000053683345 | -2.251 |
| ZINC000000000242 | -2.25 |
| ZINC000000000242 | -2.25 |
| ZINC000003794601 | -2.248 |
| ZINC000000896695 | -2.237 |
| ZINC000000896695 | -2.237 |
| ZINC000000968305 | -2.235 |
| ZINC000000968305 | -2.235 |
| ZINC000003830716 | -2.231 |
| ZINC000003830716 | -2.231 |
| ZINC000019364225 | -2.227 |
| ZINC000004097383 | -2.227 |
| ZINC000000538065 | -2.223 |
| ZINC000000538065 | -2.223 |
| ZINC000003873295 | -2.215 |
| ZINC000003938686 | -2.212 |
| ZINC000022056030 | -2.209 |
| ZINC000022056030 | -2.209 |
| ZINC000022056030 | -2.209 |
| ZINC000049036447 | -2.208 |
| ZINC000008214614 | -2.208 |
| ZINC000003938686 | -2.203 |
| ZINC000003800706 | -2.199 |
| ZINC000003805768 | -2.195 |
| ZINC000003805768 | -2.195 |
| ZINC000001542002 | -2.185 |
| ZINC000000606383 | -2.181 |
| ZINC000070466416 | -2.178 |
| ZINC000001530601 | -2.171 |
| ZINC000001996117 | -2.17 |
| ZINC000001996117 | -2.17 |
| ZINC000003831128 | -2.17 |
| ZINC000008220878 | -2.169 |
| ZINC000008220878 | -2.169 |
| ZINC000003008621 | -2.164 |
| ZINC000001850376 | -2.154 |
| ZINC000001530728 | -2.152 |
| ZINC000001530728 | -2.152 |
| ZINC000001548097 | -2.146 |
| ZINC000001548097 | -2.146 |
| ZINC000019364242 | -2.143 |
| ZINC000019364242 | -2.143 |
| ZINC000100022637 | -2.143 |
| ZINC000100016058 | -2.143 |
| ZINC000018324776 | -2.137 |
| ZINC000018324776 | -2.136 |
| ZINC000018324776 | -2.136 |
| ZINC000002032615 | -2.132 |
| ZINC000000895316 | -2.131 |
| ZINC000019632633 | -2.129 |
| ZINC000019632633 | -2.129 |
| ZINC000019632633 | -2.129 |
| ZINC000052509463 | -2.125 |
| ZINC000000895318 | -2.124 |
| ZINC000019632668 | -2.123 |
| ZINC000019632668 | -2.123 |
| ZINC000003931840 | -2.122 |
| ZINC000003931840 | -2.122 |
| ZINC000052509463 | -2.119 |
| ZINC000001530769 | -2.118 |
| ZINC000003976838 | -2.116 |
| ZINC000003976838 | -2.116 |
| ZINC000033943508 | -2.105 |
| ZINC000001850374 | -2.102 |
| ZINC000000601250 | -2.101 |
| ZINC000094566092 | -2.1 |
| ZINC000000014007 | -2.097 |
| ZINC000003800008 | -2.095 |
| ZINC000003800008 | -2.095 |
| ZINC000000896717 | -2.092 |
| ZINC000096942201 | -2.09 |
| ZINC000038945666 | -2.089 |
| ZINC000003990451 | -2.086 |
| ZINC000000538658 | -2.086 |
| ZINC000085432544 | -2.086 |
| ZINC000085432544 | -2.086 |
| ZINC000085432544 | -2.086 |
| ZINC000085432544 | -2.086 |
| ZINC000100013500 | -2.081 |
| ZINC000100013500 | -2.081 |
| ZINC000019144231 | -2.081 |
| ZINC000003872605 | -2.08 |
| ZINC000000584092 | -2.077 |
| ZINC000098023177 | -2.075 |
| ZINC000098023177 | -2.075 |
| ZINC000003800008 | -2.074 |
| ZINC000003938744 | -2.069 |
| ZINC000019632618 | -2.066 |
| ZINC000019632618 | -2.066 |
| ZINC000019632618 | -2.066 |
| ZINC000003872605 | -2.064 |
| ZINC000001530737 | -2.062 |
| ZINC000001530737 | -2.062 |
| ZINC000001530736 | -2.062 |
| ZINC000001530736 | -2.062 |
| ZINC000003927822 | -2.055 |
| ZINC000003927822 | -2.055 |
| ZINC000000596881 | -2.05 |
| ZINC000003932831 | -2.049 |
| ZINC000008214625 | -2.045 |
| ZINC000012503187 | -2.031 |
| ZINC000004212809 | -2.029 |
| ZINC000001571009 | -2.02 |
| ZINC000053229445 | -2.019 |
| ZINC000053229445 | -2.019 |
| ZINC000072318121 | -2.013 |
| ZINC000072318121 | -2.013 |
| ZINC000003871832 | -2.011 |
| ZINC000003871832 | -2.011 |
| ZINC000003843378 | -2.008 |
| ZINC000000643055 | -2.008 |
| ZINC000001530706 | -1.998 |
| ZINC000000621893 | -1.993 |
| ZINC000000621893 | -1.993 |
| ZINC000012503187 | -1.991 |
| ZINC000003872994 | -1.99 |
| ZINC000000607971 | -1.975 |
| ZINC000000155531 | -1.97 |
| ZINC000000155531 | -1.97 |
| ZINC000004474460 | -1.969 |
| ZINC000002522669 | -1.963 |
| ZINC000002522669 | -1.963 |
| ZINC000019632670 | -1.96 |
| ZINC000019632670 | -1.96 |
| ZINC000000895048 | -1.956 |
| ZINC000043202140 | -1.952 |
| ZINC000012503187 | -1.948 |
| ZINC000001850377 | -1.937 |
| ZINC000000402830 | -1.936 |
| ZINC000000402830 | -1.936 |
| ZINC000049933061 | -1.926 |
| ZINC000066166864 | -1.917 |
| ZINC000066166864 | -1.917 |
| ZINC000002019954 | -1.91 |
| ZINC000001530707 | -1.901 |
| ZINC000004097467 | -1.889 |
| ZINC000001530613 | -1.883 |
| ZINC000001530613 | -1.883 |
| ZINC000030691763 | -1.876 |
| ZINC000000004413 | -1.873 |
| ZINC000000004413 | -1.873 |
| ZINC000000896740 | -1.868 |
| ZINC000000897251 | -1.865 |
| ZINC000000897251 | -1.865 |
| ZINC000001530981 | -1.858 |
| ZINC000001530981 | -1.858 |
| ZINC000001530981 | -1.858 |
| ZINC000001319780 | -1.853 |
| ZINC000001319780 | -1.853 |
| ZINC000003972949 | -1.844 |
| ZINC000003972949 | -1.844 |
| ZINC000019364225 | -1.844 |
| ZINC000004655029 | -1.823 |
| ZINC000004655029 | -1.823 |
| ZINC000096014710 | -1.804 |
| ZINC000100022637 | -1.793 |
| ZINC000100016058 | -1.793 |
| ZINC000004095858 | -1.788 |
| ZINC000002019954 | -1.768 |
| ZINC000008214629 | -1.756 |
| ZINC000003977777 | -1.742 |
| ZINC000003824921 | -1.736 |
| ZINC000008214619 | -1.728 |
| ZINC000011680943 | -1.719 |
| ZINC000019144231 | -1.717 |
| ZINC000011680943 | -1.717 |
| ZINC000002016037 | -1.714 |
| ZINC000011681563 | -1.71 |
| ZINC000011681563 | -1.71 |
| ZINC000001542199 | -1.702 |
| ZINC000003873936 | -1.675 |
| ZINC000003873936 | -1.675 |
| ZINC000003925861 | -1.674 |
| ZINC000018115268 | -1.649 |
| ZINC000018115268 | -1.649 |
| ZINC000002019953 | -1.644 |
| ZINC000001529425 | -1.635 |
| ZINC000008035268 | -1.632 |
| ZINC000022448983 | -1.63 |
| ZINC000022448983 | -1.63 |
| ZINC000022448983 | -1.63 |
| ZINC000002019953 | -1.627 |
| ZINC000008214614 | -1.571 |
| ZINC000000113415 | -1.567 |
| ZINC000003831332 | -1.53 |
| ZINC000008214635 | -1.517 |
| ZINC000000643143 | -1.516 |
| ZINC000036701290 | -1.513 |
| ZINC000036701290 | -1.513 |
| ZINC000000643138 | -1.496 |
| ZINC000030690433 | -1.483 |
| ZINC000030690433 | -1.483 |
| ZINC000030690433 | -1.483 |
| ZINC000000001115 | -1.451 |
| ZINC000003938652 | -1.433 |
| ZINC000001530718 | -1.425 |
| ZINC000001532805 | -1.42 |
| ZINC000003798750 | -1.397 |
| ZINC000001530703 | -1.37 |
| ZINC000003920657 | -1.25 |
| ZINC000000403618 | -1.171 |
| ZINC000000000196 | -1.146 |
| ZINC000000000196 | -1.146 |
| ZINC000001530717 | -1.141 |
| ZINC000004474564 | -1.09 |
| ZINC000000901159 | -1.076 |
| ZINC000100014909 | -1.074 |
| ZINC000100014909 | -1.074 |
| ZINC000018115268 | -1.061 |
| ZINC000018115268 | -1.061 |
| ZINC000003830321 | -1.056 |
| ZINC000096903163 | -1.002 |
| ZINC000001530703 | -0.979 |
| ZINC000001530762 | -0.955 |
| ZINC000001530762 | -0.955 |
| ZINC000001543873 | -0.949 |
| ZINC000001543873 | -0.949 |
| ZINC000001530940 | -0.895 |
| ZINC000001530940 | -0.895 |
| ZINC000000538386 | -0.871 |
| ZINC000000538386 | -0.871 |
| ZINC000001530641 | -0.793 |
| ZINC000000057522 | -0.744 |
| ZINC000004474603 | -0.726 |
| ZINC000002016257 | -0.673 |
| ZINC000001530951 | -0.671 |
| ZINC000002016258 | -0.573 |
| ZINC000001530811 | -0.572 |
| ZINC000001530811 | -0.572 |
| ZINC000000896523 | -0.542 |
| ZINC000001530950 | -0.538 |
| ZINC000006845963 | -0.485 |
| ZINC000000000257 | -0.465 |
| ZINC000000538283 | -0.461 |
| ZINC000000538283 | -0.461 |
| ZINC000001530820 | -0.456 |
| ZINC000019594594 | -0.436 |
| ZINC000019594594 | -0.436 |
| ZINC000001633887 | -0.425 |
| ZINC000003785276 | -0.422 |
| ZINC000006920384 | -0.381 |
| ZINC000014881137 | -0.325 |
| ZINC000001532525 | -0.324 |
| ZINC000001633889 | -0.266 |
| ZINC000017285869 | -0.256 |
| ZINC000017285872 | -0.25 |
| ZINC000001530303 | -0.132 |
| ZINC000000601281 | -0.125 |
| ZINC000000601281 | -0.125 |
| ZINC000001612996 | 0.051 |
| ZINC000019364219 | 0.23 |
| ZINC000019364219 | 0.244 |
| ZINC000001554392 | 0.691 |
| ZINC000001554392 | 0.697 |
| ZINC000001532179 | 1.49 |
| ZINC000001531036 | 1.834 |
| ZINC000019363537 | 2.059 |
| ZINC000019363537 | 2.061 |
| ZINC000006845860 | 2.206 |
| ZINC000004978673 | 2.681 |
| ZINC000000896409 | 3.023 |
| ZINC000000896409 | 3.023 |
| ZINC000008437287 | 3.451 |

**Table S17**. The results of structure-based virtual screening for SEPHS1.

| Title | Docking score |
| --- | --- |
| ZINC000085537014 | -11.148 |
| ZINC000085537014 | -11.148 |
| ZINC000014210876 | -10.664 |
| ZINC000085540219 | -10.604 |
| ZINC000003951740 | -10.425 |
| ZINC000008214418 | -10.342 |
| ZINC000034636383 | -10.313 |
| ZINC000001530639 | -10.199 |
| ZINC000014210876 | -10.097 |
| ZINC000003872994 | -10.043 |
| ZINC000001995484 | -9.946 |
| ZINC000001995484 | -9.906 |
| ZINC000003830960 | -9.816 |
| ZINC000001886617 | -9.647 |
| ZINC000001481815 | -9.603 |
| ZINC000001481815 | -9.603 |
| ZINC000001535101 | -9.578 |
| ZINC000000596881 | -9.558 |
| ZINC000003792417 | -9.497 |
| ZINC000000601250 | -9.472 |
| ZINC000009164421 | -9.412 |
| ZINC000085540223 | -9.394 |
| ZINC000085540215 | -9.373 |
| ZINC000003913937 | -9.329 |
| ZINC000000896740 | -9.278 |
| ZINC000001530922 | -9.172 |
| ZINC000001530922 | -9.172 |
| ZINC000001530922 | -9.172 |
| ZINC000000000416 | -9.154 |
| ZINC000003812851 | -9.151 |
| ZINC000000000416 | -9.148 |
| ZINC000003812851 | -9.147 |
| ZINC000003812863 | -9.147 |
| ZINC000001530973 | -9.141 |
| ZINC000002016037 | -9.14 |
| ZINC000001543916 | -9.125 |
| ZINC000001543916 | -9.12 |
| ZINC000000403011 | -9.112 |
| ZINC000003812863 | -9.111 |
| ZINC000043100953 | -9.111 |
| ZINC000043100953 | -9.111 |
| ZINC000043100953 | -9.111 |
| ZINC000000403011 | -9.103 |
| ZINC000000004319 | -9.102 |
| ZINC000000004319 | -9.1 |
| ZINC000000403010 | -9.09 |
| ZINC000000403010 | -9.087 |
| ZINC000021982937 | -9.075 |
| ZINC000021982937 | -9.075 |
| ZINC000000606383 | -8.997 |
| ZINC000012468792 | -8.987 |
| ZINC000003781943 | -8.969 |
| ZINC000003873371 | -8.956 |
| ZINC000003873371 | -8.956 |
| ZINC000003871978 | -8.927 |
| ZINC000009212427 | -8.916 |
| ZINC000003801163 | -8.913 |
| ZINC000000643055 | -8.913 |
| ZINC000009212427 | -8.91 |
| ZINC000009212427 | -8.907 |
| ZINC000003819392 | -8.877 |
| ZINC000002005305 | -8.866 |
| ZINC000016052277 | -8.831 |
| ZINC000016052277 | -8.831 |
| ZINC000000607971 | -8.827 |
| ZINC000016052277 | -8.81 |
| ZINC000002005305 | -8.794 |
| ZINC000000004448 | -8.777 |
| ZINC000003791297 | -8.768 |
| ZINC000001530974 | -8.755 |
| ZINC000002568036 | -8.747 |
| ZINC000002568036 | -8.747 |
| ZINC000252679615 | -8.747 |
| ZINC000252679615 | -8.747 |
| ZINC000007997966 | -8.747 |
| ZINC000007997966 | -8.747 |
| ZINC000029571072 | -8.741 |
| ZINC000000897256 | -8.729 |
| ZINC000000897256 | -8.728 |
| ZINC000000537891 | -8.713 |
| ZINC000000537891 | -8.713 |
| ZINC000003873295 | -8.712 |
| ZINC000001530713 | -8.71 |
| ZINC000001530713 | -8.705 |
| ZINC000003873296 | -8.705 |
| ZINC000009212428 | -8.689 |
| ZINC000003871967 | -8.683 |
| ZINC000003830958 | -8.675 |
| ZINC000003830218 | -8.674 |
| ZINC000003830218 | -8.662 |
| ZINC000001534965 | -8.662 |
| ZINC000000643143 | -8.638 |
| ZINC000000897385 | -8.615 |
| ZINC000033965961 | -8.606 |
| ZINC000033965961 | -8.606 |
| ZINC000029571072 | -8.6 |
| ZINC000003819138 | -8.582 |
| ZINC000003860453 | -8.581 |
| ZINC000085537017 | -8.575 |
| ZINC000013682481 | -8.569 |
| ZINC000009212427 | -8.566 |
| ZINC000011677857 | -8.543 |
| ZINC000033965961 | -8.514 |
| ZINC000033965961 | -8.514 |
| ZINC000033965961 | -8.514 |
| ZINC000003830449 | -8.51 |
| ZINC000003813061 | -8.507 |
| ZINC000035801098 | -8.506 |
| ZINC000035801098 | -8.504 |
| ZINC000005844788 | -8.501 |
| ZINC000003916214 | -8.478 |
| ZINC000003916214 | -8.478 |
| ZINC000085537017 | -8.474 |
| ZINC000085537017 | -8.474 |
| ZINC000019364229 | -8.467 |
| ZINC000019364229 | -8.467 |
| ZINC000019364229 | -8.467 |
| ZINC000022448097 | -8.466 |
| ZINC000022448097 | -8.466 |
| ZINC000022448097 | -8.466 |
| ZINC000052716421 | -8.464 |
| ZINC000052716421 | -8.464 |
| ZINC000000538273 | -8.463 |
| ZINC000000538273 | -8.463 |
| ZINC000003871703 | -8.462 |
| ZINC000002599970 | -8.444 |
| ZINC000003798757 | -8.443 |
| ZINC000002599970 | -8.442 |
| ZINC000027428713 | -8.434 |
| ZINC000003964126 | -8.433 |
| ZINC000027428713 | -8.415 |
| ZINC000002005305 | -8.399 |
| ZINC000019796080 | -8.398 |
| ZINC000019796080 | -8.398 |
| ZINC000019796080 | -8.398 |
| ZINC000000608101 | -8.379 |
| ZINC000001853205 | -8.341 |
| ZINC000009212427 | -8.338 |
| ZINC000009212428 | -8.326 |
| ZINC000001540998 | -8.326 |
| ZINC000009212428 | -8.306 |
| ZINC000009212428 | -8.305 |
| ZINC000000075126 | -8.286 |
| ZINC000003873160 | -8.284 |
| ZINC000003873160 | -8.284 |
| ZINC000013682481 | -8.283 |
| ZINC000004228257 | -8.28 |
| ZINC000003830959 | -8.28 |
| ZINC000003922770 | -8.272 |
| ZINC000002005305 | -8.272 |
| ZINC000004228258 | -8.271 |
| ZINC000004228258 | -8.271 |
| ZINC000005844788 | -8.264 |
| ZINC000004228258 | -8.24 |
| ZINC000000001735 | -8.23 |
| ZINC000043100709 | -8.223 |
| ZINC000004228257 | -8.221 |
| ZINC000002005305 | -8.211 |
| ZINC000003810860 | -8.209 |
| ZINC000003830957 | -8.207 |
| ZINC000000601316 | -8.198 |
| ZINC000019364230 | -8.19 |
| ZINC000019364230 | -8.19 |
| ZINC000019364230 | -8.19 |
| ZINC000242437512 | -8.189 |
| ZINC000009212427 | -8.188 |
| ZINC000000896731 | -8.183 |
| ZINC000000896731 | -8.17 |
| ZINC000011616925 | -8.167 |
| ZINC000002036848 | -8.161 |
| ZINC000002036848 | -8.161 |
| ZINC000005844792 | -8.158 |
| ZINC000005844792 | -8.158 |
| ZINC000004474405 | -8.144 |
| ZINC000002005305 | -8.135 |
| ZINC000013585233 | -8.113 |
| ZINC000022447798 | -8.107 |
| ZINC000022447798 | -8.107 |
| ZINC000021982937 | -8.104 |
| ZINC000021982937 | -8.104 |
| ZINC000021982937 | -8.104 |
| ZINC000000643138 | -8.101 |
| ZINC000000897258 | -8.1 |
| ZINC000013648755 | -8.098 |
| ZINC000038945666 | -8.093 |
| ZINC000001530555 | -8.092 |
| ZINC000013648755 | -8.089 |
| ZINC000000897258 | -8.089 |
| ZINC000003830215 | -8.087 |
| ZINC000003830215 | -8.08 |
| ZINC000003785268 | -8.078 |
| ZINC000003813083 | -8.068 |
| ZINC000029319828 | -8.059 |
| ZINC000000643153 | -8.051 |
| ZINC000001536109 | -8.048 |
| ZINC000004213946 | -8.048 |
| ZINC000004213946 | -8.042 |
| ZINC000000538564 | -8.041 |
| ZINC000000005823 | -8.035 |
| ZINC000029319828 | -8.031 |
| ZINC000000895154 | -8.025 |
| ZINC000000895154 | -8.02 |
| ZINC000003871701 | -8.016 |
| ZINC000100001964 | -8.006 |
| ZINC000003871960 | -7.998 |
| ZINC000003812306 | -7.998 |
| ZINC000003830212 | -7.997 |
| ZINC000003830212 | -7.997 |
| ZINC000002539827 | -7.992 |
| ZINC000003779042 | -7.986 |
| ZINC000003918453 | -7.961 |
| ZINC000013986658 | -7.957 |
| ZINC000014879992 | -7.956 |
| ZINC000000000850 | -7.952 |
| ZINC000000000850 | -7.952 |
| ZINC000003806262 | -7.946 |
| ZINC000003806262 | -7.946 |
| ZINC000000538483 | -7.941 |
| ZINC000000538483 | -7.941 |
| ZINC000043202140 | -7.933 |
| ZINC000000000856 | -7.928 |
| ZINC000000896958 | -7.919 |
| ZINC000003830993 | -7.912 |
| ZINC000011681534 | -7.911 |
| ZINC000000000856 | -7.911 |
| ZINC000043202140 | -7.908 |
| ZINC000003830993 | -7.907 |
| ZINC000000001899 | -7.903 |
| ZINC000001530636 | -7.891 |
| ZINC000009212428 | -7.889 |
| ZINC000009212428 | -7.889 |
| ZINC000004228257 | -7.887 |
| ZINC000028957444 | -7.885 |
| ZINC000004097309 | -7.883 |
| ZINC000012466082 | -7.869 |
| ZINC000252678020 | -7.869 |
| ZINC000012466082 | -7.867 |
| ZINC000252678020 | -7.867 |
| ZINC000002008866 | -7.848 |
| ZINC000100296828 | -7.84 |
| ZINC000100296828 | -7.84 |
| ZINC000003977764 | -7.825 |
| ZINC000014879992 | -7.821 |
| ZINC000000005423 | -7.818 |
| ZINC000003807804 | -7.807 |
| ZINC000003830579 | -7.806 |
| ZINC000031274852 | -7.801 |
| ZINC000031274852 | -7.801 |
| ZINC000001530760 | -7.797 |
| ZINC000001481956 | -7.794 |
| ZINC000001481956 | -7.794 |
| ZINC000000895154 | -7.782 |
| ZINC000013986658 | -7.781 |
| ZINC000002539827 | -7.777 |
| ZINC000002539827 | -7.773 |
| ZINC000003831282 | -7.767 |
| ZINC000000001505 | -7.754 |
| ZINC000000020243 | -7.752 |
| ZINC000014210642 | -7.747 |
| ZINC000000607986 | -7.739 |
| ZINC000006716957 | -7.738 |
| ZINC000000005823 | -7.738 |
| ZINC000014210642 | -7.734 |
| ZINC000000001317 | -7.73 |
| ZINC000000014864 | -7.727 |
| ZINC000084668739 | -7.719 |
| ZINC000095626706 | -7.717 |
| ZINC000030691736 | -7.713 |
| ZINC000100074252 | -7.705 |
| ZINC000006467621 | -7.693 |
| ZINC000038197764 | -7.69 |
| ZINC000000001758 | -7.686 |
| ZINC000000020220 | -7.672 |
| ZINC000000020220 | -7.669 |
| ZINC000000607986 | -7.668 |
| ZINC000116473771 | -7.662 |
| ZINC000000538621 | -7.649 |
| ZINC000004214700 | -7.639 |
| ZINC000004214700 | -7.639 |
| ZINC000003875439 | -7.629 |
| ZINC000084589076 | -7.629 |
| ZINC000003830999 | -7.628 |
| ZINC000001999441 | -7.627 |
| ZINC000000601305 | -7.627 |
| ZINC000000011012 | -7.627 |
| ZINC000001999441 | -7.623 |
| ZINC000013818943 | -7.606 |
| ZINC000003794794 | -7.604 |
| ZINC000000538621 | -7.604 |
| ZINC000100008319 | -7.604 |
| ZINC000003830713 | -7.599 |
| ZINC000003820029 | -7.595 |
| ZINC000014879992 | -7.594 |
| ZINC000014879992 | -7.594 |
| ZINC000003830999 | -7.593 |
| ZINC000038197764 | -7.583 |
| ZINC000053084692 | -7.581 |
| ZINC000003812869 | -7.581 |
| ZINC000053084692 | -7.58 |
| ZINC000019632628 | -7.58 |
| ZINC000019632628 | -7.58 |
| ZINC000000537957 | -7.568 |
| ZINC000007997897 | -7.565 |
| ZINC000007997897 | -7.565 |
| ZINC000007997897 | -7.565 |
| ZINC000005819214 | -7.564 |
| ZINC000003808779 | -7.564 |
| ZINC000003786192 | -7.563 |
| ZINC000002570817 | -7.557 |
| ZINC000000537822 | -7.546 |
| ZINC000000537822 | -7.546 |
| ZINC000000003911 | -7.546 |
| ZINC000003830947 | -7.545 |
| ZINC000000004009 | -7.54 |
| ZINC000000538550 | -7.533 |
| ZINC000000538550 | -7.533 |
| ZINC000003818726 | -7.53 |
| ZINC000013912394 | -7.522 |
| ZINC000100296832 | -7.518 |
| ZINC000100296832 | -7.518 |
| ZINC000003818726 | -7.509 |
| ZINC000003607120 | -7.504 |
| ZINC000004655029 | -7.5 |
| ZINC000004655029 | -7.5 |
| ZINC000000000746 | -7.498 |
| ZINC000003607120 | -7.491 |
| ZINC000003812869 | -7.485 |
| ZINC000000005151 | -7.481 |
| ZINC000000005151 | -7.481 |
| ZINC000005733652 | -7.474 |
| ZINC000005733652 | -7.474 |
| ZINC000005733652 | -7.474 |
| ZINC000005733652 | -7.474 |
| ZINC000242437511 | -7.468 |
| ZINC000000057278 | -7.468 |
| ZINC000116473771 | -7.449 |
| ZINC000001690324 | -7.447 |
| ZINC000001690324 | -7.447 |
| ZINC000001529323 | -7.443 |
| ZINC000003982483 | -7.441 |
| ZINC000003982483 | -7.441 |
| ZINC000003982483 | -7.441 |
| ZINC000013597823 | -7.436 |
| ZINC000084758479 | -7.431 |
| ZINC000084758479 | -7.431 |
| ZINC000030691797 | -7.43 |
| ZINC000013597823 | -7.425 |
| ZINC000222731806 | -7.42 |
| ZINC000003918138 | -7.418 |
| ZINC000000002281 | -7.414 |
| ZINC000036520252 | -7.407 |
| ZINC000000601283 | -7.397 |
| ZINC000000897222 | -7.394 |
| ZINC000000005560 | -7.383 |
| ZINC000116473771 | -7.38 |
| ZINC000000002273 | -7.375 |
| ZINC000003927870 | -7.371 |
| ZINC000003927870 | -7.371 |
| ZINC000001530948 | -7.363 |
| ZINC000001530636 | -7.361 |
| ZINC000012503177 | -7.357 |
| ZINC000035902489 | -7.348 |
| ZINC000001546066 | -7.34 |
| ZINC000003812865 | -7.339 |
| ZINC000000049153 | -7.338 |
| ZINC000000601250 | -7.33 |
| ZINC000003830264 | -7.327 |
| ZINC000019364222 | -7.323 |
| ZINC000019364222 | -7.323 |
| ZINC000019364222 | -7.323 |
| ZINC000000601229 | -7.314 |
| ZINC000000601229 | -7.314 |
| ZINC000000601229 | -7.314 |
| ZINC000049643479 | -7.313 |
| ZINC000012360535 | -7.31 |
| ZINC000001530975 | -7.296 |
| ZINC000001530975 | -7.296 |
| ZINC000003873160 | -7.294 |
| ZINC000001550477 | -7.294 |
| ZINC000003873160 | -7.29 |
| ZINC000005162311 | -7.278 |
| ZINC000004475353 | -7.273 |
| ZINC000002522669 | -7.272 |
| ZINC000002522669 | -7.272 |
| ZINC000002016037 | -7.271 |
| ZINC000000120286 | -7.269 |
| ZINC000000602128 | -7.263 |
| ZINC000012503099 | -7.258 |
| ZINC000012503099 | -7.258 |
| ZINC000084757007 | -7.256 |
| ZINC000000509440 | -7.256 |
| ZINC000000121541 | -7.248 |
| ZINC000013512456 | -7.247 |
| ZINC000000608101 | -7.238 |
| ZINC000018203737 | -7.235 |
| ZINC000018203737 | -7.235 |
| ZINC000100022637 | -7.23 |
| ZINC000100016058 | -7.23 |
| ZINC000003776633 | -7.227 |
| ZINC000003776633 | -7.227 |
| ZINC000008855117 | -7.219 |
| ZINC000000034157 | -7.216 |
| ZINC000008855117 | -7.216 |
| ZINC000001530654 | -7.213 |
| ZINC000001530654 | -7.213 |
| ZINC000000002272 | -7.212 |
| ZINC000001543475 | -7.209 |
| ZINC000001543475 | -7.209 |
| ZINC000001543475 | -7.209 |
| ZINC000100001965 | -7.208 |
| ZINC000000968301 | -7.207 |
| ZINC000003827556 | -7.205 |
| ZINC000008577218 | -7.205 |
| ZINC000003800475 | -7.204 |
| ZINC000003794601 | -7.203 |
| ZINC000001536779 | -7.193 |
| ZINC000100036924 | -7.192 |
| ZINC000000004028 | -7.19 |
| ZINC000000004028 | -7.19 |
| ZINC000000020240 | -7.179 |
| ZINC000013597823 | -7.178 |
| ZINC000013597823 | -7.178 |
| ZINC000022059268 | -7.177 |
| ZINC000100022637 | -7.176 |
| ZINC000100016058 | -7.176 |
| ZINC000002169830 | -7.175 |
| ZINC000013587680 | -7.172 |
| ZINC000072267023 | -7.166 |
| ZINC000072267023 | -7.166 |
| ZINC000000006156 | -7.16 |
| ZINC000004474443 | -7.149 |
| ZINC000028973446 | -7.144 |
| ZINC000030691754 | -7.142 |
| ZINC000012859773 | -7.142 |
| ZINC000000001408 | -7.135 |
| ZINC000000896819 | -7.131 |
| ZINC000003802690 | -7.129 |
| ZINC000001035331 | -7.129 |
| ZINC000034608502 | -7.125 |
| ZINC000013986658 | -7.123 |
| ZINC000034676245 | -7.122 |
| ZINC000034676245 | -7.122 |
| ZINC000019364224 | -7.121 |
| ZINC000019364224 | -7.121 |
| ZINC000019364224 | -7.121 |
| ZINC000013986658 | -7.115 |
| ZINC000004474682 | -7.113 |
| ZINC000022448696 | -7.112 |
| ZINC000022448696 | -7.112 |
| ZINC000022448696 | -7.112 |
| ZINC000022448696 | -7.112 |
| ZINC000022448696 | -7.112 |
| ZINC000019632614 | -7.107 |
| ZINC000019632614 | -7.107 |
| ZINC000000057624 | -7.104 |
| ZINC000001530759 | -7.101 |
| ZINC000000002279 | -7.095 |
| ZINC000000003876 | -7.085 |
| ZINC000013537284 | -7.085 |
| ZINC000003927870 | -7.083 |
| ZINC000000113382 | -7.083 |
| ZINC000000113382 | -7.082 |
| ZINC000003776970 | -7.077 |
| ZINC000008855117 | -7.074 |
| ZINC000008855117 | -7.073 |
| ZINC000003776970 | -7.072 |
| ZINC000100378061 | -7.068 |
| ZINC000100378061 | -7.068 |
| ZINC000100378061 | -7.068 |
| ZINC000000538627 | -7.063 |
| ZINC000000004076 | -7.059 |
| ZINC000006716957 | -7.056 |
| ZINC000000538065 | -7.043 |
| ZINC000000538065 | -7.043 |
| ZINC000000518554 | -7.038 |
| ZINC000028973441 | -7.037 |
| ZINC000003927198 | -7.034 |
| ZINC000004474443 | -7.029 |
| ZINC000003823492 | -7.026 |
| ZINC000004095696 | -7.021 |
| ZINC000000009342 | -7.02 |
| ZINC000000009342 | -7.02 |
| ZINC000011677837 | -7.02 |
| ZINC000001842633 | -7.018 |
| ZINC000003952881 | -7.015 |
| ZINC000000001958 | -7.014 |
| ZINC000003842753 | -7.009 |
| ZINC000003842753 | -7.008 |
| ZINC000003798247 | -7.005 |
| ZINC000016159083 | -7.004 |
| ZINC000016159083 | -7.004 |
| ZINC000022016981 | -6.997 |
| ZINC000022016981 | -6.997 |
| ZINC000022016981 | -6.997 |
| ZINC000013831130 | -6.996 |
| ZINC000001843099 | -6.994 |
| ZINC000012495062 | -6.982 |
| ZINC000000606383 | -6.982 |
| ZINC000013831130 | -6.978 |
| ZINC000000896543 | -6.977 |
| ZINC000022016976 | -6.977 |
| ZINC000022016976 | -6.977 |
| ZINC000022016976 | -6.977 |
| ZINC000100017856 | -6.966 |
| ZINC000100017856 | -6.966 |
| ZINC000001530947 | -6.961 |
| ZINC000019228902 | -6.959 |
| ZINC000019228902 | -6.959 |
| ZINC000019228902 | -6.959 |
| ZINC000084758235 | -6.955 |
| ZINC000000004724 | -6.947 |
| ZINC000002000707 | -6.946 |
| ZINC000000113428 | -6.941 |
| ZINC000001530886 | -6.937 |
| ZINC000002519740 | -6.937 |
| ZINC000002519740 | -6.937 |
| ZINC000002519740 | -6.937 |
| ZINC000003861768 | -6.934 |
| ZINC000001530571 | -6.929 |
| ZINC000001530571 | -6.929 |
| ZINC000000004166 | -6.928 |
| ZINC000000896463 | -6.927 |
| ZINC000028467879 | -6.907 |
| ZINC000028467879 | -6.907 |
| ZINC000000538312 | -6.904 |
| ZINC000000538312 | -6.904 |
| ZINC000014210455 | -6.894 |
| ZINC000003991624 | -6.885 |
| ZINC000013537284 | -6.875 |
| ZINC000003813088 | -6.874 |
| ZINC000006021033 | -6.869 |
| ZINC000100004343 | -6.868 |
| ZINC000019632670 | -6.866 |
| ZINC000019632670 | -6.866 |
| ZINC000011681563 | -6.862 |
| ZINC000011681563 | -6.862 |
| ZINC000002570895 | -6.856 |
| ZINC000008403947 | -6.854 |
| ZINC000003782818 | -6.842 |
| ZINC000003782818 | -6.842 |
| ZINC000000895360 | -6.841 |
| ZINC000001530776 | -6.829 |
| ZINC000000001850 | -6.826 |
| ZINC000003831531 | -6.826 |
| ZINC000003831531 | -6.826 |
| ZINC000000538275 | -6.825 |
| ZINC000000538275 | -6.825 |
| ZINC000100006264 | -6.823 |
| ZINC000004175630 | -6.822 |
| ZINC000004175630 | -6.822 |
| ZINC000003876186 | -6.821 |
| ZINC000000901736 | -6.819 |
| ZINC000084441937 | -6.817 |
| ZINC000003823475 | -6.812 |
| ZINC000003823475 | -6.812 |
| ZINC000003823475 | -6.812 |
| ZINC000001996784 | -6.809 |
| ZINC000003986735 | -6.804 |
| ZINC000011681534 | -6.802 |
| ZINC000003800706 | -6.797 |
| ZINC000003986735 | -6.797 |
| ZINC000003986735 | -6.797 |
| ZINC000035342789 | -6.793 |
| ZINC000003874185 | -6.78 |
| ZINC000043207238 | -6.775 |
| ZINC000242437513 | -6.771 |
| ZINC000006627681 | -6.77 |
| ZINC000000643114 | -6.767 |
| ZINC000000057147 | -6.763 |
| ZINC000019632668 | -6.762 |
| ZINC000019632668 | -6.762 |
| ZINC000000002688 | -6.761 |
| ZINC000000000373 | -6.76 |
| ZINC000003872931 | -6.759 |
| ZINC000003872931 | -6.759 |
| ZINC000000607971 | -6.758 |
| ZINC000000537795 | -6.756 |
| ZINC000004676424 | -6.755 |
| ZINC000004097310 | -6.749 |
| ZINC000001485935 | -6.747 |
| ZINC000001530973 | -6.745 |
| ZINC000011679756 | -6.744 |
| ZINC000001538857 | -6.742 |
| ZINC000000621893 | -6.74 |
| ZINC000000621893 | -6.74 |
| ZINC000000020230 | -6.738 |
| ZINC000001530981 | -6.734 |
| ZINC000001530981 | -6.734 |
| ZINC000001530981 | -6.734 |
| ZINC000003936683 | -6.734 |
| ZINC000003936683 | -6.734 |
| ZINC000011679756 | -6.733 |
| ZINC000001530638 | -6.729 |
| ZINC000003861599 | -6.726 |
| ZINC000006661227 | -6.724 |
| ZINC000006661227 | -6.724 |
| ZINC000006661227 | -6.724 |
| ZINC000022010649 | -6.723 |
| ZINC000000020251 | -6.722 |
| ZINC000003795098 | -6.718 |
| ZINC000003861806 | -6.715 |
| ZINC000003784120 | -6.714 |
| ZINC000003875357 | -6.708 |
| ZINC000003830441 | -6.706 |
| ZINC000000000931 | -6.705 |
| ZINC000000000931 | -6.705 |
| ZINC000000601254 | -6.704 |
| ZINC000000005823 | -6.695 |
| ZINC000000608382 | -6.692 |
| ZINC000000608382 | -6.692 |
| ZINC000004099200 | -6.686 |
| ZINC000002015035 | -6.684 |
| ZINC000002015035 | -6.684 |
| ZINC000003872605 | -6.684 |
| ZINC000000006310 | -6.683 |
| ZINC000014210876 | -6.668 |
| ZINC000019632834 | -6.667 |
| ZINC000004216238 | -6.667 |
| ZINC000034089131 | -6.666 |
| ZINC000003872605 | -6.665 |
| ZINC000003816514 | -6.665 |
| ZINC000095452610 | -6.664 |
| ZINC000000018635 | -6.66 |
| ZINC000003816514 | -6.66 |
| ZINC000001552908 | -6.655 |
| ZINC000000000565 | -6.655 |
| ZINC000000000565 | -6.655 |
| ZINC000000004321 | -6.65 |
| ZINC000022059926 | -6.65 |
| ZINC000000035804 | -6.649 |
| ZINC000003798064 | -6.648 |
| ZINC000003813078 | -6.647 |
| ZINC000034220093 | -6.646 |
| ZINC000000896740 | -6.645 |
| ZINC000019632834 | -6.644 |
| ZINC000019632834 | -6.644 |
| ZINC000001542392 | -6.634 |
| ZINC000003873296 | -6.63 |
| ZINC000001531009 | -6.626 |
| ZINC000001531009 | -6.626 |
| ZINC000000901791 | -6.626 |
| ZINC000035653009 | -6.625 |
| ZINC000000001984 | -6.624 |
| ZINC000003831477 | -6.613 |
| ZINC000014210876 | -6.613 |
| ZINC000001531009 | -6.611 |
| ZINC000000007295 | -6.61 |
| ZINC000000896546 | -6.61 |
| ZINC000000896546 | -6.608 |
| ZINC000000000346 | -6.605 |
| ZINC000000538266 | -6.605 |
| ZINC000022116608 | -6.603 |
| ZINC000013129998 | -6.603 |
| ZINC000085205448 | -6.602 |
| ZINC000018279854 | -6.6 |
| ZINC000000596881 | -6.599 |
| ZINC000012414057 | -6.599 |
| ZINC000003976838 | -6.597 |
| ZINC000003976838 | -6.597 |
| ZINC000022116608 | -6.592 |
| ZINC000000008492 | -6.588 |
| ZINC000003940470 | -6.587 |
| ZINC000000001688 | -6.585 |
| ZINC000000001688 | -6.585 |
| ZINC000000013156 | -6.582 |
| ZINC000006036847 | -6.579 |
| ZINC000003929022 | -6.578 |
| ZINC000019796168 | -6.575 |
| ZINC000019796168 | -6.575 |
| ZINC000019796168 | -6.575 |
| ZINC000021303210 | -6.574 |
| ZINC000004693575 | -6.574 |
| ZINC000000020248 | -6.571 |
| ZINC000000000922 | -6.566 |
| ZINC000094566093 | -6.566 |
| ZINC000000012346 | -6.564 |
| ZINC000001530706 | -6.563 |
| ZINC000096942201 | -6.562 |
| ZINC000009302239 | -6.555 |
| ZINC000001530697 | -6.551 |
| ZINC000001530697 | -6.551 |
| ZINC000001530697 | -6.551 |
| ZINC000000005823 | -6.55 |
| ZINC000001482049 | -6.547 |
| ZINC000001482049 | -6.547 |
| ZINC000000025958 | -6.541 |
| ZINC000000643046 | -6.54 |
| ZINC000003806104 | -6.534 |
| ZINC000003782818 | -6.531 |
| ZINC000003782818 | -6.531 |
| ZINC000002539702 | -6.529 |
| ZINC000003803652 | -6.528 |
| ZINC000001530974 | -6.527 |
| ZINC000022059930 | -6.526 |
| ZINC000000000507 | -6.526 |
| ZINC000000000507 | -6.526 |
| ZINC000001530599 | -6.523 |
| ZINC000003875980 | -6.52 |
| ZINC000019168887 | -6.52 |
| ZINC000003830891 | -6.514 |
| ZINC000049036447 | -6.509 |
| ZINC000043206370 | -6.507 |
| ZINC000000057464 | -6.507 |
| ZINC000001530580 | -6.506 |
| ZINC000051951647 | -6.503 |
| ZINC000003938704 | -6.5 |
| ZINC000001530580 | -6.5 |
| ZINC000013911941 | -6.5 |
| ZINC000052509366 | -6.496 |
| ZINC000004658553 | -6.494 |
| ZINC000001530707 | -6.494 |
| ZINC000000000096 | -6.49 |
| ZINC000052509366 | -6.49 |
| ZINC000000056652 | -6.488 |
| ZINC000003953037 | -6.486 |
| ZINC000006733300 | -6.486 |
| ZINC000001530599 | -6.484 |
| ZINC000001530599 | -6.484 |
| ZINC000001530599 | -6.482 |
| ZINC000007997952 | -6.473 |
| ZINC000007997952 | -6.473 |
| ZINC000000601301 | -6.471 |
| ZINC000001997125 | -6.464 |
| ZINC000013585233 | -6.463 |
| ZINC000004340269 | -6.461 |
| ZINC000000002043 | -6.456 |
| ZINC000000002043 | -6.456 |
| ZINC000004340269 | -6.454 |
| ZINC000084400879 | -6.454 |
| ZINC000084400879 | -6.454 |
| ZINC000084400879 | -6.454 |
| ZINC000013512456 | -6.451 |
| ZINC000022448983 | -6.442 |
| ZINC000022448983 | -6.442 |
| ZINC000022448983 | -6.442 |
| ZINC000001530695 | -6.441 |
| ZINC000001530695 | -6.441 |
| ZINC000001530695 | -6.441 |
| ZINC000003802417 | -6.439 |
| ZINC000003802417 | -6.439 |
| ZINC000003831475 | -6.437 |
| ZINC000003604264 | -6.436 |
| ZINC000003775644 | -6.435 |
| ZINC000006661227 | -6.431 |
| ZINC000006661227 | -6.431 |
| ZINC000019362737 | -6.429 |
| ZINC000019362737 | -6.429 |
| ZINC000019362737 | -6.429 |
| ZINC000022116612 | -6.429 |
| ZINC000018203737 | -6.428 |
| ZINC000018203737 | -6.428 |
| ZINC000000119717 | -6.427 |
| ZINC000022851765 | -6.424 |
| ZINC000022851765 | -6.424 |
| ZINC000022851765 | -6.424 |
| ZINC000003929508 | -6.424 |
| ZINC000006661227 | -6.42 |
| ZINC000003797541 | -6.42 |
| ZINC000001530935 | -6.418 |
| ZINC000000968336 | -6.411 |
| ZINC000000002191 | -6.411 |
| ZINC000040430143 | -6.41 |
| ZINC000003813010 | -6.406 |
| ZINC000003813010 | -6.405 |
| ZINC000000013156 | -6.405 |
| ZINC000094566092 | -6.403 |
| ZINC000003831474 | -6.401 |
| ZINC000018516586 | -6.4 |
| ZINC000030691727 | -6.397 |
| ZINC000003925861 | -6.39 |
| ZINC000000020255 | -6.388 |
| ZINC000000001644 | -6.384 |
| ZINC000000643055 | -6.381 |
| ZINC000000537752 | -6.379 |
| ZINC000000537752 | -6.379 |
| ZINC000000968274 | -6.378 |
| ZINC000000895302 | -6.375 |
| ZINC000004097426 | -6.374 |
| ZINC000004097426 | -6.374 |
| ZINC000004097426 | -6.374 |
| ZINC000000007673 | -6.367 |
| ZINC000000057146 | -6.366 |
| ZINC000000968303 | -6.363 |
| ZINC000001530427 | -6.361 |
| ZINC000001530427 | -6.361 |
| ZINC000001530427 | -6.361 |
| ZINC000003953037 | -6.361 |
| ZINC000000896595 | -6.36 |
| ZINC000000000693 | -6.356 |
| ZINC000003874715 | -6.351 |
| ZINC000003830635 | -6.347 |
| ZINC000000895199 | -6.345 |
| ZINC000000895457 | -6.345 |
| ZINC000001530637 | -6.343 |
| ZINC000000002028 | -6.339 |
| ZINC000001547851 | -6.334 |
| ZINC000016929327 | -6.333 |
| ZINC000000897089 | -6.332 |
| ZINC000003920355 | -6.332 |
| ZINC000003830405 | -6.332 |
| ZINC000000967597 | -6.331 |
| ZINC000003775644 | -6.33 |
| ZINC000001611274 | -6.326 |
| ZINC000098023177 | -6.325 |
| ZINC000098023177 | -6.325 |
| ZINC000064033452 | -6.324 |
| ZINC000001611274 | -6.324 |
| ZINC000001530788 | -6.321 |
| ZINC000003831490 | -6.315 |
| ZINC000003831490 | -6.315 |
| ZINC000052971887 | -6.314 |
| ZINC000052971887 | -6.313 |
| ZINC000003830990 | -6.312 |
| ZINC000003927198 | -6.311 |
| ZINC000000643143 | -6.31 |
| ZINC000013585233 | -6.308 |
| ZINC000022116612 | -6.302 |
| ZINC000000007782 | -6.301 |
| ZINC000000896455 | -6.3 |
| ZINC000008214402 | -6.297 |
| ZINC000008214402 | -6.297 |
| ZINC000008214703 | -6.296 |
| ZINC000000000469 | -6.294 |
| ZINC000003830813 | -6.293 |
| ZINC000003830813 | -6.293 |
| ZINC000034676245 | -6.293 |
| ZINC000003830813 | -6.293 |
| ZINC000000039089 | -6.291 |
| ZINC000001853550 | -6.291 |
| ZINC000003798763 | -6.289 |
| ZINC000000591993 | -6.278 |
| ZINC000003830569 | -6.278 |
| ZINC000021981454 | -6.274 |
| ZINC000012404516 | -6.273 |
| ZINC000012404516 | -6.273 |
| ZINC000000897240 | -6.269 |
| ZINC000000897240 | -6.269 |
| ZINC000000897240 | -6.269 |
| ZINC000003872931 | -6.269 |
| ZINC000003872931 | -6.269 |
| ZINC000003956788 | -6.269 |
| ZINC000000001261 | -6.268 |
| ZINC000003787097 | -6.268 |
| ZINC000006827693 | -6.268 |
| ZINC000013537284 | -6.265 |
| ZINC000001530862 | -6.26 |
| ZINC000014768621 | -6.26 |
| ZINC000003831139 | -6.258 |
| ZINC000000001341 | -6.257 |
| ZINC000014768621 | -6.255 |
| ZINC000019702309 | -6.255 |
| ZINC000000968330 | -6.253 |
| ZINC000003831332 | -6.252 |
| ZINC000003798734 | -6.25 |
| ZINC000000622123 | -6.249 |
| ZINC000000020241 | -6.249 |
| ZINC000000001370 | -6.249 |
| ZINC000000001931 | -6.247 |
| ZINC000000001931 | -6.247 |
| ZINC000000968255 | -6.243 |
| ZINC000000000882 | -6.243 |
| ZINC000000074836 | -6.243 |
| ZINC000000000882 | -6.241 |
| ZINC000000001773 | -6.24 |
| ZINC000000001773 | -6.24 |
| ZINC000000001773 | -6.24 |
| ZINC000003812862 | -6.235 |
| ZINC000003981610 | -6.233 |
| ZINC000002510358 | -6.232 |
| ZINC000002510358 | -6.231 |
| ZINC000000897408 | -6.229 |
| ZINC000019632713 | -6.229 |
| ZINC000000113404 | -6.228 |
| ZINC000003812841 | -6.227 |
| ZINC000095452610 | -6.225 |
| ZINC000000003642 | -6.225 |
| ZINC000000056556 | -6.222 |
| ZINC000000085733 | -6.222 |
| ZINC000000137884 | -6.221 |
| ZINC000003830453 | -6.22 |
| ZINC000000001341 | -6.216 |
| ZINC000003871923 | -6.215 |
| ZINC000019144216 | -6.214 |
| ZINC000019144216 | -6.214 |
| ZINC000000389747 | -6.213 |
| ZINC000000389747 | -6.213 |
| ZINC000000389747 | -6.213 |
| ZINC000000057255 | -6.211 |
| ZINC000003812862 | -6.21 |
| ZINC000003812862 | -6.21 |
| ZINC000100070937 | -6.209 |
| ZINC000001530769 | -6.209 |
| ZINC000008034120 | -6.207 |
| ZINC000019419017 | -6.206 |
| ZINC000019419017 | -6.206 |
| ZINC000000006016 | -6.205 |
| ZINC000000006016 | -6.205 |
| ZINC000019419017 | -6.203 |
| ZINC000001843099 | -6.203 |
| ZINC000014879972 | -6.202 |
| ZINC000006382803 | -6.202 |
| ZINC000000097996 | -6.201 |
| ZINC000013298313 | -6.201 |
| ZINC000004658290 | -6.2 |
| ZINC000000008667 | -6.197 |
| ZINC000000896569 | -6.197 |
| ZINC000001550499 | -6.192 |
| ZINC000000000575 | -6.19 |
| ZINC000072316335 | -6.187 |
| ZINC000000113410 | -6.183 |
| ZINC000000602632 | -6.181 |
| ZINC000043200832 | -6.181 |
| ZINC000043200832 | -6.181 |
| ZINC000043200832 | -6.181 |
| ZINC000003874498 | -6.18 |
| ZINC000000607790 | -6.177 |
| ZINC000012503187 | -6.176 |
| ZINC000006827693 | -6.175 |
| ZINC000245204949 | -6.173 |
| ZINC000000968305 | -6.172 |
| ZINC000000968305 | -6.172 |
| ZINC000000033882 | -6.167 |
| ZINC000038212689 | -6.165 |
| ZINC000038212689 | -6.163 |
| ZINC000000002216 | -6.163 |
| ZINC000005133378 | -6.163 |
| ZINC000000007601 | -6.163 |
| ZINC000034781704 | -6.159 |
| ZINC000000403533 | -6.157 |
| ZINC000000403533 | -6.157 |
| ZINC000000403533 | -6.157 |
| ZINC000008015016 | -6.154 |
| ZINC000008015016 | -6.154 |
| ZINC000034051848 | -6.154 |
| ZINC000001533877 | -6.153 |
| ZINC000001533877 | -6.153 |
| ZINC000001533877 | -6.153 |
| ZINC000001533877 | -6.153 |
| ZINC000034051848 | -6.151 |
| ZINC000001533877 | -6.151 |
| ZINC000001533877 | -6.151 |
| ZINC000000402909 | -6.15 |
| ZINC000019796155 | -6.15 |
| ZINC000019796155 | -6.15 |
| ZINC000000002159 | -6.149 |
| ZINC000000136138 | -6.143 |
| ZINC000001530579 | -6.14 |
| ZINC000051133897 | -6.139 |
| ZINC000008015016 | -6.137 |
| ZINC000000621853 | -6.136 |
| ZINC000000136138 | -6.135 |
| ZINC000100017856 | -6.135 |
| ZINC000100017856 | -6.135 |
| ZINC000000056568 | -6.133 |
| ZINC000000402830 | -6.129 |
| ZINC000000402830 | -6.129 |
| ZINC000000002216 | -6.128 |
| ZINC000001482184 | -6.127 |
| ZINC000013537284 | -6.123 |
| ZINC000000896755 | -6.122 |
| ZINC000000896698 | -6.121 |
| ZINC000000896698 | -6.121 |
| ZINC000001530579 | -6.118 |
| ZINC000011617039 | -6.117 |
| ZINC000001552174 | -6.115 |
| ZINC000006382803 | -6.114 |
| ZINC000019203912 | -6.113 |
| ZINC000019203912 | -6.113 |
| ZINC000019203912 | -6.113 |
| ZINC000001530775 | -6.111 |
| ZINC000000537795 | -6.108 |
| ZINC000072316335 | -6.107 |
| ZINC000002522648 | -6.103 |
| ZINC000035328014 | -6.099 |
| ZINC000001997127 | -6.097 |
| ZINC000006827695 | -6.094 |
| ZINC000006827695 | -6.094 |
| ZINC000006827695 | -6.094 |
| ZINC000000537931 | -6.093 |
| ZINC000003872738 | -6.091 |
| ZINC000000000431 | -6.087 |
| ZINC000012503291 | -6.086 |
| ZINC000004658552 | -6.083 |
| ZINC000003875368 | -6.081 |
| ZINC000003875368 | -6.081 |
| ZINC000035328014 | -6.08 |
| ZINC000000004778 | -6.08 |
| ZINC000000896968 | -6.079 |
| ZINC000008214614 | -6.079 |
| ZINC000019144226 | -6.078 |
| ZINC000068247389 | -6.077 |
| ZINC000068247389 | -6.077 |
| ZINC000000000471 | -6.077 |
| ZINC000008466459 | -6.075 |
| ZINC000000004893 | -6.074 |
| ZINC000000004893 | -6.074 |
| ZINC000000004893 | -6.074 |
| ZINC000002015928 | -6.071 |
| ZINC000008101109 | -6.069 |
| ZINC000008101109 | -6.069 |
| ZINC000008101109 | -6.069 |
| ZINC000012503187 | -6.069 |
| ZINC000000020244 | -6.069 |
| ZINC000000020244 | -6.069 |
| ZINC000001530912 | -6.067 |
| ZINC000001530912 | -6.067 |
| ZINC000001530912 | -6.067 |
| ZINC000000006427 | -6.066 |
| ZINC000000002212 | -6.065 |
| ZINC000000896634 | -6.065 |
| ZINC000001530939 | -6.063 |
| ZINC000005133329 | -6.062 |
| ZINC000003801919 | -6.062 |
| ZINC000100009278 | -6.059 |
| ZINC000100009278 | -6.059 |
| ZINC000003801919 | -6.056 |
| ZINC000003801919 | -6.056 |
| ZINC000000056653 | -6.056 |
| ZINC000000968233 | -6.055 |
| ZINC000003813003 | -6.054 |
| ZINC000022002214 | -6.054 |
| ZINC000022002214 | -6.054 |
| ZINC000000896717 | -6.054 |
| ZINC000004693575 | -6.052 |
| ZINC000000001382 | -6.049 |
| ZINC000095616937 | -6.048 |
| ZINC000003812913 | -6.047 |
| ZINC000001542002 | -6.041 |
| ZINC000003794711 | -6.04 |
| ZINC000003794711 | -6.04 |
| ZINC000003794711 | -6.04 |
| ZINC000001530761 | -6.038 |
| ZINC000000113355 | -6.038 |
| ZINC000001530863 | -6.037 |
| ZINC000003917708 | -6.037 |
| ZINC000000388081 | -6.031 |
| ZINC000000388081 | -6.031 |
| ZINC000000388081 | -6.031 |
| ZINC000013298313 | -6.029 |
| ZINC000000001132 | -6.026 |
| ZINC000100004345 | -6.024 |
| ZINC000000000905 | -6.024 |
| ZINC000000000903 | -6.021 |
| ZINC000009224016 | -6.021 |
| ZINC000009224016 | -6.021 |
| ZINC000094566093 | -6.02 |
| ZINC000000527386 | -6.019 |
| ZINC000000155269 | -6.017 |
| ZINC000000155269 | -6.017 |
| ZINC000000057532 | -6.017 |
| ZINC000034806477 | -6.016 |
| ZINC000001542113 | -6.013 |
| ZINC000001542113 | -6.013 |
| ZINC000000002055 | -6.011 |
| ZINC000000002055 | -6.011 |
| ZINC000053022902 | -6.006 |
| ZINC000053022902 | -6.006 |
| ZINC000000000323 | -6.005 |
| ZINC000000508068 | -6.001 |
| ZINC000022010375 | -6.001 |
| ZINC000022010375 | -6.001 |
| ZINC000022010375 | -6.001 |
| ZINC000000596731 | -5.993 |
| ZINC000000596731 | -5.993 |
| ZINC000000000122 | -5.991 |
| ZINC000000000122 | -5.991 |
| ZINC000001639567 | -5.99 |
| ZINC000100006770 | -5.989 |
| ZINC000000403566 | -5.988 |
| ZINC000000897291 | -5.983 |
| ZINC000000000882 | -5.983 |
| ZINC000000000882 | -5.983 |
| ZINC000003809490 | -5.981 |
| ZINC000001554588 | -5.979 |
| ZINC000000057001 | -5.978 |
| ZINC000000004949 | -5.978 |
| ZINC000013520815 | -5.977 |
| ZINC000006858022 | -5.977 |
| ZINC000003813042 | -5.969 |
| ZINC000014961096 | -5.968 |
| ZINC000019144231 | -5.966 |
| ZINC000001530977 | -5.965 |
| ZINC000001530977 | -5.965 |
| ZINC000001530977 | -5.965 |
| ZINC000000896703 | -5.965 |
| ZINC000000000905 | -5.964 |
| ZINC000003875332 | -5.961 |
| ZINC000100055899 | -5.956 |
| ZINC000003873295 | -5.955 |
| ZINC000001532728 | -5.954 |
| ZINC000000001084 | -5.953 |
| ZINC000000599734 | -5.952 |
| ZINC000001530621 | -5.947 |
| ZINC000000020228 | -5.944 |
| ZINC000017146904 | -5.944 |
| ZINC000000020228 | -5.944 |
| ZINC000000120319 | -5.943 |
| ZINC000000120319 | -5.943 |
| ZINC000001530621 | -5.937 |
| ZINC000001481910 | -5.936 |
| ZINC000003914810 | -5.936 |
| ZINC000001542930 | -5.934 |
| ZINC000000114127 | -5.93 |
| ZINC000019632917 | -5.929 |
| ZINC000003814422 | -5.928 |
| ZINC000043195697 | -5.927 |
| ZINC000043195697 | -5.927 |
| ZINC000000001267 | -5.927 |
| ZINC000003830842 | -5.925 |
| ZINC000000001464 | -5.923 |
| ZINC000017146904 | -5.92 |
| ZINC000019632718 | -5.919 |
| ZINC000035653007 | -5.918 |
| ZINC000004215736 | -5.915 |
| ZINC000004215736 | -5.915 |
| ZINC000012503187 | -5.913 |
| ZINC000000607939 | -5.913 |
| ZINC000001895505 | -5.909 |
| ZINC000012503187 | -5.907 |
| ZINC000002847375 | -5.904 |
| ZINC000002847375 | -5.904 |
| ZINC000000000596 | -5.903 |
| ZINC000000000596 | -5.903 |
| ZINC000003079340 | -5.902 |
| ZINC000000089763 | -5.898 |
| ZINC000013831141 | -5.896 |
| ZINC000096006009 | -5.892 |
| ZINC000096006009 | -5.891 |
| ZINC000100036536 | -5.89 |
| ZINC000100036536 | -5.89 |
| ZINC000095619101 | -5.886 |
| ZINC000095619101 | -5.886 |
| ZINC000043195697 | -5.885 |
| ZINC000043195697 | -5.885 |
| ZINC000043195697 | -5.885 |
| ZINC000000584092 | -5.883 |
| ZINC000003873936 | -5.882 |
| ZINC000003873936 | -5.882 |
| ZINC000049933061 | -5.879 |
| ZINC000095626706 | -5.877 |
| ZINC000000896918 | -5.876 |
| ZINC000001542392 | -5.872 |
| ZINC000003792789 | -5.87 |
| ZINC000003932831 | -5.87 |
| ZINC000004097225 | -5.87 |
| ZINC000019361042 | -5.868 |
| ZINC000019361042 | -5.868 |
| ZINC000001530816 | -5.868 |
| ZINC000001530816 | -5.866 |
| ZINC000000000941 | -5.866 |
| ZINC000019362735 | -5.864 |
| ZINC000019362735 | -5.864 |
| ZINC000019362735 | -5.864 |
| ZINC000003875560 | -5.862 |
| ZINC000003875259 | -5.858 |
| ZINC000003875259 | -5.858 |
| ZINC000003806413 | -5.857 |
| ZINC000000057464 | -5.855 |
| ZINC000003806413 | -5.854 |
| ZINC000003787060 | -5.854 |
| ZINC000000000061 | -5.853 |
| ZINC000000000853 | -5.852 |
| ZINC000003873921 | -5.85 |
| ZINC000000537805 | -5.849 |
| ZINC000016052277 | -5.848 |
| ZINC000003875484 | -5.846 |
| ZINC000000403079 | -5.844 |
| ZINC000100014475 | -5.844 |
| ZINC000001850376 | -5.841 |
| ZINC000000089763 | -5.84 |
| ZINC000095616600 | -5.84 |
| ZINC000001849548 | -5.839 |
| ZINC000000643138 | -5.837 |
| ZINC000004215234 | -5.837 |
| ZINC000000537805 | -5.836 |
| ZINC000000537795 | -5.833 |
| ZINC000003964325 | -5.831 |
| ZINC000000057533 | -5.83 |
| ZINC000000403079 | -5.826 |
| ZINC000000897244 | -5.824 |
| ZINC000118912517 | -5.824 |
| ZINC000000105216 | -5.822 |
| ZINC000004641374 | -5.816 |
| ZINC000004428529 | -5.816 |
| ZINC000000968327 | -5.815 |
| ZINC000000968327 | -5.815 |
| ZINC000018203737 | -5.814 |
| ZINC000018203737 | -5.814 |
| ZINC000001996117 | -5.812 |
| ZINC000001996117 | -5.812 |
| ZINC000019156872 | -5.808 |
| ZINC000019156872 | -5.808 |
| ZINC000019156872 | -5.808 |
| ZINC000000266964 | -5.805 |
| ZINC000004693574 | -5.803 |
| ZINC000095616601 | -5.802 |
| ZINC000000537805 | -5.801 |
| ZINC000000000490 | -5.8 |
| ZINC000019594599 | -5.798 |
| ZINC000019594599 | -5.798 |
| ZINC000019594599 | -5.798 |
| ZINC000013550868 | -5.797 |
| ZINC000003824921 | -5.795 |
| ZINC000001571009 | -5.794 |
| ZINC000000000449 | -5.794 |
| ZINC000058581064 | -5.793 |
| ZINC000000897085 | -5.792 |
| ZINC000035024346 | -5.792 |
| ZINC000148723177 | -5.79 |
| ZINC000148723177 | -5.79 |
| ZINC000043207851 | -5.788 |
| ZINC000003830500 | -5.787 |
| ZINC000003872994 | -5.783 |
| ZINC000004468778 | -5.782 |
| ZINC000000001706 | -5.781 |
| ZINC000000049154 | -5.78 |
| ZINC000003830500 | -5.777 |
| ZINC000003589203 | -5.77 |
| ZINC000000592419 | -5.769 |
| ZINC000001482197 | -5.769 |
| ZINC000003830339 | -5.768 |
| ZINC000001530625 | -5.768 |
| ZINC000000896663 | -5.766 |
| ZINC000000005895 | -5.763 |
| ZINC000000020231 | -5.76 |
| ZINC000000000655 | -5.759 |
| ZINC000000537964 | -5.759 |
| ZINC000003875483 | -5.757 |
| ZINC000003875483 | -5.757 |
| ZINC000003875483 | -5.757 |
| ZINC000012503151 | -5.756 |
| ZINC000253917094 | -5.756 |
| ZINC000094566092 | -5.755 |
| ZINC000060325170 | -5.752 |
| ZINC000001530618 | -5.744 |
| ZINC000000121480 | -5.742 |
| ZINC000004618208 | -5.72 |
| ZINC000003830986 | -5.719 |
| ZINC000000395010 | -5.717 |
| ZINC000070466416 | -5.715 |
| ZINC000000075126 | -5.71 |
| ZINC000001530803 | -5.709 |
| ZINC000100017856 | -5.709 |
| ZINC000100017856 | -5.709 |
| ZINC000006627681 | -5.707 |
| ZINC000003807804 | -5.706 |
| ZINC000003990451 | -5.702 |
| ZINC000003872277 | -5.702 |
| ZINC000006021043 | -5.702 |
| ZINC000003875393 | -5.701 |
| ZINC000003875393 | -5.701 |
| ZINC000003875393 | -5.701 |
| ZINC000118912393 | -5.701 |
| ZINC000000015515 | -5.7 |
| ZINC000001883067 | -5.696 |
| ZINC000003830314 | -5.693 |
| ZINC000004258316 | -5.693 |
| ZINC000043450324 | -5.693 |
| ZINC000043450324 | -5.693 |
| ZINC000001530283 | -5.69 |
| ZINC000095616603 | -5.686 |
| ZINC000003812989 | -5.682 |
| ZINC000003812989 | -5.682 |
| ZINC000003920266 | -5.678 |
| ZINC000000001681 | -5.677 |
| ZINC000000001681 | -5.677 |
| ZINC000003813047 | -5.676 |
| ZINC000018516586 | -5.676 |
| ZINC000011726211 | -5.675 |
| ZINC000011726211 | -5.675 |
| ZINC000000388081 | -5.673 |
| ZINC000000388081 | -5.673 |
| ZINC000000388081 | -5.673 |
| ZINC000018043251 | -5.671 |
| ZINC000022010379 | -5.668 |
| ZINC000022010379 | -5.668 |
| ZINC000022010379 | -5.668 |
| ZINC000003920266 | -5.667 |
| ZINC000001530617 | -5.666 |
| ZINC000001532526 | -5.663 |
| ZINC000021303210 | -5.658 |
| ZINC000000000607 | -5.654 |
| ZINC000096942202 | -5.654 |
| ZINC000004097304 | -5.653 |
| ZINC000000044027 | -5.653 |
| ZINC000001530694 | -5.65 |
| ZINC000018099446 | -5.648 |
| ZINC000100019007 | -5.648 |
| ZINC000003960338 | -5.643 |
| ZINC000004099200 | -5.642 |
| ZINC000000016154 | -5.641 |
| ZINC000004658603 | -5.639 |
| ZINC000004658603 | -5.639 |
| ZINC000000001655 | -5.635 |
| ZINC000001530689 | -5.63 |
| ZINC000001530689 | -5.63 |
| ZINC000001532517 | -5.63 |
| ZINC000001530725 | -5.63 |
| ZINC000000014037 | -5.627 |
| ZINC000008034121 | -5.625 |
| ZINC000003782807 | -5.618 |
| ZINC000000014360 | -5.615 |
| ZINC000253476027 | -5.612 |
| ZINC000100015775 | -5.612 |
| ZINC000000968273 | -5.608 |
| ZINC000000020253 | -5.604 |
| ZINC000001530652 | -5.6 |
| ZINC000001530652 | -5.6 |
| ZINC000003872055 | -5.6 |
| ZINC000001530600 | -5.599 |
| ZINC000001530600 | -5.599 |
| ZINC000000000053 | -5.596 |
| ZINC000003872566 | -5.595 |
| ZINC000000057206 | -5.595 |
| ZINC000003805768 | -5.595 |
| ZINC000003805768 | -5.595 |
| ZINC000001530600 | -5.594 |
| ZINC000000057206 | -5.591 |
| ZINC000000001982 | -5.591 |
| ZINC000001539579 | -5.59 |
| ZINC000001530611 | -5.59 |
| ZINC000003816287 | -5.59 |
| ZINC000011616882 | -5.59 |
| ZINC000000001795 | -5.588 |
| ZINC000003812888 | -5.583 |
| ZINC000003812888 | -5.583 |
| ZINC000004097467 | -5.582 |
| ZINC000004258316 | -5.582 |
| ZINC000000968264 | -5.578 |
| ZINC000000968264 | -5.578 |
| ZINC000000000215 | -5.574 |
| ZINC000000000215 | -5.574 |
| ZINC000022002218 | -5.573 |
| ZINC000022002218 | -5.573 |
| ZINC000001530575 | -5.572 |
| ZINC000000537791 | -5.572 |
| ZINC000001851149 | -5.57 |
| ZINC000001851149 | -5.57 |
| ZINC000100009383 | -5.569 |
| ZINC000005179119 | -5.566 |
| ZINC000000537791 | -5.565 |
| ZINC000006745272 | -5.564 |
| ZINC000000006157 | -5.562 |
| ZINC000253498282 | -5.56 |
| ZINC000019796018 | -5.559 |
| ZINC000019796018 | -5.559 |
| ZINC000019796018 | -5.559 |
| ZINC000003830391 | -5.558 |
| ZINC000003830391 | -5.558 |
| ZINC000001530601 | -5.557 |
| ZINC000095616601 | -5.557 |
| ZINC000003818808 | -5.555 |
| ZINC000000000740 | -5.555 |
| ZINC000000001331 | -5.552 |
| ZINC000003079342 | -5.548 |
| ZINC000001530568 | -5.546 |
| ZINC000003978006 | -5.546 |
| ZINC000004102194 | -5.543 |
| ZINC000095616600 | -5.543 |
| ZINC000001843099 | -5.539 |
| ZINC000003629271 | -5.537 |
| ZINC000000057513 | -5.533 |
| ZINC000000057513 | -5.533 |
| ZINC000001482094 | -5.53 |
| ZINC000002548959 | -5.528 |
| ZINC000118912450 | -5.527 |
| ZINC000000001011 | -5.516 |
| ZINC000001548097 | -5.513 |
| ZINC000001548097 | -5.513 |
| ZINC000100016084 | -5.513 |
| ZINC000000156792 | -5.507 |
| ZINC000100001918 | -5.507 |
| ZINC000000113442 | -5.504 |
| ZINC000003979899 | -5.504 |
| ZINC000002008310 | -5.502 |
| ZINC000100061056 | -5.501 |
| ZINC000012503068 | -5.501 |
| ZINC000000000973 | -5.498 |
| ZINC000001280665 | -5.497 |
| ZINC000001280665 | -5.497 |
| ZINC000001280665 | -5.497 |
| ZINC000000000973 | -5.495 |
| ZINC000001999487 | -5.494 |
| ZINC000000968326 | -5.493 |
| ZINC000066166864 | -5.491 |
| ZINC000066166864 | -5.491 |
| ZINC000000968326 | -5.488 |
| ZINC000085205451 | -5.485 |
| ZINC000095564694 | -5.484 |
| ZINC000095564694 | -5.484 |
| ZINC000095564694 | -5.484 |
| ZINC000000895081 | -5.482 |
| ZINC000116473771 | -5.482 |
| ZINC000003872520 | -5.482 |
| ZINC000001850377 | -5.481 |
| ZINC000116473771 | -5.48 |
| ZINC000000114124 | -5.479 |
| ZINC000068153186 | -5.475 |
| ZINC000001846431 | -5.472 |
| ZINC000001530938 | -5.472 |
| ZINC000001530938 | -5.472 |
| ZINC000003938704 | -5.468 |
| ZINC000068153186 | -5.468 |
| ZINC000004468780 | -5.467 |
| ZINC000001530652 | -5.467 |
| ZINC000001530652 | -5.467 |
| ZINC000003806721 | -5.46 |
| ZINC000003806721 | -5.46 |
| ZINC000000001281 | -5.46 |
| ZINC000100005670 | -5.46 |
| ZINC000000005152 | -5.455 |
| ZINC000003818808 | -5.454 |
| ZINC000000968330 | -5.451 |
| ZINC000013540519 | -5.451 |
| ZINC000001530741 | -5.447 |
| ZINC000410428674 | -5.446 |
| ZINC000003807917 | -5.445 |
| ZINC000001493878 | -5.441 |
| ZINC000000056647 | -5.441 |
| ZINC000002525885 | -5.439 |
| ZINC000002525885 | -5.439 |
| ZINC000000006251 | -5.438 |
| ZINC000000006251 | -5.438 |
| ZINC000001554010 | -5.438 |
| ZINC000001698306 | -5.437 |
| ZINC000003945984 | -5.436 |
| ZINC000030691763 | -5.436 |
| ZINC000000020221 | -5.435 |
| ZINC000000538621 | -5.433 |
| ZINC000000538621 | -5.433 |
| ZINC000019594557 | -5.432 |
| ZINC000019594557 | -5.432 |
| ZINC000008214514 | -5.43 |
| ZINC000004392649 | -5.42 |
| ZINC000000155905 | -5.414 |
| ZINC000000020259 | -5.41 |
| ZINC000003831551 | -5.408 |
| ZINC000000000083 | -5.405 |
| ZINC000116473771 | -5.404 |
| ZINC000000009689 | -5.401 |
| ZINC000000537791 | -5.401 |
| ZINC000000057313 | -5.399 |
| ZINC000000057341 | -5.398 |
| ZINC000003784182 | -5.398 |
| ZINC000001530728 | -5.396 |
| ZINC000001530728 | -5.396 |
| ZINC000000000711 | -5.396 |
| ZINC000000057253 | -5.393 |
| ZINC000000057253 | -5.393 |
| ZINC000000002009 | -5.391 |
| ZINC000000000751 | -5.388 |
| ZINC000000000751 | -5.388 |
| ZINC000000000751 | -5.388 |
| ZINC000000001148 | -5.387 |
| ZINC000003876023 | -5.382 |
| ZINC000001530764 | -5.38 |
| ZINC000019632917 | -5.377 |
| ZINC000008101126 | -5.375 |
| ZINC000000895103 | -5.373 |
| ZINC000011680943 | -5.373 |
| ZINC000000057435 | -5.372 |
| ZINC000100014909 | -5.37 |
| ZINC000100014909 | -5.37 |
| ZINC000000083315 | -5.363 |
| ZINC000003831429 | -5.363 |
| ZINC000003876136 | -5.362 |
| ZINC000011680943 | -5.362 |
| ZINC000000057512 | -5.356 |
| ZINC000100071256 | -5.356 |
| ZINC000004217732 | -5.352 |
| ZINC000000897385 | -5.347 |
| ZINC000003830961 | -5.345 |
| ZINC000000119344 | -5.344 |
| ZINC000012503156 | -5.343 |
| ZINC000001482164 | -5.336 |
| ZINC000008552123 | -5.334 |
| ZINC000008552123 | -5.334 |
| ZINC000008552123 | -5.334 |
| ZINC000000968328 | -5.334 |
| ZINC000100009280 | -5.331 |
| ZINC000100009280 | -5.331 |
| ZINC000000001728 | -5.33 |
| ZINC000012360535 | -5.324 |
| ZINC000035999642 | -5.323 |
| ZINC000001530654 | -5.322 |
| ZINC000001530654 | -5.322 |
| ZINC000035999642 | -5.322 |
| ZINC000001530817 | -5.321 |
| ZINC000000002041 | -5.321 |
| ZINC000000000353 | -5.319 |
| ZINC000035902489 | -5.316 |
| ZINC000004632106 | -5.316 |
| ZINC000004632106 | -5.316 |
| ZINC000007997568 | -5.315 |
| ZINC000007997568 | -5.315 |
| ZINC000000057512 | -5.311 |
| ZINC000000105196 | -5.311 |
| ZINC000000001554 | -5.304 |
| ZINC000001530817 | -5.301 |
| ZINC000000000973 | -5.298 |
| ZINC000003875392 | -5.296 |
| ZINC000003875392 | -5.296 |
| ZINC000003875392 | -5.296 |
| ZINC000001690604 | -5.293 |
| ZINC000003831040 | -5.293 |
| ZINC000043450326 | -5.288 |
| ZINC000043450326 | -5.288 |
| ZINC000008214651 | -5.287 |
| ZINC000052509463 | -5.287 |
| ZINC000052509463 | -5.285 |
| ZINC000000895042 | -5.284 |
| ZINC000100037020 | -5.279 |
| ZINC000100037020 | -5.279 |
| ZINC000100037020 | -5.279 |
| ZINC000003815424 | -5.278 |
| ZINC000000004840 | -5.278 |
| ZINC000004693574 | -5.274 |
| ZINC000000643153 | -5.273 |
| ZINC000000057340 | -5.271 |
| ZINC000002020233 | -5.269 |
| ZINC000003831050 | -5.257 |
| ZINC000013973998 | -5.255 |
| ZINC000101489663 | -5.254 |
| ZINC000008214629 | -5.253 |
| ZINC000003875334 | -5.253 |
| ZINC000000896711 | -5.25 |
| ZINC000000968310 | -5.25 |
| ZINC000000968310 | -5.25 |
| ZINC000000968310 | -5.25 |
| ZINC000003875259 | -5.244 |
| ZINC000000895034 | -5.243 |
| ZINC000000895034 | -5.243 |
| ZINC000003938695 | -5.241 |
| ZINC000040899447 | -5.239 |
| ZINC000000968328 | -5.236 |
| ZINC000000010164 | -5.233 |
| ZINC000000010164 | -5.233 |
| ZINC000000895032 | -5.231 |
| ZINC000003875259 | -5.228 |
| ZINC000084843283 | -5.221 |
| ZINC000004097476 | -5.221 |
| ZINC000008035268 | -5.218 |
| ZINC000002561203 | -5.217 |
| ZINC000095616599 | -5.209 |
| ZINC000011677376 | -5.209 |
| ZINC000011677376 | -5.209 |
| ZINC000011677376 | -5.209 |
| ZINC000000597013 | -5.201 |
| ZINC000000597013 | -5.201 |
| ZINC000012402836 | -5.2 |
| ZINC000000000506 | -5.2 |
| ZINC000095616599 | -5.199 |
| ZINC000003831051 | -5.196 |
| ZINC000000001145 | -5.192 |
| ZINC000000001145 | -5.191 |
| ZINC000011680067 | -5.19 |
| ZINC000000002101 | -5.19 |
| ZINC000003782599 | -5.188 |
| ZINC000001530688 | -5.188 |
| ZINC000087515509 | -5.186 |
| ZINC000003781664 | -5.183 |
| ZINC000004658290 | -5.181 |
| ZINC000001532522 | -5.178 |
| ZINC000004577910 | -5.176 |
| ZINC000100032379 | -5.176 |
| ZINC000000388462 | -5.171 |
| ZINC000000001411 | -5.169 |
| ZINC000001530716 | -5.167 |
| ZINC000001530751 | -5.166 |
| ZINC000001530751 | -5.166 |
| ZINC000019632706 | -5.165 |
| ZINC000003871541 | -5.164 |
| ZINC000003812983 | -5.163 |
| ZINC000003812983 | -5.163 |
| ZINC000000002005 | -5.156 |
| ZINC000000020783 | -5.148 |
| ZINC000022010387 | -5.143 |
| ZINC000022010387 | -5.143 |
| ZINC000022010387 | -5.143 |
| ZINC000000001145 | -5.142 |
| ZINC000000897322 | -5.139 |
| ZINC000001482113 | -5.139 |
| ZINC000000001145 | -5.136 |
| ZINC000000004351 | -5.126 |
| ZINC000000004351 | -5.126 |
| ZINC000000000973 | -5.125 |
| ZINC000000000242 | -5.124 |
| ZINC000000000242 | -5.124 |
| ZINC000005764759 | -5.123 |
| ZINC000000020245 | -5.119 |
| ZINC000096272772 | -5.118 |
| ZINC000021297660 | -5.118 |
| ZINC000021297660 | -5.118 |
| ZINC000033943508 | -5.11 |
| ZINC000100032379 | -5.107 |
| ZINC000000057522 | -5.106 |
| ZINC000000394284 | -5.106 |
| ZINC000019418959 | -5.105 |
| ZINC000019418959 | -5.105 |
| ZINC000019418959 | -5.105 |
| ZINC000022443609 | -5.104 |
| ZINC000000057512 | -5.103 |
| ZINC000003799072 | -5.102 |
| ZINC000030691760 | -5.102 |
| ZINC000000895099 | -5.1 |
| ZINC000000000509 | -5.099 |
| ZINC000000000509 | -5.099 |
| ZINC000000000509 | -5.099 |
| ZINC000003876068 | -5.098 |
| ZINC000003876068 | -5.098 |
| ZINC000000002647 | -5.097 |
| ZINC000019144226 | -5.096 |
| ZINC000000113398 | -5.095 |
| ZINC000036294079 | -5.09 |
| ZINC000036294079 | -5.09 |
| ZINC000003973334 | -5.089 |
| ZINC000019144231 | -5.089 |
| ZINC000000004785 | -5.087 |
| ZINC000000057254 | -5.084 |
| ZINC000000057254 | -5.084 |
| ZINC000022065398 | -5.082 |
| ZINC000004474414 | -5.079 |
| ZINC000004393164 | -5.075 |
| ZINC000000968275 | -5.073 |
| ZINC000003843198 | -5.071 |
| ZINC000043194409 | -5.056 |
| ZINC000003926298 | -5.053 |
| ZINC000001489478 | -5.05 |
| ZINC000000538658 | -5.05 |
| ZINC000003079336 | -5.048 |
| ZINC000095619100 | -5.044 |
| ZINC000095619100 | -5.044 |
| ZINC000038140873 | -5.043 |
| ZINC000000020231 | -5.034 |
| ZINC000001489478 | -5.03 |
| ZINC000000601317 | -5.03 |
| ZINC000012360535 | -5.025 |
| ZINC000008214692 | -5.023 |
| ZINC000003812974 | -5.023 |
| ZINC000001530737 | -5.021 |
| ZINC000001530737 | -5.021 |
| ZINC000001530736 | -5.021 |
| ZINC000001530736 | -5.021 |
| ZINC000013545634 | -5.021 |
| ZINC000013545634 | -5.02 |
| ZINC000052957434 | -5.019 |
| ZINC000052957434 | -5.019 |
| ZINC000001530569 | -5.018 |
| ZINC000022010382 | -5.015 |
| ZINC000022010382 | -5.015 |
| ZINC000022010382 | -5.015 |
| ZINC000001530710 | -5.015 |
| ZINC000000601281 | -5.009 |
| ZINC000000601281 | -5.009 |
| ZINC000019632633 | -5.008 |
| ZINC000019632633 | -5.008 |
| ZINC000019632633 | -5.008 |
| ZINC000003812897 | -5.008 |
| ZINC000003782550 | -5.001 |
| ZINC000000156395 | -4.997 |
| ZINC000003995809 | -4.996 |
| ZINC000003782550 | -4.996 |
| ZINC000003927200 | -4.994 |
| ZINC000000018087 | -4.991 |
| ZINC000006094354 | -4.991 |
| ZINC000006094354 | -4.991 |
| ZINC000008220878 | -4.991 |
| ZINC000008220878 | -4.991 |
| ZINC000001542392 | -4.991 |
| ZINC000000968263 | -4.986 |
| ZINC000003972949 | -4.984 |
| ZINC000003972949 | -4.984 |
| ZINC000100007011 | -4.978 |
| ZINC000000391812 | -4.975 |
| ZINC000000391812 | -4.975 |
| ZINC000000391812 | -4.975 |
| ZINC000000075008 | -4.965 |
| ZINC000000075008 | -4.965 |
| ZINC000000000973 | -4.953 |
| ZINC000000896709 | -4.948 |
| ZINC000000403609 | -4.946 |
| ZINC000019632912 | -4.945 |
| ZINC000001530756 | -4.944 |
| ZINC000003812867 | -4.942 |
| ZINC000000009073 | -4.942 |
| ZINC000000009073 | -4.942 |
| ZINC000000009073 | -4.942 |
| ZINC000000000973 | -4.937 |
| ZINC000072318121 | -4.934 |
| ZINC000072318121 | -4.934 |
| ZINC000000968257 | -4.932 |
| ZINC000003651680 | -4.932 |
| ZINC000003651680 | -4.93 |
| ZINC000003831586 | -4.929 |
| ZINC000000113426 | -4.926 |
| ZINC000003795819 | -4.91 |
| ZINC000003795819 | -4.91 |
| ZINC000009212654 | -4.91 |
| ZINC000003876069 | -4.909 |
| ZINC000000000196 | -4.909 |
| ZINC000000000196 | -4.909 |
| ZINC000000004448 | -4.907 |
| ZINC000000057512 | -4.906 |
| ZINC000003876069 | -4.905 |
| ZINC000000000271 | -4.902 |
| ZINC000000154964 | -4.887 |
| ZINC000000154964 | -4.887 |
| ZINC000000154964 | -4.887 |
| ZINC000242437514 | -4.876 |
| ZINC000003881958 | -4.871 |
| ZINC000003806063 | -4.869 |
| ZINC000000005878 | -4.865 |
| ZINC000000491073 | -4.854 |
| ZINC000000491073 | -4.854 |
| ZINC000000491073 | -4.854 |
| ZINC000001531008 | -4.853 |
| ZINC000001481833 | -4.844 |
| ZINC000000389149 | -4.836 |
| ZINC000000538386 | -4.831 |
| ZINC000000538386 | -4.831 |
| ZINC000084441937 | -4.829 |
| ZINC000084441937 | -4.829 |
| ZINC000096014710 | -4.828 |
| ZINC000000056427 | -4.827 |
| ZINC000000000456 | -4.822 |
| ZINC000000000456 | -4.822 |
| ZINC000000000456 | -4.822 |
| ZINC000003800008 | -4.816 |
| ZINC000000001979 | -4.814 |
| ZINC000003831404 | -4.808 |
| ZINC000003831404 | -4.808 |
| ZINC000001530805 | -4.805 |
| ZINC000000000347 | -4.801 |
| ZINC000000000347 | -4.801 |
| ZINC000000000347 | -4.801 |
| ZINC000100018854 | -4.801 |
| ZINC000100018854 | -4.801 |
| ZINC000100018854 | -4.801 |
| ZINC000001187543 | -4.801 |
| ZINC000003812944 | -4.798 |
| ZINC000000056645 | -4.795 |
| ZINC000022056030 | -4.795 |
| ZINC000022056030 | -4.795 |
| ZINC000022056030 | -4.795 |
| ZINC000003830347 | -4.793 |
| ZINC000003830347 | -4.793 |
| ZINC000014263142 | -4.782 |
| ZINC000019702309 | -4.78 |
| ZINC000019166988 | -4.776 |
| ZINC000003977777 | -4.771 |
| ZINC000000897288 | -4.768 |
| ZINC000001542199 | -4.767 |
| ZINC000019796087 | -4.764 |
| ZINC000019796087 | -4.764 |
| ZINC000019796087 | -4.764 |
| ZINC000003831165 | -4.75 |
| ZINC000000057062 | -4.749 |
| ZINC000000968345 | -4.74 |
| ZINC000095619105 | -4.737 |
| ZINC000095619105 | -4.737 |
| ZINC000095619105 | -4.737 |
| ZINC000001530929 | -4.736 |
| ZINC000001530929 | -4.735 |
| ZINC000027990463 | -4.735 |
| ZINC000000901552 | -4.733 |
| ZINC000003645145 | -4.73 |
| ZINC000003938686 | -4.729 |
| ZINC000052509463 | -4.727 |
| ZINC000000897251 | -4.723 |
| ZINC000000897251 | -4.723 |
| ZINC000001530814 | -4.722 |
| ZINC000001530814 | -4.722 |
| ZINC000001530814 | -4.722 |
| ZINC000003826253 | -4.715 |
| ZINC000014164617 | -4.715 |
| ZINC000003938686 | -4.713 |
| ZINC000003881640 | -4.711 |
| ZINC000000049154 | -4.707 |
| ZINC000001530806 | -4.707 |
| ZINC000003831430 | -4.689 |
| ZINC000001530752 | -4.687 |
| ZINC000001530752 | -4.687 |
| ZINC000000599985 | -4.678 |
| ZINC000001530567 | -4.675 |
| ZINC000002016257 | -4.673 |
| ZINC000004629876 | -4.673 |
| ZINC000003812933 | -4.668 |
| ZINC000100299039 | -4.665 |
| ZINC000001530703 | -4.663 |
| ZINC000004640636 | -4.661 |
| ZINC000004640636 | -4.657 |
| ZINC000003807172 | -4.657 |
| ZINC000004097416 | -4.656 |
| ZINC000000538509 | -4.653 |
| ZINC000000538509 | -4.653 |
| ZINC000001843047 | -4.647 |
| ZINC000008214614 | -4.629 |
| ZINC000003816292 | -4.623 |
| ZINC000001530570 | -4.621 |
| ZINC000030690433 | -4.618 |
| ZINC000030690433 | -4.618 |
| ZINC000030690433 | -4.618 |
| ZINC000000599985 | -4.613 |
| ZINC000019796158 | -4.61 |
| ZINC000019796158 | -4.61 |
| ZINC000000006481 | -4.606 |
| ZINC000003874950 | -4.604 |
| ZINC000000402954 | -4.604 |
| ZINC000000402954 | -4.604 |
| ZINC000000402954 | -4.604 |
| ZINC000018324776 | -4.603 |
| ZINC000018324776 | -4.603 |
| ZINC000001530572 | -4.601 |
| ZINC000003874950 | -4.6 |
| ZINC000000968375 | -4.598 |
| ZINC000003581355 | -4.594 |
| ZINC000005752191 | -4.593 |
| ZINC000000113446 | -4.591 |
| ZINC000001532529 | -4.588 |
| ZINC000053683151 | -4.586 |
| ZINC000053683151 | -4.586 |
| ZINC000053683151 | -4.586 |
| ZINC000013545636 | -4.584 |
| ZINC000001530635 | -4.58 |
| ZINC000018324776 | -4.58 |
| ZINC000003830321 | -4.578 |
| ZINC000004658562 | -4.574 |
| ZINC000003995811 | -4.573 |
| ZINC000003985982 | -4.564 |
| ZINC000000537805 | -4.563 |
| ZINC000001850374 | -4.556 |
| ZINC000053683345 | -4.556 |
| ZINC000000896695 | -4.551 |
| ZINC000000896695 | -4.551 |
| ZINC000001530703 | -4.543 |
| ZINC000003931840 | -4.542 |
| ZINC000003931840 | -4.542 |
| ZINC000002032615 | -4.539 |
| ZINC000001530703 | -4.537 |
| ZINC000013545636 | -4.534 |
| ZINC000000001798 | -4.525 |
| ZINC000000001798 | -4.525 |
| ZINC000000001798 | -4.525 |
| ZINC000000006226 | -4.524 |
| ZINC000000056646 | -4.506 |
| ZINC000003833821 | -4.497 |
| ZINC000000002299 | -4.48 |
| ZINC000000002299 | -4.48 |
| ZINC000000002299 | -4.48 |
| ZINC000000537795 | -4.473 |
| ZINC000000901061 | -4.466 |
| ZINC000253476025 | -4.462 |
| ZINC000100015780 | -4.462 |
| ZINC000000014257 | -4.461 |
| ZINC000005224188 | -4.457 |
| ZINC000000896484 | -4.457 |
| ZINC000000896484 | -4.457 |
| ZINC000003995807 | -4.454 |
| ZINC000001576892 | -4.454 |
| ZINC000000001792 | -4.451 |
| ZINC000004212851 | -4.45 |
| ZINC000002005550 | -4.447 |
| ZINC000002005550 | -4.446 |
| ZINC000003921872 | -4.436 |
| ZINC000019364225 | -4.431 |
| ZINC000004676424 | -4.431 |
| ZINC000003930376 | -4.428 |
| ZINC000019364225 | -4.426 |
| ZINC000000113415 | -4.421 |
| ZINC000003831138 | -4.418 |
| ZINC000003977978 | -4.408 |
| ZINC000003831417 | -4.391 |
| ZINC000003831405 | -4.386 |
| ZINC000003831405 | -4.386 |
| ZINC000000020257 | -4.385 |
| ZINC000000020237 | -4.38 |
| ZINC000000020237 | -4.38 |
| ZINC000003871832 | -4.359 |
| ZINC000003871832 | -4.359 |
| ZINC000019875504 | -4.354 |
| ZINC000100015048 | -4.352 |
| ZINC000003812988 | -4.351 |
| ZINC000003812988 | -4.351 |
| ZINC000001530703 | -4.346 |
| ZINC000012358719 | -4.335 |
| ZINC000000020250 | -4.334 |
| ZINC000003814395 | -4.327 |
| ZINC000001530641 | -4.327 |
| ZINC000000901555 | -4.323 |
| ZINC000000001115 | -4.321 |
| ZINC000004658557 | -4.314 |
| ZINC000004212809 | -4.29 |
| ZINC000004658560 | -4.288 |
| ZINC000003927822 | -4.287 |
| ZINC000003927822 | -4.287 |
| ZINC000019166991 | -4.279 |
| ZINC000100003902 | -4.278 |
| ZINC000100003902 | -4.278 |
| ZINC000000000494 | -4.271 |
| ZINC000004474460 | -4.258 |
| ZINC000004095858 | -4.25 |
| ZINC000052955754 | -4.248 |
| ZINC000052955754 | -4.248 |
| ZINC000052955754 | -4.248 |
| ZINC000019875504 | -4.244 |
| ZINC000000967566 | -4.218 |
| ZINC000003776875 | -4.216 |
| ZINC000003780893 | -4.206 |
| ZINC000003875342 | -4.204 |
| ZINC000001530701 | -4.199 |
| ZINC000018115268 | -4.199 |
| ZINC000018115268 | -4.199 |
| ZINC000000014007 | -4.19 |
| ZINC000003843378 | -4.189 |
| ZINC000003914809 | -4.182 |
| ZINC000000155531 | -4.169 |
| ZINC000000155531 | -4.169 |
| ZINC000070466416 | -4.159 |
| ZINC000000967521 | -4.154 |
| ZINC000001530968 | -4.149 |
| ZINC000019632618 | -4.14 |
| ZINC000019632618 | -4.14 |
| ZINC000019632618 | -4.14 |
| ZINC000000897002 | -4.139 |
| ZINC000000897002 | -4.139 |
| ZINC000000897002 | -4.139 |
| ZINC000003798537 | -4.137 |
| ZINC000001530968 | -4.122 |
| ZINC000036766734 | -4.106 |
| ZINC000004212945 | -4.093 |
| ZINC000004097305 | -4.086 |
| ZINC000006409735 | -4.084 |
| ZINC000006409735 | -4.083 |
| ZINC000001530930 | -4.077 |
| ZINC000001530930 | -4.074 |
| ZINC000000896666 | -4.072 |
| ZINC000003800008 | -4.064 |
| ZINC000003800008 | -4.064 |
| ZINC000100013500 | -4.054 |
| ZINC000100013500 | -4.054 |
| ZINC000005456939 | -4.046 |
| ZINC000001530812 | -4.039 |
| ZINC000001530812 | -4.039 |
| ZINC000001530812 | -4.039 |
| ZINC000000010163 | -4.032 |
| ZINC000000010163 | -4.032 |
| ZINC000000000128 | -4.029 |
| ZINC000000967520 | -4.019 |
| ZINC000002016258 | -4.017 |
| ZINC000003201907 | -4.002 |
| ZINC000003201907 | -4.002 |
| ZINC000003812984 | -3.983 |
| ZINC000003812984 | -3.983 |
| ZINC000002019954 | -3.972 |
| ZINC000002019954 | -3.971 |
| ZINC000000057534 | -3.968 |
| ZINC000100070954 | -3.968 |
| ZINC000004632106 | -3.966 |
| ZINC000004632106 | -3.966 |
| ZINC000008214625 | -3.965 |
| ZINC000086040406 | -3.955 |
| ZINC000004097392 | -3.951 |
| ZINC000000537791 | -3.948 |
| ZINC000003882036 | -3.946 |
| ZINC000000968345 | -3.94 |
| ZINC000000004413 | -3.932 |
| ZINC000000004413 | -3.932 |
| ZINC000017285869 | -3.928 |
| ZINC000001530762 | -3.915 |
| ZINC000001530762 | -3.915 |
| ZINC000012503076 | -3.896 |
| ZINC000001543873 | -3.893 |
| ZINC000001543873 | -3.893 |
| ZINC000000002176 | -3.883 |
| ZINC000003978005 | -3.873 |
| ZINC000003978005 | -3.873 |
| ZINC000003978005 | -3.873 |
| ZINC000014881137 | -3.839 |
| ZINC000001530820 | -3.827 |
| ZINC000000968256 | -3.824 |
| ZINC000003008621 | -3.815 |
| ZINC000003977981 | -3.811 |
| ZINC000014210457 | -3.81 |
| ZINC000008214573 | -3.806 |
| ZINC000000895316 | -3.806 |
| ZINC000001530810 | -3.8 |
| ZINC000001529425 | -3.793 |
| ZINC000000006300 | -3.785 |
| ZINC000003914808 | -3.743 |
| ZINC000068202099 | -3.678 |
| ZINC000018089317 | -3.654 |
| ZINC000004097286 | -3.629 |
| ZINC000008214619 | -3.624 |
| ZINC000004074875 | -3.61 |
| ZINC000004074875 | -3.61 |
| ZINC000001530940 | -3.587 |
| ZINC000001530940 | -3.587 |
| ZINC000001530613 | -3.58 |
| ZINC000001530613 | -3.58 |
| ZINC000003938751 | -3.569 |
| ZINC000000403618 | -3.533 |
| ZINC000003830716 | -3.504 |
| ZINC000003830716 | -3.504 |
| ZINC000004212854 | -3.49 |
| ZINC000018115268 | -3.454 |
| ZINC000018115268 | -3.454 |
| ZINC000000895048 | -3.44 |
| ZINC000001633889 | -3.408 |
| ZINC000004097308 | -3.364 |
| ZINC000000537928 | -3.363 |
| ZINC000002019953 | -3.349 |
| ZINC000002019953 | -3.348 |
| ZINC000001532805 | -3.312 |
| ZINC000000895318 | -3.292 |
| ZINC000003798750 | -3.289 |
| ZINC000003993846 | -3.258 |
| ZINC000019594594 | -3.189 |
| ZINC000019594594 | -3.189 |
| ZINC000001530717 | -3.155 |
| ZINC000001633887 | -3.086 |
| ZINC000001530951 | -3.016 |
| ZINC000001530950 | -3.012 |
| ZINC000019364242 | -3.001 |
| ZINC000019364242 | -3.001 |
| ZINC000005029557 | -2.959 |
| ZINC000003079337 | -2.946 |
| ZINC000001530718 | -2.896 |
| ZINC000006845963 | -2.859 |
| ZINC000004474603 | -2.845 |
| ZINC000036701290 | -2.819 |
| ZINC000036701290 | -2.819 |
| ZINC000017285872 | -2.728 |
| ZINC000006920384 | -2.711 |
| ZINC000000538283 | -2.692 |
| ZINC000000538283 | -2.692 |
| ZINC000000896523 | -2.67 |
| ZINC000001530811 | -2.637 |
| ZINC000001530811 | -2.637 |
| ZINC000001532525 | -2.58 |
| ZINC000001532179 | -2.523 |
| ZINC000004097427 | -2.209 |
| ZINC000004097427 | -2.209 |
| ZINC000000000257 | -2.138 |
| ZINC000001554392 | -2.11 |
| ZINC000001554392 | -2.106 |
| ZINC000001530303 | -2.026 |
| ZINC000019364219 | -1.869 |
| ZINC000019364219 | -1.859 |
| ZINC000006845860 | -1.762 |
| ZINC000000901159 | -1.742 |
| ZINC000004978673 | -1.425 |
| ZINC000001531036 | -0.184 |
| ZINC000019363537 | -0.012 |
| ZINC000019363537 | -0.006 |
| ZINC000000896409 | 0.268 |
| ZINC000000896409 | 0.268 |
| ZINC000001612996 | 0.31 |
| ZINC000026011099 | 0.496 |
| ZINC000026011099 | 0.496 |
| ZINC000008437287 | 0.756 |
| ZINC000003938652 | 3.298 |
